# A scoping review of the problems and solutions associated with contamination in trials of complex interventions in mental health

**DOI:** 10.1186/s12874-018-0646-z

**Published:** 2019-01-07

**Authors:** Nicholas Magill, Ruth Knight, Paul McCrone, Khalida Ismail, Sabine Landau

**Affiliations:** 1 Department of Biostatistics and Health Informatics, Institute of Psychiatry, Psychology and Neuroscience, Kings College London, 16 De Crespigny Park, London, SE5 8AF United Kingdom; 2 Department of Medical Statistics, London School of Hygiene and Tropical Medicine, London, UK; 3 Department of Health Service and Population Research, Institute of Psychiatry, Psychology, and Neuroscience, King’s College London, London, UK; 4 Institute of Diabetes, Endocrinology and Obesity, King’s Health Partners, London, UK

**Keywords:** Treatment contamination, Randomised controlled trials, Mental health, Complex interventions

## Abstract

**Background:**

In a randomised controlled trial, contamination is defined as the receipt of active intervention amongst participants in the control arm. This review assessed the processes leading to contamination, its typical quantity, methods used to mitigate it, and impact of use of cluster randomisation to prevent it on study findings in trials of complex interventions in mental health.

**Methods:**

This is a scoping review of trial design approaches and methods of study conduct to address contamination. Studies included were randomised controlled trials of complex interventions in mental health that described the process leading to, amount of, or solution used to counter contamination. The Medline, Embase, and PsycInfo databases were searched for trials published between 2000 and 2015. Risk of bias was assessed using the Jadad score and domains recommended by Cochrane plus some relevant to cluster randomised trials.

**Results:**

Two hundred and thirty-four articles were included in the review. The main processes that led to contamination were health professionals delivering both active and comparator treatments and communication among clinicians and participants from the different trial arms. Twenty-three trials (10%) measured binary treatment receipt in the control arm with median 13% of participants found to be contaminated (IQR 5–33%). The most common design approach for dealing with contamination was the use of cluster randomisation (*n* = 93). In addition, many researchers used simple trial conduct methods to minimise contamination due to suspected contamination processes, such as organising for each clinician to provide only one treatment and separating trial arms spatially or temporally. There was little evidence for a relationship between cluster randomisation to avoid contamination and size of treatment effect estimate.

**Conclusion:**

There was some evidence of modest levels of treatment contamination with a large range, although a minority of studies reported the amount of contamination. A limitation was that many trials described the problem in little detail. Overall there is a need for greater measurement and reporting of treatment receipt in the control arm of trials. Researchers should be aware of trial conduct methods that can be used to minimise contamination without resorting to cluster randomisation.

**Electronic supplementary material:**

The online version of this article (10.1186/s12874-018-0646-z) contains supplementary material, which is available to authorized users.

## Background

Treatment contamination is defined as the receipt of active intervention amongst participants in the control arm of a randomised controlled trial (RCT) [1]. It is thought to be particularly prevalent in RCTs of complex interventions in mental health. Psychological therapies are complex interventions that comprise several interacting constituent parts [2]. Such intervention components are often transportable and difficult to confine, meaning that their receipt by participants within the control arm is possible. The effect of contamination is to make the control arm more similar to the active intervention arm, i.e. to dilute the treatment contrast. This is a concern to researchers because the contrast between the randomised groups (intention-to-treat estimator) will be biased for the effect of treatment receipt (efficacy).

The processes leading to contamination in trials of complex interventions in mental health have never been reviewed comprehensively and the literature is unclear about their relative frequencies. This is necessary in order to plan what steps should be taken to address the problem. In mental health the typical quantity of contamination, some of the methods that researchers take to minimise or prevent it, and the extent to which it impacts on study findings are either little known or poorly formalised within the literature. Here we undertake a comprehensive scoping review that addresses these points. This type of review is defined as a map of literature within a research area that identifies key concepts; gaps in research; and types and sources of evidence, in order to inform practice, policymaking and research [3].

Previous reviews have assessed the extent of contamination in certain areas of medicine. For example, a literature review of 235 RCTs of guideline dissemination and implementation strategies for healthcare professionals identified eight trials that quantified contamination [1]. The review assessed the proportion of participants in the control arm who were considered to have received treatment and found a median of 24% of participants to be contaminated (range 0–65%). In oncology, a large breast cancer screening trial (*n* = 9780) found that 22% of those in control arm received a mammogram outside the trial compared to 5% of the intervention group doing likewise [4]. A review of cancer trials using Zelen’s design, where patients consent to their randomly allocated treatment before being asked for consent to participate in the study, found 11 trials that reported the number of patients who switched treatments [5, 6]. The average was 18% (range 10–36%). However, this figure did not represent solely contamination as many of the studies in the review described switches from active to comparator treatments or provided an overall summary of switches in either direction. The scale of the problem in mental health trials remains unclear.

In terms of steps that might be undertaken to minimise the occurrence of contamination, we distinguish between statistical design methods, trial conduct solutions, and analytical approaches. The main statistical design method is the use of cluster randomisation, which can prevent contamination provided that clusters are constructed at the level at which it takes place [7]. By ensuring that all participants within a cluster receive the same treatment, contamination of the control condition due to participants being affected by each other’s treatment receipt can be avoided. However, the cost of such a design is that correlation within clusters must be factored into a power calculation and will inflate the sample size requirement. Sample size is inflated by design factor, *D*.

$$ D:= 1+I\left(k-1\right) $$

where *I* is the intraclass correlation coefficient and *k* is the cluster size. In addition, cluster randomised controlled trials (cRCTs) often suffer from selection biases, mainly due to treatment being known before participant entry into trial (recruitment bias) and differential loss to follow-up between trial arms (attrition bias) [8]. The second type of strategy (trial conduct solutions) relates to methods that can be used in the running of the trial to reduce exposure of the control arm to active intervention. Education of clinicians and participants against contamination and provision of clear information about the purposes of the trial have been suggested [1]. However, it remains an open question as to what methods researchers use in practice. In terms of analytical methods that adjust for contamination, the use of modern causal estimation techniques has been advocated for this purpose [9–11], but it is not known how widely these have been used.

The review of educational interventions in RCTs also assessed whether there was any link between the prevention of contamination (e.g. through use of cluster randomisation) and an increase in the size of treatment effect estimates [1]. When assessing all studies in the set there was no evidence for such a link; however, a more homogeneous sample showed some evidence of a relationship. Other reviews have found similarly mixed results. For example, a review of 14 hip protector trials showed large positive effects in cluster RCTs and a mixture of positive and negative effects in RCTs with individual-level randomisation with suspected contamination [12]. On the other hand, a meta-analysis of 34 RCTs of enhanced care for depression found very similar treatment effect sizes when comparing cRCTs with individual-randomised RCTs [13]. One particular statistical design approach may provide extra information about the link between contamination and estimated treatment effect sizes. Specifically, in trials that use treatment allocation at more than one level, a comparison of treatment effect estimates between cluster- and participant-randomised sub-trials may provide some information as to the impact of contamination or the ability of cluster randomisation to prevent it.

The aims of this article were fourfold: to identify the processes that are considered to lead to contamination in trials of complex interventions in mental health, to quantify typical levels of contamination, to summarise what researchers do in order to prevent or mitigate it, and to compare treatment effect estimates within trials of complex interventions that used both cluster- and individual-level treatment allocation to quantify the contamination bias.

## Methods

### Type of review

We carried out a scoping review of trial design and conduct methods in RCTs of complex interventions in mental health. This type of review was chosen on the basis that our objectives were to summarise researchers’ perceptions of and solutions to a trial design problem where there is limited literature and potentially highly heterogeneous evidence. This was a methodology review and did not focus on a particular patient outcome, therefore was not eligible for registration with PROSPERO.

### Eligibility criteria

All articles were screened using full texts and were assessed using five inclusion criteria. First, the text described a trial purporting to have used random allocation. Second, the intervention was complex, which in this review meant it comprised multiple components. It was not possible to assess whether these elements acted together to provide some added benefit (as per MRC guidance definition) so we used a general and therefore wide definition for this. Third, the publication gave some information about the process leading to, amount of, or solution used to counter treatment contamination. Fourth, the abstract and main body of the article were written in English. And finally, the trial was related to mental health, psychology, or psychiatry – this meant that a minimum of one of the target population, intervention, or primary outcome was directly related to one of these fields. Many trials in these fields test unblinded treatments where the suspicion is that they may be subject to contamination. The scoping review was limited to these areas of medicine for this reason and because of the apparent gap in the literature surrounding contamination in these fields.

### Information sources

The search for contamination in RCTs of complex interventions in mental health was done using the Ovid platform and included the databases Medline, Embase, and PsycInfo. Articles that were published between January 2000 and April 2015 were searched. Results were restricted to those articles published after 2000 because this was the year when the first MRC framework paper on complex interventions was first published [14]. The publication of this framework marked the point at which the design and evaluation of complex interventions were formalised.

### Search

Randomised controlled trials were searched for using the sensitivity-maximising 11-step process recommended by Cochrane [15]. The search terms “contamination” and “spillover” were included in the procedure. Synonymous terms for complex interventions that were used included all combinations of “multicomponent”, “psychosocial”, and “behavioural”, with “interventions”, “treatments”, and “training”. The search was restricted to articles that mentioned “mental health”, “psychology”, or “psychiatry”. All terms were searched for in the main body of the text. The full search procedure can be found in the supplementary materials (see file Additional file 1).

### Study selection

Duplicates were removed from the set and the remaining articles were assessed for each of the exclusion criteria. Any potentially relevant article that was referred to by a paper in the results of the search and was not already in the set was followed up by a single author (NM). If the article was judged to have met the inclusion criteria it was included in the set and the full text was reviewed, also by a single author (NM). In order to assess the reliability of study selection, a second reviewer (RK) re-screened 70 articles (11%).

### Data collection process

Any studies that were included in the review that featured sub-studies that used both cluster- and individual-level treatment allocation were reviewed as two separate sub-trials because of the different contamination processes and methods used to address these. Treatment effect sizes were extracted for trials that reported effects separately depending on the level of treatment allocation. Data from any such studies that did not report results at the different levels of treatment allocation were obtained from the authors in order to allow the comparison.

### Data items

Abstracted data included an assessment of bias, summaries of trial design (e.g. study population, intervention, primary outcome, unit of treatment allocation), details about contamination (e.g. how it was thought to take place, its quantity, steps taken to avoid it), and records of trial summaries (e.g. extent of clustering, power, sample size, treatment effect). In order to assess the reliability of data abstraction, a second reviewer (RK) re-extracted data from 20 articles (8%) using the same procedure described above.

### Risk of bias in individual studies

The review of trial bias included recording the “Jadad score” (a single item measure of methodological quality of RCTs [16]) and most of the domains of Cochrane’s classification scheme for bias [17]. In addition to these, some other domains that were pertinent to cluster randomised trials were used. These included whether randomisation occurred after participant consent was obtained, baseline measures were completed before randomisation, baseline outcome measurements were similar across trial arms, other clinical and demographic characteristics were similar across arms, and whether attrition was similar in the arms. These additional assessments of bias were based on outcomes used in a review of cRCTs [8].

## Results

### Reliability

At the screening stage agreement was 71%; all discrepancies were discussed and subsequently resolved. Agreement was 81% for all assessments of bias, and 82% for details of contamination processes.

### Summary of trials

**Fig. 1:**
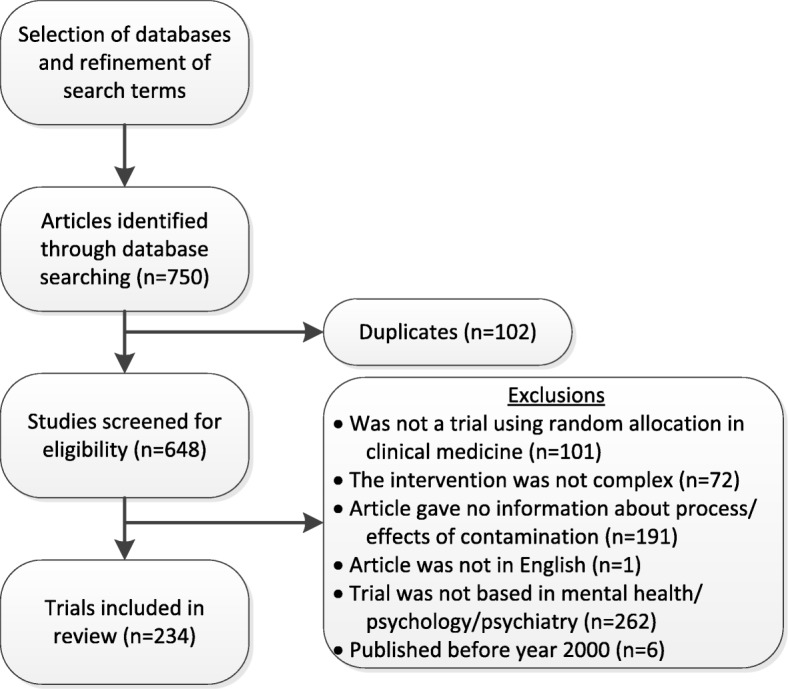
Flow diagram for searching for relevant articles (articles could be excluded for more than one reason)

Two hundred and thirty-four studies were identified as meeting the eligibility criteria. This included seven trials that were referred to by an article in the main search and were found to meet the eligibility criteria. The results of the implementation strategy and numbers of exclusions are summarised in Fig. 1. Four hundred and fourteen articles were excluded. A list of these articles together with reasons for exclusions can be found in the supplementary materials (see Additional file 2).

Details of the 234 trials in the review are given in Table 1. The table shows that the overwhelming majority of articles described the primary analysis of an RCT, were based in either North America or Western Europe, and were late phase (i.e. not pilot or feasibility trials). Most target populations were adult patients and the most commonly targeted conditions were depression, substance abuse, and psychosis. The two most common interventions were cognitive behavioural therapy and care management; there were many small categories. The sample size of participant randomised trials ranged from 16 to 14,910; that of cluster randomised trials ranged from 13 to 6076. A full list of references can be found in the supplementary materials (see Additional file 3).

**Table 1:** Summary of characteristics of articles

Variable	Level	Number of articles
Type of article (n)	Results of primary analysis of clinical trial	228 (97.4%)
	Design / protocol of clinical trial	3 (1.3%)
	Results of secondary analysis of clinical trial	3 (1.3%)
Year (n)	2000–2004	47 (20.0%)
	2005–2009	85 (36.3%)
	2010–2015	102 (43.6%)
Country of origin (n)	USA	101 (43.2%)
	UK	39 (16.7%)
	Netherlands	19 (8.1%)
	Canada	14 (6.0%)
	Australia	11 (4.7%)
	Other	50 (21.4%)
Target population (n)	Adult patients	172 (73.5%)
	Children / adolescent patients	45 (19.2%)
	People at risk	5 (2.1%)
	Workers	12 (5.1%)
Target condition (n)	Depression	30 (28.6%)
	Substance abuse	18 (17.1%)
	Psychosis	14 (13.3%)
	Neurodegeneration	13 (12.4%)
	Anxiety	6 (5.7%)
	Attention deficit hyperactivity disorder	5 (4.8%)
	Others	19 (18.1%)
Intervention (n)	Cognitive behavioural therapy / CBT skills	33 (14.1%)
	Care management / interdisciplinary care	26 (11.1%)
	Education	21 (9.0%)
	Motivational interviewing / motivational enhancement therapy	19 (8.1%)
	Other psychotherapy / counselling	16 (6.8%)
	Assessment and feedback	8 (3.4%)
	Parenting interventions	8 (3.4%)
	Others	103 (44.0%)
Phase (n)	Early (pilot and feasibility trials)	29 (12.4%)
	Late	205 (87.6%)
Level of treatment allocation (n)	Participant level	141 (60.3%)
	Cluster level	93 (39.7%)
Sample size (median; IQR)	Participant-level allocation	143 (84–261)
	Cluster-level allocation	285 (158–579)
Cluster size in cRCTs (median; range)		10 (3–200)

### Summary of assessment of bias

Summaries of assessments of bias using the Jadad scale, items recommended by Cochrane, and items aimed at identifying possible bias in cluster randomised trials are reported in Table 2. The table demonstrates the potential for bias split by the level of treatment allocation. In general, it shows that the greatest potential for bias arose due to lack of knowledge of allocation, incomplete outcome data, differences in attrition between trial arms, and randomisation occurring before consent was obtained and before baseline measures were completed. More evidence for bias was found in cluster randomised trials when assessing whether randomisation took place after consent and after baseline measures were completed, whether outcome assessment was blind, and whether baseline demographic characteristics were similar between trial arms. Assessments of bias for each trial together with the support for judgement associated with each assessment can be found in the supplementary materials (see Additional file 3).

**Table 2:** Summary of assessment of bias

Variable	Level	Trials with individual-level randomisation (n)	Trials with cluster randomisation (n)
Jadad score (possible range of 0–5; higher scores indicate lower likelihood of bias)	0	2 (1.4%)	1 (1.1%)
	1	26 (18.8%)	22 (23.9%)
	2	44 (31.9%)	32 (34.8%)
	3	66 (47.8%)	37 (40.2%)
Allocation sequence adequately generated	Low risk	75 (53.2%)	44 (47.3%)
	High risk	3 (2.1%)	1 (1.1%)
	Unclear	63 (44.7%)	48 (51.6%)
Allocation sequence adequately concealed	Low risk	30 (21.3%)	18 (19.3%)
	High risk	3 (2.1%)	1 (1.1%)
	Unclear	108 (76.6%)	74 (79.6%)
Randomisation after consent obtained	Low risk	116 (82.3%)	23 (24.7%)
	High risk	6 (4.3%)	39 (41.9%)
	Unclear / NA	19 (13.5%)	31 (33.3%)
Randomisation after baseline measures were completed	Low risk	56 (39.7%)	15 (16.1%)
	High risk	14 (9.9%)	41 (44.1%)
	Unclear	71 (50.4)	37 (39.8%)
Baseline outcome measurements similar across trial arms	Low risk	109 (77.3%)	70 (75.3%)
	High risk	9 (6.4%)	11 (11.8%)
	Unclear / NA	23 (16.3%)	12 (12.9%)
Baseline demographic characteristics similar across trial arms	Low risk	121 (85.8%)	69 (74.2%)
	High risk	3 (2.1%)	18 (19.4%)
	Unclear / NA	17 (12.1%)	6 (6.5%)
Knowledge of allocation adequately concealed	Low risk	0 (0%)	0 (0%)
	High risk	141 (100%)	93 (100%)
Blinded outcome assessment	Low risk	63 (44.7%)	29 (31.2%)
	High risk	8 (5.7%)	17 (18.3%)
	Unclear / NA	70 (49.6%)	47 (50.5%)
Incomplete outcome data	Low risk	35 (24.8%)	22 (23.7%)
	High risk	82 (58.2%)	57 (61.3)
	Unclear / NA	24 (17.0%)	14 (15.1%)
Similar attrition between trial arms	Low risk	85 (60.3%)	51 (54.8%)
	High risk	30 (21.3%)	25 (26.9%)
	Unclear / NA	26 (18.4%)	17 (18.3%)

### Processes driving contamination

There were perceived to be five main processes that led to contamination. The first two processes, staff delivering the active intervention in the control arm (*n* = 82, 35%) and communication between trial arms (*n* = 79, 34%), were the most common. Staff delivering the active intervention in the control arm happened either due to a given clinician delivering both the active and control treatments (*n* = 76, e.g. [18]) or due to control participants being exposed to the intervention as a consequence of clinicians, who were not directly involved in providing the treatment, treating participants in both arms and thereby potentially learning about the active intervention and passing this on to participants in the control arm (*n* = 6, e.g. [19]). The other main contamination process was communication between individuals in different trial arms. This could be either at the level of the clinician (*n* = 20, e.g. [20]), participant (*n* = 57, e.g. [21]), or both (*n* = 2). Communication between providers of interventions was often a worry in environments in which the people giving the treatment worked closely together, for example GP surgeries, hospital units, and schools. Communication between participants was thought to be most likely in environments in which participants came into close contact. Examples of this included interaction between participants who were family members, patients in a waiting room, school children, employees working on the same worksite, and university students. Particular healthcare settings that were thought to be highly likely to foster communication were antenatal clinics/childbirth classes, specialist clinics (e.g. substance misuse, dialysis), and wards for those admitted to hospital.

There were perceived to be three other, more minor processes that drove contamination. First, participants switching clinicians (*n* = 4, 2%, e.g. [22]), where control participants were treated by multiple clinicians of whom one was trained in the active intervention. Second, participants seeking treatment outside the trial (*n* = 6, 3%, e.g. [23]). And finally, background noise, where the treatment already existed to some extent within the healthcare system (*n* = 5, 2%, e.g. [24]). Fifty-nine articles did not provide information about the contamination process.

### Quantity of contamination

Twenty-seven studies (12%) attempted to quantify contamination. Twenty-three trials measured individual-level contamination on a binary scale and summaries of these quantities are given in Table 3. The median level of contamination was 13% (IQR 5–33%).

**Table 3:** Quantifying treatment contamination where treatment receipt was defined as binary

Reference	Control treatment	Active intervention	Measure of contamination	Contamination (control participants receiving intervention)
Aveyard et al. [40]	Basic behavioural support for smoking cessation	Behavioural support for smoking cessation	Nurse visit (1st extra); Telephone call; Nurse visit (2nd extra)	12/469 (3%) 12/469 (3%) 5/469 (1%)
Barton et al. [41]	No treatment	Mammography education (pamphlet and videotape) focusing on anxiety	Patient recall of: Pamphlet; Videotape	9% 1%
Bernstein et al. [42]	No treatment	Cognitive behavioural therapy	Service Questionnaire of anxiety treatment	0/24 (0%)
Borland et al. [43]	Minimal information	Behavioural support	Patients reporting use of extensive behavioural support	45/378 (12%)
Clarkson et al. [36]	Routine care	Self-efficacy education	Participants reporting use of electric toothbrush	9/113 (8%)^p^ 9/180 (5%)^c^
Courneya et al. [44]	Group psychotherapy	Group psychotherapy and exercise programme	Patient-reported exercise	10/45 (22%)
Dilley et al. [45]	Usual care	Cognitive counselling	Patient-reported receipt of counselling	45/158 (29%)
Forchuk et al. [46]	Usual care	Transitional discharge from hospital	Patient-reported receipt of peer support and staff contact	27%
Heirich & Sieck [47]	Health education	Proactive follow-up counselling	Patients requesting personal counselling	56%
Johnson et al. [20]	Usual treatment	Clinical training in dual diagnosis of psychosis and substance misuse	Patients not taken on by trained case manager	19/105 (18%)
Khumalo-Sakutukwa et al. [48]	Standard HIV voluntary counselling and testing	HIV counselling, testing and self-management	Participants seeking out treatment from intervention centres	1%
Lamers et al. [49]	Usual care	Nurse-led minimal psychological intervention (MPI)	Patients who reported knowledge of MPI	9/178 (5%)
Lee & Gay^p^ [37]	Attention control	Sleep hygiene package	Patient-reported use of: Bassinet; White noise device; Low lighting	33/46 (72%)^p^ 47/75 (62%)^c^ 11/75 (14%)^c^ 27/75 (36%)^c^
Merritt et al. [50]	No intervention	Postcards with information about depression	Patients reporting having seen the postcards	7/78 (1%)
Moadel et al. [51]	Standard care	Smoking cessation group support and encouragement	Patients reporting discussion of active intervention patients; Patients reporting familiarity with program’s strategies	6% 17%
Mohr et al. [52]	Treatment as usual	Cognitive behavioural therapy	Patients who had contact with non-study therapist	18/44 (41%)
Phillips et al. [53]	Routine public health practice	Community engagement in healthy eating	Participants reporting participation in intervention programme	1%
Saitz et al. [54]	Usual care	Chronic care management (multidisciplinary care coordination; motivational therapy; counselling)	Patients who received a session of motivational enhancement therapy	9/281 (3%)
Shemilt et al. [55]	No funding for breakfast club	Funding for school-based breakfast club	School pupils with school breakfast club	77%
Stewart-Brown et al. [56]	No intervention	Incredible Years (parenting techniques) training	Participants attending community-based parenting programme	4/44 (9%)
Waghorn et al. [57]	Enhanced routine mental health case management	Supported employment and specialist illness management	Patients opting to transfer to intervention after 6 months	28/102 (27%)
Walpole et al. [58]	Social skills training	Motivational interviewing (MI)	Patients whose treatment was MI adherent	37%
Wells et al. [59]	Usual care	Quality improvement therapy (CBT) and medications (assessment and education)	Receipt of speciality counselling within 6 months	13%

^p^Using participant-level treatment allocation

^c^Using cluster-level treatment allocation

Four trials measured contamination using a continuous scale; three were trials of cognitive behavioural therapy and one of cognitive analytic therapy. One created a treatment fidelity scale and asked participants in each trial arm (behavioural weight control instructions, cognitive behavioural therapy, standard counselling) about their knowledge of all three treatments at the beginning and end of treatment [25]. The sub-scales showed high knowledge of behavioural weight control in the group allocated to receive behavioural weight control instructions (mean change of 1.1 compared to 0.5 and 0.5 in cognitive behavioural therapy and standard arms), high knowledge of cognitive behavioural therapy in those allocated to receive this (mean change of 1.6 compared to 0.0 and 0.8 in behavioural weight control and standard groups), and high knowledge of standard intervention in the control group (mean change of 0.5 compared to 0.1 and 0.1 in behavioural weight control and cognitive behavioural therapy arms). This seemed to indicate receipt of treatment in the control arm. Three RCTs showed negligible evidence of treatment contamination. Of these RCTs, one used a cognitive behavioural therapy adherence scale (adapted CTACS) to record adherence and contamination in the active intervention and control arms [26]. The CTACS means were 98.0 and 98.8 in the cognitive behavioural therapy and education intervention (control) arms respectively, indicating that contamination did not occur. Another trial found that the family-focused cognitive behavioural therapy (FCBT; active intervention) group scored higher than the traditional child-focused cognitive behavioural therapy (control) group on two scales, Family Focus (mean = 4.90 and 1.55) and Parenting Style Focus (mean = 4.75 and 1.00) [27]. This suggested that only the FCBT group incorporated family and parenting interventions and therefore that there was little evidence of contamination. The fourth trial used a scale to measure the fidelity of the control intervention, which was good clinical care [28]. This scale included a sub-scale for cognitive analytic therapy and the mean for this was very low: 0.52 (SD 0.11). This represented negligible contamination.

### Solutions used to counter contamination

Methods that were used to counter contamination are summarised in three categories: statistical design, trial conduct, and analysis methods. Statistical design includes the use of cluster randomisation, where clusters are chosen based on groups of participants who are thought potentially to become contaminated by direct or indirect links (e.g. via a shared therapist). One trial inflated the sample size in order to account for reduced statistical power caused in part by contamination bias [29]. The great majority of other methods for preventing contamination were aspects of trial conduct, such as recruitment of more clinicians to ensure that each clinician only delivered one of the interventions. In terms of analysis methods, one trial used per protocol analysis, meaning that participants whose treatment was contaminated were dropped from the analysis [30]. This review found no trials that addressed the problem of contamination by using methods from the causal inference field.

Categorisations of trial conduct solutions that were used to avoid treatment contamination can be found in Table 4. The sections of the table match the processes of contamination described in the earlier section on this. The majority of solutions used to prevent contamination related either to preventing staff delivering the active intervention in the control arm or preventing communication between clinicians or participants.

**Table 4:** Trial conduct solutions to treatment contamination

Process driving contamination	Trial conduct solution	Number of papers
Clinicians deliver both active and control treatments	Recruiting groups of clinicians, each one of which is responsible for a single treatment	16
	Monitoring contamination using supervision/therapy session recordings	10
	Formalising differences between interventions, e.g. using structured manual during therapist training	6
	Asking clinicians not to use intervention content when treating those in control arm	3
	Providing active intervention within the research project rather than health service	1
	Using a script for contact with control participants during treatment	1
Clinicians not involved in active intervention treating participants in both trial arms	Blinding usual care clinicians	4
	Confining intervention to provision by specialist clinicians	2
Communication between clinicians in different trial arms	Asking clinicians not to share details of the intervention with each other	5
Communication between participants in different trial arms	Holding treatment sessions at different times / in different locations	13
	Staggering the scheduling of data collection appointments / reducing waiting time so that participants do not meet in waiting room	3
	Allocating separate therapists / modes of delivery for individual and group therapies when usual group therapy was shared by participants in both arms	2
	Asking participants not to share contents of intervention with others	2
	Excluding potential participants who know someone else attending screening	2
	Holding separate sessions of existing group treatments for participants in separate trial arms in order to prevent contact	1
	Restricting the release of intervention materials in order to reduce the chance of their being shared with control participants	1
	Recruiting participants in blocks and providing one treatment at a time, with no new participants recruited during the final week of each period in order to maintain separation between trial arms	1
Participants switching clinicians and therefore trial arms	Preventing referrals for add-on care by clinicians who are members of study team	1
	Avoiding transfer of participants between clinicians	1
Participants seeking treatment outside the trial	Informing participants only about the treatment they were allocated to receive (Zelen’s design)	8
	Promising the intervention to control participants at the end of follow-up	2
Active treatment is available to some extent within the healthcare system	Making intervention distinct from usual care by adapting one or other	2
	Establishing common treatment for all participants	1
	Excluding institutions that already offer some aspect of the intervention	1

Four trials were concerned about contamination during data collection and aimed to prevent this by minimising interaction between researchers and participants [31–34]. Another temporally separated the control and active treatments with data collection following each. This meant that treatment could only influence data from active intervention participants [35].

### Trials using both cluster- and participant-level treatment allocation

**Fig. 2:**
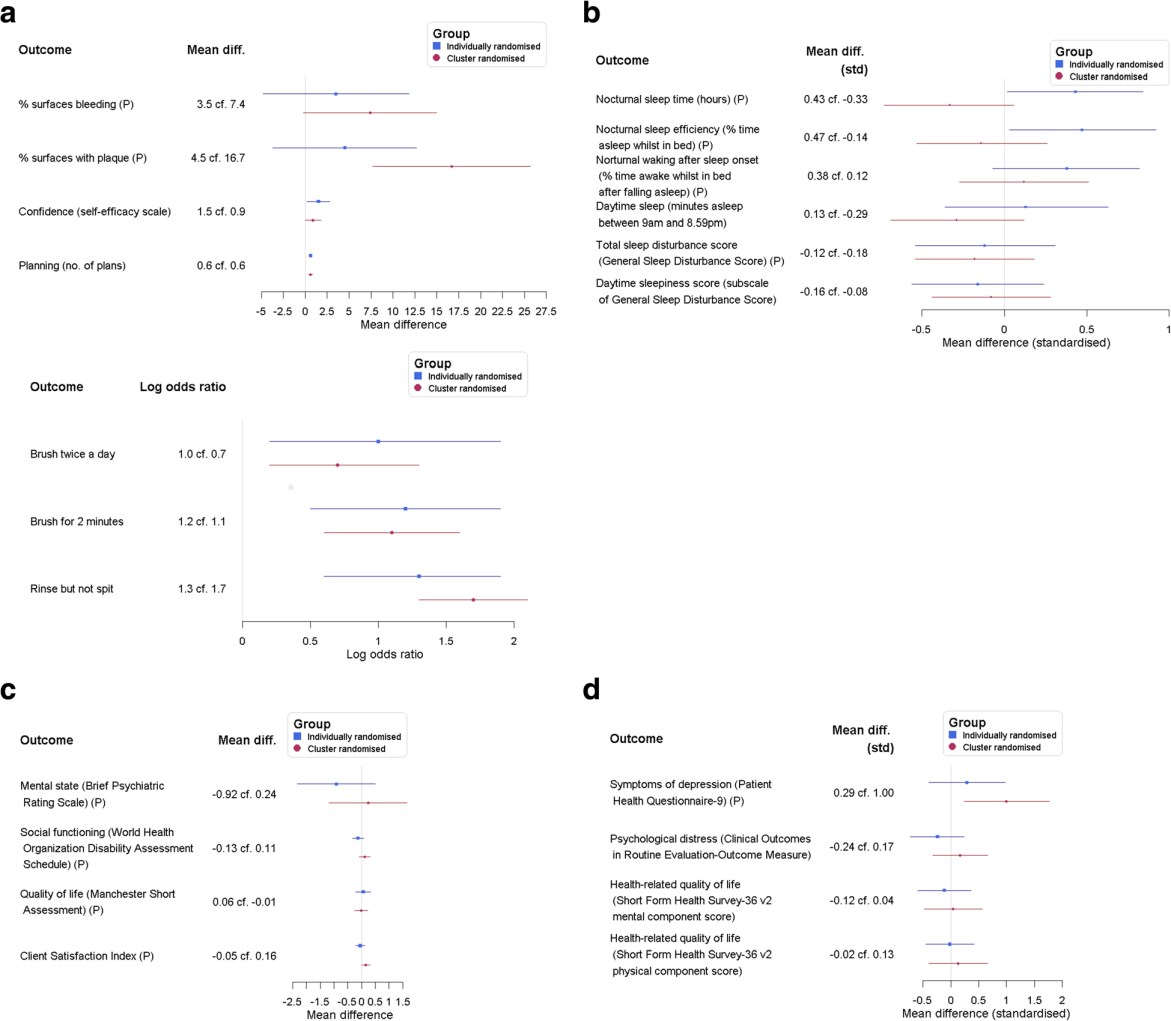
Forest plots for four trials that used both individual- and cluster-level randomisation; (P) = primary outcome. a) Clarkson et al. (2009) [36]. Choice of primary outcomes is based on sample size calculation; estimates are adjusted for baseline measures. Larger (more positive) treatment effects indicate benefit. b) Lee & Gay (2011) [37]. Estimates were standardised and calculated from summaries of means and SDs (mothers’ scores only). Larger (more positive) treatment effects indicate benefit. c) Marshall et al. (2004) [38]. Estimates used same adjustments as in the trial publication. Larger (more positive) treatment effects indicate benefit. d) Richards et al. (2008) [39]. Estimates were standardised and calculated from summaries of means and SDs. Larger (more positive) treatment effects indicate benefit

The results of the review included four trials that used both participant- and cluster-level treatment allocation [36–39]. Treatment effect estimates and confidence intervals for these trials are shown in Fig. 2. The figure shows treatment effects arranged such that greater benefit (or less harm) of treatment is represented by a greater number on the horizontal axis. The figure enables the comparison of the absolute size of treatment effect between participant- and cluster-level allocation to assess the impact of contamination on effect size estimation. Of the 21 outcomes investigated, just under half of outcomes showed a difference in the anticipated direction, i.e. smaller estimated absolute effect sizes under participant-level random allocation. In particular, an attenuated treatment effect (lesser distance from the null line in Fig. 2) was found under participant-level allocation in eight out of 21 outcomes with a tie in one outcome.

## Discussion

The review identified 234 articles that described either the processes driving treatment contamination, its quantity, or steps that researchers took to prevent or alleviate the problem in trials of complex interventions in mental health. The principal processes leading to contamination were found to be clinicians being required to treat participants in both treatment and control conditions and communication among clinicians or participants in different trial arms. Typically, around one in eight participants in the control arm of a trial were assessed as having received the active intervention. The most common steps that researchers took to prevent or mitigate contamination were the use of cluster randomisation, organising for each clinician to provide only one type of treatment, monitoring treatment receipt, spatially or temporally separating trial arms, and informing participants about only the treatment that they were allocated to receive. There was little evidence of a difference in the magnitude of treatment effects within trials that used both cluster- and participant-level treatment allocation.

The classification of two main processes and three more minor types of contamination was based on the processes that researchers and clinicians described in such trials. The main trial conduct steps that researchers took to minimise contamination were in line with the processes that were found to be driving it. Many researchers attempted to design against contamination by carefully controlling the treatment’s delivery. These processes were often anticipated and then prevented or attenuated by the designers of the trials. There were no examples of researchers first having evaluated in detail treatment receipt within the control arm. The processes described here therefore partly represent researchers’ expectations and not entirely clinician or participant behaviour.

The small number of trials that measured and reported treatment receipt in the control arm found it to be affecting a minority of the control participants. The distribution of this was similar to the quantity found previously in other areas of medicine such as educational interventions [1], breast cancer screening [4], and cancer trials using Zelen’s design [5]. Thus while there is a lot of concern about contamination it is not clear that this problem is indeed widespread. The extent of the problem may be related to the complexity, intensity, and nature of the intervention.

Researchers often used cluster randomisation to prevent treatment contamination, amongst other reasons. While cRCTs can avoid contamination bias they are at risk of other biases. Our set of articles included 93 cluster randomised trials. Assessments of bias suggested that cRCTs were more likely to be affected by bias when considering whether randomisation took place after consent was obtained and after baseline measures were completed, whether outcome assessment was blind, and whether demographic characteristics were similar between trial arms. This was consistent with an earlier review of cRCTs that were published in three prominent medical journals which found evidence of recruitment bias [8].

The small number of trials that allocated treatment at both cluster and participant levels did not find any evidence for differences in effect size estimates. The lack of evidence for a link between the level of randomisation and treatment effect size suggested that either the employment of cluster randomisation did not prevent contamination, the anticipated contamination was overstated, or that the use of cluster randomisation led to a similar degree of bias as that caused by contamination in the participant-randomised trials. Overall, the finding was consistent with those of a review of trials of enhanced care in depression [12], and of educational interventions [1]. Similarly to previous reviews, there was considerable heterogeneity between trials identified in this study that used both cluster- and participant-level randomisation. However, the variability here is between trials and not within them because randomisation implies that the sub-trials were balanced for every variable except the level at which treatment allocation took place. It is possible that the impacts of contamination and cluster randomisation on bias are dependent on the disease or type of intervention. In order to draw substantive conclusions about the effect of treatment allocation level on contamination, a systematic review of this particular trial design is needed.

## Conclusions

This is the most comprehensive review of contamination in mental health trials to date. It is the first to identify the processes leading to contamination and the measures that researchers take in order to minimise the problem. The main limitation is that the trials were heterogeneous in that they represented a large range of illnesses and interventions. With regard to the causes of contamination, it is an assumption that the processes described by authors were the drivers of contamination. Another limitation is the time interval between the literature search and publication of this review. It is possible that the problems, solutions and reporting of contamination have evolved since the search took place.

The results of this review suggest that treatment contamination is perceived to be a significant problem in trials of complex interventions in mental health. However, the trials that measured and reported it suggest that the phenomenon is often modest (with a large range). The reporting of it is infrequent and almost certainly not as commonplace as that of treatment non-compliance. This implies a need for greater measurement and reporting of treatment receipt in the control arm of trials in this field. The findings also show that there are many steps that researchers can take to minimise contamination without resorting to the use of cluster randomisation. In addition, we found that modern causal analysis methods, including the techniques developed particularly for contamination adjustment [9, 10], are yet to be utilised to deal with contamination bias at the analysis stage. This is likely to be in part a reflection of the infrequency of measurement of treatment receipt for all participants in the control trial arm.

## Additional files

Additional file 1: Search procedure for Ovid platform. The file includes the steps used in the Ovid search procedure for trials of complex interventions in mental health where contamination was a problem are listed below. (DOCX 15 kb)

Additional file 2: Studies excluded at full text screening. The file contains a list of references for all studies that were excluded at the screening stage together with reasons for exclusion. (DOCX 87 kb)

Additional file 3: List of references with assessment of bias and support for judgement. The file contains a list of references for all studies that were included in the review together with the assessment of risk of bias and the support for judgement. (DOCX 323 kb)

## Abbreviations

CBT: cognitive behavioural therapy
cRCT: cluster randomised controlled trial
CTACS: cognitive behavioural therapy adherence scale
FCBT: family-focused cognitive behavioural therapy
IQR: interquartile range
MRC: Medical Research Council
RCT: randomised controlled trial
