# Supplementary material for: A scoping review of the problems and solutions associated with contamination in trials of complex interventions in mental health
Source: BMC Med Res Methodol. 2019 Jan 7;19:4. doi: 10.1186/s12874-018-0646-z (PMC6323722; doi:10.1186/s12874-018-0646-z)
Supplement: Supplementary file 1 — Search procedure for Ovid platform. The file includes the steps used in the Ovid search procedure for trials of complex interventions in mental health where contamination was a problem are listed below. (DOCX 15 kb) [file 12874_2018_646_MOESM1_ESM.docx]

**Additional file 1: Search procedure for Ovid**

The steps used in the Ovid search procedure for trials of complex interventions in mental

health where contamination was a problem are listed below.

1. (randomized controlled trial or randomised controlled trial).pt,dt.

2. controlled clinical trial.pt,dt.

3. (randomized or randomised).ab.

4. placebo.ab.

5. clinical trial.sh.

6. randomly.ab.

7. trial.ti.

8. 1 or 2 or 3 or 4 or 5 or 6 or 7

9. (animals not humans).sh.

10. 8 not 9

11. (complex adj3 intervention).af.

12. (complex adj3 treatment).af.

13. (complex adj3 training).af.

14. (multicomponent adj3 intervention).af.

15. (multicomponent adj3 treatment).af.

16. (multicomponent adj3 training).af.

17. (multifaceted adj3 intervention).af.

18. (multifaceted adj3 treatment).af.

19. (multifaceted adj3 training).af.

20. (social adj3 intervention).af.

21. (social adj3 treatment).af.

22. (social adj3 training).af.

23. (psychological adj3 intervention).af.

24. (psychological adj3 treatment).af.

25. (psychological adj3 training).af.

26. (psychological adj3 therapy).af.

27. (psychosocial adj3 intervention).af.

28. (psychosocial adj3 treatment).af.

29. (psychosocial adj3 training).af.

30. psychotherap*.af.

31. therapist.af.

32. (behavio?ral adj3 intervention).af.

33. (behavio?ral adj3 treatment).af.

34. (behavio?ral adj3 training).af.

35. 11 or 12 or 13 or 14 or 15 or 16 or 17 or 18 or 19 or 20 or 21 or 22 or 23 or 24 or

25 or 26 or 27 or 28 or 29 or 30 or 31 or 32 or 33 or 34

36. (treatment adj6 contaminat*).af.

37. (arm adj6 contaminat*).af.

38. (control adj6 contaminat*).af.

39. (group*1 adj6 contaminat*).af.

40. (outcome adj6 contaminat*).af.

41. (trial adj6 contaminat*).af.

42. (patient*1 adj6 contaminat*).af.

43. (intervention adj6 contaminat*).af.

44. (treatment adj6 spillover).af.

45. (arm adj6 spillover).af.

46. (control adj6 spillover).af.

47. (group*1 adj6 spillover).af.

48. (outcome adj6 spillover).af.

49. (trial adj6 spillover).af.

50. (patient*1 adj6 spillover).af.

51. (intervention adj6 spillover).af.

52. 36 or 37 or 38 or 39 or 40 or 41 or 42 or 43 or 44 or 45 or 46 or 47 or 48 or 49 or

50 or 51

53. (blood adj3 contaminat*).af.

54. (microb* adj3 contaminat*).af.

55. 53 or 54

56. 52 not 55

57. 10 and 35 and 56

58. device.ti.

59. device.sh.

60. device.ab.

61. vaccine.ti.

62. vaccine.sh.

63. vaccine.ab.

64. surgery.ti.

65. surgery.sh.

66. surgery.ab.

67. microb*.ti.

68. microb*.sh.

69. microb*.ab.

70. antimicrob*.ti.

71. antimicrob*.sh.

72. antimicrob*.ab.

73. (genes or genetic*).ti.

74. (genes or genetic*).sh.

75. (genes or genetic*).ab.

76. screening.ti.

77. decision aid.ti.

78. decision support.ti.

79. 58 or 59 or 60 or 61 or 62 or 63 or 64 or 65 or 66 or 67 or 68 or 69 or 70 or 71 or

72 or 73 or 74 or 75 or 76 or 77 or 78

80. 57 not 79

81. limit 80 to yr=“2000 -Current”

82. mental health.af.

83. psycholog*.af.

84. psychiatr*.af.

85. 82 or 83 or 84

86. 81 and 85
