# Supplementary material for: A scoping review of the problems and solutions associated with contamination in trials of complex interventions in mental health
Source: BMC Med Res Methodol. 2019 Jan 7;19:4. doi: 10.1186/s12874-018-0646-z (PMC6323722; doi:10.1186/s12874-018-0646-z)
Supplement: Supplementary file 2 — Studies excluded at full text screening. The file contains a list of references for all studies that were excluded at the screening stage together with reasons for exclusion. (DOCX 87 kb) [file 12874_2018_646_MOESM2_ESM.docx]

**Additional file 2: Studies excluded at full text screening**

| **Reference** | **Is it within the context of a specific randomised clinical trial? (Yes; no)** | **Is it a complex intervention? I.e. an intervention where multiple components work together to produce some extra benefit. (Yes; no)** | **Does it discuss the process that leads to contamination of the control arm? (Yes; no)** | **Is it in English? (Yes; no)** | **Is it mental health related (target population; intervention; primary outcome)? (Yes; no)** |
| --- | --- | --- | --- | --- | --- |
| * NCHSPTG. Selection of populations represented in the NIMH Collaborative HIV/STD Prevention Trial. AIDS. 2007;21 Suppl(2):S19-S28. | Yes | Yes | Yes | Yes | No |
| ** NCHSPTG. Methodological overview of a five-country community-level HIV/sexually transmitted disease prevention trial. AIDS. 2007;21 Suppl(2):S3-S18. | Yes | Yes | Yes | Yes | No |
| Abikoff H, Gallagher R, Wells KC, Murray DW, Huang L, Lu F, et al. Remediating Organizational Functioning in Children With ADHD: Immediate and Long-Term Effects From a Randomized Controlled Trial. Journal of Consulting & Clinical Psychology. 2013;81(1):113-28. | Yes | Yes | No | Yes | Yes |
| Abraham NS, Moayyedi P, Daniels B, Van Zanten SJOV. The methodological quality of trials affects estimates of treatment efficacy in functional (non-ulcer) dyspepsia. Alimentary Pharmacology & Therapeutics. 2004;19(6):631-41. | Yes | No | No | Yes | No |
| Albaladejo CMD, Kovacs FMMDP, Royuela AM, del Pino RBS, Zamora JP, Spanish Back Pain Research N. The Efficacy of a Short Education Program and a Short Physiotherapy Program for Treating Low Back Pain in Primary Care: A Cluster Randomized Trial. Spine. 2010;35(5):483-96. | Yes | Yes | Yes | Yes | No |
| Aliasgharpour MBSMS, Shomali M, Moghaddam MZMS, Faghihzadeh S. EFFECT OF A SELF-EFFICACY PROMOTION TRAINING PROGRAMME ON THE BODY WEIGHT CHANGES IN PATIENTS UNDERGOING HAEMODIALYSIS. Journal of Renal Care. 2012;38(3):155-61. | Yes | Yes | Yes | Yes | No |
| Als H, Duffy FH, McAnulty GB, Fischer CB, Kosta S, Butler SC, et al. Is the Newborn Individualized Developmental Care and Assessment Program (NIDCAP) effective for preterm infants with intrauterine growth restriction? Journal of Perinatology. 2011;31(2):130-6. | Yes | Yes | Yes | Yes | No |
| Altfeld SJP, Shier GEMSW, Rooney ML, Johnson TJP, Golden RLL, Karavolos KMA, et al. Effects of an Enhanced Discharge Planning Intervention for Hospitalized Older Adults: A Randomized Trial. Gerontologist. 2013;53(3):430-40. | Yes | Yes | Yes | Yes | No |
| Althaus FMDa, Paroz SMAb, Hugli OMDMPHc, Ghali WAMDMPHf, Daeppen J-BMDd, Peytremann-Bridevaux IMDMPHDe, et al. Effectiveness of Interventions Targeting Frequent Users of Emergency Departments: A Systematic Review. Annals of Emergency Medicine. 2011;58(1):41-52e42. | Yes | Yes | Yes | Yes | No |
| Ammentorp JPM, Sabroe SMD, Kofoed P-EP, Mainz JP. Effects of a communication course for clinicians on parents' perception of care - a randomized controlled trial. Scandinavian Journal of Caring Sciences. 2009;23(3):506-17. | Yes | Yes | Yes | Yes | No |
| Annesi J. Effects of treatment differences on psychosocial predictors of exercise and improved eating in obese, middle-age adults. Journal of Physical Activity & Health. 2013 Sep;10(7):1024-31. PubMed PMID: 2013-33440-010. | Yes | Yes | No | Yes | Yes |
| Arafa MEAa, Hassan Mb. Psychoeducational program for breast cancer survivors, effect on cancer-related fatigue and quality of life. Egyptian Journal of Psychiatry. 2013;34(1):25-33. | No | Yes | No | Yes | Yes |
| Atlantis E, Barnes EH, Fiatarone Singh MA. Efficacy of exercise for treating overweight in children and adolescents: a systematic review. International Journal of Obesity. 2006;30(7):1027-40. | Yes | Yes | Yes | Yes | No |
| Auvinen A, Hakama M, Ala-Opas M, Vornanen T, Leppilahti M, Salminen P, et al. A randomized trial of choice of treatment in prostate cancer: the effect of intervention on the treatment chosen. BJU International. 2004;93(1):52-6. | Yes | Yes | No | Yes | No |
| Baggaley RFa, White RGb, Hollingsworth TDa, Boily M-Ca. Heterosexual HIV-1 Infectiousness and Antiretroviral Use: Systematic Review of Prospective Studies of Discordant Couples. Epidemiology. 2013;24(1):110-21. | Yes | No | No | Yes | No |
| Baker-Ericzen MJP, Connelly CDPRN, Hazen ALP, Duenas CP, Landsverk JAP, Horwitz SMP. A Collaborative Care Telemedicine Intervention to Overcome Treatment Barriers for Latina Women With Depression During the Perinatal Period. Families, Systems, & Health. 2012;30(3):224-40. | No | Yes | Yes | Yes | Yes |
| Bakitas MDA, Lyons KDSOTR, Hegel MTP, Balan SMD, Brokaw FCMDMS, Seville JP, et al. Effects of a Palliative Care Intervention on Clinical Outcomes in Patients With Advanced Cancer: The Project ENABLE II Randomized Controlled Trial. JAMA. 2009;302(7):741-9. | Yes | Yes | No | Yes | Yes |
| Bakker FC, Robben SHM, Olde Rikkert MGM. Effects of hospital-wide interventions to improve care for frail older inpatients: a systematic review. BMJ Quality & Safety. 2011;20(8):680-91. | Yes | Yes | Yes | Yes | No |
| Bar-Eli M, Dreshman R, Blumenstein B, Weinstein Y. The Effect of Mental Training with Biofeedback on the Performance of Young Swimmers. Applied Psychology. 2002;51(4):567-81. | No | Yes | Yes | Yes | No |
| Barlow DHP, Gorman JMMD, Shear MKMD, Woods SWMD. Cognitive-Behavioral Therapy, Imipramine, or Their Combination for Panic Disorder: A Randomized Controlled Trial. JAMA. 2000;283(19):2529-36. | Yes | Yes | No | Yes | Yes |
| Barras SBMOT, Currently undertaking a Ph DaUoSA, Deputy Chief Occupational T. A systematic and critical review of the literature: The effectiveness of Occupational Therapy Home Assessment on a range of outcome measures. Australian Occupational Therapy Journal. 2005;52(4):326-36. | Yes | Yes | Yes | Yes | No |
| Bates-Jensen BMPRNC, Alessi CAMD, Al-Samarrai NRMS, Schnelle JFP. The Effects of an Exercise and Incontinence Intervention on Skin Health Outcomes in Nursing Home Residents. Journal of the American Geriatrics Society. 2003;51(3):348-55. | Yes | Yes | Yes | Yes | No |
| Baxter P, Akhtar-Danesh N, Landeen J, Norman GF. Teaching Critical Management Skills to Senior Nursing Students: Videotaped or Interactive Hands-On Instruction? Nursing Education Perspectives March/April. 2012;33(2):106-10. | Yes | Yes | Yes | Yes | No |
| Becker CB, Smith LM, Ciao AC. Peer-Facilitated Eating Disorder Prevention: A Randomized Effectiveness Trial of Cognitive Dissonance and Media Advocacy. Journal of Counseling Psychology. 2006;53(4):550-5. | Yes | Yes | No | Yes | Yes |
| Belleville G, Guay C, Guay B, Morin CM. Hypnotic Taper With or Without Self-Help Treatment of Insomnia: A Randomized Clinical Trial. Journal of Consulting & Clinical Psychology. 2007;75(2):325-35. | Yes | Yes | No | Yes | Yes |
| Benedetti Fab, Arduino Cab, Costa Sc, Vighetti Sab, Tarenzi La, Rainero Iad, et al. Loss of expectation-related mechanisms in Alzheimer's disease makes analgesic therapies less effective. Pain. 2006;121(1-2):133-44. | Yes | Yes | No | Yes | Yes |
| Bennell KLP, Egerton TP, Martin JB, Abbott JHP, Metcalf BB, McManus FB, et al. Effect of Physical Therapy on Pain and Function in Patients With Hip Osteoarthritis: A Randomized Clinical Trial. JAMA. 2014;311(19):1987-97. | Yes | Yes | Yes | Yes | No |
| Bergman GJDM, Winters JCPMD, Groenier KHM, Pool JJM, Jong BM-dPMD, Postema KPMD, et al. Manipulative Therapy in Addition to Usual Medical Care for Patients with Shoulder Dysfunction and Pain: A Randomized, Controlled Trial. Annals of Internal Medicine. 2004;141(6):432-9. | Yes | Yes | No | Yes | No |
| Bernasconi F, Schmidt A, Pokorny T, Kometer M, Seifritz E, Vollenweider FX. Spatiotemporal Brain Dynamics of Emotional Face Processing Modulations Induced by the Serotonin 1A/2A Receptor Agonist Psilocybin. Cerebral Cortex. 2014;24(12):3221-31. | Yes | No | No | Yes | Yes |
| Beutler LE, Forrester B. What Needs to Change: Moving From "Research Informed" Practice to "Empirically Effective" Practice. Journal of Psychotherapy Integration. 2014;24(3):168-77. | Yes | Yes | No | Yes | Yes |
| Bocchi EAMD, Cruz FRN, Guimaraes GP, Moreira LFPMD, Issa VSMD, Ferreira SMAMD, et al. Long-Term Prospective, Randomized, Controlled Study Using Repetitive Education at Six-Month Intervals and Monitoring for Adherence in Heart Failure Outpatients: The REMADHE Trial. Circulation: Heart Failure. 2008;1(2):115-24. | Yes | Yes | Yes | Yes | No |
| Bodenmann G, Plancherel B, Beach SRH, Widmer K, Gabriel B, Meuwly N, et al. Effects of Coping-Oriented Couples Therapy on Depression: A Randomized Clinical Trial. Journal of Consulting & Clinical Psychology. 2008;76(6):944-54. | Yes | Yes | No | Yes | Yes |
| Bohus MMa, Dyer ASASa, Priebe KKa, Kruger AAa, Kleindienst NNa, Schmahl CCa, et al. Dialectical Behaviour Therapy for Post-traumatic Stress Disorder after Childhood Sexual Abuse in Patients with and without Borderline Personality Disorder: A Randomised Controlled Trial. Psychotherapy & Psychosomatics. 2013;82(4):221-33. | Yes | Yes | No | Yes | Yes |
| Bonell CP, Hargreaves J, Cousens S, Ross D, Hayes R, Petticrew M, et al. Alternatives to randomisation in the evaluation of public health interventions: design challenges and solutions. Journal of Epidemiology & Community Health. 2011;65(7):582-7. | No | Yes | Yes | Yes | Yes |
| Bonner GJ, Wang E, Wilkie DJ, Ferrans CE, Dancy B, Watkins Y. Advance care treatment plan (ACT-Plan) for African American family caregivers: A pilot study. Dementia. 2014;13(1):79-95. | No | Yes | Yes | Yes | Yes |
| Booth AO, Nowson CA, Matters H. Evaluation of an interactive, Internet-based weight loss program: a pilot study. Health Education Research. 2008;23(3):371-81. | Yes | Yes | Yes | Yes | No |
| Borgaonkar MR, Townson G, Donnelly M, Irvine EJ. Providing Disease-Related Information Worsens Health-Related Quality of Life in Inflammatory Bowel Disease. Inflammatory Bowel Diseases. 2002;8(4):264-9. | Yes | Yes | Yes | Yes | No |
| Bowen A, Agboatwalla M, Ayers T, Tobery T, Tariq M, Luby SP. Sustained improvements in handwashing indicators more than 5 years after a cluster-randomised, community-based trial of handwashing promotion in Karachi, Pakistan. Tropical Medicine & International Health. 2013;18(3):259-67. | Yes | Yes | No | Yes | No |
| Bridge JA, Axelson DA. The contribution of pharmacoepidemiology to the antidepressant-suicidality debate in children and adolescents. International Review of Psychiatry. 2008 Apr;20(2):209-14. PubMed PMID: 2008-04938-015. | No | No | No | Yes | Yes |
| Brinn MP, Carson KV, Esterman AJ, Chang AB, Smith BJ. Cochrane Review: Mass media interventions for preventing smoking in young people. Evidence-Based Child Health: A Cochrane Review Journal. 2012;7(1):86-144. | Yes | No | Yes | Yes | No |
| Brock K, Haase G, Rothacher G, Cotton S. Does physiotherapy based on the Bobath concept, in conjunction with a task practice, achieve greater improvement in walking ability in people with stroke compared to physiotherapy focused on structured task practice alone? A pilot randomized controlled trial. Clinical Rehabilitation. 2011;25(10):903-12. | Yes | Yes | Yes | Yes | No |
| Brown SARNPF, Blozis SAP, Kouzekanani KP, Garcia AARNP, Winchell MMS, Hanis CLP. Health Beliefs of Mexican Americans With Type 2 Diabetes: The Starr County Border Health Initiative. Diabetes Educator March/April. 2007;33(2):300-8. | Yes | Yes | Yes | Yes | No |
| Brown SARNPHDF, Garcia AARNMSN, Kouzekanani KPHD, Hanis CLPHD. Culturally Competent Diabetes Self-Management Education for Mexican Americans: The Starr County Border Health Initiative. Diabetes Care. 2002;25(2):259-68. | Yes | Yes | No | Yes | No |
| Bruckert E, Giral P, Paillard F, Ferrieres J, Schlienger J-L, Renucci J-F, et al. Effect of an Educational Program (PEGASE) on Cardiovascular Risk in Hypercholesterolaemic Patients. Cardiovascular Drugs & Therapy. 2008;22(6):495-505. | Yes | Yes | Yes | Yes | No |
| Burns J, Dudley M, Hazell P, Patton G. Clinical management of deliberate self-harm in young people: the need for evidence-based approaches to reduce repetition. Australian & New Zealand Journal of Psychiatry. 2005;39(3):121-8. | Yes | Yes | No | Yes | Yes |
| Burton CDPBNRGN, Gibbon BPMDDRMNRGN. Expanding the role of the stroke nurse: a pragmatic clinical trial. Journal of Advanced Nursing. 2005;52(6):640-50. | Yes | Yes | Yes | Yes | No |
| Carnes MMDMS, Devine PGP, Baier Manwell LMS, Byars-Winston AP, Fine EP, Ford CEP, et al. The Effect of an Intervention to Break the Gender Bias Habit for Faculty at One Institution: A Cluster Randomized, Controlled Trial. Academic Medicine. 2015;90(2):221-30. | Yes | Yes | Yes | Yes | No |
| Carney PAP, Nierenberg DWMD, Pipas CFMD, Brooks WBMD, Stukel TAP, Keller AMMPH. Educational Epidemiology: Applying Population-Based Design and Analytic Approaches to Study Medical Education. JAMA. 2004;292(9):1044-50. | Yes | Yes | Yes | Yes | No |
| Carpenter JS, Burns DS, Wu J, Yu M, Ryker K, Tallman E, et al. Strategies Used and Data Obtained During Treatment Fidelity Monitoring. Nursing Research January/February. 2013;62(1):59-65. | Yes | Yes | Yes | Yes | No |
| Carr LJ, Dunsiger SI, Lewis B, Ciccolo JT, Hartman S, Bock B, et al. Randomized Controlled Trial Testing an Internet Physical Activity Intervention for Sedentary Adults. Health Psychology. 2013;32(3):328-36. | Yes | Yes | Yes | Yes | No |
| Carvalho TS, Sampaio FC, Diniz A, Bonecker M, Van Amerongen WE. Two years survival rate of Class II ART restorations in primary molars using two ways to avoid saliva contamination. International Journal of Paediatric Dentistry. 2010;20(6):419-25. | Yes | No | No | Yes | No |
| Celentano DDSMHS, Bond KCS, Lyles CMP, Eiumtrakul SMD, Go VFLMPH, Beyrer CMDMPH, et al. Preventive Intervention to Reduce Sexually Transmitted Infections: A Field Trial in the Royal Thai Army. Archives of Internal Medicine. 2000;160(4):535-40. | Yes | Yes | Yes | Yes | No |
| Cepeda MSMD, Lau JMD, Carr DBMD. Defining the Therapeutic Role of Local Anesthetic Sympathetic Blockade in Complex Regional Pain Syndrome: A Narrative and Systematic Review. Clinical Journal of Pain July/August. 2002;18(4):216-33. | Yes | No | No | Yes | No |
| Chan DYL, Chan CCH, Au DKS. Motor relearning programme for stroke patients: a randomized controlled trial. Clinical Rehabilitation. 2006;20(3):191-200. | Yes | Yes | No | Yes | No |
| Chan JCM, So W-YF, Yeung C-YM, Ko GTM, Lau I-TF, Tsang M-WF, et al. Effects of Structured Versus Usual Care on Renal Endpoint in Type 2 Diabetes: The SURE Study: A randomized multicenter translational study. Diabetes Care. 2009;32(6):977-82. | Yes | Yes | Yes | Yes | No |
| Chan JCNMD, Sui YP, Oldenburg BP, Zhang YMB, Chung HHYM, Goggins WP, et al. Effects of Telephone-Based Peer Support in Patients With Type 2 Diabetes Mellitus Receiving Integrated Care: A Randomized Clinical Trial. JAMA Internal Medicine. 2014;174(6):972-81. | Yes | Yes | Yes | Yes | No |
| Chan SSC, Leung DYP, Wong DCN, Lau C-P, Wong VT, Lam T-H. A randomized controlled trial of stage-matched intervention for smoking cessation in cardiac out-patients. Addiction. 2012;107(4):829-37. | Yes | Yes | No | Yes | Yes |
| Cheater FMRGNP, Baker RMDM, Reddish SM, Spiers NP, Wailoo AP, Gillies CM, et al. Cluster Randomized Controlled Trial of the Effectiveness of Audit and Feedback and Educational Outreach on Improving Nursing Practice and Patient Outcomes. Medical Care. 2006;44(6):542-51. | Yes | Yes | Yes | Yes | No |
| Cherkin DCP, Eisenberg DMD, Sherman KJP, Barlow WP, Kaptchuk TJOMD, Street JRNMNPNP, et al. Randomized Trial Comparing Traditional Chinese Medical Acupuncture, Therapeutic Massage, and Self-care Education for Chronic Low Back Pain. Archives of Internal Medicine. 2001;161(8):1081-8. | Yes | Yes | Yes | Yes | No |
| Chow E, Tsao MN, Harth T. Does psychosocial intervention improve survival in cancer? A meta-analysis. Palliative Medicine. 2004;18(1):25-31. | No | Yes | Yes | Yes | Yes |
| Christodoulides T, Dudley R, Brown S, Turkington D, Beck AT. Cognitive behaviour therapy in patients with schizophrenia who are not prescribed antipsychotic medication: A case series. Psychology & Psychotherapy: Theory, Research & Practice. 2008;81(2):199-207. | Yes | Yes | No | Yes | Yes |
| Christofoletti G, Oliani MM, Gobbi S, Stella F, Teresa L, Gobbi B, et al. A controlled clinical trial on the effects of motor intervention on balance and cognition in institutionalized elderly patients with dementia. Clinical Rehabilitation. 2008;22(7):618-26. | Yes | Yes | No | Yes | Yes |
| Chu BC, Talbott Crocco S, Arnold CC, Brown R, Southam-Gerow MA, Weisz JR. Sustained Implementation of Cognitive-Behavioral Therapy for Youth Anxiety and Depression: Long-Term Effects of Structured Training and Consultation on Therapist Practice in the Field. Professional Psychology - Research & Practice. 2015;46(1):70-9. | No | Yes | Yes | Yes | Yes |
| Chung MMPH, Raman GMD, Trikalinos TMDP, Lau JMD, Ip SMD. Interventions in Primary Care to Promote Breastfeeding: An Evidence Review for the U.S. Preventive Services Task Force. Annals of Internal Medicine. 2008;149(8):565-82. | No | Yes | No | Yes | No |
| Clemson L, Singh MF, Bundy A, Cumming RG, Weissel E, Munro J, et al. LiFE Pilot Study: A randomised trial of balance and strength training embedded in daily life activity to reduce falls in older adults. Australian Occupational Therapy Journal. 2010;57(1):42-50. | Yes | Yes | Yes | Yes | No |
| Clever SLMDMS, Ford DEMDMPH, Rubenstein LVMDM, Rost KMP, Meredith LSP, Sherbourne CDP, et al. Primary Care Patients' Involvement in Decision-Making Is Associated With Improvement in Depression. Medical Care. 2006;44(5):398-405. | No | Yes | No | Yes | Yes |
| Collard DCM, Chinapaw MJM, van Mechelen W, Verhagen EALM. Design of the iPlay Study: Systematic Development of a Physical Activity Injury Prevention Programme for Primary School Children. Sports Medicine. 2009;39(11):889-901. | No | Yes | Yes | Yes | No |
| Collins CEPBDNDDCEAPD, Warren JMPBRD, Neve MBNDAPD, McCoy PBAPD, Stokes BBMM. Systematic review of interventions in the management of overweight and obese children which include a dietary component. International Journal of Evidence-Based Healthcare. 2007;5(1):2-53. | Yes | Yes | No | Yes | No |
| Collins TCMD, Mph, Lunos SMS, Carlson TBA, Henderson KBA, Lightbourne MMD, et al. Effects of a Home-Based Walking Intervention on Mobility and Quality of Life in People With Diabetes and Peripheral Arterial Disease: A randomized controlled trial. Diabetes Care. 2011;34(10):2174-9. | Yes | Yes | Yes | Yes | No |
| Connelly JBMDMF. Evaluating complex public health interventions: theory, methods and scope of realist enquiry. Journal of Evaluation in Clinical Practice. 2007;13(6):935-41. | No | Yes | No | Yes | No |
| Conrod PJP, O'Leary-Barrett MBA, Newton NP, Topper LM, Castellanos-Ryan NP, Mackie CP, et al. Effectiveness of a Selective, Personality-Targeted Prevention Program for Adolescent Alcohol Use and Misuse: A Cluster Randomized Controlled Trial. JAMA Psychiatry. 2013;70(3):334-42. | Yes | Yes | No | Yes | Yes |
| Cook JAm, McCulloch Pcris, Blazeby JMpos, Beard DJpoms, Marinac-Dabic Dd, Sedrakyan Aapoph, et al. IDEAL framework for surgical innovation 3: randomised controlled trials in the assessment stage and evaluations in the long term study stage. BMJ June. 2013;22(346). | No | No | Yes | Yes | No |
| Cooke PAMMM, Tully MAP, Cupples MEMDF, Gilliland AEMDF, Gormley GJMDF. A randomised control trial of experiential learning to promote physical activity. Education for Primary Care. 2013;24(6):427-35. | Yes | Yes | Yes | Yes | No |
| Cordioli AV. Cognitive-behavioral therapy in obsessive-compulsive disorder. Revista Brasileira de Psiquiatria. 2008 Oct;30(Suppl2):S65-S72. PubMed PMID: 2009-01863-002. | No | Yes | No | Yes | Yes |
| Coren E, Hossain R, Pardo JP, Veras MMS, Chakraborty K, Harris H, et al. Interventions for promoting reintegration and reducing harmful behaviour and lifestyles in street-connected children and young people. Evidence-Based Child Health: A Cochrane Review Journal. 2013;8(4):1140-272. | No | Yes | Yes | Yes | Yes |
| Counsell SRMD, Callahan CMMD, Clark DOP, Tu WP, Buttar ABMDMS, Stump TEMS, et al. Geriatric Care Management for Low-Income Seniors: A Randomized Controlled Trial. JAMA. 2007;298(22):2623-33. | Yes | Yes | Yes | Yes | No |
| Courneya K, Friedenreich C, Quinney H, Fields A, Jones L, Fairey A. A randomized trial of exercise and quality of life in colorectal cancer survivors. European Journal of Cancer Care. 2003 Dec;12(4):347-57. PubMed PMID: 2003-10123-006. | Yes | Yes | Yes | Yes | No |
| Courneya K, Friedenreich C, Quinney H, Fields A, Jones L, Fairey A. A randomized trial of exercise and quality of life in colorectal cancer survivors. European Journal of Cancer Care. 2003 Dec;12(4):347-57. PubMed PMID: 2003-10123-006. | Yes | Yes | Yes | Yes | No |
| Courneya KS, Friedenreich CM, Quinney H, Fields AL, Jones LW, Fairey AS. Predictors of Adherence and Contamination in a Randomized Trial of Exercise in Colorectal Cancer Survivors. Psycho-Oncology. 2004 Dec;13(12):857-66. PubMed PMID: 2004-22264-005. | Yes | Yes | Yes | Yes | No |
| Courneya KS, Friedenreich CM, Sela RA, Quinney H, Rhodes RE, Jones LW. Exercise motivation and adherence in cancer survivors after participation in a randomized controlled trial: An attribution theory perspective. International Journal of Behavioral Medicine. 2004 Mar;11(1):8-17. PubMed PMID: 2004-12879-002. | Yes | Yes | No | Yes | Yes |
| Courneya KS, Segal RJ, Gelmon K, Reid RD, Mackey JR, Friedenreich CM, et al. Predictors of Supervised Exercise Adherence during Breast Cancer Chemotherapy. Medicine & Science in Sports & Exercise. 2008;40(6):1180-7. | Yes | Yes | No | Yes | No |
| Courneya KS, Stevinson C, McNeely ML, Sellar CM, Friedenreich CM, Peddle-McIntyre CJ, et al. Predictors of follow-up exercise behavior 6 months after a randomized trial of supervised exercise training in lymphoma patients. Pscyho-Oncology. 2012;21(10):1124-31. | Yes | Yes | No | Yes | No |
| Cristea IA, Kok RN, Cuijpers P. Efficacy of cognitive bias modification interventions in anxiety and depression: meta-analysis. British Journal of Psychiatry. 2015;206(1):7-16. | No | Yes | No | Yes | Yes |
| Crocker T, Young J, Forster A, Brown L, Ozer S, Greenwood DC. The effect of physical rehabilitation on activities of daily living in older residents of long-term care facilities: systematic review with meta-analysis. Age & Ageing. 2013;42(6):682-8. | No | Yes | No | Yes | No |
| Cuthbertson BHcoccm, professor of a, Rattray Jsl, Campbell MKd, professor, Gager Micf-un, et al. The PRaCTICaL study of nurse led, intensive care follow-up programmes for improving long term outcomes from critical illness: a pragmatic randomised controlled trial. BMJ October. 2009;17(339). | Yes | Yes | Yes | Yes | No |
| Dainiak NMDF, Gent RNMBCF, Carr ZMDP, Schneider RMD, Bader JMD, Buglova EMDPDS, et al. Literature Review and Global Consensus on Management of Acute Radiation Syndrome Affecting Nonhematopoietic Organ Systems. Disaster Medicine & Public Health Preparedness. 2011;5(3):183-201. | No | No | No | Yes | No |
| Daley AJ, Crank H, Mutrie N, Saxton JM, Coleman R. Determinants of adherence to exercise in women treated for breast cancer. European Journal of Oncology Nursing. 2007 Dec;11(5):392-9. PubMed PMID: 2008-09107-002. | Yes | Yes | No | Yes | No |
| Dalleur O, Boland B, Losseau C, Henrard S, Wouters D, Speybroeck N, et al. Reduction of Potentially Inappropriate Medications Using the STOPP Criteria in Frail Older Inpatients: A Randomised Controlled Study. Drugs & Aging. 2014;31(4):291-8. | Yes | Yes | Yes | Yes | No |
| Davis MPab, Mitchell GKc. Topics in research: structuring studies in palliative care. Current Opinion in Supportive & Palliative Care. 2012;6(4):483-9. | No | Yes | No | Yes | No |
| Day TBMRGNCERNT, Wainwright SPBMRGNP, Wilson-Barnett JBMPSRNFF. An evaluation of a teaching intervention to improve the practice of endotracheal suctioning in intensive care units. Journal of Clinical Nursing. 2001;10(5):682-96. | Yes | Yes | Yes | Yes | No |
| de Heer HD, Koehly L, Pederson R, Morera O. Effectiveness and spillover of an after-school health promotion program for Hispanic elementary school children. American Journal of Public Health. 2011 Oct;101(10):1907-13. PubMed PMID: 2011-26105-013. | Yes | Yes | No | Yes | No |
| de Jong PJ, de Graaf-Peters V, van Hout WJ, van Wees R. Covariation bias for social events and signs of (dis)approval in high and low socially anxious individuals. Journal of Behavior Therapy and Experimental Psychiatry. 2009 Jun;40(2):359-73. PubMed PMID: 2009-06418-020. | No | No | No | Yes | Yes |
| de Jonge PP, Latour CHMRNa, Huyse FJMDP. Implementing Psychiatric Interventions on a Medical Ward: Effects on Patients' Quality of Life and Length of Hospital Stay. Psychosomatic Medicine November/December. 2003;65(6):997-1002. | No | Yes | Yes | Yes | Yes |
| De Visschere L, Schols J, van der Putten G-J, de Baat C, Vanobbergen J. Effect evaluation of a supervised versus non-supervised implementation of an oral health care guideline in nursing homes: a cluster randomised controlled clinical trial. Gerodontology. 2012;29(2):e96-e106. | Yes | Yes | Yes | Yes | No |
| Deakin TA, Cade JE, Williams R, Greenwood DC. Structured patient education: the Diabetes X-PERT Programme makes a difference. Diabetic Med. 2006;23(9):944-54. | Yes | Yes | Yes | Yes | No |
| Dechamps AP, Diolez PP, Thiaudiere EP, Tulon AM, Onifade CMD, Vuong TMD, et al. Effects of Exercise Programs to Prevent Decline in Health-Related Quality of Life in Highly Deconditioned Institutionalized Elderly Persons: A Randomized Controlled Trial. Archives of Internal Medicine. 2010;170(2):162-9. | Yes | Yes | No | Yes | Yes |
| Delaney CP, Zutshi M, Senagore AJ, Remzi FH, Hammel J, Fazio VW. Prospective, randomized, controlled trial between a pathway of controlled rehabilitation with early ambulation and diet and traditional postoperative care after laparotomy and intestinal resection. Diseases of the Colon & Rectum. 2003 Jul 1;46(7):851-9. | Yes | Yes | Yes | Yes | No |
| Delvaux N, Razavi D, Marchal S, Bredart A, Farvacques C, Slachmuylder JL. Effects of a 105 hours psychological training program on attitudes, communication skills and occupational stress in oncology: a randomised study. British journal of cancer. 2004 Jan 6;90(1):106. | Yes | Yes | No | Yes | Yes |
| Denys Da, Burger Hb, van Megen Ha, de Geus Fa, Westenberg Ha. A score for predicting response to pharmacotherapy in obsessive-compulsive disorder. International Clinical Psychopharmacology. 2003;18(6):315-22. | Yes | No | No | Yes | Yes |
| Desrosiers J, Bourbonnais D, Corriveau H, Gosselin S, Bravo G. Effectiveness of unilateral and symmetrical bilateral task training for arm during the subacute phase after stroke: a randomized controlled trial. Clinical Rehabilitation. 2005;19(6):581-93. | Yes | Yes | Yes | Yes | No |
| Dey P, Woodman M, Gibbs A, Steele R, Stocks SJ, Wagstaff S, et al. Early assessment by a mobile stroke team: a randomised controlled trial. Age & Ageing. 2005;34(4):331-8. | Yes | Yes | Yes | Yes | No |
| Di Carlo A, Lamassa M, Wellwood I, Bovis F, Baldereschi M, Nencini P, et al. Stroke unit care in clinical practice: an observational study in the Florence center of the European Registers of Stroke (EROS) Project. European Journal of Neurology. 2011;18(5):686-94. | No | Yes | Yes | Yes | No |
| Dizon JMRM, Grimmer-Somers KAP, Kumar SP. Current evidence on evidence-based practice training in allied health: a systematic review of the literature. International Journal of Evidence-Based Healthcare. 2012;10(4):347-60. | Yes | Yes | No | Yes | No |
| Dracup KRNP, Moser DKRND, Pelter MMRNP, Nesbitt TSMDMPH, Southard JMD, Paul SMP, et al. Randomized, Controlled Trial to Improve Self-Care in Patients With Heart Failure Living in Rural Areas. Circulation. 2014;130(3):256-64. | Yes | Yes | Yes | Yes | No |
| Driessen MT, Proper KI, Anema JR, Knol DL, Bongers PM, van der Beek AJ. Participatory ergonomics to reduce exposure to psychosocial and physical risk factors for low back pain and neck pain: results of a cluster randomised controlled trial. Occupational & Environmental Medicine. 2011;68(9):674-81. | Yes | Yes | Yes | Yes | No |
| Dunn G. Estimating the causal effects of treatment. Epidemiologia e Psichiatria Sociale. 2002 Jul-Sep;11(3):206-15. PubMed PMID: 2002-08114-007. | No | No | No | Yes | No |
| Eaton LH, Doorenbos AZ, Schmitz KL, Carpenter KM, McGregor BA. Establishing Treatment Fidelity in a Web-Based Behavioral Intervention Study. Nursing Research November/December. 2011;60(6):430-5. | Yes | Yes | No | Yes | Yes |
| Ekers DMDCBTRMN, Richards DPRN, McMillan DDP, Bland JMMP, Gilbody SDF. Behavioural activation delivered by the non-specialist: phase II randomised controlled trial. British Journal of Psychiatry. 2011;198(1):66-72. | Yes | Yes | No | Yes | Yes |
| Eldredge JD. The Randomised Controlled Trial design: unrecognized opportunities for health sciences librarianship. Health Information & Libraries Journal Supplement. 2003;20 Supplement(1):34-44. | No | Yes | No | Yes | Yes |
| Elliott CM, Radomsky AS. Mental contamination: The effects of imagined physical dirt and immoral behaviour. Behaviour Research and Therapy. 2012 Jun;50(6):422-7. PubMed PMID: 2012-12215-010. | No | No | No | Yes | Yes |
| Enns EMD, Rhemtulla RNP, Ewa VMD, Fruetel KMDM, Holroyd-Leduc JMMD. A Controlled Quality Improvement Trial to Reduce the Use of Physical Restraints in Older Hospitalized Adults. Journal of the American Geriatrics Society. 2014;62(3):541-5. | Yes | Yes | Yes | Yes | No |
| Ersser SJ, Cowdell FC, Nicholls PG, Latter SM, Healy E. A pilot randomized controlled trial to examine the feasibility and efficacy of an educational nursing intervention to improve self-management practices in patients with mild-moderate psoriasis. Journal of the European Academy of Dermatology & Venereology. 2012;26(6):738-45. | Yes | Yes | Yes | Yes | No |
| Evans DWPBO, Breen ACPDC, Pincus TPM, Sim JPM, Underwood MMD, Vogel SDO, et al. The Effectiveness of a Posted Information Package on the Beliefs and Behavior of Musculoskeletal Practitioners: The UK Chiropractors, Osteopaths, and Musculoskeletal Physiotherapists Low Back Pain ManagemENT (COMPLeMENT) Randomized Trial. Spine. 2010;35(8):858-66. | Yes | Yes | Yes | Yes | No |
| Evans MM. Evidence-Based Practice Protocol to Improve Glucose Control in Individuals with Type 2 Diabetes Mellitus. MEDSURG Nursing November/December. 2010;19(6):317-22. | No | Yes | Yes | Yes | No |
| Farmer TW, Hall CM, Petrin R, Hamm JV, Dadisman K. Evaluating the Impact of a Multicomponent Intervention Model on Teachers' Awareness of Social Networks at the Beginning of Middle School in Rural Communities. School Psychology Quarterly. 2010;25(2):94-106. | Yes | Yes | Yes | Yes | No |
| Feeley NRNP, Zelkowitz PE, Charbonneau LRNB, Cormier CRNMPNC, Lacroix ARNMN, Marie CSP, et al. Assessing the Feasibility and Acceptability of an Intervention to Reduce Anxiety and Enhance Sensitivity Among Mothers of Very Low Birth-Weight Infants. Advances in Neonatal Care. 2008;8(5):276-84. | No | Yes | Yes | Yes | Yes |
| Ferenci P. Review article: diagnosis and current therapy of Wilson's disease. Alimentary Pharmacology & Therapeutics. 2004;19(2):157-65. | No | No | No | Yes | No |
| Ferenczi Sa, Nunez Cb, Pinter-Kubler Ba, Foldes Aa, Martin Fb, Ladnyanszky Markus Va, et al. Changes in metabolic-related variables during chronic morphine treatment. Neurochemistry International. 2010;57(3):323-30. | No | No | No | Yes | No |
| Fernandez-Hermida JR, Calafat A, Becona E, Tsertsvadze A, Foxcroft DR. Assessment of generalizability, applicability and predictability (GAP) for evaluating external validity in studies of universal family-based prevention of alcohol misuse in young people: systematic methodological review of randomized controlled trials. Addiction. 2012;107(9):1570-9. | No | Yes | No | Yes | Yes |
| Figueiredo PA, Powers SK, Ferreira RM, Amado F, Appell HJ, Duarte JA. Impact of Lifelong Sedentary Behavior on Mitochondrial Function of Mice Skeletal Muscle. Journals of Gerontology Series A, Biological Sciences & Medical Sciences. 2009;64A(9):927-39. | No | No | No | Yes | No |
| Fillion L, Gagnon P, Leblond F, Gelinas C, Savard J, Dupuis R, et al. A brief intervention for fatigue management in breast cancer survivors. Cancer Nursing. 2008 Mar-Apr;31(2):145-59. PubMed PMID: 2008-03179-007. | Yes | Yes | No | Yes | Yes |
| Fischer K, Goetghebeur E, Vrijens B, White IR. A structural mean model to allow for noncompliance in a randomized trial comparing 2 active treatments. Biostatistics. 2011;12(2):247-57. | No | No | No | Yes | No |
| Floyd AH, Moyer A. Effects of participant preferences in unblinded randomized controlled trials. Journal of Empirical Research on Human Research Ethics. 2010 Jun;5(2):81-93. PubMed PMID: 2010-13386-009. | No | No | Yes | Yes | No |
| Fordis MMD, King JEP, Ballantyne CMMD, Jones PHMD, Schneider KHMBA, Spann SJMD, et al. Comparison of the Instructional Efficacy of Internet-Based CME With Live Interactive CME Workshops: A Randomized Controlled Trial. JAMA. 2005;294(9):1043-51. | Yes | Yes | Yes | Yes | No |
| Foster JMPa, Usherwood TBMDBSb, Smith LPc, Sawyer SMMMDdef, Xuan WMMPg, Rand CSPh, et al. Inhaler reminders improve adherence with controller treatment in primary care patients with asthma. Journal of Allergy & Clinical Immunology. 2014;134(6):1260-8e3. | Yes | Yes | Yes | Yes | No |
| Foxcroft DR, Ireland D, Lister-Sharp DJ, Lowe G, Breen R. Longer-term primary prevention for alcohol misuse in young people: a systematic review. Addiction. 2003;98(4):397-411. | Yes | Yes | No | Yes | Yes |
| Foxcroft DR, Tsertsvadze A. Cochrane Review: Universal school-based prevention programs for alcohol misuse in young people. Evidence-Based Child Health: A Cochrane Review Journal. 2012;7(2):450-575. | Yes | Yes | No | Yes | Yes |
| Frasure-Smith NP, Koszycki DP, Swenson JRMD, Baker BM, van Zyl LTMD, Laliberte M-AMD, et al. Design and Rationale for a Randomized, Controlled Trial of Interpersonal Psychotherapy and Citalopram for Depression in Coronary Artery Disease (CREATE). Psychosomatic Medicine January/February. 2006;68(1):87-93. | Yes | Yes | No | Yes | Yes |
| Frayne CA, Geringer JM. Self-Management Training for Improving Job Performance: A Field Experiment Involving Salespeople. Journal of Applied Psychology. 2000;85(3):361-72. | No | Yes | Yes | Yes | No |
| Galantino MLPTPM, Galbavy RPTMPT, Quinn LDPT. Therapeutic Effects of Yoga for Children: A Systematic Review of the Literature. Pediatric Physical Therapy Spring. 2008;20(1):66-80. | Yes | Yes | No | Yes | Yes |
| Gao R, Lv Y, Li X, Zhou K, Jin X, Dang S, et al. Effects of comprehensive sleep management on sleep quality in university students in mainland China. Sleep & Biological Rhythms. 2014;12(3):194-202. | Yes | Yes | Unclear | Yes | Yes |
| Garcia DOP, Thomson CAPRD. Physical Activity and Cancer Survivorship. Nutrition in Clinical Practice. 2014;29(6):768-79. | Yes | Yes | No | Yes | Yes |
| Genberg BLMPH, Kulich MP, Kawichai SMP, Modiba PMA, Chingono AM, Kilonzo GPMBCMFMD, et al. HIV Risk Behaviors in Sub-Saharan Africa and Northern Thailand: Baseline Behavioral Data From Project Accept. JAIDS Journal of Acquired Immune Deficiency Syndromes. 2008;49(3):309-19. | Yes | Yes | Yes | Yes | No |
| Gilbody SDM, Whitty PMDM, Grimshaw JPF, Thomas RP. Educational and Organizational Interventions to Improve the Management of Depression in Primary Care: A Systematic Review. JAMA. 2003;289(23):3145-51. | No | Yes | No | Yes | Yes |
| Gillham S, Endacott R. Impact of enhanced secondary prevention on health behaviour in patients following minor stroke and transient ischaemic attack: a randomized controlled trial. Clinical Rehabilitation. 2010;24(9):822-30. | Yes | Yes | No | Yes | Yes |
| Gleeson JFM, Chanen A, Cotton SM, Pearce T, Newman B, McCutcheon L. Treating co-occurring first-episode psychosis and borderline personality: a pilot randomized controlled trial. Early Intervention in Psychiatry. 2012;6(1):21-9. | Yes | Yes | No | Yes | Yes |
| Gollub EL, French P, Loundou A, Latka M, Rogers C, Stein Z. A randomized trial of hierarchical counseling in a short, clinic-based intervention to reduce the risk of sexually transmitted diseases in women. AIDS. 2000;14(9):1249-55. | Yes | Yes | Yes | Yes | No |
| Gozalo PPM, Prakash SM, Qato DMPMPHP, Sloane PDMDMPH, Mor VP. Effect of the Bathing Without a Battle Training Intervention on Bathing-Associated Physical and Verbal Outcomes in Nursing Home Residents with Dementia: A Randomized Crossover Diffusion Study. Journal of the American Geriatrics Society. 2014;62(5):797-804. | Yes | Yes | No | Yes | Yes |
| Greenberg MT, Domitrovich C, Bumbarger B. The Prevention of Mental Disorders in School-Aged Children: Current State of the Field. Prevention & Treatment March. 2001;4:1. | No | Yes | No | Yes | Yes |
| Greenberg RP. Reflections On The Emperor's New Drugs. Prevention & Treatment July. 2002;15:5. | No | No | No | Yes | Yes |
| Gridchyna I, Cloutier A-M, Nkeng L, Craig C, Frise S, Moride Y. Methodological gaps in the assessment of risk minimization interventions: a systematic review. Pharmacoepidemiology & Drug Safety. 2014;23(6):572-9. | Yes | No | No | Yes | No |
| Gringras Ppips, neurodisability, Gamble Crims, Jones APss, Wiggs Lrip, Williamson PRdomfcrnctu, et al. Melatonin for sleep problems in children with neurodevelopmental disorders: randomised double masked placebo controlled trial. BMJ November. 2012;10(345). | Yes | No | Yes | Yes | Yes |
| Gustavsson C, Denison E, von Koch L. Self-management of persistent neck pain: A randomized controlled trial of a multi-component group intervention in primary health care. European Journal of Pain. 2010;14(6):630e1-e11. | Yes | Yes | Yes | Yes | No |
| Gutner CA, Weinberger J, Hofmann SG. The effect of D-cycloserine on subliminal cue exposure in spider fearful individuals. Cognitive Behaviour Therapy. 2012 Dec;41(4):335-44. PubMed PMID: 2012-34459-006. | Yes | No | No | Yes | Yes |
| Hakkennes S, Dodd K. Guideline implementation in allied health professions: a systematic review of the literature. Quality & Safety in Health Care. 2008;17(4):296-300. | Yes | Yes | No | Yes | No |
| Halford WK, Sanders MR, Behrens BC. Can Skills Training Prevent Relationship Problems in At-Risk Couples? Four-Year Effects of a Behavioral Relationship Education Program. Journal of Family Psychology. 2001;15(4):750-68. | Yes | Yes | Yes | Yes | No |
| Hamani CMDP, Pilitsis JMDP, Rughani AIMD, Rosenow JMMD, Patil PGMDP, Slavin KSMD, et al. Deep Brain Stimulation for Obsessive-Compulsive Disorder: Systematic Review and Evidence-Based Guideline Sponsored by the American Society for Stereotactic and Functional Neurosurgery and the Congress of Neurological Surgeons (CNS) and Endorsed by the CNS and American Association of Neurological Surgeons. Neurosurgery. 2014;75(4):327-33. | Yes | No | No | Yes | Yes |
| Hansson H, Rundberg J, Zetterlind U, Johnsson KO, Berglund M. AN INTERVENTION PROGRAM FOR UNIVERSITY STUDENTS WHO HAVE PARENTS WITH ALCOHOL PROBLEMS: A RANDOMIZED CONTROLLED TRIAL. Alcohol & Alcoholism November/December. 2006;41(6):655-63. | Yes | Yes | No | Yes | Yes |
| Harris JEM, Eng JJP, Miller WCP, Dawson ASMD. A Self-Administered Graded Repetitive Arm Supplementary Program (GRASP) Improves Arm Function During Inpatient Stroke Rehabilitation: A Multi-Site Randomized Controlled Trial. Stroke. 2009;40(6):2123-8. | Yes | Yes | Yes | Yes | No |
| Hartling LP, Newton ASP, Liang YP, Jou HMD, Hewson KBMTMTA, Klassen TPMDM, et al. Music to Reduce Pain and Distress in the Pediatric Emergency Department: A Randomized Clinical Trial. JAMA Pediatrics. 2013;167(9):826-35. | Yes | No | Yes | Yes | Yes |
| Harvey EL, Glenny AM, Kirk SFL, Summerbell CD. An updated systematic review of interventions to improve health professionals' management of obesity. Obesity Reviews. 2002;3(1):45-55. | Yes | Yes | No | Yes | No |
| Hatcher SMD, Sharon CM, Coggan CP. Beyond Randomized Controlled Trials in Attempted Suicide Research. Suicide and Life-Threatening Behavior. 2009;39(4):396-407. | No | Yes | No | Yes | Yes |
| Hauer KP, Becker CMDP, Lindemann UP, Beyer NP. Effectiveness of Physical Training on Motor Performance and Fall Prevention in Cognitively Impaired Older Persons: A Systematic Review. American Journal of Physical Medicine & Rehabilitation. 2006;85(10):847-57. | Yes | Yes | No | Yes | No |
| Hawkes AL, Gollschewski S, Lynch BM, Chambers S. A telephone-delivered lifestyle intervention for colorectal cancer survivors 'CanChange': a pilot study. Pscyho-Oncology. 2009;18(4):449-55. | No | Yes | No | Yes | Yes |
| Hebert R, Robichaud L, Roy P-M, Bravo G, Voyer L. Efficacy of a nurse-led multidimensional preventive programme for older people at risk of functional decline. A randomized controlled trial. Age & Ageing. 2001;30(2):147-53. | Yes | Yes | Yes | Yes | No |
| Heffner JLPD, Lewis DFBA, Winhusen TMPD. Preliminary Evidence That Adherence to Counseling Mediates the Effects of Pretreatment Self-efficacy and Motivation on Outcome of a Cessation Attempt in Smokers with ADHD. Nicotine & Tobacco Research. 2013;15(2):393-400. | Yes | No | No | Yes | Yes |
| Hendriks MRCM, Bleijlevens MHCM, van Haastregt JCMP, Crebolder HFJMPMD, Diederiks JPMP, Evers SMAAP, et al. Lack of Effectiveness of a Multidisciplinary Fall-Prevention Program in Elderly People at Risk: A Randomized, Controlled Trial. Journal of the American Geriatrics Society. 2008;56(8):1390-7. | Yes | Yes | Yes | Yes | No |
| Higginson IJ, Booth S. The randomized fast-track trial in palliative care: Role, utility and ethics in the evaluation of interventions in palliative care? Palliative Medicine. 2011;25(8):741-7. | No | Yes | No | Yes | No |
| Hilfinger Messias DKPRNF, Parra-Medina DPMPH, Sharpe PAPMPH, Trevino LM, Koskan AMP, Morales-Campos DP. Promotoras de Salud: Roles, Responsibilities, and Contributions in a Multisite Community-Based Randomized Controlled Trial. Hispanic Health Care International. 2013;11(2):62-71. | Yes | Yes | Yes | Yes | No |
| Hivert MF, Langlois MF, Berard P, Cuerrier JP, Carpentier AC. Prevention of weight gain in young adults through a seminar-based intervention program. International Journal of Obesity. 2007;31(8):1262-9. | Yes | Yes | Yes | Yes | No |
| Hodnett EDp, Stremler Rap, Willan ARss, Weston JAstc, Lowe NKp, Simpson KRcns, et al. Effect on birth outcomes of a formalised approach to care in hospital labour assessment units: international, randomised controlled trial. BMJ. 2008;337(7670):618-25. | Yes | Yes | Yes | Yes | No |
| Holling H, van Breukelen GJP. Optimal Experimental Design With Nesting of Persons in Organizations. Zeitschrift fur Psychologie/Journal of Psychology. 2013;221(3):145-59. | No | No | Yes | Yes | No |
| Hu W, Adey P, Jia X, Liu J, Zhang L, Li J, et al. Effects of a 'Learn to Think' intervention programme on primary school students. British Journal of Educational Psychology. 2011;81(4):531-57. | No | Yes | Yes | Yes | No |
| Huculak S. The placebo effect in psychiatry: problem or solution? Journal of Medical Ethics. 2014;40(6):376-80. | No | No | No | Yes | Yes |
| Humphreys K, Blodgett JC, Wagner TH. Estimating the Efficacy of Alcoholics Anonymous without Self-Selection Bias: An Instrumental Variables Re-Analysis of Randomized Clinical Trials. Alcoholism: Clinical & Experimental Research. 2014;38(11):2688-94. | Yes | Yes | No | Yes | Yes |
| Ialongo NS, Werthamer L, Kellam SG, Brown CH, Wang S, Lin Y. Proximal impact of two first‐grade preventive interventions on the early risk behaviors for later substance abuse, depression, and antisocial behavior. American journal of community psychology. 1999 Oct;27(5):599-641. | N/A | N/A | N/A | N/A | N/A |
| Inauen J, Tobias R, Mosler H-J. The role of commitment strength in enhancing safe water consumption: Mediation analysis of a cluster-randomized trial. British Journal of Health Psychology. 2014;19(4):701-19. | Yes | Yes | No | Yes | Yes |
| Irwin MLPMPH, Cadmus LP, Alvarez-Reeves MMS, O'Neil MMD, Mierzejewski EMS, Latka RMPH, et al. Recruiting and Retaining Breast Cancer Survivors Into A Randomized Controlled Exercise Trial: The Yale Exercise and Survivorship Study. Cancer. 2008;112 Supplement(11):2593-606. | Yes | Yes | No | Yes | No |
| Javitt DC. Glutamate as a therapeutic target in psychiatric disorders. Molecular Psychiatry. 2004;9(11):984-97. | No | No | No | Yes | Yes |
| Jemmott JBIIIP, Jemmott LSRNP, O'Leary AP, Ngwane ZP, Icard LDP, Heeren GAMDP, et al. Cluster-Randomized Controlled Trial of an HIV/Sexually Transmitted Infection Risk-Reduction Intervention for South African Men. American Journal of Public Health. 2014;104(3):467-73. | Yes | Yes | Yes | Yes | No |
| Jensen PS. A 14-month randomized clinical trial of treatment strategies for attention-deficit/hyperactivity disorder. Archives of general psychiatry. 1999 Dec;56(12):1073-86. | N/A | N/A | N/A | N/A | N/A |
| Jewkes Rd, Nduna Ml, Levin Jcs, Jama Nd, Dunkle Kap, Puren Add, et al. Impact of Stepping Stones on incidence of HIV and HSV-2 and sexual behaviour in rural South Africa: cluster randomised controlled trial. BMJ. 2008;337(7666):391-401. | Yes | Yes | Yes | Yes | No |
| Johnson RAPRNF, Meadows RLDVMD, Haubner JSRNBSN, Sevedge KRNMAACNS. Animal-Assisted Activity Among Patients With Cancer: Effects on Mood, Fatigue, Self-Perceived Health, and Sense of Coherence. Oncology Nursing Forum. 2008;35(2):225-32. | Yes | Yes | Yes | Yes | No |
| Johnson SPCaf, Whitelaw AMDFb, Glazebrook CPCc, Israel CRNRb, Turner RPd, White IRMd, et al. Randomized Trial of a Parenting Intervention for Very Preterm Infants: Outcome at 2 Years. Journal of Pediatrics. 2009;155(4):488-94e1. | N/A | N/A | N/A | N/A | N/A |
| Jones LW, Courneya KS, Fairey AS, Mackey JR. Does the Theory of Planned Behavior Mediate the Effects of an Oncologist's Recommendation to Exercise in Newly Diagnosed Breast Cancer Survivors? Results From a Randomized Controlled Trial. Health Psychology. 2005;24(2):189-97. | Yes | Yes | No | Yes | No |
| Jones SM, Brown JL, Hoglund WL, Aber JL. A School-Randomized Clinical Trial of an Integrated Social-Emotional Learning and Literacy Intervention: Impacts After 1 School Year. Journal of Consulting & Clinical Psychology. 2010;78(6):829-42. | Yes | Yes | No | Yes | No |
| Jung KK, Steil RR. A Randomized Controlled Trial on Cognitive Restructuring and Imagery Modification to Reduce the Feeling of Being Contaminated in Adult Survivors of Childhood Sexual Abuse Suffering from Posttraumatic Stress Disorder. Psychotherapy & Psychosomatics. 2013;82(4):213-20. | Yes | Yes | No | Yes | Yes |
| Jurg ME, Kremers SPJ, Candel MJJM, Van der Wal MF, De Meij JSB. A controlled trial of a school-based environmental intervention to improve physical activity in Dutch children: JUMP-in, kids in motion. Health Promotion International. 2006;21(4):320-30. | Yes | Yes | Yes | Yes | No |
| Juthani-Mehta M, Van Ness PH, McGloin J, Argraves S, Chen S, Charpentier P, et al. A Cluster-Randomized Controlled Trial of a Multicomponent Intervention Protocol for Pneumonia Prevention Among Nursing Home Elders. Clinical Infectious Diseases. 2015;60(6):849-57. | Yes | Yes | Yes | Yes | No |
| Kalichman SC, Cherry C, Cain D, Pope H, Kalichman M, Eaton L, et al. Internet-Based Health Information Consumer Skills Intervention for People Living With HIV/AIDS. Journal of Consulting & Clinical Psychology. 2006;74(3):545-54. | Yes | Yes | Yes | Yes | No |
| Kangovi SMDMS, Mitra NP, Grande DMDMPA, White ML, McCollum S, Sellman JBA, et al. Patient-Centered Community Health Worker Intervention to Improve Posthospital Outcomes: A Randomized Clinical Trial. JAMA Internal Medicine. 2014;174(4):535-43. | Yes | Yes | Yes | Yes | No |
| Kara MPRN. Using the Roper, Logan and Tierney Model in care of people with COPD. Journal of Clinical Nursing. 2007;16(7b):223-33. | Yes | Yes | Yes | Yes | No |
| Kasari C, Freeman S, Paparella T. Joint attention and symbolic play in young children with autism: a randomized controlled intervention study. Journal of Child Psychology & Psychiatry. 2006;47(6):611-20. | Yes | Yes | No | Yes | Yes |
| Keeton CPPD, Ginsburg GSPD, Drake KLPD, Sakolsky DMDPD, Kendall PCPD, Birmaher BMD, et al. BENEFITS OF CHILD-FOCUSED ANXIETY TREATMENTS FOR PARENTS AND FAMILY FUNCTIONING. Depression and Anxiety. 2013;30(9):865-72. | Yes | Yes | No | Yes | Yes |
| Kemp L, Harris E. The challenges of establishing and researching a sustained nurse home visiting programme within the universal child and family health service system. Journal of Research in Nursing. 2012;17(2):127-38. | Yes | Yes | Yes | Yes | No |
| Kendall PC, Hudson JL, Gosch E, Flannery-Schroeder E, Suveg C. Cognitive-Behavioral Therapy for Anxiety Disordered Youth: A Randomized Clinical Trial Evaluating Child and Family Modalities. Journal of Consulting & Clinical Psychology. 2008;76(2):282-97. | Yes | Yes | No | Yes | Yes |
| Kendall PC, Kessler RC. The Impact of Childhood Psychopathology Interventions on Subsequent Substance Abuse: Policy Implications, Comments, and Recommendations. Journal of Consulting & Clinical Psychology. 2002;70(6):1303-6. | No | Yes | No | Yes | Yes |
| Kennedy AP, Nelson E, Reeves D, Richardson G, Roberts C, Robinson A, et al. A randomised controlled trial to assess the effectiveness and cost of a patient orientated self management approach to chronic inflammatory bowel disease. Gut. 2004;53(11):1639-45. | Yes | Yes | Yes | Yes | No |
| Kerse Nap, Peri Krf, Robinson Eb, Wilkinson Tpogm, von Randow Ms, Kiata Lrf, et al. Does a functional activity programme improve function, quality of life, and falls for residents in long term care? Cluster randomised controlled trial. BMJ. 2008;337(7675):912-8. | Yes | Yes | Yes | Yes | No |
| Khunti K, Gray L, Skinner T, Carey M, et al. Effectiveness of a diabetes education and self management programme (DESMOND) for people with newly diagnosed type 2 diabetes mellitus: three year follow-up of a cluster randomised controlled trial in primary care. BMJ April. 2012;28(344). | Yes | Yes | Yes | Yes | No |
| Khunti K, Stone M, Paul S, Baines J, Gisborne L, Farooqi A, et al. Disease management programme for secondary prevention of coronary heart disease and heart failure in primary care: a cluster randomised controlled trial. Heart. 2007;93(11):1398-405. | Yes | Yes | Yes | Yes | No |
| Kiernan M, Brown SD, Schoffman DE, Lee K, King AC, Taylor CB, et al. Promoting Healthy Weight With "Stability Skills First": A Randomized Trial. Journal of Consulting & Clinical Psychology. 2013;81(2):336-46. | Yes | Yes | Yes | Yes | No |
| Kilbourne AMab, Post EPac, Nossek Ad, Sonel Ed, Drill LJd, Cooley Sd, et al. Service delivery in older patients with bipolar disorder: a review and development of a medical care model. Bipolar Disorders. 2008;10(6):672-83. | Yes | Yes | No | Yes | Yes |
| Kim C-JPRN, Kang D-HPRNF, Smith BAPRNF, Landers KAMA. Cardiopulmonary Responses and Adherence to Exercise in Women Newly Diagnosed With Breast Cancer Undergoing Adjuvant Therapy. Cancer Nursing March/April. 2006;29(2):156-65. | Yes | Yes | No | Yes | No |
| King AIIRNBP, Parsons MRGNBMP, Robinson EM, Jorgensen DP. Assessing the impact of a restorative home care service in New Zealand: a cluster randomised controlled trial. Health & Social Care in the Community. 2012;20(4):365-74. | Yes | Yes | Yes | Yes | No |
| Kinnunen TI, Puhkala J, Raitanen J, Ahonen S, Aittasalo M, Virtanen SM, et al. Effects of dietary counselling on food habits and dietary intake of Finnish pregnant women at increased risk for gestational diabetes - a secondary analysis of a cluster-randomized controlled trial. Maternal and Child Nutrition. 2014;10(2):184-97. | Yes | Yes | No | Yes | No |
| Kircher TTJ, Wormstall H, Muller PH, Schwarzler F, Buchkremer G, Wild K, et al. A randomised trial of a geriatric evaluation and management consultation services in frail hospitalised patients. Age & Ageing. 2007;36(1):36-42. | Yes | Yes | Yes | Yes | No |
| Kisely S, Campbell LA, Scott A, Preston NJ, Xiao J. Randomized and non-randomized evidence for the effect of compulsory community and involuntary out-patient treatment on health service use: systematic review and meta-analysis. Psychological Medicine. 2007;37(1):3-14. | Yes | Yes | No | Yes | Yes |
| Kiuru N, Koivisto P, Mutanen P, Vuori J, Nurmi J-E. How Do Efforts to Enhance Career Preparation Affect Peer Groups? Journal of Research on Adolescence. 2011;21(3):677-90. | No | Yes | Yes | Yes | No |
| Knols R, Aaronson NK, Uebelhart D, Fransen J, Aufdemkampe G. Physical Exercise in Cancer Patients During and After Medical Treatment: A Systematic Review of Randomized and Controlled Clinical Trials. Journal of Clinical Oncology. 2005;23(16):3830-42. | Yes | Yes | No | Yes | No |
| Koniak-Griffin D, Verzemnieks IL, Anderson NLR, Brecht M-L, Lesser J, Kim S, et al. Nurse Visitation for Adolescent Mothers: Two-Year Infant Health and Maternal Outcomes. Nursing Research March/April. 2003;52(2):127-36. | Yes | Yes | Yes | Yes | No |
| Konstantinou KPMa, Foster NDPb, Rushton AEMc, Baxter DDd, Wright CCMe, Breen APDCf. FLEXION MOBILIZATIONS WITH MOVEMENT TECHNIQUES: THE IMMEDIATE EFFECTS ON RANGE OF MOVEMENT AND PAIN IN SUBJECTS WITH LOW BACK PAIN. Journal of Manipulative & Physiological Therapeutics March/April. 2007;30(3):178-85. | Yes | Yes | Yes | Yes | No |
| Kornblith ABP, Dowell JMMS, Herndon JEIIP, Engelman BJMS, Bauer-Wu SDRN, Small EJMD, et al. Telephone monitoring of distress in patients aged 65 years or older with advanced stage cancer: A cancer and leukemia group B study. Cancer. 2006;107(11):2706-14. | Yes | Yes | No | Yes | Yes |
| Kravitz RLa, Tancredi DJb, Grennan Tc, Kalauokalani Dd, Street RLJe, Slee CKf, et al. Cancer Health Empowerment for Living without Pain (Ca-HELP): effects of a tailored education and coaching intervention on pain and impairment. Pain. 2011;152(7):1572-82. | Yes | Yes | Yes | Yes | No |
| Kuttner L, Bowman M, Teasdale M. Psychological treatment of distress, pain, and anxiety for young children with cancer. Journal of Developmental and Behavioral Pediatrics. 1988 Dec. | N/A | N/A | N/A | N/A | N/A |
| LaFrance WCJMDMPH, Keitner GIM, Papandonatos GDP, Blum ASMDP, Machan JTP, Ryan CEP, et al. Pilot pharmacologic randomized controlled trial for psychogenic nonepileptic seizures (e-Pub ahead of print) (LOE Classification). Neurology. 2010;75(13):1166-73. | Yes | No | No | Yes | Yes |
| Laidlaw K, Davidson K, Toner H, Jackson G, Clark S, Law J, et al. A randomised controlled trial of cognitive behaviour therapy vs treatment as usual in the treatment of mild to moderate late life depression. International Journal of Geriatric Psychiatry. 2008;23(8):843-50. | Yes | Yes | No | Yes | Yes |
| Lambert-Kerzner AM, Del Giacco EJMD, Fahdi IEMD, Bryson CLMDMS, Melnyk SDPMHS, Bosworth HBP, et al. Patient-Centered Adherence Intervention After Acute Coronary Syndrome Hospitalization. Circulation: Cardiovascular Quality & Outcomes. 2012;5(4):571-6. | Yes | Yes | Yes | Yes | No |
| Lancee JPD, van den Bout JPD, van Straten APD, Spoormaker VIPD. BASELINE DEPRESSION LEVELS DO NOT AFFECT EFFICACY OF COGNITIVE-BEHAVIORAL SELF-HELP TREATMENT FOR INSOMNIA. Depression and Anxiety. 2013;30(2):149-56. | No | Yes | No | Yes | Yes |
| Lapane KLP, Hughes CMP, Daiello LAP, Cameron KARMPH, Feinberg JPJD. Effect of a Pharmacist-Led Multicomponent Intervention Focusing on the Medication Monitoring Phase to Prevent Potential Adverse Drug Events in Nursing Homes. Journal of the American Geriatrics Society. 2011;59(7):1238-45. | Yes | Yes | Yes | Yes | No |
| Latimer AE, Ginis KAM, Arbour KP. The Efficacy of an Implementation Intention Intervention for Promoting Physical Activity Among Individuals With Spinal Cord Injury: A Randomized Controlled Trial. Rehabilitation Psychology. 2006;51(4):273-80. | Yes | Yes | Yes | Yes | No |
| Latimer EAP, Lecomte TP, Becker DRM, Drake REMP, Duclos IP, Piat MP, et al. Generalisability of the individual placement and support model of supported employment: results of a Canadian randomised controlled trial. British Journal of Psychiatry. 2006;189(1):65-73. | Yes | Yes | No | Yes | Yes |
| Lawton BAd, Rose SBrf, Elley CRsl, Dowell ACp, Fenton Ae, Moyes SAb. Exercise on prescription for women aged 40-74 recruited through primary care: two year randomised controlled trial. BMJ. 2009;338(7686):88-94. | Yes | Yes | Yes | Yes | No |
| Lazovich D, Murray DM, Brosseau LM, Parker DL, Milton FT, Dugan SK. Sample Size Considerations for Studies of Intervention Efficacy in the Occupational Setting. Annals of Occupational Hygiene. 2002;46(2):219-27. | Yes | Yes | Yes | Yes | No |
| Leahey TMP, Thomas GP, Fava JLP, Subak LLMD, Schembri MBS, Krupel KMS, et al. Adding Evidence-Based Behavioral Weight Loss Strategies to a Statewide Wellness Campaign: A Randomized Clinical Trial. American Journal of Public Health. 2014;104(7):1300-6. | Yes | Yes | Yes | Yes | No |
| Lederman RPP, Mian TSMD. The Parent-Adolescent Relationship Education (PARE) Program: A Curriculum for Prevention of STDs and Pregnancy in Middle School Youth. Behavioral Medicine Spring. 2003;29(1):33-41. | Yes | Yes | Yes | Yes | No |
| Lee L-L, Kuo Y-C, Fanaw D, Perng S-J, Juang I-F. The effect of an intervention combining self-efficacy theory and pedometers on promoting physical activity among adolescents. Journal of Clinical Nursing. 2012;21(7-8):914-22. | Yes | Yes | Yes | Yes | No |
| Lee M, Shafran R, Burgess C, Carpenter J, Millard E, Thorpe S. The induction of mental and contact contamination. Clinical Psychologist. 2013 Mar;17(1):9-16. PubMed PMID: 2013-09251-002. | No | No | No | Yes | No |
| Legare F, Turcotte S, Stacey D, Ratte S, Kryworuchko J, Graham ID. Patients' Perceptions of Sharing in Decisions: A Systematic Review of Interventions to Enhance Shared Decision Making in Routine Clinical Practice. The Patient: Patient-Centered Outcomes Research. 2012;5(1):1-19. | Yes | Yes | No | Yes | No |
| LeMay S, Johnston C, Choiniere M, Fortin C, Hubert I, Frechette G, et al. Pain management interventions with parents in the emergency department: a randomized trial. Journal of Advanced Nursing. 2010;66(11):2442-9. | Yes | Yes | Yes | Yes | No |
| Levitan MN, Papelbaum M, Nardi AE. A Review of Preliminary Observations on Agomelatine in the Treatment of Anxiety Disorders. Experimental & Clinical Psychopharmacology. 2012;20(6):504-9. | Yes | No | No | Yes | Yes |
| Li LP, Wu ZP, Liang L-JP, Lin CP, Guan JMD, Jia MMD, et al. Reducing HIV-Related Stigma in Health Care Settings: A Randomized Controlled Trial in China. American Journal of Public Health. 2013;103(2):286-92. | Yes | Yes | Yes | Yes | No |
| Li Q, Babor TF, Zeigler D, Xuan Z, Morisky D, Hovell MF, et al. Health promotion interventions and policies addressing excessive alcohol use: a systematic review of national and global evidence as a guide to health-care reform in China. Addiction. 2015;110 Supplement(1):68-78. | Yes | Yes | No | Yes | Yes |
| Lin W-C, Yuan S-C, Chien J-Y, Weng S-C, Chou M-C, Kuo H-W. The effects of respiratory training for chronic obstructive pulmonary disease patients: a randomised clinical trial. Journal of Clinical Nursing. 2012;21(19pt20):2870-8. | Yes | Yes | Yes | Yes | No |
| Lindsay B, Bradley PM. Cochrane Review: Care delivery and self-management strategies for children with epilepsy. Evidence-Based Child Health: A Cochrane Review Journal. 2012;7(1):220-38. | Yes | Yes | Yes | Yes | No |
| Lipman EL, Boyle MH. Social support and education groups for single mothers: a randomized controlled trial of a community-based program. CMAJ Canadian Medical Association Journal. 2005;173(12):1451-6. | Yes | Yes | No | Yes | Yes |
| Lipman ELMD, Boyle MHPD, Cunningham CPD, Kenny MMA, Sniderman CMS, Duku EMS, et al. Testing Effectiveness of a Community-Based Aggression Management Program for Children 7 to 11 Years Old and Their Families. Journal of the American Academy of Child & Adolescent Psychiatry. 2006;45(9):1085-93. | Yes | Yes | No | Yes | Yes |
| Liu JLY, Wyatt JC. The case for randomized controlled trials to assess the impact of clinical information systems. Journal of the American Medical Informatics Association. 2011;18(2):173-80. | No | No | Yes | Yes | No |
| Loeb KL, Wilson GT, Labouvie E, Pratt EM, Hayaki J, Walsh BT, et al. Therapeutic Alliance and Treatment Adherence in Two Interventions for Bulimia Nervosa: A Study of Process and Outcome. Journal of Consulting & Clinical Psychology. 2005;73(6):1097-106. | Yes | Yes | No | Yes | Yes |
| Lombard Csrf, Deeks Asrf, Jolley Dapib, Ball Kapibe, Teede Hpiwsh. A low intensity, community based lifestyle programme to prevent weight gain in women with young children: cluster randomised controlled trial. BMJ July. 2010;17(341). | Yes | Yes | Yes | Yes | No |
| Long AB, Donelson RMD, Fung TP. Does it Matter Which Exercise?: A Randomized Control Trial of Exercise for Low Back Pain. Spine. 2004;29(23):2593-602. | Yes | Yes | Yes | Yes | No |
| Lord C, Wagner A, Rogers S, Szatmari P, Aman M, Charman T, et al. Challenges in Evaluating Psychosocial Interventions for Autistic Spectrum Disorders. Journal of Autism & Developmental Disorders. 2005;35(6):695-708. | No | Yes | Yes | Yes | Yes |
| Lord SRP, Castell SDRGRT, Corcoran JBSGDIM, Dayhew JBS, Matters BBA, Shan AB, et al. The Effect of Group Exercise on Physical Functioning and Falls in Frail Older People Living in Retirement Villages: A Randomized, Controlled Trial. Journal of the American Geriatrics Society. 2003;51(12):1685-92. | Yes | Yes | Yes | Yes | No |
| Lorenz RAPD, Gooneratne NMD, Cole CSPD, Kleban MHPD, Kalra GKMS, Richards KCPD. Exercise and Social Activity Improve Everyday Function in Long-Term Care Residents. American Journal of Geriatric Psychiatry. 2012;20(6):468-76. | Yes | Yes | Yes | Yes | No |
| Macleod M, Craigie AM, Barton KL, Treweek S, Anderson AS, on behalf of the WeighWell t. Recruiting and retaining postpartum women from areas of social disadvantage in a weight-loss trial - an assessment of strategies employed in the WeighWell feasibility study. Maternal and Child Nutrition. 2013;9(3):322-31. | Yes | Yes | Yes | Yes | No |
| Mahoney JEMD, Shea TAPT, Przybelski RMDMS, Jaros LMPA, Gangnon RP, Cech SRN, et al. Kenosha County Falls Prevention Study: A Randomized, Controlled Trial of an Intermediate-Intensity, Community-Based Multifactorial Falls Intervention. Journal of the American Geriatrics Society. 2007;55(4):489-98. | Yes | Yes | Yes | Yes | No |
| Mak SSBRNMN, Zee CYBMP, Molassiotis ARNP, Chan SJRNBONCM, Leung SM, Mo KFBM, et al. A Comparison of Wound Treatments in Nasopharyngeal Cancer Patients Receiving Radiation Therapy. Cancer Nursing November/December. 2005;28(6):436-545. | Yes | No | No | Yes | No |
| Manios Y, Androutsos O, Katsarou C, Iotova V, Socha P, Geyer C, et al. Designing and implementing a kindergarten-based, family-involved intervention to prevent obesity in early childhood: the ToyBox-study. Obesity Reviews. 2014;15 Supplement(3):5-13. | Yes | Yes | Yes | Yes | No |
| Marcus JL, Buisker T, Horvath T, Amico KR, Fuchs JD, Buchbinder SP, et al. Helping our patients take HIV pre-exposure prophylaxis (PrEP): a systematic review of adherence interventions. HIV Medicine. 2014;15(7):385-95. | Yes | Yes | Yes | Yes | No |
| Marshall PWMP, Kennedy SB, Brooks CB, Lonsdale CP. Pilates Exercise or Stationary Cycling for Chronic Nonspecific Low Back Pain: Does it Matter? A Randomized Controlled Trial With 6-Month Follow-up. Spine. 2013;38(15):E952-E9. | Yes | Yes | Yes | Yes | No |
| Marshall SJ, Nicaise V, Ji M, Huerta C, Haubenstricker J, Levy SS, et al. Using Step Cadence Goals to Increase Moderate-to-Vigorous-Intensity Physical Activity. Medicine & Science in Sports & Exercise. 2013;45(3):592-602. | Yes | Yes | Yes | Yes | No |
| Martiniuk ALC, O'Connor KS, King WD. A cluster randomized trial of a sex education programme in Belize, Central America. International Journal of Epidemiology. 2003;32(1):131-6. | Yes | Yes | Yes | Yes | No |
| Masi G, Millepiedi S, Perugi G, Pfanner C, Berloffa S, Pari C, et al. Pharmacotherapy in Paediatric Obsessive-Compulsive Disorder: A Naturalistic, Retrospective Study. CNS Drugs. 2009;23(3):241-52. | No | No | No | Yes | Yes |
| Mastrangelo AMPTP, Conway DMPT, Legendre DMPT, Canella CSPT, House LSPT, Kondos LSPT, et al. Quality of Life Issues During the Menopause Transition. Journal of Women's Health Physical Therapy Winter. 2006;30(3):6-12. | Yes | No | No | Yes | No |
| Mataix-Cols D, Marks IM, Greist JH, Kobak KA, Baer L. Obsessive-compulsive symptom dimensions as predictors of compliance with and response to behaviour therapy: Results from a controlled trial. Psychotherapy and Psychosomatics. 2002 Sep-Oct;71(5):255-62. PubMed PMID: 2002-04173-003. | Yes | Yes | No | Yes | Yes |
| Mathes T, Pieper D, Antoine SL, Eikermann M. Adherence-enhancing interventions for highly active antiretroviral therapy in HIV-infected patients - a systematic review. HIV Medicine. 2013;14(10):583-95. | No | Yes | Yes | Yes | Yes |
| Mathieu E, McGeechan K, Barratt A, Herbert R. Internet-based randomized controlled trials: a systematic review. Journal of the American Medical Informatics Association. 2013;20(3):568-76. | No | Yes | Yes | Yes | No |
| Mbeba MMRNMSN, Kaponda CPNMRNMRMP, Jere DLRNM, Kachingwe SIMSN, Crittenden KSP, McCreary LLRNP, et al. Peer Group Intervention Reduces Personal HIV Risk for Malawian Health Workers. Journal of Nursing Scholarship. 2011;43(1):72-81. | Yes | Yes | Yes | Yes | No |
| McClure EA, Gipson CD, Malcolm RJ, Kalivas PW, Gray KM. Potential Role of N-Acetylcysteine in the Management of Substance Use Disorders. CNS Drugs. 2014;28(2):95-106. | No | No | No | Yes | Yes |
| McCluskey S, Burton AK, Main CJ. The implementation of occupational health guidelines principles for reducing sickness absence due to musculoskeletal disorders. Occupational Medicine (Oxford). 2006;56(4):237-42. | No | Yes | Yes | Yes | No |
| McCusker JMDD, Verdon JMD, Tousignant PMDM, de Courval LPMDDPH, Dendukuri NP, Belzile EM. Rapid Emergency Department Intervention for Older People Reduces Risk of Functional Decline: Results of a Multicenter Randomized Trial. Journal of the American Geriatrics Society. 2001;49(10):1272-81. | Yes | Yes | Yes | Yes | No |
| McGowan L, Cooke LJ, Gardner B, Beeken RJ, Croker H, Wardle J. Healthy feeding habits: efficacy results from a cluster-randomized, controlled exploratory trial of a novel, habit-based intervention with parents1-3. The American Journal of Clinical Nutrition. 2013;98(3):769-77. | Yes | Yes | Yes | Yes | No |
| McGuire R, Waltman N, Zimmerman L. Intervention components promoting adherence to strength training exercise in breast cancer survivors with bone loss. Western Journal of Nursing Research. 2011 Aug;33(5):671-89. PubMed PMID: 2011-13608-005. | Yes | Yes | No | Yes | Yes |
| McKellar J, Wagner T, Harris A, Oehlert M, Buckley S, Moos R. One-year outcomes of telephone case monitoring for patients with substance use disorder. Addictive Behaviors. 2012 Oct;37(10):1069-74. PubMed PMID: 2012-14448-001. | Yes | Yes | No | Yes | Yes |
| Meng K, Musekamp G, Seekatz B, Glatz J, Karger G, Kiwus U, et al. Evaluation of a self-management patient education program for patients with chronic heart failure undergoing inpatient cardiac rehabilitation: study protocol of a cluster randomized controlled trial. BMC Cardiovascular Disorders. 2013;13:60. PubMed PMID: 23968340. Pubmed Central PMCID: PMC3765303. | Yes | Yes | Yes | Yes | No |
| Mertz DMD, Dafoe NRN, Walter SDP, Brazil KP, Loeb MMDM. Effect of a Multifaceted Intervention on Adherence to Hand Hygiene among Healthcare Workers: A Cluster-Randomized Trial. Infection Control & Hospital Epidemiology. 2010;31(11):1170-6. | Yes | Yes | No | Yes | No |
| Merz CJ, Tabbert K, Schweckendiek J, Klucken T, Vaitl D, Stark R, et al. Neuronal correlates of extinction learning are modulated by sex hormones. Social Cognitive & Affective Neuroscience. 2012;7(7):819-30. | No | No | No | Yes | No |
| Merz CJab, Tabbert Kb, Schweckendiek Jab, Klucken Tab, Vaitl Db, Stark Rab, et al. Oral contraceptive usage alters the effects of cortisol on implicit fear learning. Hormones & Behavior. 2012;62(4):531-8. | No | No | No | Yes | No |
| Metzelthin SFsr, van Rossum Eliiicffe, de Witte LPpotic, Ambergen AWs, Hobma SOgp, Sipers Wg, et al. Effectiveness of interdisciplinary primary care approach to reduce disability in community dwelling frail older people: cluster randomised controlled trial. BMJ September. 2013;14(347). | Yes | Yes | Yes | Yes | No |
| Milos Vab, Jakobsson Ub, Westerlund Tcd, Melander Ee, Molstad Sb, Midlov Pb. Theory-based interventions to reduce prescription of antibiotics-a randomized controlled trial in Sweden. Family Practice. 2013;30(6):634-40. | Yes | Yes | Yes | Yes | No |
| Moffatt FWRNP, Hodnett ERNPF, Esplen MJRNP, Watt-Watson JRNP. Effects of Guided Imagery on Blood Pressure in Pregnant Women with Hypertension: A Pilot Randomized Controlled Trial. Birth. 2010;37(4):296-306. | Yes | Yes | Yes | Yes | No |
| Mohammadzadeh A, Rezaie A, Yaghoubi H, Pirkhaefi A. The relationship between obsessive compulsive and schizotypal personality features among university students. Iranian Journal of Psychiatry and Clinical Psychology. 2011 Win;16(4):490-5. PubMed PMID: 2011-26327-001. | No | No | No | No | Yes |
| Mohr DCP, Lovera JMD, Brown TMD, Cohen BMD, Neylan TMD, Henry RP, et al. A randomized trial of stress management for the prevention of new brain lesions in MS. Neurology. 2012;79(5):412-9. | Yes | Yes | No | Yes | Yes |
| Montgomery AAslipcr, Emmett CLtc, Fahey Tpogp, Jones Cra, Ricketts Ipoas, healthcare c, et al. Two decision aids for mode of delivery among women with previous caesarean section: randomised controlled trial. BMJ. 2007;334(7607):1305-12. | Yes | Yes | Yes | Yes | No |
| Montgomery L, Burlew AK, Kosinski AS, Forcehimes AA. Motivational Enhancement Therapy for African American Substance Users: A Randomized Clinical Trial. Cultural Diversity & Ethnic Minority Psychology. 2011;17(4):357-65. | Yes | Yes | No | Yes | Yes |
| Morgan M, Studney DR, Barnett GO, Winickoff RN. Computerized concurrent review of prenatal care. QRB Qual Rev Bull. 1978;4(9):33-6. | N/A | N/A | N/A | N/A | N/A |
| Morrison-Beedy D, Carey MP, Seibold-Simpson SM, Xia Y, Tu X. Preliminary Efficacy of a Comprehensive HIV Prevention Intervention for Abstinent Adolescent Girls: Pilot Study Findings. Research in Nursing & Health. 2009;32(6):569-81. | Yes | Yes | Yes | Yes | No |
| Morthorst Ba, Krogh Ja, Erlangsen Ac, Alberdi Fd, Nordentoft Me. Effect of assertive outreach after suicide attempt in the AID (assertive intervention for deliberate self harm) trial: randomised controlled trial. BMJ August. 2012;25(345). | Yes | Yes | No | Yes | Yes |
| Moyer A, Sohl SJ, Knapp-Oliver SK, Schneider S. Characteristics and methodological quality of 25 years of research investigating psychosocial interventions for cancer patients. Cancer Treatment Reviews. 2009 Aug;35(5):475-84. PubMed PMID: 19264411. Pubmed Central PMCID: NIHMS95921 | No | Yes | Yes | Yes | No |
| Moyer A. The psychology of human research participation. Handbook of the psychology of science. New York, NY: Springer Publishing Co; US; 2013. p. 419-36. | No | No | No | Yes | No |
| Moyer AP, Knapp-Oliver SKP, Sohl SJP, Schnieder SDP, Floyd AHLP. Lessons to Be Learned From 25 Years of Research Investigating Psychosocial Interventions for Cancer Patients. Cancer Journal September/October. 2009;15(5):345-51. | Yes | Yes | Yes | Yes | No |
| Mudge AM, Denaro CP, Scott AC, Atherton JJ, Meyers DE, Marwick TH, et al. Exercise training in recently hospitalized heart failure patients enrolled in a disease management programme: design of the EJECTION-HF randomized controlled trial. European Journal of Heart Failure. 2011;13(12):1370-5. | Yes | Yes | Yes | Yes | No |
| Mudge AM, Maussen C, Duncan J, Denaro CP. Improving quality of delirium care in a general medical service with established interdisciplinary care: a controlled trial. Internal Medicine Journal. 2013;43(3):270-7. | No | Yes | Yes | Yes | Yes |
| Mullen SP, Wojcicki TR, Mailey EL, Szabo AN, Gothe NP, Olson EA, et al. A profile for predicting attrition from exercise in older adults. Prevention Science. 2013 Oct;14(5):489-96. PubMed PMID: 2013-05811-001. | Yes | Yes | No | Yes | No |
| Multiple Risk Factor Intervention Trial Research Group. Multiple Risk Factor Intervention Trial: risk factor changes and mortality results. Jama. 1982;248:1465-77. | N/A | N/A | N/A | N/A | N/A |
| Muthukumaraswamy SDa, Myers JFMb, Wilson SJc, Nutt DJc, Lingford-Hughes Ac, Singh KDa, et al. The effects of elevated endogenous GABA levels on movement-related network oscillations. Neuroimage. 2013;66 Supplement(C):36-41. | No | No | No | Yes | No |
| Nakamura Y, Donaldson GW, Kuhn R, Bradshaw DH, Jacobson RC, Chapman RC. Investigating dose-dependent effects of placebo analgesia: A psychophysiological approach. Pain. 2012;153(1):227-37. | No | No | No | Yes | No |
| Norris SLMDMPH, Engelgau MMMDMSC, Narayan KMVMDMPH. Effectiveness of Self-Management Training in Type 2 Diabetes: A systematic review of randomized controlled trials. Diabetes Care. 2001;24(3):561-87. | Yes | Yes | No | Yes | No |
| Norris SLMDMPH, Lau JMD, Smith SJMISMSC, Schmid CHPHD, Engelgau MMMDMSC. Self-Management Education for Adults With Type 2 Diabetes: A meta-analysis of the effect on glycemic control. Diabetes Care. 2002;25(7):1159-71. | Yes | Yes | No | Yes | No |
| Olatunji BO, Armstrong T. Contamination Fear and Effects of Disgust on Distress in a Public Restroom. Emotion. 2009;9(4):592-7. | No | No | No | Yes | No |
| Olatunji BO, Rosenfield D, Tart CD, Cottraux J, Powers MB, Smits JA. Behavioral Versus Cognitive Treatment of Obsessive-Compulsive Disorder: An Examination of Outcome and Mediators of Change. Journal of Consulting & Clinical Psychology. 2013;81(3):415-28. | Yes | Yes | No | Yes | Yes |
| Olino TMPD, Gillo SMA, Rowe D, Palermo SMPH, Nuhfer ECBS, Birmaher BMD, et al. EVIDENCE FOR SUCCESSFUL IMPLEMENTATION OF EXPOSURE AND RESPONSE PREVENTION IN A NATURALISTIC GROUP FORMAT FOR PEDIATRIC OCD+. Depression and Anxiety. 2011;28(4):342-8. | No | Yes | No | Yes | Yes |
| Oliver-Africano P, Dickens S, Ahmed Z, Bouras N, Cooray S, Deb S, et al. Overcoming the barriers experienced in conducting a medication trial in adults with aggressive challenging behaviour and intellectual disabilities. Journal of Intellectual Disability Research. 2010;54(1):17-25. | Yes | No | Yes | Yes | Yes |
| Orgogozo J-MMD, Rigaud A-SMDP, Stoffler AMD, Mobius H-JMD, Forette FMD. Efficacy and Safety of Memantine in Patients With Mild to Moderate Vascular Dementia: A Randomized, Placebo-Controlled Trial (MMM 300). Stroke. 2002;33(7):1834-9. | Yes | No | No | Yes | Yes |
| Orrell M, Aguirre E, Spector A, Hoare Z, Woods RT, Streater A, et al. Maintenance cognitive stimulation therapy for dementia: single-blind, multicentre, pragmatic randomised controlled trial. British Journal of Psychiatry. 2014;204(6):454-61. | Yes | Yes | No | Yes | Yes |
| Ostrowsky BE, Whitener C, Bredenberg HK, Carson LA, Holt S, Hutwagner L, et al. Serratia marcescens Bacteremia Traced to an Infused Narcotic. New England Journal of Medicine. 2002;346(20):1529-37. | No | No | No | Yes | No |
| Pai AB, Boyd A, Chavez A, Manley HJ. Health-related quality of life is maintained in hemodialysis patients receiving pharmaceutical care: A 2-year randomized, controlled study. Hemodialysis International. 2009;13(1):72-9. | Yes | Yes | Yes | Yes | No |
| Patterson PPBARMNRGNCE, Whittington RPBACAP, Bogg JPBAMC. Testing the effectiveness of an educational intervention aimed at changing attitudes to self-harm. Journal of Psychiatric & Mental Health Nursing. 2007;14(1):100-5. | No | Yes | No | Yes | Yes |
| Petersen TPTB, Kryger PMDP, Ekdahl CPTP, Olsen SPTMDT, Jacobsen SMDP. The Effect of McKenzie Therapy as Compared With That of Intensive Strengthening Training for the Treatment of Patients With Subacute or Chronic Low Back Pain: A Randomized Controlled Trial. Spine. 2002;27(16):1702-9. | Yes | Yes | No | Yes | No |
| Piatt GAMC, Orchard TJM, Emerson SCC, Simmons DM, Songer TJP, Brooks MMP, et al. Translating the Chronic Care Model Into the Community: Results from a randomized controlled trial of a multifaceted diabetes care intervention. Diabetes Care. 2006;29(4):811-7. | Yes | Yes | No | Yes | No |
| Pickar DMD, Bartko JJPD. Effect Size of Symptom Status in Withdrawal of Typical Antipsychotics and Subsequent Clozapine Treatment in Patients With Treatment-Resistant Schizophrenia. American Journal of Psychiatry. 2003;160(6):1133-8. | No | No | No | Yes | Yes |
| Pinto BM, Frierson GM, Rabin C, Trunzo JJ, Marcus BH. Home-Based Physical Activity Intervention for Breast Cancer Patients. Journal of Clinical Oncology. 2005;23(15):3577-87. | Yes | Yes | No | Yes | No |
| Pinto BM, Rabin C, Dunsiger S. Home-based exercise among cancer survivors: Adherence and its predictors. Psycho-Oncology. 2009 Apr;18(4):369-76. PubMed PMID: 2009-05653-005. | Yes | Yes | No | Yes | No |
| Plant HPBARGN, Bredin MMARGN, Krishnasamy MMBARGND, Corner JPBRGN. Working with resistance, tension and objectivity: Conducting a randomised controlled trial of a nursing intervention for breathlessness. Journal of Research in Nursing. 2000;5(6):426-34. | No | No | No | Yes | Yes |
| Ponniah K, Magiati I, Hollon SD. An update on the efficacy of psychological treatments for obsessive-compulsive disorder in adults. Journal of Obsessive-Compulsive and Related Disorders. 2013 Apr;2(2):207-18. PubMed PMID: 2014-33295-019. | Yes | Yes | No | Yes | Yes |
| Porter MM. Older Driver Training Using Video and Global Positioning System Technology-a Randomized Controlled Trial. Journals of Gerontology Series A, Biological Sciences & Medical Sciences. 2013;68(5):574-80. | No | Yes | Yes | Yes | No |
| Pradier C, Bentz L, Spire B, Tourette-Turgis C, Morin M, Souville M, et al. Efficacy of an Educational and Counseling Intervention on Adherence to Highly Active Antiretroviral Therapy: French Prospective Controlled Study. HIV Clinical Trials March/April. 2003;4(2):121-31. | Yes | Yes | No | Yes | Yes |
| Puetz TW, Herring MP. Differential effects of exercise on cancer-related fatigue during and following treatment: A meta-analysis. American Journal of Preventive Medicine. 2012 Aug;43(2):e1-e24. PubMed PMID: 2012-19831-004. | Yes | No | No | Yes | No |
| Purcell DW, Garfein RS, Latka MH, Thiede H, Hudson S, Bonner S, et al. Development, description, and acceptability of a small-group, behavioral intervention to prevent HIV and hepatitis C virus infections among young adult injection drug users. Drug & Alcohol Dependence. 2007 Nov;91 Suppl 1:S73-80. PubMed PMID: 17466465. | Yes | Yes | Yes | Yes | No |
| Purcell DWJDP, Latka MHP, Metsch LRP, Latkin CAP, Gomez CAP, Mizuno YP, et al. Results From a Randomized Controlled Trial of a Peer-Mentoring Intervention to Reduce HIV Transmission and Increase Access to Care and Adherence to HIV Medications Among HIV-Seropositive Injection Drug Users. JAIDS Journal of Acquired Immune Deficiency Syndromes. 2007;46 Supplement 2, HIV Prevention and Clinical Care for HIV-Positive Injection Drug(Users):Lessons from the INSPIRE Study:S35-S47. | Yes | Yes | Yes | Yes | No |
| Rachman S, Shafran R, Radomsky AS, Zysk E. Reducing contamination by exposure plus safety behaviour. Journal of Behavior Therapy & Experimental Psychiatry. 2011 Sep;42(3):397-404. PubMed PMID: 21458404. | No | No | No | Yes | Yes |
| Radecki Breitkopf C, Dawson L, Grady JJ, Breitkopf DM, Nelson-Becker C, Snyder RR. Intervention to Improve Follow-Up for Abnormal Papanicolaou Tests: A Randomized Clinical Trial. Health Psychology. 2014;33(4):307-16. | Yes | Yes | Yes | Yes | No |
| Radziewicz RMRNP-BC, Rose JHPMA, Bowman KFP, Berila RAMSNRN, O'Toole EEMD, Given BPRNF. Establishing Treatment Fidelity in a Coping and Communication Support Telephone Intervention for Aging Patients With Advanced Cancer and Their Family Caregivers. Cancer Nursing May/June. 2009;32(3):193-202. | Yes | Yes | No | Yes | Yes |
| Rains JCP, Penzien DBP. Behavioral Research and the Double-Blind Placebo-Controlled Methodology: Challenges in Applying the Biomedical Standard to Behavioral Headache Research. Headache SPECIAL SERIES: HEADACHE RESEARCH METHODOLOGY. 2005;45(5):479-86. | No | Yes | No | Yes | Yes |
| Rapee RM, Kennedy S, Ingram M, Edwards S, Sweeney L. Prevention and Early Intervention of Anxiety Disorders in Inhibited Preschool Children. Journal of Consulting & Clinical Psychology. 2005;73(3):488-97. | Yes | Yes | No | Yes | Yes |
| Reading R, Harvey I, McLean M, on behalf of the CAB, Family Health Study T. Cluster randomised trials in maternal and child health: implications for power and sample size. Archives of Disease in Childhood. 2000;82(1):79-83. | Yes | Yes | No | Yes | No |
| Resnick B, Luisi D, Vogel A. Testing the Senior Exercise Self-efficacy Project (SESEP) for Use with Urban Dwelling Minority Older Adults. Public Health Nursing May/June. 2008;25(3):221-34. | Yes | Yes | Yes | Yes | No |
| Reyna VF, Mills BA. Theoretically Motivated Interventions for Reducing Sexual Risk Taking in Adolescence: A Randomized Controlled Experiment Applying Fuzzy-Trace Theory. Journal of Experimental Psychology: General. 2014;143(4):1627-48. | Yes | Yes | Yes | Yes | No |
| Ribeiro LHS, Prota C, Gomes CM, de Bessa J, Jr., Boldarine MP, Dall'Oglio MF, et al. Long-Term Effect of Early Postoperative Pelvic Floor Biofeedback on Continence in Men Undergoing Radical Prostatectomy: A Prospective, Randomized, Controlled Trial. Journal of Urology. 2010;184(3):1034-9. | Yes | Yes | Yes | Yes | No |
| Rice ME, Harris GT. The size and sign of treatment effects in sex offender therapy. Annals of the New York Academy of Sciences: New York Academy of Sciences; 2003. p. 428-40. | No | Yes | No | Yes | Yes |
| Robbins LB, Gretebeck KA, Kazanis AS, Pender NJ. Girls on the Move Program to Increase Physical Activity Participation. Nursing Research May/June. 2006;55(3):206-16. | Yes | Yes | Yes | Yes | No |
| Roberts LPM, Little PF, Chapman JMM, Cantrell TF, Pickering RP, Langridge JMF. The Back Home Trial: General Practitioner-Supported Leaflets May Change Back Pain Behavior. Spine. 2002;27(17):1821-8. | Yes | No | Yes | Yes | No |
| Robertson AR, St, Morse DT, Baird-Thomas C, Liew H, Gresham K. The Healthy Teen Girls Project: Comparison of health education and STD risk reduction intervention for incarcerated adolescent females. Health Education & Behavior. 2011 Jun;38(3):241-50. PubMed PMID: 2011-10560-004. | Yes | Yes | Yes | Yes | No |
| Rogers LQMDMPH, Anton PMP, Fogleman ABS, Hopkins-Price PP, Verhulst SP, Rao KMDP, et al. Pilot, randomized trial of resistance exercise during radiation therapy for head and neck cancer. Head & Neck. 2013;35(8):1178-88. | Yes | Yes | Yes | Yes | No |
| Rooks DSS, Gautam SP, Romeling MBS, Cross MLBS, Stratigakis DBA, Evans BBS, et al. Group Exercise, Education, and Combination Self-management in Women With Fibromyalgia: A Randomized Trial. Archives of Internal Medicine. 2007;167(20):2192-200. | Yes | Yes | Yes | Yes | No |
| Rooney AG, Brown PD, Reijneveld JC, Grant R. Depression in glioma: a primer for clinicians and researchers. Journal of Neurology, Neurosurgery & Psychiatry. 2014;85(2):230-5. | No | No | No | Yes | Yes |
| Rosen L, Zucker D, Brody D, Engelhard D, Manor O. The effect of a handwashing intervention on preschool educator beliefs, attitudes, knowledge and self-efficacy. Health Education Research. 2009;24(4):686-98. | Yes | Yes | Yes | Yes | No |
| Rosendahl E, Lindelof N, Littbrand H, Yifter-Lindgren E, Lundin-Olsson L, Haglin L, et al. High-intensity functional exercise program and proteinenriched energy supplement for older persons dependent in activities of daily living: A randomised controlled trial. Australian Journal of Physiotherapy. 2006;52(2):105-13. | Yes | Yes | Yes | Yes | No |
| Rotheram-Borus MJ, Wu Z, Liang L-J, Li L, Detels R, Guan J, et al. Reductions in sexually transmitted infections associated with popular opinion leaders in China in a randomised controlled trial. Sexually Transmitted Infections. 2011;87(4):337-43. | Yes | Yes | Yes | Yes | No |
| Rowan CRMPMA, Bick DRMBAMP, Bastos MHdSMMDP. Postnatal Debriefing Interventions to Prevent Maternal Mental Health Problems After Birth: Exploring the Gap Between the Evidence and UK Policy and Practice. Worldviews on Evidence-Based Nursing. 2007;4(2):97-105. | Yes | Yes | No | Yes | Yes |
| Saenger C, Torero M, Qaim M. Impact of Third-party Contract Enforcement in Agricultural Markets-A Field Experiment in Vietnam. American Journal of Agricultural Economics. 2014;96(4):1220-38. | No | No | Yes | Yes | No |
| Safren SA, O'Cleirigh CM, Bullis JR, Otto MW, Stein MD, Pollack MH. Cognitive Behavioral Therapy for Adherence and Depression (CBT-AD) in HIV-Infected Injection Drug Users: A Randomized Controlled Trial. Journal of Consulting & Clinical Psychology. 2012;80(3):404-15. | Yes | Yes | No | Yes | Yes |
| Salmon J, Ball K, Crawford D, Booth M, Telford A, Hume C, et al. Reducing sedentary behaviour and increasing physical activity among 10-year-old children: overview and process evaluation of the 'Switch-Play' intervention. Health Promotion International. 2005;20(1):7-17. | Yes | Yes | Yes | Yes | No |
| Sandbaek A, Griffin SJ, Sharp SJ, Simmons RK, Borch-Johnsen K, Rutten GEHM, et al. Effect of Early Multifactorial Therapy Compared With Routine Care on Microvascular Outcomes at 5 Years in People With Screen-Detected Diabetes: A Randomized Controlled Trial: The ADDITION-Europe Study. Diabetes Care. 2014;37(7):2015-23. | Yes | Yes | Yes | Yes | No |
| Sanford MMBCB, Boyle MPD, McCleary LPD, Miller JBSN, Steele MMD, Duku EMS, et al. A Pilot Study of Adjunctive Family Psychoeducation in Adolescent Major Depression: Feasibility and Treatment Effect. Journal of the American Academy of Child & Adolescent Psychiatry. 2006;45(4):386-495. | Yes | Yes | No | Yes | Yes |
| Schachman KA, Lee RK, Lederma RP. Baby Boot Camp: Facilitating Maternal Role Adaptation Among Military Wives. Nursing Research March/April. 2004;53(2):107-15. | Yes | Yes | Yes | Yes | No |
| Schein CRNM, Gagnon AJRNMPHP, Chan LRNM, Morin IM, Grondines JBS. The Association Between Specific Nurse Case Management Interventions and Elder Health. Journal of the American Geriatrics Society. 2005;53(4):597-602. | No | Yes | No | Yes | No |
| Schnabel M, Ferrari R, Vassiliou T, Kaluza G. Randomised, controlled outcome study of active mobilisation compared with collar therapy for whiplash injury. Emergency Medicine Journal. 2004;21(3):306-10. | Yes | No | Yes | Yes | No |
| Schneider JAMDMPH, Laumann EOP. Alternative Explanations for Negative Findings in the Community Popular Opinion Leader Multisite Trial and Recommendations for Improvements of Health Interventions Through Social Network Analysis. JAIDS Journal of Acquired Immune Deficiency Syndromes. 2011;56(4):e119-e20. | Yes | Yes | Yes | Yes | No |
| Schofield P, Ugalde A, Gough K, Reece J, Krishnasamy M, Carey M, et al. A tailored, supportive care intervention using systematic assessment designed for people with inoperable lung cancer: a randomised controlled trial. Pscyho-Oncology. 2013;22(11):2445-53. | Yes | Yes | Yes | Yes | No |
| Schumacher J, Runte J, Brinker A, Prior K, Heringlake M, Eichler W. Respiratory protection during high-fidelity simulated resuscitation of casualties contaminated with chemical warfare agents *. Anaesthesia. 2008;63(6):593-8. | Yes | No | No | Yes | No |
| Shang JPRNOCN, Wenzel JP, Krumm SP, Griffith KP, Stewart KE. Who Will Drop Out and Who Will Drop In: Exercise Adherence in a Randomized Clinical Trial Among Patients Receiving Active Cancer Treatment. Cancer Nursing July/August. 2012;35(4):312-22. | Yes | Yes | Yes | Yes | No |
| Shaw FEAsSrf, Bond Jp, Richardson DAcra, Dawson Psl, Steen INs, McKeith IGp, et al. Multifactorial intervention after a fall in older people with cognitive impairment and dementia presenting to the accident and emergency department: randomised controlled trial. BMJ. 2003;326(7380):73. | Yes | Yes | Yes | Yes | No |
| Shaw WSP, Feuerstein MP, Lincoln AES, Miller VIMD, Wood PMRNC-SCCM. Case Management Services for Work Related Upper Extremity Disorders: Integrating Workplace Accommodation and Problem Solving. AAOHN Journal. 2001;49(8):378-89. | Yes | Yes | Yes | Yes | No |
| Shepperd SMD, Doll HMD, Angus RMM, Clarke MJMAD, Iliffe SBM, Kalra LMDP, et al. Avoiding hospital admission through provision of hospital care at home: a systematic review and meta-analysis of individual patient data. CMAJ Canadian Medical Association Journal. 2009;180(2):175-82. | No | Yes | No | Yes | No |
| Shirazi KK, Wallace LM, Niknami S, Hidarnia A, Torkaman G, Gilchrist M, et al. A home-based, transtheoretical change model designed strength training intervention to increase exercise to prevent osteoporosis in Iranian women aged 40-65 years: a randomized controlled trial. Health Education Research. 2007;22(3):305-17. | Yes | Yes | Yes | Yes | No |
| Sillem HMCHT, lowast, Backman CLPOTF, Miller WCPOT, Li LCPPT. Comparison of Two Carpometacarpal Stabilizing Splints for Individuals with Thumb Osteoarthritis. Journal of Hand Therapy July/August/September. 2011;24(3):216-26. | Yes | No | Yes | Yes | No |
| Silva MN, Markland D, Carraca EV, Vieira PN, Coutinho SR, Minderico CS, et al. Exercise Autonomous Motivation Predicts 3-yr Weight Loss in Women. Medicine & Science in Sports & Exercise. 2011;43(4):728-37. | Yes | Yes | No | Yes | Yes |
| Simons Ma, Schneider Sb, Herpertz-Dahlmann Ba. Metacognitive Therapy versus Exposure and Response Prevention for Pediatric Obsessive-Compulsive Disorder: A Case Series with Randomized Allocation. Psychotherapy & Psychosomatics. 2006;75(4):257-64. | Yes | Yes | No | Yes | Yes |
| Sjogren T, Nissinen KJ, Jarvenpaa SK, Ojanen MT, Vanharanta H, Malkia EA. Effects of a physical exercise intervention on subjective physical well-being, psychosocial functioning and general well-being among office workers: A cluster randomized-controlled cross-over design. Scandinavian Journal of Medicine & Science in Sports Special topic: Neurobiology and exercise: Does exercise give better well-being in healthy and diseased individuals? 2006;16(6):381-90. | Yes | Yes | Yes | Yes | No |
| Sjogren Tab, Nissinen KJc, Jarvenpaa SKc, Ojanen MTd, Vanharanta He, Malkia EAa. Effects of a workplace physical exercise intervention on the intensity of headache and neck and shoulder symptoms and upper extremity muscular strength of office workers: A cluster randomized controlled cross-over trial. Pain. 2005;116(1-2):119-28. | Yes | Yes | Yes | Yes | No |
| Skidmore ER, Dawson DR, Whyte EM, Butters MA, Amanda Dew M, Grattan ES, et al. Developing complex interventions: lessons learned from a pilot study examining strategy training in acute stroke rehabilitation. Clinical Rehabilitation. 2014;28(4):378-87. | No | Yes | Yes | Yes | No |
| Slack MK, Draugalis JR. Establishing the internal and external validity of experimental studies. American Journal of Health-System Pharmacy. 2001;58(22):2173-84. | No | No | Yes | Yes | No |
| Slater JS, Finnegan JR, Jr., Madigan SD. Incorporation of a Successful Community-Based Mammography Intervention: Dissemination Beyond a Community Trial. Health Psychology. 2005;24(5):463-9. | No | No | Yes | Yes | No |
| Smith J, Forster A, Young J. A randomized trial to evaluate an education programme for patients and carers after stroke. Clinical Rehabilitation. 2004;18(7):726-36. | Yes | Yes | Yes | Yes | No |
| Smith SMapogp, Soubhi Hapofm, Fortin Mpofm, Hudon Capofm, O'Dowd Tpogp. Managing patients with multimorbidity: systematic review of interventions in primary care and community settings. BMJ September. 2012;8(345). | Yes | Yes | No | Yes | Yes |
| Speck BJ, Looney SW. Effects of a Minimal Intervention to Increase Physical Activity in Women: Daily Activity Records. Nursing Research November/December. 2001;50(6):374-8. | Yes | Yes | Yes | Yes | No |
| Speed-Andrews AEP, Rhodes REP, Blanchard CMP, Culos-Reed SNP, Friedenreich CMP, Belanger LJM, et al. Medical, demographic and social cognitive correlates of physical activity in a population-based sample of colorectal cancer survivors. European Journal of Cancer Care. 2012;21(2):187-96. | No | No | No | Yes | No |
| Speirs VRNBN, Johnson MMPRN, Jirojwong SPRNMPH. A systematic review of interventions for homeless women. Journal of Clinical Nursing. 2013;22(7-8):1080-93. | Yes | Yes | Yes | Yes | No |
| Spinewine AP, Swine CMD, Dhillon SP, Lambert PP, Nachega JBMDMPHDTM, H, et al. Effect of a Collaborative Approach on the Quality of Prescribing for Geriatric Inpatients: A Randomized, Controlled Trial. Journal of the American Geriatrics Society. 2007;55(5):658-65. | Yes | Yes | Yes | Yes | No |
| Spreckley MMa, Boyd RPMbc. Efficacy of Applied Behavioral Intervention in Preschool Children with Autism for Improving Cognitive, Language, and Adaptive Behavior: A Systematic Review and Meta-analysis. Journal of Pediatrics. 2009;154(3):338-44. | Yes | Yes | No | Yes | Yes |
| Ssewamala FMP, Han C-KP, Neilands TBP, Ismayilova LP, Sperber EBA. Effect of Economic Assets on Sexual Risk-Taking Intentions Among Orphaned Adolescents in Uganda. American Journal of Public Health. 2010;100(3):483-8. | Yes | Yes | Yes | Yes | No |
| STD Prevention Trial for African American Couples Group. Supervision of facilitators in a multisite study: goals, process, and outcomes. Journal of acquired immune deficiency syndromes (1999). 2008 Sep 1;49(Suppl 1):S59. | Yes | Yes | Yes | Yes | No |
| Steinberg DMPMSRD, Askew SMPH, Lanpher MGBA, Foley PBMPHMSW, Levine ELMPH, Bennett GGP. The Effect of a "Maintain, Don't Gain" Approach to Weight Management on Depression Among Black Women: Results From a Randomized Controlled Trial. American Journal of Public Health. 2014;104(9):1766-73. | Yes | Yes | No | Yes | Yes |
| Stepans MBFPRN, Wilhelm SLPRNC, Dolence KP. Smoking Hygiene: Reducing Infant Exposure to Tobacco. Biological Research for Nursing. 2006;8(2):104-14. | Yes | Yes | Yes | Yes | No |
| Strauman TJ, Vieth AZ, Merrill KA, Kolden GG, Woods TE, Klein MH, et al. Self-System Therapy as an Intervention for Self-Regulatory Dysfunction in Depression: A Randomized Comparison With Cognitive Therapy. Journal of Consulting & Clinical Psychology. 2006;74(2):367-76. | Yes | Yes | No | Yes | Yes |
| Sulch D, Melbourn A, Perez I, Kalra L. Integrated care pathways and quality of life on a stroke rehabilitation unit. Stroke. 2002 Jun 1;33(6):1600-4. | Yes | Yes | Yes | Yes | No |
| Sulch D, Perez I, Melbourn A, Kalra L. Randomized controlled trial of integrated (managed) care pathway for stroke rehabilitation. Stroke. 2000 Aug;31(8):1929-34. | Yes | Yes | Yes | Yes | No |
| Sullivan-Bolyai SDCNSRN, Bova CPRNANP, Lee MMD, Gruppuso PAMD. Mentoring Fathers of Children Newly Diagnosed with T1DM. MCN, American Journal of Maternal Child Nursing July/August. 2011;36(4):224-31. | Yes | Yes | Yes | Yes | No |
| Swenson KKPRNA, Nissen MJPMPH, Henly SJPRN. Physical Activity in Women Receiving Chemotherapy for Breast Cancer: Adherence to a Walking Intervention. Oncology Nursing Forum. 2010;37(3):321-30. | No | Yes | No | Yes | Yes |
| Tabet N, Howard R. Non-pharmacological interventions in the prevention of delirium. Age & Ageing. 2009;38(4):374-9. | No | No | Yes | Yes | Yes |
| Talley NJ. Evaluation of drug treatment in irritable bowel syndrome. British Journal of Clinical Pharmacology. 2003;56(4):362-9. | No | No | No | Yes | No |
| Tamagawa R, Moss-Morris R, Martin A, Robinson E, Booth RJ. Dispositional emotion coping styles and physiological responses to expressive writing. British Journal of Health Psychology. 2013;18(3):574-92. | Yes | Yes | No | Yes | Yes |
| Tansella M, Thornicroft G, Barbui C, Cipriani A, Saraceno B. Seven criteria for improving effectiveness trials in psychiatry. Psychological Medicine. 2006;36(5):711-20. | No | No | Yes | Yes | Yes |
| Tate D, Kalpakjian C, Kwon C. The Use of Randomized Clinical Trials in Rehabilitation Psychology. Rehabilitation Psychology. 2008;53(3):268-78. | No | No | No | Yes | Yes |
| Taylor CL, Demoor C, Smith MA, Dunn AL, Basen-Engquist K, Nielsen I, et al. Active for life after cancer: A randomized trial examining a lifestyle physical activity program for prostate cancer patients. Psycho-Oncology. 2006 Oct;15(10):847-62. PubMed PMID: 2006-20755-001. | Yes | No | No | Yes | No |
| Taylor JL, Novaco RW, Gillmer BT, Robertson A, Thorne I. Individual cognitive-behavioural anger treatment for people with mild-borderline intellectual disabilities and histories of aggression: A controlled trial. British Journal of Pharmacology. 2005;44(3):367-82. | No | Yes | Yes | Yes | Yes |
| Taylor WJ, Brown M, William L, McPherson KM, Reed K, Dean SG, et al. A pilot cluster randomized controlled trial of structured goal-setting following stroke. Clinical Rehabilitation. 2012;26(4):327-38. | Yes | Yes | Yes | Yes | No |
| Tolou-Shams MP, Stewart AP, Fasciano JBS, Brown LKMD. A Review of HIV Prevention Interventions for Juvenile Offenders. Journal of Pediatric Psychology. 2010;35(3):250-61. | No | Yes | Yes | Yes | No |
| Toroyan T, Roberts I, Oakley A, Laing G, Mugford M, Frost C. Effectiveness of out-of-home day care for disadvantaged families: randomised controlled trial. BMJ. 2003 Oct 16;327(7420):906. | Yes | Yes | Yes | Yes | No |
| Trenkwalder CMD, Kohnen RMD, Allen RPMD, Benes HMD, Ferini-Strambi LMD, Garcia-Borreguero DMD, et al. Clinical trials in restless legs syndrome-Recommendations of the European RLS Study Group (EURLSSG). Movement Disorders. 2007;22(18):S495-S504. | No | No | No | Yes | No |
| Tsai ACMDP, Karasic DHMD, Hammer GPAP, Charlebois EDP, Ragland KP, Moss ARP, et al. Directly Observed Antidepressant Medication Treatment and HIV Outcomes Among Homeless and Marginally Housed HIV-Positive Adults: A Randomized Controlled Trial. American Journal of Public Health. 2013;103(2):308-15. | Yes | No | Yes | Yes | Yes |
| Tsaih P-LMSPT, Shih Y-LPPT, Hu M-HPPT. Low-Intensity Task-Oriented Exercise for Ambulation-Challenged Residents in Long-Term Care Facilities: A Randomized, Controlled Trial. American Journal of Physical Medicine & Rehabilitation. 2012;91(7):616-24. | Yes | Yes | Yes | Yes | No |
| Tsapakis EMBMMM, Soldani FMDSMP, Tondo LMDSM, Baldessarini RJMD. Efficacy of antidepressants in juvenile depression: meta-analysis. British Journal of Psychiatry. 2008;193(1):10-7. | Yes | No | No | Yes | Yes |
| Tucker JA, Reed GM. Evidentiary Pluralism as a Strategy for Research and Evidence-Based Practice in Rehabilitation Psychology. Rehabilitation Psychology. 2008;53(3):279-93. | No | No | No | Yes | Yes |
| Tucker JA, Roth DL. Extending the evidence hierarchy to enhance evidence-based practice for substance use disorders. Addiction. 2006;101(7):918-32. | No | No | No | Yes | Yes |
| Underhill KDJD, Dumont DPMPH, Operario DP. HIV Prevention for Adults With Criminal Justice Involvement: A Systematic Review of HIV Risk-Reduction Interventions in Incarceration and Community Settings. American Journal of Public Health. 2014;104(11):e27-e53. | No | Yes | Yes | Yes | No |
| Valenstein MMD, Eisenberg DPD, McCarthy JFPDMPH, Austin KLMPH, Ganoczy DMPH, Kim HMSD, et al. Service Implications of Providing Intensive Monitoring During High-Risk Periods for Suicide Among VA Patients With Depression. Psychiatric Services. 2009;60(4):439-44. | No | No | No | Yes | Yes |
| Van Craen KRNM, Braes TRNP, Wellens NSLPP, Denhaerynck KRNP, Flamaing JMDP, Moons PRNP, et al. The Effectiveness of Inpatient Geriatric Evaluation and Management Units: A Systematic Review and Meta-Analysis. Journal of the American Geriatrics Society. 2010;58(1):83-92. | No | Yes | Yes | Yes | No |
| Van den Branden S, Van den Broucke S, Leroy R, Declerck D, Bogaerts K, Hoppenbrouwers K. Effect evaluation of an oral health promotion intervention in preschool children. European Journal of Public Health. 2014;24(6):892-7. | No | Yes | Yes | Yes | No |
| van der Meer EWC, Boot CRL, Twisk JWR, Coenraads PJ, Jungbauer FHW, van der Gulden JWJ, et al. Hands4U: the effectiveness of a multifaceted implementation strategy on behaviour related to the prevention of hand eczema-a randomised controlled trial among healthcare workers. Occupational & Environmental Medicine. 2014;71(7):492-9. | Yes | Yes | Yes | Yes | No |
| Van der Molen MJ, Van Luit JEH, Van der Molen MW, Klugkist I, Jongmans MJ. Effectiveness of a computerised working memory training in adolescents with mild to borderline intellectual disabilities. Journal of Intellectual Disability Research. 2010;54(5):433-47. | Yes | Yes | No | Yes | Yes |
| Van Houtven CHP, Thorpe JMP, Chestnutt DMSNRN, Molloy MMSNRN, Boling JCMA, Davis LLPRNF. Do Nurse-Led Skill Training Interventions Affect Informal Caregivers' Out-of-Pocket Expenditures? Gerontologist. 2013;53(1):60-70. | Yes | Yes | No | Yes | Yes |
| van Sluijs EMFP, van Poppel MNMP, Twisk JWRP, Chin A Paw MJP, Calfas KJP, van Mechelen WMDP. Effect of a Tailored Physical Activity Intervention Delivered in General Practice Settings: Results of a Randomized Controlled Trial. American Journal of Public Health PRISONS AND HEALTH. 2005;95(10):1825-31. | Yes | Yes | Yes | Yes | No |
| van Straten A, Hill J, Richards DA, Cuijpers P. Stepped care treatment delivery for depression: a systematic review and meta-analysis. Psychological Medicine. 2015;45(2):231-46. | Yes | Yes | No | Yes | Yes |
| van Vliet PM, Lincoln NB, Foxall A. Comparison of Bobath based and movement science based treatment for stroke: a randomised controlled trial. Journal of Neurology, Neurosurgery & Psychiatry. 2005;76(4):503-8. | Yes | Yes | Yes | Yes | No |
| Vasarainen H, Lokman U, Ruutu M, Taari K, Rannikko A. Prostate cancer active surveillance and health-related quality of life: results of the Finnish arm of the prospective trial. BJU International. 2012;109(11):1614-9. | No | No | No | Yes | No |
| Vazir S, Engle P, Balakrishna N, Griffiths PL, Johnson SL, Creed-Kanashiro H, et al. Cluster-randomized trial on complementary and responsive feeding education to caregivers found improved dietary intake, growth and development among rural Indian toddlers. Maternal and Child Nutrition. 2013;9(1):99-117. | Yes | Yes | Yes | Yes | No |
| Velligan DI, Draper M, Stutes D, Maples N, Mintz J, Tai S, et al. Multimodal Cognitive Therapy: Combining Treatments That Bypass Cognitive Deficits and Deal With Reasoning and Appraisal Biases. Schizophrenia Bulletin. 2009;35(5):884-93. | Yes | Yes | No | Yes | Yes |
| Wagner EF, Tubman JG, Gil AG. Implementing school-based substance abuse interventions: methodological dilemmas and recommended solutions. Addiction Supplement. 2004;99 Supplement(2):106-19. | No | Yes | Yes | Yes | Yes |
| Wake Mp, Lycett Kra, Clifford SAro, Sabin MApe, Gunn Jp, Gibbons Kd, et al. Shared care obesity management in 3-10 year old children: 12 month outcomes of HopSCOTCH randomised trial. BMJ June. 2013;15(346). | Yes | Yes | Yes | Yes | No |
| Wald HLMDM, Glasheen JJMD, Guerrasio JMD, Youngwerth JMMD, Cumbler EUMD. Evaluation of a hospitalist-run acute care for the elderly service. Journal Of Hospital Medicine July/August. 2011;6(6):313-21. | Yes | Yes | Yes | Yes | No |
| Watkins KEMDM, Hunter SBP, Hepner KAP, Paddock SMP, de la Cruz EBA, Zhou AJMS, et al. An Effectiveness Trial of Group Cognitive Behavioral Therapy for Patients With Persistent Depressive Symptoms in Substance Abuse Treatment. Archives of General Psychiatry. 2011;68(6):577-84. | No | Yes | Yes | Yes | Yes |
| Watson C, Burley MC, Purdon C. Verbal repetition in the reappraisal of contamination-related thoughts. Behavioural & Cognitive Psychotherapy. 2010 May;38(3):337-53. PubMed PMID: 20380778. | Yes | Yes | No | Yes | Yes |
| Webb CA, DeRubeis RJ, Amsterdam JD, Shelton RC, Hollon SD, Dimidjian S. Two Aspects of the Therapeutic Alliance: Differential Relations With Depressive Symptom Change. Journal of Consulting & Clinical Psychology. 2011;79(3):279-83. | Yes | Yes | No | Yes | Yes |
| Webb DR, Khunti K, Gray LJ, Srinivasan BT, Farooqi A, Wareham N, et al. Intensive multifactorial intervention improves modelled coronary heart disease risk in screen-detected Type 2 diabetes mellitus: a cluster randomized controlled trial. Diabetic Med. 2012;29(4):531-40. | Yes | Yes | Yes | Yes | No |
| Weinmann S, Koesters M, Becker T. Effects of implementation of psychiatric guidelines on provider performance and patient outcome: systematic review. Acta Psychiatrica Scandinavica. 2007;115(6):420-33. | Yes | Yes | No | Yes | Yes |
| Welch GP, Allen NAPANP, Zagarins SEP, Stamp KDPANP, Bursell S-EP, Kedziora RJMBA. Comprehensive Diabetes Management Program for Poorly Controlled Hispanic Type 2 Patients at a Community Health Center. Diabetes Educator September/October. 2011;37(5):680-8. | Yes | Yes | Yes | Yes | No |
| West RR, Jones DA, Henderson AH. Rehabilitation after myocardial infarction trial (RAMIT): multi-centre randomised controlled trial of comprehensive cardiac rehabilitation in patients following acute myocardial infarction. Heart. 2012;98(8):637-44. | Yes | Yes | No | Yes | No |
| Westen D, Novotny CM, Thompson-Brenner H. The Empirical Status of Empirically Supported Psychotherapies: Assumptions, Findings, and Reporting in Controlled Clinical Trials. Psychological Bulletin. 2004;130(4):631-63. | No | Yes | No | Yes | Yes |
| Whittal ML, Robichaud M, Thordarson DS, McLean PD. Group and individual treatment of obsessive-compulsive disorder using cognitive therapy and exposure plus response prevention: A 2-year follow-up of two randomized trials. Journal of Consulting and Clinical Psychology. 2008 Dec;76(6):1003-14. PubMed PMID: 2008-16943-001. | Yes | Yes | No | Yes | Yes |
| Wilhelm SP, Steketee GP, Reilly-Harrington NAP, Deckersbach TP, Buhlmann UP, Baer LP. Effectiveness of Cognitive Therapy for Obsessive-Compulsive Disorder: An Open Trial. Journal of Cognitive Psychotherapy Summer. 2005;19(2):173-9. | No | Yes | No | Yes | Yes |
| Williams AD, Grisham JR. Cognitive Bias Modification (CBM) of obsessive compulsive beliefs. BMC Psychiatry. 2013 Oct;13:256. PubMed PMID: 2013-37375-001. | Yes | Yes | No | Yes | Yes |
| Wilson TG, Wilfley DE, Agras SW, Bryson SW. Allegiance Bias and Therapist Effects: Results of a Randomized Controlled Trial of Binge Eating Disorder. Clinical Psychology: Science & Practice. 2011;18(2):119-25. | Yes | Yes | No | Yes | Yes |
| Wolfenden L, Wyse R, Campbell E, Brennan L, Campbell KJ, Fletcher A, et al. Randomized controlled trial of a telephone-based intervention for child fruit and vegetable intake: long-term follow-up1-3. The American Journal of Clinical Nutrition. 2014;99(3):543-50. | Yes | Yes | Yes | Yes | No |
| Wu F, Zhang K-l, Shan G-l. An HIV/AIDS intervention programme with Buddhist aid in Yunnan Province. Chinese Medical Journal (English Edition). 2010;123(8):1011-6. | Yes | Yes | Yes | Yes | No |
| Wu Za, Rotheram-Borus MJb, Detels Rb, Li Lb, Guan Jd, Liang Gc, et al. Selecting at-risk populations for sexually transmitted disease/HIV intervention studies. AIDS. 2007;21 Suppl(8):S81-S7. | Yes | Yes | Yes | Yes | No |
| Xie X-JP, Titler MGRNPF, Clarke WRP. Accounting for Intraclass Correlations and Controlling for Baseline Differences in a Cluster-Randomised Evidence-Based Practice Intervention Study. Worldviews on Evidence-Based Nursing. 2008;5(2):95-101. | Yes | Yes | No | Yes | No |
| Yang C-Y, Tsai J-C, Huang Y-C, Lin C-C. Effects of a home-based walking program on perceived symptom and mood status in postoperative breast cancer women receiving adjuvant chemotherapy. Journal of Advanced Nursing. 2011 Jan;67(1):158-68. PubMed PMID: 2011-00899-018. | Yes | Yes | No | Yes | Yes |
| Yeung ASMDSD, Jing YPD, Brenneman SKPD, Chang TEMDMPH, Baer LPD, Hebden TPD, et al. CLINICAL OUTCOMES IN MEASUREMENT-BASED TREATMENT (COMET): A TRIAL OF DEPRESSION MONITORING AND FEEDBACK TO PRIMARY CARE PHYSICIANS. Depression and Anxiety. 2012;29(10):865-73. | No | Yes | Yes | Yes | Yes |
| Zang Y, Zhao Y, Yang Q, Pan Y, Li N, Liu T. A randomised trial on pubertal development and health in China. Journal of Clinical Nursing. 2011;20(21-22):3081-91. | Yes | Yes | Yes | Yes | No |
| Zhang JMD, Chen GBM, Lu WBM, Yan XP, Zhu SMS, Dai YBM, et al. Effects of physical exercise on health-related quality of life and blood lipids in perimenopausal women: a randomized placebo-controlled trial. Menopause. 2014;21(12):1269-76. | Yes | Yes | Yes | Yes | No |
| Zimmermann CMDM, Riechelmann RMD, Krzyzanowska MMDMPH, Rodin GMD, Tannock IMDP. Effectiveness of Specialized Palliative Care: A Systematic Review. JAMA. 2008;299(14):1698-709. | Yes | Yes | Yes | Yes | No |
| Zimmermann MB, Connolly K, Bozo M, Bridson J, Rohner F, Grimci L. Iodine supplementation improves cognition in iodine-deficient schoolchildren in Albania: a randomized, controlled, double-blind study1-4. The American Journal of Clinical Nutrition. 2006;83(1):108-14. | Yes | No | Yes | Yes | Yes |
