# Supplementary material for: A scoping review of the problems and solutions associated with contamination in trials of complex interventions in mental health
Source: BMC Med Res Methodol. 2019 Jan 7;19:4. doi: 10.1186/s12874-018-0646-z (PMC6323722; doi:10.1186/s12874-018-0646-z)
Supplement: Supplementary file 3 — List of references with assessment of bias and support for judgement. The file contains a list of references for all studies that were included in the review together with the assessment of risk of bias and the support for judgement. (DOCX 323 kb) [file 12874_2018_646_MOESM3_ESM.docx]

**Additional file 3: List of references with assessment of bias and support for judgement.**

| **Reference** | **Assessment of bias** | **Judgement** | **Support for judgement** |
| --- | --- | --- | --- |
| [1] Aiarzaguena JM, Grandes G, Gaminde I, Salazar A, Sanchez A, Arino J. A randomized controlled clinical trial of a psychosocial and communication intervention carried out by GPs for patients with medically unexplained symptoms. Psychological Medicine. 2007;37:283-94. | Jadad score (0-5; N/A for trial protocols) | 2 | Study was described as randomised and included a description of withdrawals/dropouts. |
|  | Allocation sequence (selection bias) | Unclear | “GPs were assigned randomly to two groups”.  Randomisation method is not described. |
|  | Allocation concealment (selection bias) | Unclear | Not enough information to make judgement. |
|  | Order of randomisation and consent (selection bias) | Low risk | Recruitment (including consent) took place prior to treatment allocation. |
|  | Order of randomisation and baseline measures (selection bias) | Low risk | The trial flow chart (Figure 1) indicates that baseline assessments took place before randomisation. |
|  | Similarity of baseline outcome measurements across trial arms (selection bias) | Low risk | Summary of baseline outcome measurements (Table 1) shows little difference between trial arms. |
|  | Similarity of baseline characteristics across trial arms (selection bias) | Low risk | Summary of baseline characteristics (Table 1) shows little difference between trial arms. |
|  | Blinding of participants and personnel (performance bias) | High risk | Trial was unblinded. |
|  | Blinded outcome assessment (assessor bias) | Low risk | “Patients completed the SF-36 questionnaire at home, assisted by a trained interviewer who remained blind to the group status throughout the study period.”  Outcome assessment was blind. |
|  | Incomplete outcome data (attrition bias) | Low risk | Amount of missing data was low and balanced across trial arms. |
|  | Similarity in attrition across trial arms (attrition bias) | Low risk | Similar attrition (6% and 8%) across both trial arms. |
| [2] Albers L, Heinen F, Landgraf M, Straube A, Blum B, Filippopulos F, et al. Headache cessation by an educational intervention in grammar schools: a cluster randomized trial. European Journal of Neurology. 2015;22:270-e22. | Jadad score (0-5; N/A for trial protocols) | 2 | Study was described as randomised and included a description of withdrawals/dropouts. |
|  | Allocation sequence (selection bias) | Unclear | Not enough information to make judgement. |
|  | Allocation concealment (selection bias) | Unclear | Not enough information to make judgement. |
|  | Order of randomisation and consent (selection bias) | Low risk | Figure 1 indicates that consent was obtained before randomisation. |
|  | Order of randomisation and baseline measures (selection bias) | Unclear | Not enough information to make judgement. |
|  | Similarity of baseline outcome measurements across trial arms (selection bias) | Unclear | Not enough information to make judgement. |
|  | Similarity of baseline characteristics across trial arms (selection bias) | Low risk | Summary of baseline outcome measurements (Table 1) shows little difference between trial arms. |
|  | Blinding of participants and personnel (performance bias) | High risk | Trial was unblinded. |
|  | Blinded outcome assessment (assessor bias) | N/A | No outcome assessors (participants completed questionnaires). |
|  | Incomplete outcome data (attrition bias) | High risk | Amount of missing data was enough to induce bias in intervention effect estimate. |
|  | Similarity in attrition across trial arms (attrition bias) | Low risk | Similar attrition (35% and 36%) across trial arms. |
| [3] Alessi CA, Martin JL, Webber AP, Kim CE, Harker JO, Josephson KR. Randomized, Controlled Trial of a Nonpharmacological Intervention to Improve Abnormal Sleep/Wake Patterns in Nursing Home Residents. Journal of the American Geriatrics Society. 2005;53:803-10. | Jadad score (0-5; N/A for trial protocols) | 2 | Study was described as randomised and included a description of withdrawals/dropouts. |
|  | Allocation sequence (selection bias) | Unclear | Not enough information to make judgement. |
|  | Allocation concealment (selection bias) | Unclear | Not enough information to make judgement. |
|  | Order of randomisation and consent (selection bias) | Low risk | Figure 1 indicates that consent was obtained before randomisation. |
|  | Order of randomisation and baseline measures (selection bias) | Low risk | “After baseline assessment, participants were randomly allocated to intervention or control groups”  Randomisation occurred after baseline measurements. |
|  | Similarity of baseline outcome measurements across trial arms (selection bias) | Low risk | Summary of baseline outcome measurements (Table 1) shows little difference between trial arms. |
|  | Similarity of baseline characteristics across trial arms (selection bias) | Low risk | Summary of baseline characteristics (Table 1) shows little difference between trial arms. |
|  | Blinding of participants and personnel (performance bias) | High risk | Trial was unblinded. |
|  | Blinded outcome assessment (assessor bias) | High risk | “Research staff who performed outcome assessments could not be adequately blinded to study condition at follow-up because the characteristics of the intervention were directly observable”  Outcome assessment was not blind. |
|  | Incomplete outcome data (attrition bias) | Low risk | Amount of missing data was low and balanced across trial arms. |
|  | Similarity in attrition across trial arms (attrition bias) | Low risk | Similar attrition (<11%) across both trial arms. |
| [4] Alexopoulos GS, Reynolds CF, Bruce ML, Katz IR, Raue PJ, Mulsant BH, et al. Reducing Suicidal Ideation and Depression in Older Primary Care Patients: 24-Month Outcomes of the PROSPECT Study. American Journal of Psychiatry. 2009;166:882-90. | Jadad score (0-5; N/A for trial protocols) | 2 | Study was described as randomised and included a description of withdrawals/dropouts. |
|  | Allocation sequence (selection bias) | Unclear | Not enough information to make judgement. |
|  | Allocation concealment (selection bias) | Unclear | Not enough information to make judgement. |
|  | Order of randomisation and consent (selection bias) | High risk | Clusters were randomised before participants were recruited. |
|  | Order of randomisation and baseline measures (selection bias) | High risk | Clusters were randomised before participants were recruited. |
|  | Similarity of baseline outcome measurements across trial arms (selection bias) | High risk | Some baseline differences in outcome between trial arms (Table 1) |
|  | Similarity of baseline characteristics across trial arms (selection bias) | Low risk | Summary of baseline characteristics (reported elsewhere) shows little difference between trial arms. |
|  | Blinding of participants and personnel (performance bias) | High risk | Trial was unblinded. |
|  | Blinded outcome assessment (assessor bias) | High risk | “Randomization by practice prevented blinded assessment.”  Outcome assessment was not blind. |
|  | Incomplete outcome data (attrition bias) | High risk | Amount of missing data was enough to induce bias in intervention effect estimate. |
|  | Similarity in attrition across trial arms (attrition bias) | High risk | Small difference in follow-up between trial arms (43% and 37%) |
| [5] Anderson BK, Larimer ME. Problem Drinking and the Workplace: An Individualized Approach to Prevention. Psychology of Addictive Behaviors. 2002;16:243-51. | Jadad score (0-5; N/A for trial protocols) | 1 | Study was described as randomised. |
|  | Allocation sequence (selection bias) | Unclear | Not enough information to make judgement. |
|  | Allocation concealment (selection bias) | Unclear | Not enough information to make judgement. |
|  | Order of randomisation and consent (selection bias) | Unclear | Not enough information to make judgement. |
|  | Order of randomisation and baseline measures (selection bias) | High risk | “Employees assigned to either the intervention group (n=82) or the no-treatment control group (n=73) were mailed an assessment packet and asked to respond…”  Baseline outcome measurements took place after randomisation. |
|  | Similarity of baseline outcome measurements across trial arms (selection bias) | High risk | Some differences in outcomes between trial arms (Table 2) |
|  | Similarity of baseline characteristics across trial arms (selection bias) | Low risk | “No significant differences between the intervention and no-treatment control groups in terms of age, gender, or ethnicity.”  No evidence of differences in baseline characteristics between trial arms (also Table 1). |
|  | Blinding of participants and personnel (performance bias) | High risk | Trial was unblinded. |
|  | Blinded outcome assessment (assessor bias) | N/A | No outcome assessors (participants completed questionnaires). |
|  | Incomplete outcome data (attrition bias) | High risk | Amount of missing data was enough to induce bias in intervention effect estimate. |
|  | Similarity in attrition across trial arms (attrition bias) | High risk | Difference in follow-up between trial arms (27% and 18%) |
| [6] Araya R, Flynn T, Rojas G, Fritsch R, Simon G. Cost-Effectiveness of a Primary Care Treatment Program for Depression in Low-Income Women in Santiago, Chile. American Journal of Psychiatry. 2006;163:1379-87. | Jadad score (0-5; N/A for trial protocols) | 2 | Study was described as randomised and included a description of withdrawals/dropouts. |
|  | Allocation sequence (selection bias) | Low risk | “Random assignment… using computer-generated random numbers”.  Allocation sequence was random. |
|  | Allocation concealment (selection bias) | Low risk | “Individuals recruiting patients were neither involved nor aware of the procedure used in generating the allocations.”  Allocation was concealed. |
|  | Order of randomisation and consent (selection bias) | Low risk | The trial flow chart (Figure 1) indicates that consent took place before randomisation. |
|  | Order of randomisation and baseline measures (selection bias) | Low risk | The trial flow chart (Figure 1) indicates that baseline assessments took place before randomisation. |
|  | Similarity of baseline outcome measurements across trial arms (selection bias) | Low risk | Baseline outcomes were similar between trial arms (Table 1). |
|  | Similarity of baseline characteristics across trial arms (selection bias) | Low risk | Baseline characteristics were similar between trial arms (Table 1). |
|  | Blinding of participants and personnel (performance bias) | High risk | Trial was unblinded. |
|  | Blinded outcome assessment (assessor bias) | Low risk | “The primary outcome measure … was administered by independent clinicians blind to treatment assignments.”  Outcome assessments were blind. |
|  | Incomplete outcome data (attrition bias) | High risk | Amount of missing data was enough to induce bias in intervention effect estimate. |
|  | Similarity in attrition across trial arms (attrition bias) | Low risk | No difference in follow-up between trial arms (20% and 21%) |
| [7] Arean PA, Gum A, McCulloch CE, Bostrom A, Gallagher-Thompson D, Thompson L. Treatment of Depression in Low-Income Older Adults. Psychology & Aging. 2005;20:601-9. | Jadad score (0-5; N/A for trial protocols) | 2 | Study was described as randomised and included a description of withdrawals/dropouts. |
|  | Allocation sequence (selection bias) | Unclear | Not enough information to make judgement. |
|  | Allocation concealment (selection bias) | Unclear | Not enough information to make judgement. |
|  | Order of randomisation and consent (selection bias) | Low risk | The trial flow chart (Figure 1) indicates that consent took place before randomisation. |
|  | Order of randomisation and baseline measures (selection bias) | Unclear | Not enough information to make judgement. |
|  | Similarity of baseline outcome measurements across trial arms (selection bias) | Low risk | Baseline outcomes were similar between trial arms (Table 2). |
|  | Similarity of baseline characteristics across trial arms (selection bias) | Unclear | Not enough information to make judgement. |
|  | Blinding of participants and personnel (performance bias) | High risk | Trial was unblinded. |
|  | Blinded outcome assessment (assessor bias) | Low risk | “Trained research assistants… administered all interviews… They did not know participants’ treatment condition.”  Outcome assessment was blind. |
|  | Incomplete outcome data (attrition bias) | High risk | Amount of missing data was enough to induce bias in intervention effect estimate. |
|  | Similarity in attrition across trial arms (attrition bias) | Low risk | Similar numbers of participants (8 for each trial arm) were lost over follow-up. |
| [8] Atlantis E, Chow C-M, Kirby A, Fiatarone Singh MA. Worksite Intervention Effects on Sleep Quality: A Randomized Controlled Trial. Journal of Occupational Health Psychology. 2006;11:291-304. | Jadad score (0-5; N/A for trial protocols) | 3 | Study was described as randomised, with appropriate utilisation of random allocation, and included a description of withdrawals/dropouts. |
|  | Allocation sequence (selection bias) | Low risk | Randomisation used “computer-generated permuted blocks”. |
|  | Allocation concealment (selection bias) | Unclear | Not enough information to make judgement. |
|  | Order of randomisation and consent (selection bias) | Low risk | “Written information consent… was obtained from each subject before study entry.”  Consent preceded randomisation, |
|  | Order of randomisation and baseline measures (selection bias) | Low risk | Baseline outcomes were needed for randomisation, therefore were recorded before it took place. |
|  | Similarity of baseline outcome measurements across trial arms (selection bias) | Low risk | No evidence of trial arm differences in baseline outcomes (Table 1) |
|  | Similarity of baseline characteristics across trial arms (selection bias) | Low risk | No evidence of trial arm differences in baseline characteristics (Table 1) |
|  | Blinding of participants and personnel (performance bias) | High risk | Trial was unblinded. |
|  | Blinded outcome assessment (assessor bias) | N/A | No outcome assessors (participants completed questionnaires). |
|  | Incomplete outcome data (attrition bias) | High risk | Amount of missing data was enough to induce bias in intervention effect estimate. |
|  | Similarity in attrition across trial arms (attrition bias) | High risk | Difference in proportion of participants lost to follow-up between trial arms (44% and 35%) |
| [9] Aune T, Stiles TC. Universal-Based Prevention of Syndromal and Subsyndromal Social Anxiety: A Randomized Controlled Study. Journal of Consulting & Clinical Psychology. 2009;77:867-79. | Jadad score (0-5; N/A for trial protocols) | 1 | Study was described as randomised. |
|  | Allocation sequence (selection bias) | Unclear | Not enough information to make judgement. |
|  | Allocation concealment (selection bias) | Unclear | Not enough information to make judgement. |
|  | Order of randomisation and consent (selection bias) | High risk | Randomisation preceded consent (Figure 1) |
|  | Order of randomisation and baseline measures (selection bias) | High risk | Randomisation preceded baseline measurement (Figure 1) |
|  | Similarity of baseline outcome measurements across trial arms (selection bias) | High risk | Evidence of differences in means between trial arms for some outcomes (Table 2). |
|  | Similarity of baseline characteristics across trial arms (selection bias) | Low risk | No evidence of trial arm differences in baseline characteristics (Table 1) |
|  | Blinding of participants and personnel (performance bias) | High risk | Trial was unblinded. |
|  | Blinded outcome assessment (assessor bias) | N/A | No outcome assessors (participants completed questionnaires). |
|  | Incomplete outcome data (attrition bias) | High risk | Amount of missing data was enough to induce bias in intervention effect estimate. |
|  | Similarity in attrition across trial arms (attrition bias) | High risk | Difference in proportion of participants lost to follow-up between trial arms (28% and 38%) |
| [10] Aveyard P, Brown K, Saunders C, Alexander A, Johnstone E, Munafo MR, et al. Weekly versus basic smoking cessation support in primary care: a randomised controlled trial. Thorax. 2007;62:898-903. | Jadad score (0-5; N/A for trial protocols) | 2 | Study was described as randomised and included a description of withdrawals/dropouts. |
|  | Allocation sequence (selection bias) | Unclear | Not enough information to make judgement. |
|  | Allocation concealment (selection bias) | Unclear | Used sealed numbered envelopes, which were generated by a statistician. Unclear if they were opaque.  Not enough information to make judgement. |
|  | Order of randomisation and consent (selection bias) | Low risk | “Nurses opened the [sealed numbered] envelopes in sequence following eligibility assessment and consent.”  Consent preceded randomisation. |
|  | Order of randomisation and baseline measures (selection bias) | Unclear | Not enough information to make judgement. |
|  | Similarity of baseline outcome measurements across trial arms (selection bias) | Low risk | No evidence of trial arm differences in outcomes at baseline (Table 1) |
|  | Similarity of baseline characteristics across trial arms (selection bias) | Low risk | No evidence of trial arm differences in baseline characteristics (Table 1) |
|  | Blinding of participants and personnel (performance bias) | High risk | Trial was unblinded. |
|  | Blinded outcome assessment (assessor bias) | Low risk | “Research staff making follow-up telephone calls at 3, 6 and 12 months were [blind].”  Outcome assessment was blind. |
|  | Incomplete outcome data (attrition bias) | High risk | Amount of missing data was enough to induce bias in intervention effect estimate. |
|  | Similarity in attrition across trial arms (attrition bias) | Low risk | Difference in proportion of participants lost to follow-up between trial arms (26% and 22%) |
| [11] Baillargeon L, Landreville P, Verreault R, Beauchemin J-P, Gregoire J-P, Morin CM. Discontinuation of benzodiazepines among older insomniac adults treated with cognitive-behavioural therapy combined with gradual tapering: a randomized trial. CMAJ Canadian Medical Association Journal. 2003;169:1015-20. | Jadad score (0-5; N/A for trial protocols) | 3 | Study was described as randomised, with appropriate utilisation of random allocation, and included a description of withdrawals/dropouts. |
|  | Allocation sequence (selection bias) | Low risk | Used table of random numbers. |
|  | Allocation concealment (selection bias) | Unclear | Not enough information to make judgement. |
|  | Order of randomisation and consent (selection bias) | Low risk | Consent took place after randomisation. |
|  | Order of randomisation and baseline measures (selection bias) | Low risk | Baseline outcomes were measured before randomisation. |
|  | Similarity of baseline outcome measurements across trial arms (selection bias) | Low risk | No evidence of trial arm differences in baseline outcome measurements (Table 1) |
|  | Similarity of baseline characteristics across trial arms (selection bias) | Low risk | No evidence of trial arm differences in baseline characteristics (Table 1) |
|  | Blinding of participants and personnel (performance bias) | High risk | Trial was unblinded. |
|  | Blinded outcome assessment (assessor bias) | N/A | Primary outcome was a blood drug concentration measured by machine, therefore no outcome assessor. |
|  | Incomplete outcome data (attrition bias) | Low risk | Amount of missing data was low and balanced across trial arms. |
|  | Similarity in attrition across trial arms (attrition bias) | Low risk | Difference in number of participants lost to follow-up was small (one and two people) |
| [12] Baker-Henningham H, Scott S, Jones K, Walker S. Reducing child conduct problems and promoting social skills in a middle-income country: cluster randomised controlled trial +. British Journal of Psychiatry. 2012;201:101-8. | Jadad score (0-5; N/A for trial protocols) | 3 | Study was described as randomised, with appropriate utilisation of random allocation, and included a description of withdrawals/dropouts. |
|  | Allocation sequence (selection bias) | Low risk | Used computer-generated randomisation sequence. |
|  | Allocation concealment (selection bias) | Low risk | The person who designed the randomisation system was masked to school [unit of randomisation] identity. |
|  | Order of randomisation and consent (selection bias) | Unclear | Not enough information to make judgement. |
|  | Order of randomisation and baseline measures (selection bias) | High risk | Randomisation preceded baseline measurements (Figure 1) |
|  | Similarity of baseline outcome measurements across trial arms (selection bias) | Low risk | No evidence of trial arm differences in outcomes at baseline (Table 2) |
|  | Similarity of baseline characteristics across trial arms (selection bias) | Low risk | No evidence of trial arm differences in baseline characteristics (Table 1) |
|  | Blinding of participants and personnel (performance bias) | High risk | Trial was unblinded. |
|  | Blinded outcome assessment (assessor bias) | Low risk | “Assessments were conducted by trained researchers masked to… group allocation.”  Outcome assessment was blind. |
|  | Incomplete outcome data (attrition bias) | High risk | Used inappropriate simple imputation (replacing missing data post intervention with baseline data) |
|  | Similarity in attrition across trial arms (attrition bias) | Low risk | Difference in proportions of participants lost to follow-up was small (7% and 6%) |
| [13] Ball SA, Martino S, Nich C, Frankforter TL, Van Horn D, Crits-Christoph P, et al. Site Matters: Multisite Randomized Trial of Motivational Enhancement Therapy in Community Drug Abuse Clinics. Journal of Consulting & Clinical Psychology. 2007;75:556-67. | Jadad score (0-5; N/A for trial protocols) | 2 | Study was described as randomised and included a description of withdrawals/dropouts. |
|  | Allocation sequence (selection bias) | Low risk | Used a computerised program. |
|  | Allocation concealment (selection bias) | Low risk | Used central allocation. |
|  | Order of randomisation and consent (selection bias) | Low risk | Randomisation followed consent (Figure 1) |
|  | Order of randomisation and baseline measures (selection bias) | Low risk | Randomisation followed baseline assessment (Figure 1) |
|  | Similarity of baseline outcome measurements across trial arms (selection bias) | Low risk | No evidence of trial arm differences in outcomes at baseline (Table 2) |
|  | Similarity of baseline characteristics across trial arms (selection bias) | Unclear | Not enough information to make judgement. |
|  | Blinding of participants and personnel (performance bias) | High risk | Trial was unblinded. |
|  | Blinded outcome assessment (assessor bias) | Unclear | Not enough information to make judgement. |
|  | Incomplete outcome data (attrition bias) | High risk | Amount of missing data was enough to induce bias in intervention effect estimate. |
|  | Similarity in attrition across trial arms (attrition bias) | Low risk | Difference in proportions of participants lost to follow-up was small (32% and 31%) |
| [14] Bannink R, Broeren S, Heydelberg J, van 't Klooster E, van Baar C, Raat H. Your Health, an intervention at senior vocational schools to promote adolescents' health and health behaviors. Health Education Research. 2014;29:773-85. | Jadad score (0-5; N/A for trial protocols) | 2 | Study was described as randomised and included a description of withdrawals/dropouts. |
|  | Allocation sequence (selection bias) | Low risk | Used computer-generated list of random numbers. |
|  | Allocation concealment (selection bias) | Unclear | Not enough information to make judgement. |
|  | Order of randomisation and consent (selection bias) | Unclear | Not enough information to make judgement. |
|  | Order of randomisation and baseline measures (selection bias) | Unclear | Not enough information to make judgement. |
|  | Similarity of baseline outcome measurements across trial arms (selection bias) | High risk | Evidence of a difference in baseline outcome measurements between trial arms (Table 1). |
|  | Similarity of baseline characteristics across trial arms (selection bias) | High risk | Evidence of a difference in baseline characteristics between trial arms (Table 1). |
|  | Blinding of participants and personnel (performance bias) | High risk | Trial was unblinded. |
|  | Blinded outcome assessment (assessor bias) | N/A | No outcome assessors (participants completed questionnaires). |
|  | Incomplete outcome data (attrition bias) | Low risk | Amount of missing data was low and balanced across trial arms. |
|  | Similarity in attrition across trial arms (attrition bias) | Low risk | Difference in proportions of participants lost to follow-up was small (1% and 1%) |
| [15] Barkhof E, Meijer CJ, de Sonneville LMJ, Linszen DH, de Haan L. The Effect of Motivational Interviewing on Medication Adherence and Hospitalization Rates in Nonadherent Patients with Multi-Episode Schizophrenia. Schizophrenia Bulletin. 2013;39:1242-51. | Jadad score (0-5; N/A for trial protocols) | 3 | Study was described as randomised, with appropriate utilisation of random allocation, and included a description of withdrawals/dropouts. |
|  | Allocation sequence (selection bias) | Low risk | Used computerised randomisation program |
|  | Allocation concealment (selection bias) | Low risk | Allocations were “one by one revealed by the coordinating researcher”.  Allocations were concealed. |
|  | Order of randomisation and consent (selection bias) | Low risk | Randomisation followed consent (Figure 1) |
|  | Order of randomisation and baseline measures (selection bias) | Unclear | Not enough information to make judgement. |
|  | Similarity of baseline outcome measurements across trial arms (selection bias) | Low risk | No evidence of a difference in baseline outcome measurements between trial arms (Table 1). |
|  | Similarity of baseline characteristics across trial arms (selection bias) | Low risk | No evidence of a difference in baseline characteristics between trial arms (Table 1). |
|  | Blinding of participants and personnel (performance bias) | High risk | Trial was unblinded. |
|  | Blinded outcome assessment (assessor bias) | Low risk | “All assessments were performed by trained psychologists and psychiatrists, who were masked to which condition a patient was allocated”.  Outcome assessment was blind. |
|  | Incomplete outcome data (attrition bias) | High risk | Performed per-protocol analysis instead of intention to treat because “the majority of patients who dropped out of the intervention refused follow-up assessments or were lost to follow-up.”  Potential for bias in this analysis approach. |
|  | Similarity in attrition across trial arms (attrition bias) | Low risk | Difference in proportions of participants lost to follow-up was small (18% and 14%) |
| [16] Barsky AJ, Ahern DK. Cognitive Behavior Therapy for Hypochondriasis: A Randomized Controlled Trial. JAMA. 2004;291:1464-70. | Jadad score (0-5; N/A for trial protocols) | 2 | Study was described as randomised and included a description of withdrawals/dropouts. |
|  | Allocation sequence (selection bias) | Low risk | Used random numbers table. |
|  | Allocation concealment (selection bias) | Low risk | Physicians were randomized… by a staff member not connected to the research.” “Study therapists and investigators had no foreknowledge of treatment assignment.”  Allocation was concealed. |
|  | Order of randomisation and consent (selection bias) | Low risk | “Physicians were randomised immediately following the baseline research interview.”  Randomisation followed baseline measures. |
|  | Order of randomisation and baseline measures (selection bias) | Unclear | Not enough information to make judgement. |
|  | Similarity of baseline outcome measurements across trial arms (selection bias) | Low risk | No evidence of a difference in baseline outcome measurements between trial arms (Table 2). |
|  | Similarity of baseline characteristics across trial arms (selection bias) | Low risk | No evidence of a difference in baseline characteristics between trial arms (Table 1). |
|  | Blinding of participants and personnel (performance bias) | High risk | Trial was unblinded. |
|  | Blinded outcome assessment (assessor bias) | Low risk | “All research data were collected by research assistants who were blind to the patient’s treatment status.”  Outcome assessment was blind. |
|  | Incomplete outcome data (attrition bias) | Low risk | Amount of missing data was low and balanced across trial arms. |
|  | Similarity in attrition across trial arms (attrition bias) | Low risk | Similar attrition (10% and 8%) across both trial arms. |
| [17] Barton MB, Morley DS, Moore S, Allen JD, Kleinman KP, Emmons KM, et al. Decreasing Women's Anxieties After Abnormal Mammograms: A Controlled Trial. Journal of the National Cancer Institute. 2004;96:529-38. | Jadad score (0-5; N/A for trial protocols) | 0 | Study was described as randomised but method of randomisation is inappropriate. |
|  | Allocation sequence (selection bias) | High risk | Each participant was “randomly allocated to [intervention] according to the fate of her mammogram appointment”.  Not random allocation. |
|  | Allocation concealment (selection bias) | High risk | Each participant was “randomly allocated to [intervention] according to the fate of her mammogram appointment”.  Allocation not concealed. |
|  | Order of randomisation and consent (selection bias) | Low risk | “Women who agreed to participate and who provided written informed consent were enrolled in the study.”  Allocation followed consent. |
|  | Order of randomisation and baseline measures (selection bias) | Unclear | Not enough information to make judgement. |
|  | Similarity of baseline outcome measurements across trial arms (selection bias) | Unclear | Not enough information to make judgement. |
|  | Similarity of baseline characteristics across trial arms (selection bias) | Low risk | Little evidence of a difference in baseline characteristics between trial arms (Table 1). |
|  | Blinding of participants and personnel (performance bias) | High risk | Trial was unblinded. |
|  | Blinded outcome assessment (assessor bias) | Low risk | “Interviewers were blinded to intervention group assignment.”  Outcome assessment was blind. |
|  | Incomplete outcome data (attrition bias) | High risk | Amount of missing data was enough to induce bias in intervention effect estimate. |
|  | Similarity in attrition across trial arms (attrition bias) | Low risk | Similar proportions of attrition (16%, 15%, 18% and 15%) across trial arms. |
| [18] Bazargan-Hejazi S, Bing E, Bazargan M, Der-Martirosian C, Hardin E, Bernstein J, et al. Evaluation of a Brief Intervention in an Inner-City Emergency Department. Annals of Emergency Medicine. 2005;46:67-76. | Jadad score (0-5; N/A for trial protocols) | 0 | Study was described as randomised but method of randomisation is inappropriate. |
|  | Allocation sequence (selection bias) | High risk | “Each of the 3 health promotion advocates performed random allocation for their own enrollees, assigning the first participant by a flip of a coin, and alternating status thereafter.”  Allocation was not random. |
|  | Allocation concealment (selection bias) | High risk | “Each of the 3 health promotion advocates performed random allocation for their own enrollees, assigning the first participant by a flip of a coin, and alternating status thereafter.”  Allocation was not concealed. |
|  | Order of randomisation and consent (selection bias) | Low risk | Allocation followed consent (Figure). |
|  | Order of randomisation and baseline measures (selection bias) | Low risk | “After the baseline assessment, enrollees were assigned to either the intervention or the control group.”  Allocation followed baseline assessment. |
|  | Similarity of baseline outcome measurements across trial arms (selection bias) | High risk | Evidence of a difference in baseline outcome measurement (Table 1). |
|  | Similarity of baseline characteristics across trial arms (selection bias) | Low risk | No evidence of a difference in baseline characteristics between trial arms (Table 1). |
|  | Blinding of participants and personnel (performance bias) | High risk | Trial was unblinded. |
|  | Blinded outcome assessment (assessor bias) | Low risk | “To guard against interviewer bias…, enrolees were not followed up by the same health promotion advocate who assessed them initially. Patients were notified not to reveal their group assignments to any project staff at any time.”  Outcome assessment was blind. |
|  | Incomplete outcome data (attrition bias) | High risk | Amount of missing data was enough to induce bias in intervention effect estimate. |
|  | Similarity in attrition across trial arms (attrition bias) | Low risk | Similar proportion of attrition (36% and 39%) across both trial arms. |
| [19] Beach SRH, Kogan SM, Brody GH, Chen Y-F, Lei M-K, Murry VM. Change in Caregiver Depression as a Function of the Strong African American Families Program. Journal of Family Psychology. 2008;22:241-52. | Jadad score (0-5; N/A for trial protocols) | 2 | Study was described as randomised and included a description of withdrawals/dropouts. |
|  | Allocation sequence (selection bias) | Unclear | Not enough information to make judgement. |
|  | Allocation concealment (selection bias) | Unclear | Not enough information to make judgement. |
|  | Order of randomisation and consent (selection bias) | High risk | Randomisation preceded consent (Figure 1) |
|  | Order of randomisation and baseline measures (selection bias) | High risk | Randomisation preceded baseline assessment (Figure 1) |
|  | Similarity of baseline outcome measurements across trial arms (selection bias) | Unclear | Not enough information to make judgement. |
|  | Similarity of baseline characteristics across trial arms (selection bias) | High risk | Some evidence of a difference in baseline characteristics between trial arms (Table 1). |
|  | Blinding of participants and personnel (performance bias) | High risk | Trial was unblinded. |
|  | Blinded outcome assessment (assessor bias) | Low risk | “Field interviewers were blind to the families’ group assignments.”  Assessment was blind. |
|  | Incomplete outcome data (attrition bias) | Low risk | Amount of missing data was low and balanced across trial arms. |
|  | Similarity in attrition across trial arms (attrition bias) | Low risk | Similar proportion of attrition (3% and 7%) across both trial arms. |
| [20] Beaver K, Campbell M, Williamson S, Procter D, Sheridan J, Heath J, et al. An exploratory randomized controlled trial comparing telephone and hospital follow-up after treatment for colorectal cancer. Colorectal Disease. 2012;14:1201-9. | Jadad score (0-5; N/A for trial protocols) | 3 | Study was described as randomised, with appropriate utilisation of random allocation, and included a description of withdrawals/dropouts. |
|  | Allocation sequence (selection bias) | Low risk | Allocation used computerised system. |
|  | Allocation concealment (selection bias) | Low risk | “Allocation sequences were concealed until interventions were assigned”.  Allocation was concealed. |
|  | Order of randomisation and consent (selection bias) | Low risk | Allocation followed consent (Figure 1). |
|  | Order of randomisation and baseline measures (selection bias) | Unclear | Not enough information to make judgement. |
|  | Similarity of baseline outcome measurements across trial arms (selection bias) | Low risk | No evidence of a difference in baseline outcome measurement between trial arms (Table 2). |
|  | Similarity of baseline characteristics across trial arms (selection bias) | Low risk | No evidence of a difference in baseline characteristics between trial arms (Table 1). |
|  | Blinding of participants and personnel (performance bias) | High risk | Trial was unblinded. |
|  | Blinded outcome assessment (assessor bias) | N/A | No outcome assessors (participants completed questionnaires). |
|  | Incomplete outcome data (attrition bias) | High risk | Amount of missing data was enough to induce bias in intervention effect estimate. |
|  | Similarity in attrition across trial arms (attrition bias) | Low risk | Similar number of participants lost to follow-up across both trial arms (8 and 7 participants). |
| [21] Beck CK, Vogelpohl TS, Rasin JH, Uriri JT, O'Sullivan P, Walls R, et al. Effects of Behavioral Interventions on Disruptive Behavior and Affect in Demented Nursing Home Residents. Nursing Research July/August. 2002;51:219-28. | Jadad score (0-5; N/A for trial protocols) | 1 | Study was described as randomised. |
|  | Allocation sequence (selection bias) | Unclear | Not enough information to make judgement. |
|  | Allocation concealment (selection bias) | Unclear | Not enough information to make judgement. |
|  | Order of randomisation and consent (selection bias) | Low risk | Allocation followed consent and screening (only who “passed these screens were randomized to one of the five groups”). |
|  | Order of randomisation and baseline measures (selection bias) | High risk | “During the 12-week intervention period, the first three weeks were considered baseline and the last two weeks postintervention.”  Randomisation preceded baseline outcome measurements. |
|  | Similarity of baseline outcome measurements across trial arms (selection bias) | High risk | Evidence of a difference in baseline outcome measurement between trial arms (Table 3). |
|  | Similarity of baseline characteristics across trial arms (selection bias) | Low risk | “No statistically significant demographic differences emerged among the five groups”.  No evidence of a difference in baseline characteristics between trial arms. |
|  | Blinding of participants and personnel (performance bias) | High risk | Trial was unblinded. |
|  | Blinded outcome assessment (assessor bias) | Low risk | Research assistants who recorded the outcome “were blinded to... participants’ group assignments.”  Outcome assessment was blind. |
|  | Incomplete outcome data (attrition bias) | High risk | Amount of missing data was enough to induce bias in intervention effect estimate. |
|  | Similarity in attrition across trial arms (attrition bias) | Unclear | Not enough information to make judgement. |
| [22] Beckie TM, Beckstead JW. Predicting Cardiac Rehabilitation Attendance in a Gender-Tailored Randomized Clinical Trial. Journal of Cardiopulmonary Rehabilitation & Prevention May/June. 2010;30:147-56. | Jadad score (0-5; N/A for trial protocols) | 2 | Study was described as randomised and included a description of withdrawals/dropouts. |
|  | Allocation sequence (selection bias) | Low risk | Used coin tossing. |
|  | Allocation concealment (selection bias) | Unclear | Treatment assignment sheets “were placed in opaque envelopes, sealed, and delivered to the project director.” Not clear if envelopes were sequentially numbered.  Not enough information to make judgement. |
|  | Order of randomisation and consent (selection bias) | Low risk | “After consent and before randomization…”  Randomisation followed consent. |
|  | Order of randomisation and baseline measures (selection bias) | Low risk | “After consent and before randomization, the research assistant conducted the comprehensive evaluation, collected baseline psychosocial and physiological data…”  Randomisation followed baseline outcome measurements. |
|  | Similarity of baseline outcome measurements across trial arms (selection bias) | Low risk | “The groups did not differ in baseline psychosocial variables” (also shown in Table 1).  No evidence of a difference in baseline outcome measurement between trial arms. |
|  | Similarity of baseline characteristics across trial arms (selection bias) | Low risk | “The groups were not different at baseline on marital status, education, previous CR attendance, work status, race, risk factors or co-morbidities”  No evidence of a difference in baseline characteristics between trial arms. |
|  | Blinding of participants and personnel (performance bias) | High risk | Trial was unblinded. |
|  | Blinded outcome assessment (assessor bias) | Unclear | Not enough information to make judgement. |
|  | Incomplete outcome data (attrition bias) | Low risk | Amount of missing data was low and balanced across trial arms. |
|  | Similarity in attrition across trial arms (attrition bias) | Low risk | No evidence of loss to follow-up (Figure 1) |
| [23] Becona E, Vazquez FL. Effectiveness of Personalized Written Feedback Through a Mail Intervention for Smoking Cessation: A Randomized-Controlled Trial in Spanish Smokers. Journal of Consulting & Clinical Psychology. 2001;69:33-40. | Jadad score (0-5; N/A for trial protocols) | 1 | Study was described as randomised. |
|  | Allocation sequence (selection bias) | Unclear | Not enough information to make judgement. |
|  | Allocation concealment (selection bias) | Unclear | Not enough information to make judgement. |
|  | Order of randomisation and consent (selection bias) | Low risk | Potential participants signed a consent form in order to participate in the study.  Randomisation followed consent. |
|  | Order of randomisation and baseline measures (selection bias) | Unclear | Not enough information to make judgement. |
|  | Similarity of baseline outcome measurements across trial arms (selection bias) | Low risk | No evidence of a difference in baseline outcome measurements between trial arms (Table 1). |
|  | Similarity of baseline characteristics across trial arms (selection bias) | Low risk | No evidence of a difference in baseline characteristics between trial arms (Table 1). |
|  | Blinding of participants and personnel (performance bias) | High risk | Trial was unblinded. |
|  | Blinded outcome assessment (assessor bias) | Low risk | “End-of-treatment and three follow-up interviews (at 3, 6, and 12 months) were conducted by trained interviewers who had not been informed of the participant's treatment history as part of one of the three groups.”  Outcome assessment was blind. |
|  | Incomplete outcome data (attrition bias) | Low risk | Amount of missing data was low and balanced across trial arms. |
|  | Similarity in attrition across trial arms (attrition bias) | Low risk | No loss to follow-up at 6 months and little at 12 months. |
| [24] Befort CA, Nollen N, Ellerbeck EF, Sullivan DK, Thomas JL, Ahluwalia JS. Motivational interviewing fails to improve outcomes of a behavioral weight loss program for obese African American women: a pilot randomized trial. Journal of Behavioral Medicine. 2008;31:367-77. | Jadad score (0-5; N/A for trial protocols) | 2 | Study was described as randomised and included a description of withdrawals/dropouts. |
|  | Allocation sequence (selection bias) | Unclear | Not enough information to make judgement. |
|  | Allocation concealment (selection bias) | Low risk | Used closed envelope procedure that was done by study statistician and was “concealed from the investigators and data collection staff”. |
|  | Order of randomisation and consent (selection bias) | Low risk | Randomisation followed consent (Figure 1). |
|  | Order of randomisation and baseline measures (selection bias) | Unclear | Not enough information to make judgement. |
|  | Similarity of baseline outcome measurements across trial arms (selection bias) | Low risk | No evidence of a difference in baseline outcome measurements between trial arms (Table 1). |
|  | Similarity of baseline characteristics across trial arms (selection bias) | Low risk | No evidence of a difference in baseline characteristics between trial arms (Table 1). |
|  | Blinding of participants and personnel (performance bias) | High risk | Trial was unblinded. |
|  | Blinded outcome assessment (assessor bias) | Low risk | Treatment allocation was “concealed from… data collection staff”.  Outcome assessment was blind. |
|  | Incomplete outcome data (attrition bias) | High risk | Amount of missing data was enough to induce bias in intervention effect estimate. |
|  | Similarity in attrition across trial arms (attrition bias) | Low risk | Similar numbers of participants lost to follow-up across both trial arms (6 and 4 participants). |
| [25] Bellantonio S, Kenny AM, Fortinsky RH, Kleppinger A, Robison J, Gruman C, et al. Efficacy of a Geriatrics Team Intervention for Residents in Dementia-Specific Assisted Living Facilities: Effect on Unanticipated Transitions. Journal of the American Geriatrics Society. 2008;56:523-8. | Jadad score (0-5; N/A for trial protocols) | 2 | Study was described as randomised and included a description of withdrawals/dropouts. |
|  | Allocation sequence (selection bias) | Unclear | Not enough information to make judgement. |
|  | Allocation concealment (selection bias) | Unclear | Used sealed envelopes to conceal allocation. Not clear if they were sequentially numbered and opaque.  Not enough information to make judgement. |
|  | Order of randomisation and consent (selection bias) | Low risk | Consent was obtained at time of admission, i.e. randomisation followed consent. |
|  | Order of randomisation and baseline measures (selection bias) | Unclear | Not enough information to make judgement. |
|  | Similarity of baseline outcome measurements across trial arms (selection bias) | N/A | Outcomes (hospital transitions, visits and nursing home relocations) were not measured at baseline. |
|  | Similarity of baseline characteristics across trial arms (selection bias) | Low risk | No evidence of a difference in baseline characteristics between trial arms (Table 1). |
|  | Blinding of participants and personnel (performance bias) | High risk | Trial was unblinded. |
|  | Blinded outcome assessment (assessor bias) | Unclear | Not enough information to make judgement. |
|  | Incomplete outcome data (attrition bias) | Unclear | Not enough information to make judgement. |
|  | Similarity in attrition across trial arms (attrition bias) | Unclear | Not enough information to make judgement. |
| [26] Bermejo I, Schneider F, Kriston L, Gaebel W, Hegerl U, Berger M, et al. Improving outpatient care of depression by implementing practice guidelines: a controlled clinical trial. International Journal for Quality in Health Care. 2009;21:29-36. | Jadad score (0-5; N/A for trial protocols) | 0 | Study was not randomised. |
|  | Allocation sequence (selection bias) | High risk | Allocation was not random. |
|  | Allocation concealment (selection bias) | High risk | No allocation concealment. |
|  | Order of randomisation and consent (selection bias) | High risk | Allocation preceded participant enrolment/consent (treatment was allocated to large regions) |
|  | Order of randomisation and baseline measures (selection bias) | High risk | Allocation preceded baseline measurements (Figure 1) |
|  | Similarity of baseline outcome measurements across trial arms (selection bias) | Unclear | Not enough information to make judgement. |
|  | Similarity of baseline characteristics across trial arms (selection bias) | High risk | Evidence of a difference in baseline characteristics between trial arms (Table 1). |
|  | Blinding of participants and personnel (performance bias) | High risk | Trial was unblinded. |
|  | Blinded outcome assessment (assessor bias) | Unclear | Not enough information to make judgement. |
|  | Incomplete outcome data (attrition bias) | Unclear | Not enough information to make judgement. |
|  | Similarity in attrition across trial arms (attrition bias) | Unclear | Not enough information to make judgement. |
| [27] Bernstein GA, Layne AE, Egan EA, Tennison DM. School-Based Interventions for Anxious Children. Journal of the American Academy of Child & Adolescent Psychiatry. 2005;44:1118-27. | Jadad score (0-5; N/A for trial protocols) | 1 | Study was described as randomised. |
|  | Allocation sequence (selection bias) | Unclear | Not enough information to make judgement. |
|  | Allocation concealment (selection bias) | Unclear | Not enough information to make judgement. |
|  | Order of randomisation and consent (selection bias) | Low risk | Randomisation followed consent (Figure 1) |
|  | Order of randomisation and baseline measures (selection bias) | Unclear | Not enough information to make judgement. |
|  | Similarity of baseline outcome measurements across trial arms (selection bias) | Low risk | “Analysis further indicated that groups were comparable at baseline on [outcomes].”  No evidence of a difference in baseline outcomes between trial arms. |
|  | Similarity of baseline characteristics across trial arms (selection bias) | Low risk | “Analysis indicated that groups were balanced on demographic variables.”  No evidence of a difference in baseline characteristics between trial arms |
|  | Blinding of participants and personnel (performance bias) | High risk | Trial was unblinded. |
|  | Blinded outcome assessment (assessor bias) | Low risk | “Participants were randomly assigned to independent evaluators at baseline and again posttreatment. All independent evaluators conducted interviews with children from each of the three schools. Families were instructed not to mention their condition assignment to the independent evaluators.”  Outcome assessment was blind. |
|  | Incomplete outcome data (attrition bias) | Low risk | Amount of missing data was low and balanced across trial arms. |
|  | Similarity in attrition across trial arms (attrition bias) | Low risk | Similar numbers of participants lost to follow-up across trial arms (2, 2 and 0 participants). |
| [28] Bernstein SL, Bijur P, Cooperman N, Jearld S, Arnsten JH, Moadel A, et al. A Randomized Trial of a Multicomponent Cessation Strategy for Emergency Department Smokers. Academic Emergency Medicine. 2011;18:575-83. | Jadad score (0-5; N/A for trial protocols) | 3 | Study was described as randomised, with appropriate utilisation of random allocation, and included a description of withdrawals/dropouts. |
|  | Allocation sequence (selection bias) | Low risk | Used computerised random number generator. |
|  | Allocation concealment (selection bias) | Low risk | Used “opaque consecutively numbered envelopes with the treatment group indicated inside the envelope.”  Allocation was concealed. |
|  | Order of randomisation and consent (selection bias) | Low risk | Randomisation followed consent (Figure 1). |
|  | Order of randomisation and baseline measures (selection bias) | Unclear | Not enough information to make judgement. |
|  | Similarity of baseline outcome measurements across trial arms (selection bias) | Low risk | No evidence of a difference in baseline outcome measurements between trial arms (Table 1). |
|  | Similarity of baseline characteristics across trial arms (selection bias) | Low risk | No evidence of a difference in baseline characteristics between trial arms (Table 1). |
|  | Blinding of participants and personnel (performance bias) | High risk | Trial was unblinded. |
|  | Blinded outcome assessment (assessor bias) | Low risk | “Blinded follow-up was performed at 3 months.”  Outcome assessment was blind. |
|  | Incomplete outcome data (attrition bias) | High risk | Amount of missing data was enough to induce bias in intervention effect estimate. |
|  | Similarity in attrition across trial arms (attrition bias) | Low risk | Similar proportions of participants lost to follow-up across both trial arms (15% and 18%). |
| [29] Bombardier CH, Bell KR, Temkin NR, Fann JR, Hoffman J, Dikmen S. The Efficacy of a Scheduled Telephone Intervention for Ameliorating Depressive Symptoms During the First Year After Traumatic Brain Injury. Journal of Head Trauma Rehabilitation July/August. 2009;24:230-8. | Jadad score (0-5; N/A for trial protocols) | 3 | Study was described as randomised, with appropriate utilisation of random allocation, and included a description of withdrawals/dropouts. |
|  | Allocation sequence (selection bias) | Low risk | Used computerised random number generator. |
|  | Allocation concealment (selection bias) | Low risk | “Assignment was concealed from the study coordinator until he or she telephoned to randomize the patient.”  Allocation was concealed. |
|  | Order of randomisation and consent (selection bias) | Low risk | Randomisation followed consent (Figure 1). |
|  | Order of randomisation and baseline measures (selection bias) | Unclear | Not enough information to make judgement. |
|  | Similarity of baseline outcome measurements across trial arms (selection bias) | High risk | Evidence of a difference in baseline outcome measurements between trial arms (Table 1). |
|  | Similarity of baseline characteristics across trial arms (selection bias) | Low risk | No evidence of a difference in baseline characteristics between trial arms (Table 1). |
|  | Blinding of participants and personnel (performance bias) | High risk | Trial was unblinded. |
|  | Blinded outcome assessment (assessor bias) | Low risk | “The neurobehavioral examiner conducting the follow-up (outcome) interview and testing was not aware of the group to which the participant was assigned. Participants were instructed prior to outcome testing not to reveal assignment to the outcomes examiner.”  Outcome assessment was blind. |
|  | Incomplete outcome data (attrition bias) | Low risk | Amount of missing data was low and balanced across trial arms. |
|  | Similarity in attrition across trial arms (attrition bias) | Low risk | Similar proportions of participants lost to follow-up across both trial arms (5% and 9%). |
| [30] Borland R, Balmford J, Benda P. Population-level effects of automated smoking cessation help programs: a randomized controlled trial. Addiction. 2013;108:618-28. | Jadad score (0-5; N/A for trial protocols) | 3 | Study was described as randomised, with appropriate utilisation of random allocation, and included a description of withdrawals/dropouts. |
|  | Allocation sequence (selection bias) | Low risk | Used random number generator. |
|  | Allocation concealment (selection bias) | Unclear | Not enough information to make judgement. |
|  | Order of randomisation and consent (selection bias) | Low risk | Randomisation followed consent (Figure 1). |
|  | Order of randomisation and baseline measures (selection bias) | Low risk | “Randomization was via a random number generator embedded within the baseline survey.”  Randomisation followed baseline measures. |
|  | Similarity of baseline outcome measurements across trial arms (selection bias) | Unclear | Not enough information to make judgement. |
|  | Similarity of baseline characteristics across trial arms (selection bias) | Unclear | Not enough information to make judgement. |
|  | Blinding of participants and personnel (performance bias) | High risk | Trial was unblinded. |
|  | Blinded outcome assessment (assessor bias) | N/A | No outcome assessors (participants completed questionnaires). |
|  | Incomplete outcome data (attrition bias) | High risk | Amount of missing data was enough to induce bias in intervention effect estimate. |
|  | Similarity in attrition across trial arms (attrition bias) | Low risk | Similar proportions of participants lost to follow-up across trial arms (16%, 12%, 13%, 15% and 13%). |
| [31] Bormann JE, Gifford AL, Shively M, Smith TL, Redwine L, Kelly A, et al. Effects of Spiritual Mantram Repetition on HIV Outcomes: A Randomized Controlled Trial. Journal of Behavioral Medicine. 2006;29:359-76. | Jadad score (0-5; N/A for trial protocols) | 3 | Study was described as randomised, with appropriate utilisation of random allocation, and included a description of withdrawals/dropouts. |
|  | Allocation sequence (selection bias) | Low risk | Used table of random numbers. |
|  | Allocation concealment (selection bias) | Unclear | Not enough information to make judgement. |
|  | Order of randomisation and consent (selection bias) | Low risk | Randomisation followed consent (Figure 2). |
|  | Order of randomisation and baseline measures (selection bias) | Low risk | “Ninety-three (83%) completed baseline and were randomly assigned to the mantram group (n = 46) or control group (n = 47).”  Randomisation followed baseline measures. |
|  | Similarity of baseline outcome measurements across trial arms (selection bias) | Unclear | Not enough information to make judgement. |
|  | Similarity of baseline characteristics across trial arms (selection bias) | Low risk | “…demographic… variables at baseline were not significantly different between groups.”  No evidence of a difference in baseline characteristics between trial arms. |
|  | Blinding of participants and personnel (performance bias) | High risk | Trial was unblinded. |
|  | Blinded outcome assessment (assessor bias) | N/A | No outcome assessors (participants completed questionnaires). |
|  | Incomplete outcome data (attrition bias) | High risk | Amount of missing data was enough to induce bias in intervention effect estimate. |
|  | Similarity in attrition across trial arms (attrition bias) | Low risk | Similar proportions of participants lost to follow-up across trial arms (30% and 28%). |
| [32] Bosmans JE, Brook OH, van Hout HPJ, de Bruijne MC, Nieuwenhuyse H, Bouter LM, et al. Cost Effectiveness of a Pharmacy-Based Coaching Programme to Improve Adherence to Antidepressants. Pharmacoeconomics. 2007;25:25-37. | Jadad score (0-5; N/A for trial protocols) | 1 | Study was described as randomised. |
|  | Allocation sequence (selection bias) | Unclear | Not enough information to make judgement. |
|  | Allocation concealment (selection bias) | Unclear | Randomisation forms were “precoded and delivered in sealed envelopes” (earlier publication). Not clear if envelopes were opaque and if they were sequentially numbered.  Not enough information to make judgement. |
|  | Order of randomisation and consent (selection bias) | Low risk | “After giving written informed consent, patients were randomly allocated.”  Randomisation followed consent. |
|  | Order of randomisation and baseline measures (selection bias) | High risk | Randomisation preceded baseline measures (Figure 1). |
|  | Similarity of baseline outcome measurements across trial arms (selection bias) | Low risk | No evidence of a difference in baseline outcome measurements between trial arms (Table 1). |
|  | Similarity of baseline characteristics across trial arms (selection bias) | Low risk | No evidence of a difference in baseline characteristics between trial arms (Table 1). |
|  | Blinding of participants and personnel (performance bias) | High risk | Trial was unblinded. |
|  | Blinded outcome assessment (assessor bias) | Unclear | Not enough information to make judgement. |
|  | Incomplete outcome data (attrition bias) | High risk | Amount of missing data was enough to induce bias in intervention effect estimate. |
|  | Similarity in attrition across trial arms (attrition bias) | Low risk | Similar proportions of participants lost to follow-up across trial arms (43 and 41%). |
| [33] Bricker JB, Bush T, Zbikowski S, Mercer LD, Heffner JL. Randomized Trial of Telephone-Delivered Acceptance and Commitment Therapy Versus Cognitive Behavioral Therapy for Smoking Cessation: A Pilot Study. Nicotine & Tobacco Research. 2014;16:1446-54. | Jadad score (0-5; N/A for trial protocols) | 3 | Study was described as randomised, with appropriate utilisation of random allocation, and included a description of withdrawals/dropouts. |
|  | Allocation sequence (selection bias) | Low risk | Used computerised random allocation. |
|  | Allocation concealment (selection bias) | Low risk | “Neither research staff nor participants had access to upcoming randomized study arm assignments.”  Allocation was concealed. |
|  | Order of randomisation and consent (selection bias) | Low risk | Randomisation followed consent (Figure 1). |
|  | Order of randomisation and baseline measures (selection bias) | Low risk | Participants did baseline measures then were contacted two days later for randomisation.  Randomisation followed baseline measures. |
|  | Similarity of baseline outcome measurements across trial arms (selection bias) | Low risk | No evidence of a difference in baseline outcome measurements between trial arms (Table 1). |
|  | Similarity of baseline characteristics across trial arms (selection bias) | Low risk | No evidence of a difference in baseline characteristics between trial arms (Table 1). |
|  | Blinding of participants and personnel (performance bias) | High risk | Trial was unblinded. |
|  | Blinded outcome assessment (assessor bias) | Low risk | “All follow-up survey data collection was conducted by an FHCRC team blind to treatment arm assignment.”  Outcome assessment was blind. |
|  | Incomplete outcome data (attrition bias) | High risk | Amount of missing data was enough to induce bias in intervention effect estimate. |
|  | Similarity in attrition across trial arms (attrition bias) | High risk | Different proportions of participants lost to follow-up across trial arms (27% and 39%) |
| [34] Brouwers EPM, de Bruijne MC, Terluin B, Tiemens BG, Verhaak PFM. Cost-effectiveness of an activating intervention by social workers for patients with minor mental disorders on sick leave: a randomized controlled trial. European Journal of Public Health. 2007;17:214-20. | Jadad score (0-5; N/A for trial protocols) | 3 | Study was described as randomised, with appropriate utilisation of random allocation, and included a description of withdrawals/dropouts. |
|  | Allocation sequence (selection bias) | Low risk | Random allocation used dice. |
|  | Allocation concealment (selection bias) | Unclear | Use numbered envelopes but not clear if opaque and if they were sequentially numbered.  Not enough information to make judgement. |
|  | Order of randomisation and consent (selection bias) | Low risk | Randomisation followed consent (Figure 1). |
|  | Order of randomisation and baseline measures (selection bias) | Unclear | Not enough information to make judgement. |
|  | Similarity of baseline outcome measurements across trial arms (selection bias) | Unclear | Not enough information to make judgement. |
|  | Similarity of baseline characteristics across trial arms (selection bias) | Low risk | “No significant differences were found between the groups with respect to these [demographic] characteristics.”  No evidence of a difference in baseline outcome measurements between trial arms. |
|  | Blinding of participants and personnel (performance bias) | High risk | Trial was unblinded. |
|  | Blinded outcome assessment (assessor bias) | Unclear | Not enough information to make judgement. |
|  | Incomplete outcome data (attrition bias) | Low risk | Amount of missing data was low and balanced across trial arms. |
|  | Similarity in attrition across trial arms (attrition bias) | Low risk | Similar proportions of participants lost to follow-up across both trial arms (3% and 6%). |
| [35] Brown RA, Ramsey SE, Strong DR, Myers MG, Kahler CW, Lejuez CW, et al. Effects of motivational interviewing on smoking cessation in adolescents with psychiatric disorders. Tobacco Control. 2003;12 Supplement:iv3-iv10. | Jadad score (0-5; N/A for trial protocols) | 1 | Study was described as randomised. |
|  | Allocation sequence (selection bias) | Unclear | Not enough information to make judgement. |
|  | Allocation concealment (selection bias) | Unclear | Not enough information to make judgement. |
|  | Order of randomisation and consent (selection bias) | Low risk | “All participants provided written assent for study participation, and written consent was obtained from a parent or legal guardian. A baseline assessment battery was then completed. Participants were assigned, by cohort, to treatment condition”  Randomisation followed consent. |
|  | Order of randomisation and baseline measures (selection bias) | Low risk | “All participants provided written assent for study participation, and written consent was obtained from a parent or legal guardian. A baseline assessment battery was then completed. Participants were assigned, by cohort, to treatment condition”  Randomisation followed baseline measures. |
|  | Similarity of baseline outcome measurements across trial arms (selection bias) | Low risk | “Participants did not differ significantly by treatment condition on [baseline outcomes].”  No evidence of a difference in baseline outcome measurements between trial arms. |
|  | Similarity of baseline characteristics across trial arms (selection bias) | Low risk | Participants did not differ significantly by treatment condition on [baseline characteristics].  No evidence of a difference in baseline characteristics between trial arms. |
|  | Blinding of participants and personnel (performance bias) | High risk | Trial was unblinded. |
|  | Blinded outcome assessment (assessor bias) | N/A | No outcome assessors (participants completed questionnaires). |
|  | Incomplete outcome data (attrition bias) | Low risk | Amount of missing data was low (<10%) and balanced across trial arms. |
|  | Similarity in attrition across trial arms (attrition bias) | Low risk | “Rates of missing data were not significantly different across motivational intervention and brief advice conditions.”  Similar proportions of participants lost to follow-up across both trial arms. |
| [36] Bruce ML, Ten Have TR, Reynolds CF, Katz II, Schulberg HC, Mulsant BH, et al. Reducing Suicidal Ideation and Depressive Symptoms in Depressed Older Primary Care Patients: A Randomized Controlled Trial. JAMA. 2004;291:1081-91. | Jadad score (0-5; N/A for trial protocols) | 3 | Study was described as randomised, with appropriate utilisation of random allocation, and included a description of withdrawals/dropouts. |
|  | Allocation sequence (selection bias) | Low risk | Used coin tossing |
|  | Allocation concealment (selection bias) | Unclear | Not enough information to make judgement |
|  | Order of randomisation and consent (selection bias) | High risk | Randomisation preceded consent (Figure) |
|  | Order of randomisation and baseline measures (selection bias) | High risk | Randomisation preceded baseline measures (Figure) |
|  | Similarity of baseline outcome measurements across trial arms (selection bias) | High risk | Evidence of a difference in baseline outcome measurements between trial arms (Table 1). |
|  | Similarity of baseline characteristics across trial arms (selection bias) | Low risk | No evidence of a difference in baseline characteristics between trial arms (Table 1). |
|  | Blinding of participants and personnel (performance bias) | High risk | Trial was unblinded. |
|  | Blinded outcome assessment (assessor bias) | Unclear | Not enough information to make judgement. |
|  | Incomplete outcome data (attrition bias) | High risk | Amount of missing data was enough to induce bias in intervention effect estimate. |
|  | Similarity in attrition across trial arms (attrition bias) | Low risk | Similar proportion of participants lost to follow-up across trial arms (31% and 31%). |
| [37] Burling TA, Burling AS, Latini D. A Controlled Smoking Cessation Trial for Substance-Dependent Inpatients. Journal of Consulting & Clinical Psychology. 2001;69:295-304. | Jadad score (0-5; N/A for trial protocols) | 2 | Study was described as randomised and included a description of withdrawals/dropouts. |
|  | Allocation sequence (selection bias) | Unclear | Not enough information to make judgement. |
|  | Allocation concealment (selection bias) | Unclear | Not enough information to make judgement. |
|  | Order of randomisation and consent (selection bias) | Low risk | Randomisation occurred within a sample of participants whose eligibility had been screened and agreed to participate.  Randomisation followed consent. |
|  | Order of randomisation and baseline measures (selection bias) | Unclear | Not enough information to make judgement. |
|  | Similarity of baseline outcome measurements across trial arms (selection bias) | Low risk | “There were no significant differences among the participants in the various treatment accepter conditions.”  No evidence of a difference in baseline outcome measurements between trial arms. |
|  | Similarity of baseline characteristics across trial arms (selection bias) | Low risk | “There were no significant differences among the participants in the various treatment accepter conditions.”  No evidence of a difference in baseline characteristics between trial arms. |
|  | Blinding of participants and personnel (performance bias) | High risk | Trial was unblinded. |
|  | Blinded outcome assessment (assessor bias) | Unclear | Not enough information to make judgement. |
|  | Incomplete outcome data (attrition bias) | Low risk | Amount of missing data was low and balanced across trial arms. |
|  | Similarity in attrition across trial arms (attrition bias) | Low risk | Similar proportion of participants lost to follow-up across trial arms (6%, 10% and 8%). |
| [38] Calear AL, Christensen H, Mackinnon A, Griffiths KM, O'Kearney R. The YouthMood Project: A Cluster Randomized Controlled Trial of an Online Cognitive Behavioral Program With Adolescents. Journal of Consulting & Clinical Psychology. 2009;77:1021-32. | Jadad score (0-5; N/A for trial protocols) | 3 | Study was described as randomised, with appropriate utilisation of random allocation, and included a description of withdrawals/dropouts. |
|  | Allocation sequence (selection bias) | Low risk | Used computer-generated random number generator. |
|  | Allocation concealment (selection bias) | Low risk | “An independent statistician randomly allocated schools within each stratum to the intervention or wait-list control condition using a computerized random number generator. The identity of schools was concealed from the statistician during this process.”  Allocation was concealed. |
|  | Order of randomisation and consent (selection bias) | High risk | Randomisation preceded consent (Figure 1). |
|  | Order of randomisation and baseline measures (selection bias) | High risk | Randomisation preceded baseline measures (Figure 1). |
|  | Similarity of baseline outcome measurements across trial arms (selection bias) | Low risk | No evidence of a difference in baseline outcome measurements between trial arms (Table 2). |
|  | Similarity of baseline characteristics across trial arms (selection bias) | High risk | “Significant differences were found in the age… and gender [between the treatment groups].”  Evidence of a difference in baseline characteristics between trial arms. |
|  | Blinding of participants and personnel (performance bias) | High risk | Trial was unblinded. |
|  | Blinded outcome assessment (assessor bias) | N/A | No outcome assessors (participants completed questionnaires). |
|  | Incomplete outcome data (attrition bias) | High risk | Amount of missing data was enough to induce bias in intervention effect estimate. |
|  | Similarity in attrition across trial arms (attrition bias) | Low risk | Similar proportion of participants lost to follow-up across trial arms (57% and 61%). |
| [39] Callahan CM, Boustani MA, Unverzagt FW, Austrom MG, Damush TM, Perkins AJ, et al. Effectiveness of Collaborative Care for Older Adults With Alzheimer Disease in Primary Care: A Randomized Controlled Trial. JAMA. 2006;295:2148-57. | Jadad score (0-5; N/A for trial protocols) | 3 | Study was described as randomised, with appropriate utilisation of random allocation, and included a description of withdrawals/dropouts. |
|  | Allocation sequence (selection bias) | Low risk | Used random numbers table. |
|  | Allocation concealment (selection bias) | Low risk | “Prior to initiating the study, we obtained a list of all primary care physicians at all participating clinics” and then randomised these physicians. “Members of the diagnostic team, the geriatric nurse practitioner, and patients and caregivers were blinded to the physician’s randomization status until the counseling session described above was completed and the patient consented to participate.”  Allocation was concealed. |
|  | Order of randomisation and consent (selection bias) | High risk | Clusters (physicians) were randomised before patients consented.  Randomisation preceded consent. |
|  | Order of randomisation and baseline measures (selection bias) | High risk | Clusters (physicians) were randomised before patients completed baseline assessments.  Randomisation preceded baseline measures. |
|  | Similarity of baseline outcome measurements across trial arms (selection bias) | Low risk | No evidence of a difference in baseline outcome measurements between trial arms (Table 1). |
|  | Similarity of baseline characteristics across trial arms (selection bias) | Low risk | No evidence of a difference in baseline characteristics between trial arms (Table 1). |
|  | Blinding of participants and personnel (performance bias) | High risk | Trial was unblinded. |
|  | Blinded outcome assessment (assessor bias) | Unclear | Not enough information to make judgement. |
|  | Incomplete outcome data (attrition bias) | High risk | Amount of missing data was enough to induce bias in intervention effect estimate. |
|  | Similarity in attrition across trial arms (attrition bias) | High risk | Different proportion of participants lost to follow-up across trial arms (23% and 29%). |
| [40] Campbell-Heider N, Tuttle J, Knapp TR. The Effect of Positive Adolescent Life Skills Training on Long Term Outcomes for High-Risk Teens. Journal of Addictions Nursing. 2009;20:6-15. | Jadad score (0-5; N/A for trial protocols) | 1 | Study was described as randomised. |
|  | Allocation sequence (selection bias) | Unclear | Not enough information to make judgement. |
|  | Allocation concealment (selection bias) | Unclear | Not enough information to make judgement. |
|  | Order of randomisation and consent (selection bias) | Low risk | Eighteen students provided parental and teen consent. These participants were randomly assigned to a treatment condition.  Randomisation followed consent. |
|  | Order of randomisation and baseline measures (selection bias) | Unclear | Not enough information to make judgement. |
|  | Similarity of baseline outcome measurements across trial arms (selection bias) | Low risk | No evidence of a difference in baseline characteristics between trial arms (Table 1). |
|  | Similarity of baseline characteristics across trial arms (selection bias) | Unclear | Not enough information to make judgement. |
|  | Blinding of participants and personnel (performance bias) | High risk | Trial was unblinded. |
|  | Blinded outcome assessment (assessor bias) | Unclear | Not enough information to make judgement. |
|  | Incomplete outcome data (attrition bias) | Unclear | Not enough information to make judgement. |
|  | Similarity in attrition across trial arms (attrition bias) | Unclear | Not enough information to make judgement. |
| [41] Cappella E, Hamre BK, Kim HY, Henry DB, Frazier SL, Atkins MS, et al. Teacher Consultation and Coaching Within Mental Health Practice: Classroom and Child Effects in Urban Elementary Schools. Journal of Consulting & Clinical Psychology. 2012;80:597-610. | Jadad score (0-5; N/A for trial protocols) | 2 | Study was described as randomised, with appropriate utilisation of random allocation. |
|  | Allocation sequence (selection bias) | Low risk | Used random numbers table. |
|  | Allocation concealment (selection bias) | Unclear | Not enough information to make judgement. |
|  | Order of randomisation and consent (selection bias) | Low risk | Before Time 1, consent was obtained. “After Time 1 data were collected, researchers used a random numbers table to randomize teachers within schools to intervention and control conditions.”  Randomisation followed consent. |
|  | Order of randomisation and baseline measures (selection bias) | Low risk | “Time 1 data were collected, researchers used a random numbers table to randomize teachers within schools to intervention and control conditions”  Randomisation followed baseline measures. |
|  | Similarity of baseline outcome measurements across trial arms (selection bias) | Low risk | “No significant differences between groups for any study  variable at T1.”  No evidence of a difference in baseline outcome measurements between trial arms |
|  | Similarity of baseline characteristics across trial arms (selection bias) | Low risk | “No significant differences between groups for any study  variable at T1.”  No evidence of a difference in baseline characteristics between trial arms |
|  | Blinding of participants and personnel (performance bias) | High risk | Trial was unblinded. |
|  | Blinded outcome assessment (assessor bias) | N/A | No outcome assessors (participants completed questionnaires). |
|  | Incomplete outcome data (attrition bias) | Unclear | Not enough information to make judgement. |
|  | Similarity in attrition across trial arms (attrition bias) | Unclear | Not enough information to make judgement. |
| [42] Chan MF, Ng SE, Tien A, Man Ho RC, Thayala J. A randomised controlled study to explore the effect of life story review on depression in older Chinese in Singapore. Health & Social Care in the Community. 2013;21:545-53. | Jadad score (0-5; N/A for trial protocols) | 3 | Study was described as randomised, with appropriate utilisation of random allocation, and included a description of withdrawals/dropouts. |
|  | Allocation sequence (selection bias) | Low risk | Used computerised random number generator. |
|  | Allocation concealment (selection bias) | Unclear | Not enough information to make judgement. |
|  | Order of randomisation and consent (selection bias) | Low risk | “58 potential subjects were contacted, 12 subjects refused to participate… The remaining 26 subjects were randomly allocated into the intervention (*n* = 14) and control groups (*n* = 12).”  Randomisation followed consent. |
|  | Order of randomisation and baseline measures (selection bias) | Low risk | “Twenty were disqualified because 19 subjects had baseline GDS‐15 scores that were below four and one had hearing difficulties. The remaining 26 subjects were randomly allocated into the intervention (*n* = 14) and control groups (*n* = 12).”  Randomisation followed baseline measures. |
|  | Similarity of baseline outcome measurements across trial arms (selection bias) | Low risk | No evidence of a difference in baseline outcome measurements between trial arms (Table 2). |
|  | Similarity of baseline characteristics across trial arms (selection bias) | Low risk | “There were no apparent differences between the two groups at baseline.”  No evidence of a difference in baseline characteristics between trial arms (also, Table 1). |
|  | Blinding of participants and personnel (performance bias) | High risk | Trial was unblinded. |
|  | Blinded outcome assessment (assessor bias) | High risk | “Interviewer knew the allocation groups after randomisation.”  Outcome assessment was not blind. |
|  | Incomplete outcome data (attrition bias) | Low risk | Amount of missing data was low and balanced across trial arms. |
|  | Similarity in attrition across trial arms (attrition bias) | Low risk | Similar proportion of participants lost to follow-up across trial arms (0% and 0%). |
| [43] Chanen AM, Jackson HJ, McCutcheon LK, Jovev M, Dudgeon P, Yuen HP, et al. Early intervention for adolescents with borderline personality disorder using cognitive analytic therapy: randomised controlled trial. British Journal of Psychiatry. 2008;193:477-84. | Jadad score (0-5; N/A for trial protocols) | 3 | Study was described as randomised, with appropriate utilisation of random allocation, and included a description of withdrawals/dropouts. |
|  | Allocation sequence (selection bias) | Low risk | Used computerised randomisation. |
|  | Allocation concealment (selection bias) | Low risk | “The nature of the randomisation procedure was concealed from the therapists by using an on-site, password-protected computer program, operated by an independent ORYGEN administrative staff member who entered all participants’ data”  Allocation was concealed. |
|  | Order of randomisation and consent (selection bias) | Low risk | “Randomisation occurred following informed consent.”  Randomisation followed consent. |
|  | Order of randomisation and baseline measures (selection bias) | High risk | “Randomisation occurred… prior to baseline assessment.”  Randomisation preceded baseline measures. |
|  | Similarity of baseline outcome measurements across trial arms (selection bias) | Low risk | No evidence of a difference in baseline outcome measurements between trial arms (Table 1). |
|  | Similarity of baseline characteristics across trial arms (selection bias) | Low risk | No evidence of a difference in baseline characteristics between trial arms (online Table DS1). |
|  | Blinding of participants and personnel (performance bias) | High risk | Trial was unblinded. |
|  | Blinded outcome assessment (assessor bias) | Low risk | “No ‘unmaskings’ [of outcome assessors] were reported during the conduct of the trial.”  Outcome assessment was blind. |
|  | Incomplete outcome data (attrition bias) | High risk | Amount of missing data was enough to induce bias in intervention effect estimate. |
|  | Similarity in attrition across trial arms (attrition bias) | Low risk | Similar proportion of participants lost to follow-up across trial arms (20% and 21%). |
| [44] Chang M-Y, Chen C-H, Huang K-F. Effects of music therapy on psychological health of women during pregnancy. Journal of Clinical Nursing. 2008;17:2580-7. | Jadad score (0-5; N/A for trial protocols) | 3 | Study was described as randomised, with appropriate utilisation of random allocation, and included a description of withdrawals/dropouts. |
|  | Allocation sequence (selection bias) | Unclear | Not enough information to make judgement. |
|  | Allocation concealment (selection bias) | Unclear | Not enough information to make judgement. |
|  | Order of randomisation and consent (selection bias) | High risk | “After the allocation of participants to groups, the principal researcher explained the study purpose… Informed consent was obtained from all participants.”  Randomisation preceded consent. |
|  | Order of randomisation and baseline measures (selection bias) | High risk | “After the allocation of participants to groups, the principal researcher explained the study purpose and data collection procedure to them.”  Randomisation preceded baseline measures. |
|  | Similarity of baseline outcome measurements across trial arms (selection bias) | Low risk | No evidence of a difference in baseline outcome measurements between trial arms (Table 2). |
|  | Similarity of baseline characteristics across trial arms (selection bias) | Low risk | No evidence of a difference in baseline characteristics between trial arms (Table 1). |
|  | Blinding of participants and personnel (performance bias) | High risk | Trial was unblinded. |
|  | Blinded outcome assessment (assessor bias) | N/A | No outcome assessors (participants completed questionnaires). |
|  | Incomplete outcome data (attrition bias) | Low risk | Amount of missing data was low and balanced across trial arms. |
|  | Similarity in attrition across trial arms (attrition bias) | Low risk | Similar proportion of participants lost to follow-up across trial arms (3% and 1%). |
| [45] Chen Z, Meng Z, Milbury K, Bei W, Zhang Y, Thornton B, et al. Qigong improves quality of life in women undergoing radiotherapy for breast cancer: Results of a randomized controlled trial. Cancer. 2013;119:1690-8. | Jadad score (0-5; N/A for trial protocols) | 1 | Study was described as randomised. |
|  | Allocation sequence (selection bias) | Low risk | Used minimisation. |
|  | Allocation concealment (selection bias) | Unclear | Not enough information to make judgement. |
|  | Order of randomisation and consent (selection bias) | Low risk | “During pretreatment clinic visits, immediately after the treating radiation oncologist had briefly introduced the study to potentially eligible patients, research nurses approached patients, confirmed study eligibility, and obtained written informed consent before data collection [which was before randomisation].”  Randomisation followed consent. |
|  | Order of randomisation and baseline measures (selection bias) | Low risk | “Before… randomization, patients underwent a baseline (T1) assessment.”  Randomisation followed baseline assessment. |
|  | Similarity of baseline outcome measurements across trial arms (selection bias) | Low risk | No evidence of a difference in baseline outcome measurements between trial arms (Table 2). |
|  | Similarity of baseline characteristics across trial arms (selection bias) | Low risk | No evidence of a difference in baseline characteristics between trial arms (Table 1). |
|  | Blinding of participants and personnel (performance bias) | High risk | Trial was unblinded. |
|  | Blinded outcome assessment (assessor bias) | Unclear | Not enough information to make judgement. |
|  | Incomplete outcome data (attrition bias) | High risk | Amount of missing data was low but not balanced across trial arms. |
|  | Similarity in attrition across trial arms (attrition bias) | High risk | Difference in follow-up between trial arms (0% and 10%) |
| [46] Chochinov HM, Kristjanson LJ, Breitbart W, McClement S, Hack TF, Hassard T, et al. Effect of dignity therapy on distress and end-of-life experience in terminally ill patients: a randomised controlled trial. Lancet Oncology. 2011;12:753-62. | Jadad score (0-5; N/A for trial protocols) | 3 | Study was described as randomised, with appropriate utilisation of random allocation, and included a description of withdrawals/dropouts. |
|  | Allocation sequence (selection bias) | Low risk | Used computer-generated table of random numbers. |
|  | Allocation concealment (selection bias) | Unclear | “Blinding was achieved using opaque sealed envelopes, containing allocations that were only opened once consent had been obtained.” Not clear if envelopes were sequentially numbered.  Not enough information to make judgement. |
|  | Order of randomisation and consent (selection bias) | Low risk | “After providing written informed consent, all participants were randomly assigned to one of three study arms.”  Randomisation followed consent. |
|  | Order of randomisation and baseline measures (selection bias) | High risk | “Once patients were randomized to standard palliative care, they were asked to complete a battery of baseline psychometric questionnaires.”  Randomisation preceded baseline measures. |
|  | Similarity of baseline outcome measurements across trial arms (selection bias) | Low risk | No evidence of a difference in baseline outcome measurements between trial arms (Tables 3A and 4A). |
|  | Similarity of baseline characteristics across trial arms (selection bias) | Low risk | No evidence of a difference in baseline characteristics between trial arms (Table 2). |
|  | Blinding of participants and personnel (performance bias) | High risk | Trial was unblinded. |
|  | Blinded outcome assessment (assessor bias) | Unclear | Not enough information to make judgement. |
|  | Incomplete outcome data (attrition bias) | Unclear | Not enough information to make judgement. |
|  | Similarity in attrition across trial arms (attrition bias) | Unclear | Not enough information to make judgement. |
| [47] Chouinard M-C, Robichaud-Ekstrand S. The Effectiveness of a Nursing Inpatient Smoking Cessation Program in Individuals With Cardiovascular Disease. Nursing Research July/August. 2005;54:243-54. | Jadad score (0-5; N/A for trial protocols) | 2 | Study was described as randomised and included a description of withdrawals/dropouts. |
|  | Allocation sequence (selection bias) | Unclear | Not enough information to make judgement. |
|  | Allocation concealment (selection bias) | Unclear | Allocation was inserted “into envelopes that were sealed and would be opened by the investigator only at the time of recruitment”. Not clear if envelopes were opaque and if they were sequentially numbered.  Not enough information to make judgement. |
|  | Order of randomisation and consent (selection bias) | Low risk | Figure 1 indicates that consent was obtained before randomisation. |
|  | Order of randomisation and baseline measures (selection bias) | Unclear | Not enough information to make judgement. |
|  | Similarity of baseline outcome measurements across trial arms (selection bias) | Low risk | No evidence of a difference in baseline outcome measurements between trial arms (Table 3). |
|  | Similarity of baseline characteristics across trial arms (selection bias) | Low risk | No evidence of a difference in baseline characteristics between trial arms (Table 2). |
|  | Blinding of participants and personnel (performance bias) | High risk | Trial was unblinded. |
|  | Blinded outcome assessment (assessor bias) | Unclear | Not enough information to make judgement. |
|  | Incomplete outcome data (attrition bias) | High risk | Amount of missing data was enough to induce bias in intervention effect estimate. |
|  | Similarity in attrition across trial arms (attrition bias) | Low risk | Similar proportion of participants lost to follow-up across trial arms (11%, 18% and 13%). |
| [48] Clarke AM, Bunting B, Barry MM. Evaluating the implementation of a school-based emotional well-being programme: a cluster randomized controlled trial of Zippy's Friends for children in disadvantaged primary schools. Health Education Research. 2014;29:786-98. | Jadad score (0-5; N/A for trial protocols) | 2 | Study was described as randomised and included a description of withdrawals/dropouts. |
|  | Allocation sequence (selection bias) | Unclear | Not enough information to make judgement. |
|  | Allocation concealment (selection bias) | Unclear | Not enough information to make judgement. |
|  | Order of randomisation and consent (selection bias) | Unclear | Not enough information to make judgement. |
|  | Order of randomisation and baseline measures (selection bias) | Unclear | Not enough information to make judgement. |
|  | Similarity of baseline outcome measurements across trial arms (selection bias) | Low risk | No evidence of a difference in baseline outcome measurements between trial arms (Table 4). |
|  | Similarity of baseline characteristics across trial arms (selection bias) | Low risk | No evidence of a difference in baseline characteristics between trial arms (Table 2). |
|  | Blinding of participants and personnel (performance bias) | High risk | Trial was unblinded. |
|  | Blinded outcome assessment (assessor bias) | N/A | No outcome assessors (participants completed questionnaires). |
|  | Incomplete outcome data (attrition bias) | High risk | Amount of missing data was enough to induce bias in intervention effect estimate. |
|  | Similarity in attrition across trial arms (attrition bias) | Low risk | Similar proportion of participants lost to follow-up across trial arms (23%, 20% and 17%). |
| [49] Clarkson JE, Young L, Ramsay CR, Bonner BC, Bonetti D. How to influence patient oral hygiene behavior effectively. Journal of Dental Research. 2009;88:933-7. | Jadad score (0-5; N/A for trial protocols) | 3 | Study was described as randomised, with appropriate utilisation of random allocation, and included a description of withdrawals/dropouts. |
|  | Allocation sequence (selection bias) | Low risk | Used computerised randomisation. |
|  | Allocation concealment (selection bias) | Unclear | Used sealed opaque envelopes (online supplementary materials). Not clear if they were sequentially numbered.  Not enough information to make judgement. |
|  | Order of randomisation and consent (selection bias) | High risk | In the cluster-randomised half of the trial, clusters (dentists) were randomisation before patients were recruited. Therefore, randomisation preceded consent. |
|  | Order of randomisation and baseline measures (selection bias) | High risk | In the cluster-randomised half of the trial, clusters (dentists) were randomisation before patients were recruited. Therefore, randomisation preceded baseline measures. |
|  | Similarity of baseline outcome measurements across trial arms (selection bias) | Low risk | “No significant group difference in any baseline measure.”  No evidence of a difference in baseline outcome measures between trial arms. |
|  | Similarity of baseline characteristics across trial arms (selection bias) | Low risk | “No significant group difference in any baseline measure.”  No evidence of a difference in baseline characteristics between trial arms. |
|  | Blinding of participants and personnel (performance bias) | High risk | Trial was unblinded. |
|  | Blinded outcome assessment (assessor bias) | High risk | Outcome assessors were clinicians who were not blind. Therefore, outcome assessment was not blind. |
|  | Incomplete outcome data (attrition bias) | High risk | Amount of missing data was enough to induce bias in intervention effect estimate. |
|  | Similarity in attrition across trial arms (attrition bias) | High risk | Difference in follow-up between trial arms (19%, 16%, 12% and 31%) |
| [50] Clement S, van Nieuwenhuizen A, Kassam A, Flach C, Lazarus A, de Castro M, et al. Filmed v. live social contact interventions to reduce stigma: randomised controlled trial. British Journal of Psychiatry. 2012;201:57-64. | Jadad score (0-5; N/A for trial protocols) | 2 | Study was described as randomised and included a description of withdrawals/dropouts. |
|  | Allocation sequence (selection bias) | Unclear | Not enough information to make judgement. |
|  | Allocation concealment (selection bias) | Unclear | Used “opaque envelope containing group allocation”. Not clear if they were sequentially numbered and sealed.  Not enough information to make judgement. |
|  | Order of randomisation and consent (selection bias) | Low risk | The trial flow chart (Figure 1) indicates that consent took place before randomisation.  Randomisation followed consent. |
|  | Order of randomisation and baseline measures (selection bias) | Unclear | Not enough information to make judgement. |
|  | Similarity of baseline outcome measurements across trial arms (selection bias) | Low risk | No evidence of a difference in baseline outcome measurements between trial arms (Table 2). |
|  | Similarity of baseline characteristics across trial arms (selection bias) | Low risk | “The groups were found to be balanced for all characteristics listed in Table 1.”  No evidence of a difference in baseline characteristics between trial arms. |
|  | Blinding of participants and personnel (performance bias) | High risk | Trial was unblinded. |
|  | Blinded outcome assessment (assessor bias) | Low risk | “Questionnaire data were entered masked to group allocation.”  Outcome assessment was blind. |
|  | Incomplete outcome data (attrition bias) | High risk | Amount of missing data was enough to induce bias in intervention effect estimate. |
|  | Similarity in attrition across trial arms (attrition bias) | High risk | Difference in follow-up between trial arms (46%, 39% and 53%) |
| [51] Cole MG, McCusker J, Bellavance F, Primeau FJ, Bailey RF, Bonnycastle MJ, et al. Systematic detection and multidisciplinary care of delirium in older medical inpatients: a randomized trial. CMAJ Canadian Medical Association Journal. 2002;167:753-9. | Jadad score (0-5; N/A for trial protocols) | 3 | Study was described as randomised, with appropriate utilisation of random allocation, and included a description of withdrawals/dropouts. |
|  | Allocation sequence (selection bias) | Low risk | Used computer-generated random numbers. |
|  | Allocation concealment (selection bias) | Unclear | “The study statistician prepared two series of sealed envelopes, one for each stratum, containing the treatment allocation.” Not clear if envelopes were opaque and if they were sequentially numbered.  Not enough information to make judgement. |
|  | Order of randomisation and consent (selection bias) | Low risk | The trial flow chart (Figure 1) indicates that consent took place before randomisation. |
|  | Order of randomisation and baseline measures (selection bias) | Unclear | Not enough information to make judgement. |
|  | Similarity of baseline outcome measurements across trial arms (selection bias) | Low risk | No evidence of a difference in baseline outcome measurements between trial arms (Table 2). |
|  | Similarity of baseline characteristics across trial arms (selection bias) | Low risk | No evidence of a difference in baseline characteristics between trial arms (Table 2). |
|  | Blinding of participants and personnel (performance bias) | High risk | Trial was unblinded. |
|  | Blinded outcome assessment (assessor bias) | Low risk | “To check on the extent of blinding of the research assistant  during the study, the assistant was asked to guess which study group a convenience sample of 18 patients (whom the research assistant had recently assessed) belonged to.”  Outcome assessment was blind. |
|  | Incomplete outcome data (attrition bias) | Low risk | Amount of missing data was low and balanced across trial arms. |
|  | Similarity in attrition across trial arms (attrition bias) | Low risk | Similar proportion of participants lost to follow-up across trial arms (2% and 6%). |
| [52] Cole MG, McCusker J, Elie M, Dendukuri N, Latimer E, Belzile E. Systematic detection and multidisciplinary care of depression in older medical inpatients: a randomized trial. CMAJ Canadian Medical Association Journal. 2006;174:38-44. | Jadad score (0-5; N/A for trial protocols) | 2 | Study was described as randomised and included a description of withdrawals/dropouts. |
|  | Allocation sequence (selection bias) | Unclear | Not enough information to make judgement. |
|  | Allocation concealment (selection bias) | Unclear | “The study statistician prepared a series of sealed envelopes containing the treatment allocation.” Not clear if envelopes were opaque and if they were sequentially numbered.  Not enough information to make judgement. |
|  | Order of randomisation and consent (selection bias) | Low risk | The trial flow chart (Figure 1) indicates that consent took place before randomisation. |
|  | Order of randomisation and baseline measures (selection bias) | Unclear | Not enough information to make judgement. |
|  | Similarity of baseline outcome measurements across trial arms (selection bias) | Low risk | No evidence of a difference in baseline outcome measurements between trial arms (Table 1). |
|  | Similarity of baseline characteristics across trial arms (selection bias) | Low risk | No evidence of a difference in baseline characteristics between trial arms (Table 1). |
|  | Blinding of participants and personnel (performance bias) | High risk | Trial was unblinded. |
|  | Blinded outcome assessment (assessor bias) | Low risk | “Research assistants, blind to group allocation, collected data from the patients at enrolment and at 3 and 6 months.”  Outcome assessment was blind. |
|  | Incomplete outcome data (attrition bias) | High risk | Amount of missing data was enough to induce bias in intervention effect estimate. |
|  | Similarity in attrition across trial arms (attrition bias) | Low risk | Similar proportion of participants lost to follow-up across trial arms (61% and 58%). |
| [53] Connors GJ, Walitzer KS, Dermen KH. Preparing Clients for Alcoholism Treatment: Effects on Treatment Participation and Outcomes. Journal of Consulting & Clinical Psychology. 2002;70:1161-9. | Jadad score (0-5; N/A for trial protocols) | 1 | Study was described as randomised. |
|  | Allocation sequence (selection bias) | Unclear | Not enough information to make judgement. |
|  | Allocation concealment (selection bias) | Unclear | Not enough information to make judgement. |
|  | Order of randomisation and consent (selection bias) | Low risk | “Once consent was obtained, the client completed the baseline assessment. Following the baseline assessment, clients were randomly assigned to one of the three preparatory intervention conditions.”  Randomisation followed consent. |
|  | Order of randomisation and baseline measures (selection bias) | Low risk | “Once consent was obtained, the client completed the baseline assessment. Following the baseline assessment, clients were randomly assigned to one of the three preparatory intervention conditions.”  Randomisation followed baseline measures. |
|  | Similarity of baseline outcome measurements across trial arms (selection bias) | Low risk | “One-way analyses of variance (ANOVAs) and chi-square tests revealed no significant differences between treatment conditions on baseline characteristics, including… alcohol abstinent days, heavy drinking days, recent and lifetime drinking consequences, and use of other drugs.”  No evidence of a difference in baseline outcome measurements between trial arms |
|  | Similarity of baseline characteristics across trial arms (selection bias) | Low risk | “One-way analyses of variance (ANOVAs) and chi-square tests revealed no significant differences between treatment conditions on baseline characteristics, including age, race, employment status, years of education, household income…”  No evidence of a difference in baseline characteristics between trial arms |
|  | Blinding of participants and personnel (performance bias) | High risk | Trial was unblinded. |
|  | Blinded outcome assessment (assessor bias) | Unclear | Not enough information to make judgement. |
|  | Incomplete outcome data (attrition bias) | Unclear | Not enough information to make judgement. |
|  | Similarity in attrition across trial arms (attrition bias) | Unclear | Not enough information to make judgement. |
| [54] Cook S, Chambers E, Coleman JH. Occupational therapy for people with psychotic conditions in community settings: a pilot randomized controlled trial. Clinical Rehabilitation. 2009;23:40-52. | Jadad score (0-5; N/A for trial protocols) | 3 | Study was described as randomised, with appropriate utilisation of random allocation, and included a description of withdrawals/dropouts. |
|  | Allocation sequence (selection bias) | Low risk | Used computerised randomisation. |
|  | Allocation concealment (selection bias) | Low risk | Used central allocation and allocated treatment by telephoning treatment teams directly.  Allocation was concealed. |
|  | Order of randomisation and consent (selection bias) | Low risk | “Those consenting were randomized…”  Randomisation followed consent. |
|  | Order of randomisation and baseline measures (selection bias) | Unclear | Not enough information to make judgement. |
|  | Similarity of baseline outcome measurements across trial arms (selection bias) | Low risk | No evidence of a difference in baseline outcome measurements between trial arms (Table 2). |
|  | Similarity of baseline characteristics across trial arms (selection bias) | Low risk | No evidence of a difference in baseline characteristics between trial arms (Table 1). |
|  | Blinding of participants and personnel (performance bias) | High risk | Trial was unblinded. |
|  | Blinded outcome assessment (assessor bias) | Low risk | “The two assessors from the research team were blind to the allocation.”  Outcome assessment was blind. |
|  | Incomplete outcome data (attrition bias) | Low risk | Amount of missing data was low and balanced across trial arms. |
|  | Similarity in attrition across trial arms (attrition bias) | Low risk | Similar numbers of participants (0 and 2 for the trial arms) were lost over follow-up. |
| [55] Cooper LA, Ghods Dinoso BK, Ford DE, Roter DL, Primm AB, Larson SM, et al. Comparative Effectiveness of Standard versus Patient-Centered Collaborative Care Interventions for Depression among African Americans in Primary Care Settings: The BRIDGE Study. Health Services Research. 2013;48:150-74. | Jadad score (0-5; N/A for trial protocols) | 3 | Study was described as randomised, with appropriate utilisation of random allocation, and included a description of withdrawals/dropouts. |
|  | Allocation sequence (selection bias) | Low risk | Used computerised randomisation. See <https://doi.org/10.1186/1748-5908-5-18>. |
|  | Allocation concealment (selection bias) | Low risk | Allocation of clusters (clinicians) was done by computer and “patient recruiters were blinded to the clinicians' randomization assignment during recruitment, and the patients did not know their assigned treatment status until after enrolment.” See <https://doi.org/10.1186/1748-5908-5-18>.  Allocation was concealed. |
|  | Order of randomisation and consent (selection bias) | High risk | Clusters (clinicians) were randomised before patients were recruited.  Randomisation preceded consent. |
|  | Order of randomisation and baseline measures (selection bias) | High risk | Clusters (clinicians) were randomised before patients were recruited.  Randomisation preceded baseline measures. |
|  | Similarity of baseline outcome measurements across trial arms (selection bias) | Low risk | No evidence of a difference in baseline outcome measures between trial arms (Table 3). |
|  | Similarity of baseline characteristics across trial arms (selection bias) | Low risk | No evidence of a difference in baseline characteristics between trial arms (Table 3). |
|  | Blinding of participants and personnel (performance bias) | High risk | Trial was unblinded. |
|  | Blinded outcome assessment (assessor bias) | Low risk | “Interviewers who collected baseline and follow-up data from patients at six and 12 months were masked to clinician and patient intervention assignment.” See <https://doi.org/10.1186/1748-5908-5-18>.  Outcome assessment was blind. |
|  | Incomplete outcome data (attrition bias) | High risk | Amount of missing data was enough to induce bias in intervention effect estimate. |
|  | Similarity in attrition across trial arms (attrition bias) | Low risk | Similar proportion of participants lost to follow-up across trial arms (17% and 12%). |
| [56] Copello A, Templeton L, Orford J, Velleman R, Patel A, Moore L, et al. The relative efficacy of two levels of a primary care intervention for family members affected by the addiction problem of a close relative: a randomized trial. Addiction. 2009;104:49-58. | Jadad score (0-5; N/A for trial protocols) | 2 | Study was described as randomised and included a description of withdrawals/dropouts. |
|  | Allocation sequence (selection bias) | Unclear | Not enough information to make judgement. |
|  | Allocation concealment (selection bias) | Low risk | The trial statistician conducted randomisation, “which was hence removed from those responsible for recruitment into the trial.”  Allocation was concealed. |
|  | Order of randomisation and consent (selection bias) | High risk | The trial flow chart (Figure 1) indicates that participant enrolment took place after randomisation.  Randomisation preceded consent. |
|  | Order of randomisation and baseline measures (selection bias) | High risk | The trial flow chart (Figure 1) indicates that participant enrolment took place after randomisation.  Randomisation preceded baseline measures. |
|  | Similarity of baseline outcome measurements across trial arms (selection bias) | Low risk | No evidence of a difference in baseline outcome measures between trial arms (Table 1). |
|  | Similarity of baseline characteristics across trial arms (selection bias) | Low risk | No evidence of a difference in baseline characteristics between trial arms (Table 1). |
|  | Blinding of participants and personnel (performance bias) | High risk | Trial was unblinded. |
|  | Blinded outcome assessment (assessor bias) | High risk | Baseline data were collected by the individuals delivering the intervention.  Outcome assessment was not blind. |
|  | Incomplete outcome data (attrition bias) | High risk | Amount of missing data was enough to induce bias in intervention effect estimate. |
|  | Similarity in attrition across trial arms (attrition bias) | Low risk | Similar proportion of participants lost to follow-up across trial arms (12% and 9%). |
| [57] Courneya KS, Friedenreich CM, Sela RA, Quinney H, Rhodes RE, Handman M. The group psychotherapy and home-based physical exercise (group-hope) trial in cancer survivors: Physical fitness and quality of life outcomes. Psycho-Oncology. 2003;12:357-74. | Jadad score (0-5; N/A for trial protocols) | 3 | Study was described as randomised, with appropriate utilisation of random allocation, and included a description of withdrawals/dropouts. |
|  | Allocation sequence (selection bias) | Low risk | Used table of random numbers. |
|  | Allocation concealment (selection bias) | Unclear | Not enough information to make judgement. |
|  | Order of randomisation and consent (selection bias) | Low risk | The trial flow chart (Figure 1) indicates that participant enrolment took place before randomisation.  Randomisation followed consent. |
|  | Order of randomisation and baseline measures (selection bias) | Low risk | Baseline measures were collected at the same time as consent was obtained. This together with the information that consent preceded randomisation (Figure 1) suggests that baseline measures preceded randomisation. |
|  | Similarity of baseline outcome measurements across trial arms (selection bias) | Low risk | “We compared experimental conditions at baseline on our 13 outcome measures and found only one significant difference.”  Little evidence of a difference in baseline outcome measures between trial arms. |
|  | Similarity of baseline characteristics across trial arms (selection bias) | Low risk | “We compared experimental conditions on all demographic and medical variables and found only one difference.”  Little evidence of a difference in baseline characteristics between trial arms. |
|  | Blinding of participants and personnel (performance bias) | High risk | Trial was unblinded. |
|  | Blinded outcome assessment (assessor bias) | Low risk | “The fitness test was conducted by a certified fitness appraiser (blinded to the experimental condition”  Outcome assessment was blind. |
|  | Incomplete outcome data (attrition bias) | High risk | Amount of missing data was enough to induce bias in intervention effect estimate. |
|  | Similarity in attrition across trial arms (attrition bias) | High risk | Similar proportion of participants lost to follow-up across trial arms (6% and 15%). |
| [58] Coventry P, Lovell K, Dickens C, Bower P, Chew-Graham C, McElvenny D, et al. Integrated primary care for patients with mental and physical multimorbidity: cluster randomised controlled trial of collaborative care for patients with depression comorbid with diabetes or cardiovascular disease. BMJ February. 2015;14. | Jadad score (0-5; N/A for trial protocols) | 3 | Study was described as randomised, with appropriate utilisation of random allocation, and included a description of withdrawals/dropouts. |
|  | Allocation sequence (selection bias) | Low risk | Used minimisation. |
|  | Allocation concealment (selection bias) | Low risk | Used central randomisation service. |
|  | Order of randomisation and consent (selection bias) | High risk | The trial flow chart (CONSORT Figure) indicates that participant enrolment took place after randomisation.  Randomisation preceded consent. |
|  | Order of randomisation and baseline measures (selection bias) | High risk | The trial flow chart (CONSORT Figure) indicates that participant enrolment took place after randomisation.  Randomisation preceded baseline measures. |
|  | Similarity of baseline outcome measurements across trial arms (selection bias) | Low risk | No evidence of a difference in baseline outcome measures between trial arms (Table 2). |
|  | Similarity of baseline characteristics across trial arms (selection bias) | Low risk | No evidence of a difference in baseline characteristics between trial arms (Table 2). |
|  | Blinding of participants and personnel (performance bias) | High risk | Trial was unblinded. |
|  | Blinded outcome assessment (assessor bias) | Low risk | “Researchers who collected outcome data remained blinded to  treatment allocation throughout the course of the trial.”  Outcome assessment was blind. |
|  | Incomplete outcome data (attrition bias) | Low risk | Amount of missing data was low and balanced across trial arms. |
|  | Similarity in attrition across trial arms (attrition bias) | Low risk | Similar proportion of participants lost to follow-up across trial arms (11% and 8%). |
| [59] Craig TKJ, Johnson S, McCrone P, Afuwape S, Hughes E, Gournay K, et al. Integrated Care for Co-occurring Disorders: Psychiatric Symptoms, Social Functioning, and Service Costs at 18 Months. Psychiatric Services. 2008;59:276-82. | Jadad score (0-5; N/A for trial protocols) | 2 | Study was described as randomised and included a description of withdrawals/dropouts. |
|  | Allocation sequence (selection bias) | Unclear | Not enough information to make judgement. |
|  | Allocation concealment (selection bias) | Unclear | Not enough information to make judgement. |
|  | Order of randomisation and consent (selection bias) | Unclear | Not enough information to make judgement. |
|  | Order of randomisation and baseline measures (selection bias) | Unclear | Not enough information to make judgement. |
|  | Similarity of baseline outcome measurements across trial arms (selection bias) | Low risk | No evidence of a difference in baseline outcome measures between trial arms (Table 1). |
|  | Similarity of baseline characteristics across trial arms (selection bias) | Low risk | Results section describes the patient sample being balanced for sex, relationship status, employment status and ethnicity.  No evidence of a difference in baseline characteristics between trial arms. |
|  | Blinding of participants and personnel (performance bias) | High risk | Trial was unblinded. |
|  | Blinded outcome assessment (assessor bias) | High risk | “Although the investigators were independent of the training or clinical service, they were not blind to patients' intervention or control group status at follow-up and thus it is possible that the positive outcomes that were dependent on observer judgment could be attributed to bias.”  Outcome assessment was not blind. |
|  | Incomplete outcome data (attrition bias) | High risk | Amount of missing data was enough to induce bias in intervention effect estimate. |
|  | Similarity in attrition across trial arms (attrition bias) | High risk | Different proportion of participants lost to follow-up across trial arms (38% and 26%). |
| [60] Cullen AE, Clarke AY, Kuipers E, Hodgins S, Dean K, Fahy T. A Multisite Randomized Trial of a Cognitive Skills Program for Male Mentally Disordered Offenders: Violence and Antisocial Behavior Outcomes. Journal of Consulting & Clinical Psychology. 2012;80:1114-20. | Jadad score (0-5; N/A for trial protocols) | 2 | Study was described as randomised and included a description of withdrawals/dropouts. |
|  | Allocation sequence (selection bias) | Unclear | Not enough information to make judgement. |
|  | Allocation concealment (selection bias) | Low risk | “Block randomization stratified by site was conducted using equal block sizes with concealed allocation.”  Allocation was concealed. |
|  | Order of randomisation and consent (selection bias) | Low risk | The trial flow chart (Figure 1) indicates that participant enrolment took place befor randomisation.  Randomisation followed consent. |
|  | Order of randomisation and baseline measures (selection bias) | Low risk | “Baseline assessments, including clinical and research interviews and a review of the clinical file, were conducted prior to randomization.”  Randomisation followed baseline measures. |
|  | Similarity of baseline outcome measurements across trial arms (selection bias) | Low risk | No evidence of a difference in baseline outcome measures between trial arms (Table 1). |
|  | Similarity of baseline characteristics across trial arms (selection bias) | Low risk | No evidence of a difference in baseline characteristics between trial arms (Table 1). |
|  | Blinding of participants and personnel (performance bias) | High risk | Trial was unblinded. |
|  | Blinded outcome assessment (assessor bias) | High risk | “Researchers who conducted assessments were not blinded to allocation status.”  Outcome assessment was not blind. |
|  | Incomplete outcome data (attrition bias) | Low risk | Amount of missing data was low and balanced across trial arms. |
|  | Similarity in attrition across trial arms (attrition bias) | Low risk | Similar proportion of participants lost to follow-up across trial arms (5% and 0%). |
| [61] Dakof GA, Henderson CE, Rowe CL, Boustani M, Greenbaum PE, Wang W, et al. A randomized clinical trial of family therapy in juvenile drug court. Journal of family psychology : JFP : journal of the Division of Family Psychology of the American Psychological Association. 2015;29:232-41. | Jadad score (0-5; N/A for trial protocols) | 3 | Study was described as randomised, with appropriate utilisation of random allocation, and included a description of withdrawals/dropouts. |
|  | Allocation sequence (selection bias) | Low risk | Used urn randomisation procedure. |
|  | Allocation concealment (selection bias) | Unclear | Not enough information to make judgement. |
|  | Order of randomisation and consent (selection bias) | Low risk | The trial flow chart (Figure 1) indicates that participant enrolment took place before randomisation.  Randomisation followed consent. |
|  | Order of randomisation and baseline measures (selection bias) | Unclear | Not enough information to make judgement. |
|  | Similarity of baseline outcome measurements across trial arms (selection bias) | Low risk | No evidence of a difference in baseline outcome measures between trial arms (Tables 1 and 2). |
|  | Similarity of baseline characteristics across trial arms (selection bias) | Low risk | No evidence of a difference in baseline characteristics between trial arms (Table 1). |
|  | Blinding of participants and personnel (performance bias) | High risk | Trial was unblinded. |
|  | Blinded outcome assessment (assessor bias) | Low risk | “Efforts were made to keep assessors unaware of study hypotheses and treatment assignment.”  Outcome assessment was blind/ |
|  | Incomplete outcome data (attrition bias) | High risk | Amount of missing data was enough to induce bias in intervention effect estimate. |
|  | Similarity in attrition across trial arms (attrition bias) | High risk | Different proportion of participants lost to follow-up across trial arms (7% and 25%). |
| [62] De Wit M, Delemarre-Van De Waal HA, Bokma JA, Haasnoot K, Houdijk MC, Gemke RJ, et al. Monitoring and Discussing Health-Related Quality of Life in Adolescents With Type 1 Diabetes Improve Psychosocial Well-Being: A randomized controlled trial. Diabetes Care. 2008;31:1521-6. | Jadad score (0-5; N/A for trial protocols) | 2 | Study was described as randomised and included a description of withdrawals/dropouts. |
|  | Allocation sequence (selection bias) | Unclear | Not enough information to make judgement. |
|  | Allocation concealment (selection bias) | Unclear | Not enough information to make judgement. |
|  | Order of randomisation and consent (selection bias) | Unclear | Not enough information to make judgement. |
|  | Order of randomisation and baseline measures (selection bias) | Unclear | Not enough information to make judgement. |
|  | Similarity of baseline outcome measurements across trial arms (selection bias) | Low risk | No evidence of a difference in baseline outcome measures between trial arms (Table 1). |
|  | Similarity of baseline characteristics across trial arms (selection bias) | Low risk | No evidence of a difference in baseline characteristics between trial arms (Table 1). |
|  | Blinding of participants and personnel (performance bias) | High risk | Trial was unblinded. |
|  | Blinded outcome assessment (assessor bias) | N/A | No outcome assessors (participants completed questionnaires). |
|  | Incomplete outcome data (attrition bias) | Low risk | No incomplete outcome data. |
|  | Similarity in attrition across trial arms (attrition bias) | Low risk | No participants were lost over follow-up. |
| [63] Dechamps A, Alban R, Jen J, Decamps A, Traissac T, Dehail P. Individualized Cognition-Action intervention to prevent behavioral disturbances and functional decline in institutionalized older adults: a randomized pilot trial. International Journal of Geriatric Psychiatry. 2010;25:850-60. | Jadad score (0-5; N/A for trial protocols) | 3 | Study was described as randomised, with appropriate utilisation of random allocation, and included a description of withdrawals/dropouts. |
|  | Allocation sequence (selection bias) | Low risk | Used computerised randomisation. |
|  | Allocation concealment (selection bias) | Unclear | Not enough information to make judgement. |
|  | Order of randomisation and consent (selection bias) | Low risk | The trial flow chart (Figure 1) indicates that participant enrolment took place before randomisation.  Randomisation followed consent. |
|  | Order of randomisation and baseline measures (selection bias) | Unclear | Not enough information to make judgement. |
|  | Similarity of baseline outcome measurements across trial arms (selection bias) | Low risk | “There was no significant difference between groups at baseline in any of patient characteristics.”  No evidence of a difference in baseline outcome measures between trial arms. |
|  | Similarity of baseline characteristics across trial arms (selection bias) | Unclear | Not enough information to make judgement. |
|  | Blinding of participants and personnel (performance bias) | High risk | Trial was unblinded. |
|  | Blinded outcome assessment (assessor bias) | Unclear | Some outcome measures were assessed by blind researchers. Not enough information about whether all were. |
|  | Incomplete outcome data (attrition bias) | High risk | No incomplete outcome data. |
|  | Similarity in attrition across trial arms (attrition bias) | Low risk | No participants were lost over follow-up. |
| [64] Dennis M, Titus JC, Diamond G, Donaldson J, Godley SH, Tims FM, et al. The Cannabis Youth Treatment (CYT) experiment: rationale, study design and analysis plans. Addiction Supplement. 2002;97 Supplement:16-34. | Jadad score (0-5; N/A for trial protocols) | N/A | (Trial rationale, design and analysis plan) |
|  | Allocation sequence (selection bias) | Unclear | Not enough information to make judgement. |
|  | Allocation concealment (selection bias) | Unclear | Not enough information to make judgement. |
|  | Order of randomisation and consent (selection bias) | Low risk | “Only those who were eligible, agreed to participate, had parental consent and who completed all intake assessments were assigned to [a] treatment.”  Randomisation followed consent. |
|  | Order of randomisation and baseline measures (selection bias) | Unclear | Not enough information to make judgement. |
|  | Similarity of baseline outcome measurements across trial arms (selection bias) | Unclear | Not enough information to make judgement. |
|  | Similarity of baseline characteristics across trial arms (selection bias) | Low risk | “Adolescents in the alternative arm were more likely to be African American, female, and to be sexually active, as well as less likely to be employed.”  Little strong evidence of a difference in baseline characteristics between trial arms (see <https://doi.org/10.1046/j.1360-0443.97.s01.7.x>). |
|  | Blinding of participants and personnel (performance bias) | High risk | Trial was unblinded. |
|  | Blinded outcome assessment (assessor bias) | N/A | (Trial rationale, design and analysis plan) |
|  | Incomplete outcome data (attrition bias) | N/A | (Trial rationale, design and analysis plan) |
|  | Similarity in attrition across trial arms (attrition bias) | N/A | (Trial rationale, design and analysis plan) |
| [65] Deudon A, Maubourguet N, Gervais X, Leone E, Brocker P, Carcaillon L, et al. Non-pharmacological management of behavioural symptoms in nursing homes. International Journal of Geriatric Psychiatry. 2009;24:1386-95. | Jadad score (0-5; N/A for trial protocols) | 2 | Study was described as randomised and included a description of withdrawals/dropouts. |
|  | Allocation sequence (selection bias) | Unclear | Not enough information to make judgement. |
|  | Allocation concealment (selection bias) | Unclear | Not enough information to make judgement. |
|  | Order of randomisation and consent (selection bias) | N/A | “As the study was classified as a routine clinical procedure, no signed informed consent was required from patients or caregivers.” |
|  | Order of randomisation and baseline measures (selection bias) | High risk | Clusters (nursing homes) were assigned to treatments before baseline measures were collected.  Randomisation preceded baseline measures. |
|  | Similarity of baseline outcome measurements across trial arms (selection bias) | High risk | Evidence of a difference in baseline outcome measures between trial arms (Table 1). |
|  | Similarity of baseline characteristics across trial arms (selection bias) | Low risk | No evidence of a difference in baseline characteristics between trial arms (Table 1). |
|  | Blinding of participants and personnel (performance bias) | High risk | Trial was unblinded. |
|  | Blinded outcome assessment (assessor bias) | Low risk | “Assessments were performed by four psychologists blind to the intervention condition.”  Outcome assessors were blind. |
|  | Incomplete outcome data (attrition bias) | High risk | Amount of missing data was enough to induce bias in intervention effect estimate. |
|  | Similarity in attrition across trial arms (attrition bias) | Low risk | Similar proportion of participants lost to follow-up across trial arms (14% and 9%). |
| [66] Dilley JW, Woods WJ, Loeb L, Nelson K, Sheon N, Mullan J, et al. Brief Cognitive Counseling With HIV Testing To Reduce Sexual Risk Among Men Who Have Sex With Men: Results From a Randomized Controlled Trial Using Paraprofessional Counselors. JAIDS Journal of Acquired Immune Deficiency Syndromes. 2007;44:569-77. | Jadad score (0-5; N/A for trial protocols) | 3 | Study was described as randomised, with appropriate utilisation of random allocation, and included a description of withdrawals/dropouts. |
|  | Allocation sequence (selection bias) | Low risk | Used card shuffling |
|  | Allocation concealment (selection bias) | Unclear | Used opaque envelopes. Not clear if they were opaque.  Not enough information to make judgement. |
|  | Order of randomisation and consent (selection bias) | Low risk | “The study interviewer obtained the participant's written informed consent when he presented for testing, administered the baseline interview, and retrieved the random assignment to one of 2 study arms.”  Randomisation followed consent. |
|  | Order of randomisation and baseline measures (selection bias) | Low risk | “The study interviewer obtained the participant's written informed consent when he presented for testing, administered the baseline interview, and retrieved the random assignment to one of 2 study arms.”  Randomisation followed baseline measures. |
|  | Similarity of baseline outcome measurements across trial arms (selection bias) | Low risk | No evidence of a difference in baseline outcome measures between trial arms (Tables 2 and 3). |
|  | Similarity of baseline characteristics across trial arms (selection bias) | Low risk | “There were no significant differences between groups on any demographic characteristics (Table 1) at baseline.”  No evidence of a difference in baseline characteristics between trial arms. |
|  | Blinding of participants and personnel (performance bias) | High risk | Trial was unblinded. |
|  | Blinded outcome assessment (assessor bias) | Low risk | Used computer-assisted interview technology.  Outcome assessment was blind. |
|  | Incomplete outcome data (attrition bias) | High risk | Amount of missing data was enough to induce bias in intervention effect estimate. |
|  | Similarity in attrition across trial arms (attrition bias) | High risk | Different proportion of participants lost to follow-up across trial arms (16% and 8%). |
| [67] Dobscha SK, Corson K, Perrin NA, Hanson GC, Leibowitz RQ, Doak MN, et al. Collaborative Care for Chronic Pain in Primary Care: A Cluster Randomized Trial. JAMA. 2009;301:1242-52. | Jadad score (0-5; N/A for trial protocols) | 2 | Study was described as randomised and included a description of withdrawals/dropouts. |
|  | Allocation sequence (selection bias) | Unclear | Not enough information to make judgement. |
|  | Allocation concealment (selection bias) | Unclear | Not enough information to make judgement. |
|  | Order of randomisation and consent (selection bias) | High risk | The trial flow chart (Figure 1) indicates that participant enrolment took place after randomisation.  Randomisation preceded consent. |
|  | Order of randomisation and baseline measures (selection bias) | High risk | The trial flow chart (Figure 1) indicates that participant enrolment took place after randomisation.  Randomisation preceded baseline measures. |
|  | Similarity of baseline outcome measurements across trial arms (selection bias) | Low risk | No evidence of a difference in baseline outcome measures between trial arms (Table 2). |
|  | Similarity of baseline characteristics across trial arms (selection bias) | Low risk | No evidence of a difference in baseline characteristics between trial arms (Table 2). |
|  | Blinding of participants and personnel (performance bias) | High risk | Trial was unblinded. |
|  | Blinded outcome assessment (assessor bias) | Low risk | “Research assistants, blinded to the study group status, collected patient data at baseline, 3, 6, and 12 months.”  Outcome assessment was blinded. |
|  | Incomplete outcome data (attrition bias) | Low risk | Amount of missing data was low and balanced across trial arms. |
|  | Similarity in attrition across trial arms (attrition bias) | Low risk | Similar proportion of participants lost to follow-up across trial arms (10% and 10%). |
| [68] Dracup K, Moser DK, Doering LV, Guzy PM, Juarbe T. A controlled trial of cardiopulmonary resuscitation training for ethnically diverse parents of infants at high risk for cardiopulmonary arrest. Critical Care Medicine. 2000;28:3289-95. | Jadad score (0-5; N/A for trial protocols) | 1 | Study was described as randomised. |
|  | Allocation sequence (selection bias) | Unclear | Not enough information to make judgement. |
|  | Allocation concealment (selection bias) | Unclear | Not enough information to make judgement. |
|  | Order of randomisation and consent (selection bias) | Unclear | Not enough information to make judgement. |
|  | Order of randomisation and baseline measures (selection bias) | Unclear | Not enough information to make judgement. |
|  | Similarity of baseline outcome measurements across trial arms (selection bias) | Low risk | No evidence of a difference in baseline outcome measures between trial arms (Table 2). |
|  | Similarity of baseline characteristics across trial arms (selection bias) | High risk | Evidence of a difference in baseline characteristics between trial arms (Table 1). |
|  | Blinding of participants and personnel (performance bias) | High risk | Trial was unblinded. |
|  | Blinded outcome assessment (assessor bias) | Unclear | Not enough information to make judgement. |
|  | Incomplete outcome data (attrition bias) | High risk | Amount of missing data was enough to induce bias in intervention effect estimate. |
|  | Similarity in attrition across trial arms (attrition bias) | Unclear | Not enough information to make judgement. |
| [69] Ell K, Katon W, Xie B, Lee P-J, Kapetanovic S, Guterman J, et al. Collaborative Care Management of Major Depression Among Low-Income, Predominantly Hispanic Subjects With Diabetes: A randomized controlled trial. Diabetes Care. 2010;33:706-13. | Jadad score (0-5; N/A for trial protocols) | 3 | Study was described as randomised, with appropriate utilisation of random allocation, and included a description of withdrawals/dropouts. |
|  | Allocation sequence (selection bias) | Low risk | Used computer-generated random numbers. |
|  | Allocation concealment (selection bias) | Unclear | “Random assignment number [was] enclosed in sealed envelopes.” Not clear if envelopes were opaque and if they were sequentially numbered.  Not enough information to make judgement. |
|  | Order of randomisation and consent (selection bias) | Low risk | The trial flow chart (Figure 1) indicates that consent took place before randomisation.  Randomisation followed consent. |
|  | Order of randomisation and baseline measures (selection bias) | Low risk | “Patients selected 1 of 5 sequential envelopes [containing random allocation] following baseline interview.”  Randomisation followed baseline measures. |
|  | Similarity of baseline outcome measurements across trial arms (selection bias) | High risk | Evidence of a difference in baseline outcome measures between trial arms (Table 2). |
|  | Similarity of baseline characteristics across trial arms (selection bias) | High risk | Evidence of a difference in baseline characteristics between trial arms (Table 1). |
|  | Blinding of participants and personnel (performance bias) | High risk | Trial was unblinded. |
|  | Blinded outcome assessment (assessor bias) | Low risk | Outcomes assessed at “18-month blinded survey”.  Outcome assessment was blind. |
|  | Incomplete outcome data (attrition bias) | High risk | Amount of missing data was enough to induce bias in intervention effect estimate. |
|  | Similarity in attrition across trial arms (attrition bias) | Low risk | Similar proportion of participants lost to follow-up across trial arms (29% and 25%). |
| [70] Ersek M, Turner JA, Cain KC, Kemp CA. Results of a randomized controlled trial to examine the efficacy of a chronic pain self-management group for older adults [ISRCTN11899548]. Pain. 2008;138:29-40. | Jadad score (0-5; N/A for trial protocols) | 3 | Study was described as randomised, with appropriate utilisation of random allocation, and included a description of withdrawals/dropouts. |
|  | Allocation sequence (selection bias) | Low risk | Used computerised random number generator. |
|  | Allocation concealment (selection bias) | Unclear | Not enough information to make judgement. |
|  | Order of randomisation and consent (selection bias) | Low risk | “The facilities within each pair were randomized after the end of participant recruitment and baseline measure completion at both facilities.” Recruitment and therefore consent  Randomisation followed consent. |
|  | Order of randomisation and baseline measures (selection bias) | Low risk | “Study participants completed measures at baseline (prior to randomization).”  Randomisation followed baseline measures. |
|  | Similarity of baseline outcome measurements across trial arms (selection bias) | Low risk | No evidence of a difference in baseline outcome measures between trial arms (Table 3). |
|  | Similarity of baseline characteristics across trial arms (selection bias) | Low risk | No evidence of a difference in baseline characteristics between trial arms (Table 2). |
|  | Blinding of participants and personnel (performance bias) | High risk | Trial was unblinded. |
|  | Blinded outcome assessment (assessor bias) | Unclear | Not enough information to make judgement. |
|  | Incomplete outcome data (attrition bias) | High risk | Amount of missing data was enough to induce bias in intervention effect estimate. |
|  | Similarity in attrition across trial arms (attrition bias) | Low risk | Similar proportion of participants lost to follow-up across trial arms (14% and 16%). |
| [71] Farooq S, Nazar Z, Irfan M, Akhter J, Gul E, Irfan U, et al. Schizophrenia medication adherence in a resource-poor setting: randomised controlled trial of supervised treatment in out-patients for schizophrenia (STOPS). British Journal of Psychiatry. 2011;199:467-72. | Jadad score (0-5; N/A for trial protocols) | 2 | Study was described as randomised, and included a description of withdrawals/dropouts. |
|  | Allocation sequence (selection bias) | Unclear | Not enough information to make judgement. |
|  | Allocation concealment (selection bias) | Low risk | “The random allocations of patients to each group were enclosed in opaque envelopes which were sealed and numbered sequentially. These allocations were placed away from the site of assessment. After assessment and satisfying the inclusion criteria, the staff which were not part of the study were asked to open the sealed envelope and reveal the treatment arm for each patient.”  Allocation was concealed. |
|  | Order of randomisation and consent (selection bias) | Low risk | The trial flow chart (Figure 1) indicates that consent took place before randomisation.  Randomisation followed consent. |
|  | Order of randomisation and baseline measures (selection bias) | Unclear | Not enough information to make judgement. |
|  | Similarity of baseline outcome measurements across trial arms (selection bias) | Low risk | No evidence of a difference in baseline outcome measures between trial arms (Table 3). |
|  | Similarity of baseline characteristics across trial arms (selection bias) | Low risk | No evidence of a difference in baseline characteristics between trial arms (Table 1). |
|  | Blinding of participants and personnel (performance bias) | High risk | Trial was unblinded. |
|  | Blinded outcome assessment (assessor bias) | Low risk | “The follow-up assessments were done by researchers who were masked to participant group assignment…”  Outcome assessment was blind. |
|  | Incomplete outcome data (attrition bias) | High risk | Used inappropriate simple imputation (used last observation carried forward). |
|  | Similarity in attrition across trial arms (attrition bias) | Low risk | Similar proportion of participants lost to follow-up across trial arms (11% and 16%). |
| [72] van der Feltz-Cornelis CM, van Oppen P, Ader HJ, van Dyck R. Randomised Controlled Trial of a Collaborative Care Model with Psychiatric Consultation for Persistent Medically Unexplained Symptoms in General Practice. Psychotherapy & Psychosomatics. 2006;75:282-9. | Jadad score (0-5; N/A for trial protocols) | 2 | Study was described as randomised, and included a description of withdrawals/dropouts. |
|  | Allocation sequence (selection bias) | Unclear | Not enough information to make judgement. |
|  | Allocation concealment (selection bias) | Unclear | Not enough information to make judgement. |
|  | Order of randomisation and consent (selection bias) | Low risk | “The informed consent procedure was set up in such a way that it did not disturb the blinding procedure. The practices were randomly assigned to different treatment allocations, but the GPs and patients were only informed about this after the recruitment stage.”  Randomisation in effect followed consent. |
|  | Order of randomisation and baseline measures (selection bias) | Low risk | Part of participant enrolment involved filling in a baseline questionnaire. Random assignment was unknown by clinicians and participants before enrolment had finished.  Randomisation in effect followed baseline measures. |
|  | Similarity of baseline outcome measurements across trial arms (selection bias) | Low risk | “There were no significant differences at baseline between the two treatment groups.”  No evidence of a difference in baseline outcome measures between trial arms |
|  | Similarity of baseline characteristics across trial arms (selection bias) | Low risk | “There were no significant differences at baseline between the two treatment groups.”  No evidence of a difference in baseline characteristics between trial arms |
|  | Blinding of participants and personnel (performance bias) | High risk | Trial was unblinded. |
|  | Blinded outcome assessment (assessor bias) | N/A | No outcome assessors (participants completed questionnaires). |
|  | Incomplete outcome data (attrition bias) | Low risk | No incomplete outcome data. |
|  | Similarity in attrition across trial arms (attrition bias) | Low risk | No participants were lost over follow-up. |
| [73] Finnema E, Droes R-M, Ettema T, Ooms M, Ader H, Ribbe M, et al. The effect of integrated emotion-oriented care versus usual care on elderly persons with dementia in the nursing home and on nursing assistants: a randomized clinical trial. International Journal of Geriatric Psychiatry. 2005;20:330-43. | Jadad score (0-5; N/A for trial protocols) | 1 | Study was described as randomised. |
|  | Allocation sequence (selection bias) | Unclear | Not enough information to make judgement. |
|  | Allocation concealment (selection bias) | Unclear | Not enough information to make judgement. |
|  | Order of randomisation and consent (selection bias) | High risk | Recruitment took place in phases: selection and matching of clusters (wards), allocation of treatment to clusters, then selection of participants.  Randomisation preceded consent. |
|  | Order of randomisation and baseline measures (selection bias) | High risk | Recruitment took place in phases: selection and matching of clusters (wards), allocation of treatment to clusters, then selection of participants.  Randomisation preceded baseline measures. |
|  | Similarity of baseline outcome measurements across trial arms (selection bias) | Low risk | No evidence of a difference in baseline outcome measures between trial arms (Table 3). |
|  | Similarity of baseline characteristics across trial arms (selection bias) | High risk | Some evidence of a difference in baseline characteristics between trial arms (Table 1). |
|  | Blinding of participants and personnel (performance bias) | High risk | Trial was unblinded. |
|  | Blinded outcome assessment (assessor bias) | High risk | “… we mailed the questionnaires to the contact persons in the nursing homes, who then handled the distribution and collection of the completed questionnaires.” Treatment allocation was known within clusters (wards).  Outcome assessment was not blind. |
|  | Incomplete outcome data (attrition bias) | Low risk | Amount of missing data was very low and therefore must be balanced across trial arms. |
|  | Similarity in attrition across trial arms (attrition bias) | Low risk | Amount of missing data was very low and therefore must be balanced across trial arms. |
| [74] Forchuk C, Martin ML, Chan YL, Jensen E. Therapeutic relationships: from psychiatric hospital to community. Journal of Psychiatric & Mental Health Nursing. 2005;12:556-64. | Jadad score (0-5; N/A for trial protocols) | 1 | Study was described as randomised. |
|  | Allocation sequence (selection bias) | Unclear | Not enough information to make judgement. |
|  | Allocation concealment (selection bias) | Unclear | Not enough information to make judgement. |
|  | Order of randomisation and consent (selection bias) | High risk | “After randomization, staff training occurred on the intervention wards... Sequential patients discharged from the study wards were invited to participate in the study.”  Randomisation preceded consent. |
|  | Order of randomisation and baseline measures (selection bias) | High risk | “After randomization, staff training occurred on the intervention wards... Sequential patients discharged from the study wards were invited to participate in the study.”  Randomisation preceded baseline measures. |
|  | Similarity of baseline outcome measurements across trial arms (selection bias) | N/A | No information on baseline measures. |
|  | Similarity of baseline characteristics across trial arms (selection bias) | Low risk | “The intervention and control participants appeared similar at enrolment.”  No evidence of a difference in baseline characteristics between trial arms (Table 1). |
|  | Blinding of participants and personnel (performance bias) | High risk | Trial was unblinded. |
|  | Blinded outcome assessment (assessor bias) | Low risk | “The three research assistants and six student assistants who conducted the data collection were blind to the ward assignment in the study.”  Outcome assessment was blind. |
|  | Incomplete outcome data (attrition bias) | High risk | Amount of missing data was enough (36% drop-out) to induce bias in intervention effect estimate. |
|  | Similarity in attrition across trial arms (attrition bias) | Unclear | Not enough information to make judgement. |
| [75] Foshee VA, Bauman KE, Greene WF, Koch GG, Linder GF, MacDougall JE. The Safe Dates Program: 1-Year Follow-Up Results. American Journal of Public Health Disease Elimination and Eradication. 2000;90:1619-22. | Jadad score (0-5; N/A for trial protocols) | 1 | Study was described as randomised. |
|  | Allocation sequence (selection bias) | Unclear | Not enough information to make judgement. |
|  | Allocation concealment (selection bias) | Unclear | Not enough information to make judgement. |
|  | Order of randomisation and consent (selection bias) | Unclear | Not enough information to make judgement. |
|  | Order of randomisation and baseline measures (selection bias) | Unclear | Not enough information to make judgement. |
|  | Similarity of baseline outcome measurements across trial arms (selection bias) | Low risk | “We found no significant (P< .05) baseline differences between treatment and control groups on outcome, mediating, or demographic variables in any of the samples.”  No evidence of a difference in baseline outcome measures between trial arms. |
|  | Similarity of baseline characteristics across trial arms (selection bias) | Low risk | “We found no significant (P< .05) baseline differences between treatment and control groups on outcome, mediating, or demographic variables in any of the samples.”  No evidence of a difference in baseline characteristics between trial arms. |
|  | Blinding of participants and personnel (performance bias) | High risk | Trial was unblinded. |
|  | Blinded outcome assessment (assessor bias) | N/A | No outcome assessors (participants completed questionnaires). |
|  | Incomplete outcome data (attrition bias) | High risk | Amount of missing data was enough to induce bias in intervention effect estimate. |
|  | Similarity in attrition across trial arms (attrition bias) | Low risk | “…attrition rates were very similar for treatment (14.76%) and control (15.25%) groups.”  Similar proportion of participants lost to follow-up across trial arms. |
| [76] Fossey J, Ballard C, Juszczak E, James I, Alder N, Jacoby R, et al. Effect of enhanced psychosocial care on antipsychotic use in nursing home residents with severe dementia: cluster randomised trial. BMJ. 2006;332:756-61. | Jadad score (0-5; N/A for trial protocols) | 3 | Study was described as randomised, with appropriate utilisation of random allocation, and included a description of withdrawals/dropouts. |
|  | Allocation sequence (selection bias) | Low risk | Used computerised randomisation. |
|  | Allocation concealment (selection bias) | Low risk | Randomisation was done remotely (by trial statistician who was blind to identity of the homes).  Allocation was concealed. |
|  | Order of randomisation and consent (selection bias) | Low risk | “Consent was obtained from the management of the homes, and the family carers of residents were asked to give consent for involvement of their relatives. Research assistants carried out baseline assessments before randomisation.”  Randomisation followed consent. |
|  | Order of randomisation and baseline measures (selection bias) | Low risk | “Research assistants carried out baseline assessments before randomisation.”  Randomisation followed baseline measures. |
|  | Similarity of baseline outcome measurements across trial arms (selection bias) | Low risk | No evidence of a difference in baseline outcome measures between trial arms (Table 1 and Figure 2). |
|  | Similarity of baseline characteristics across trial arms (selection bias) | Low risk | No evidence of a difference in baseline characteristics between trial arms (Table 1). |
|  | Blinding of participants and personnel (performance bias) | High risk | Trial was unblinded. |
|  | Blinded outcome assessment (assessor bias) | High risk | “Assessments at 12 months were carried out by a psychology research assistant who had not been employed during the intervention period. This member of staff was blind to the homes' intervention… Despite efforts, because the package was designed to influence the whole care approach of staff, it is likely that the research assistant would have been able to detect which homes had received the intervention.”  Outcome assessment was likely not blind. |
|  | Incomplete outcome data (attrition bias) | Low risk | No loss to follow-up of units of analysis (care homes). |
|  | Similarity in attrition across trial arms (attrition bias) | Low risk | There was turnover of patients throughout the study but no loss of clusters (care homes). The care homes were treated as the units of analysis. |
| [77] Gallagher R, McKinley S, Dracup K. Effects of a telephone counseling intervention on psychosocial adjustment in women following a cardiac event. Heart & Lung: Journal of Acute & Critical Care March/April. 2003;32:79-87. | Jadad score (0-5; N/A for trial protocols) | 1 | Study was described as randomised. |
|  | Allocation sequence (selection bias) | Unclear | Not enough information to make judgement. |
|  | Allocation concealment (selection bias) | Unclear | Not enough information to make judgement. |
|  | Order of randomisation and consent (selection bias) | Unclear | Not enough information to make judgement. |
|  | Order of randomisation and baseline measures (selection bias) | Unclear | Not enough information to make judgement. |
|  | Similarity of baseline outcome measurements across trial arms (selection bias) | Low risk | “There were no significant differences at baseline between the control and intervention groups in… psychosocial characteristics.”  No evidence of a difference in baseline outcome measures between trial arms. |
|  | Similarity of baseline characteristics across trial arms (selection bias) | Low risk | “There were no significant differences at baseline between the control and intervention groups in sociodemographic, clinical characteristics.”  No evidence of a difference in baseline characteristics between trial arms. |
|  | Blinding of participants and personnel (performance bias) | High risk | Trial was unblinded. |
|  | Blinded outcome assessment (assessor bias) | Low risk | “Outcome data were collected at 12 weeks by telephone interview by a research assistant who was blinded to group assignment.”  Outcome assessment was blind. |
|  | Incomplete outcome data (attrition bias) | Unclear | Not enough information to make judgement. |
|  | Similarity in attrition across trial arms (attrition bias) | Unclear | Not enough information to make judgement. |
| [78] Garcia C, Pintor J, Vazquez G, Alvarez-Zumarraga E. Project Wings, a Coping Intervention for Latina Adolescents: A Pilot Study. Western Journal of Nursing Research. 2013;35:434-58. | Jadad score (0-5; N/A for trial protocols) | 3 | Study was described as randomised, with appropriate utilisation of random allocation, and included a description of withdrawals/dropouts. |
|  | Allocation sequence (selection bias) | Low risk | Used computerised random allocation. |
|  | Allocation concealment (selection bias) | Unclear | Not enough information to make judgement. |
|  | Order of randomisation and consent (selection bias) | Low risk | “Active parental consent and adolescent assent were obtained in writing prior to the initial group.” Following this, participants were randomised.  Randomisation followed consent. |
|  | Order of randomisation and baseline measures (selection bias) | Unclear | Not enough information to make judgement. |
|  | Similarity of baseline outcome measurements across trial arms (selection bias) | Low risk | No evidence of a difference in baseline outcome measures between trial arms (Table 2). |
|  | Similarity of baseline characteristics across trial arms (selection bias) | Low risk | No evidence of a difference in baseline characteristics between trial arms (Table 1). |
|  | Blinding of participants and personnel (performance bias) | High risk | Trial was unblinded. |
|  | Blinded outcome assessment (assessor bias) | N/A | No outcome assessors (participants completed questionnaires). |
|  | Incomplete outcome data (attrition bias) | High risk | Amount of missing data was enough to induce bias in intervention effect estimate. |
|  | Similarity in attrition across trial arms (attrition bias) | Low risk | Similar numbers of participants (5 and 3) were lost over follow-up. |
| [79] Garner BR, Godley SH, Dennis ML, Hunter BD, Bair CM, Godley MD. Using Pay for Performance to Improve Treatment Implementation for Adolescent Substance Use Disorders: Results From a Cluster Randomized Trial. Archives of Pediatrics & Adolescent Medicine. 2012;166:938-44. | Jadad score (0-5; N/A for trial protocols) | 3 | Study was described as randomised, with appropriate utilisation of random allocation, and included a description of withdrawals/dropouts. |
|  | Allocation sequence (selection bias) | Low risk | Used computerised randomisation. |
|  | Allocation concealment (selection bias) | Unclear | Not enough information to make judgement. |
|  | Order of randomisation and consent (selection bias) | High risk | The trial flow chart (Figure) indicates that participant enrolment took place after randomisation.  Randomisation preceded consent. |
|  | Order of randomisation and baseline measures (selection bias) | High risk | The trial flow chart (Figure) indicates that participant enrolment took place after randomisation.  Randomisation preceded baseline measures. |
|  | Similarity of baseline outcome measurements across trial arms (selection bias) | N/A | Outcomes (A-CRA competence, target A-CRA and remission status) not measured at baseline. |
|  | Similarity of baseline characteristics across trial arms (selection bias) | High risk | Evidence of a difference in baseline characteristics between trial arms (Table 1). |
|  | Blinding of participants and personnel (performance bias) | High risk | Trial was unblinded. |
|  | Blinded outcome assessment (assessor bias) | High risk | “It was impossible to blind organizations, therapists, or all research staff to condition assignment.”  Outcome assessment was not blind. |
|  | Incomplete outcome data (attrition bias) | High risk | Amount of missing data was enough to induce bias in intervention effect estimate. |
|  | Similarity in attrition across trial arms (attrition bias) | High risk | Different proportion of participants lost to follow-up across trial arms (26% and 34%). |
| [80] Gartner FR, Nieuwenhuijsen K, Ketelaar SM, van Dijk FJ, Sluiter JK. The Mental Vitality @ Work Study: Effectiveness of a Mental Module for Workers' Health Surveillance for Nurses and Allied Health Care Professionals on Their Help-Seeking Behavior. Journal of Occupational & Environmental Medicine. 2013;55:1219-29. | Jadad score (0-5; N/A for trial protocols) | 3 | Study was described as randomised, with appropriate utilisation of random allocation, and included a description of withdrawals/dropouts. |
|  | Allocation sequence (selection bias) | Low risk | Used computerised randomisation. |
|  | Allocation concealment (selection bias) | Low risk | Randomisation was done remotely (by researcher not involved in recruitment).  Allocation was concealed. |
|  | Order of randomisation and consent (selection bias) | High risk | The trial flow chart (Figure 1) indicates that participant enrolment took place after randomisation.  Randomisation preceded consent. |
|  | Order of randomisation and baseline measures (selection bias) | High risk | The trial flow chart (Figure 1) indicates that participant enrolment took place after randomisation.  Randomisation preceded baseline measures. |
|  | Similarity of baseline outcome measurements across trial arms (selection bias) | Low risk | No evidence of a difference in baseline outcome measures between trial arms (Table 2). |
|  | Similarity of baseline characteristics across trial arms (selection bias) | Low risk | No evidence of a difference in baseline characteristics between trial arms (Table 1). |
|  | Blinding of participants and personnel (performance bias) | High risk | Trial was unblinded. |
|  | Blinded outcome assessment (assessor bias) | N/A | No outcome assessors (participants completed questionnaires). |
|  | Incomplete outcome data (attrition bias) | High risk | Amount of missing data was enough to induce bias in intervention effect estimate. |
|  | Similarity in attrition across trial arms (attrition bias) | High risk | Different proportion of participants lost to follow-up across trial arms (40% and 25%). |
| [81] Gater R, Waheed W, Husain N, Tomenson B, Aseem S, Creed F. Social intervention for British Pakistani women with depression: randomised controlled trial. British Journal of Psychiatry. 2010;197:227-33. | Jadad score (0-5; N/A for trial protocols) | 3 | Study was described as randomised, with appropriate utilisation of random allocation, and included a description of withdrawals/dropouts. |
|  | Allocation sequence (selection bias) | Low risk | Used computerised randomisation. |
|  | Allocation concealment (selection bias) | Unclear | Not enough information to make judgement. |
|  | Order of randomisation and consent (selection bias) | Low risk | “Written informed consent was sought at screening and then again before randomisation.”  Randomisation followed consent. |
|  | Order of randomisation and baseline measures (selection bias) | Unclear | Not enough information to make judgement. |
|  | Similarity of baseline outcome measurements across trial arms (selection bias) | High risk | Evidence of a difference in baseline outcome measures between trial arms (see Table 1). |
|  | Similarity of baseline characteristics across trial arms (selection bias) | Low risk | “No significant differences were found between the participants in the three treatment groups with respect to demographics.”  No evidence of a difference in baseline characteristics between trial arms (also see Table 1). |
|  | Blinding of participants and personnel (performance bias) | High risk | Trial was unblinded. |
|  | Blinded outcome assessment (assessor bias) | Low risk | “Researchers independent of those providing the intervention and masked to the intervention allocation and adherence undertook outcome assessments.”  Outcome assessment was blind. |
|  | Incomplete outcome data (attrition bias) | High risk | Amount of missing data was enough to induce bias in intervention effect estimate. |
|  | Similarity in attrition across trial arms (attrition bias) | High risk | Different proportion of participants lost to follow-up across trial arms (29%, 5% and 21%). |
| [82] Gensichen J, von Korff M, Peitz M, Muth C, Beyer M, Guthlin C, et al. Case Management for Depression by Health Care Assistants in Small Primary Care Practices: A Cluster Randomized Trial. Annals of Internal Medicine. 2009;151:369-78. | Jadad score (0-5; N/A for trial protocols) | 3 | Study was described as randomised, with appropriate utilisation of random allocation, and included a description of withdrawals/dropouts. |
|  | Allocation sequence (selection bias) | Low risk | Used computerised randomisation. |
|  | Allocation concealment (selection bias) | Low risk | “Patient random assignment status was nested within the practice status. The data safety and monitoring board was responsible for allocation concealment by keeping the randomization results in a secure database.”  Allocation was concealed. |
|  | Order of randomisation and consent (selection bias) | High risk | The trial flow chart (Figure) indicates that participant enrolment took place after randomisation.  Randomisation preceded consent. |
|  | Order of randomisation and baseline measures (selection bias) | High risk | The trial flow chart (Figure) indicates that participant enrolment took place after randomisation.  Randomisation preceded baseline measures. |
|  | Similarity of baseline outcome measurements across trial arms (selection bias) | Low risk | No evidence of a difference in baseline outcome measures between trial arms (see Table 2). |
|  | Similarity of baseline characteristics across trial arms (selection bias) | Low risk | No evidence of a difference in baseline characteristics between trial arms (see Table 2). |
|  | Blinding of participants and personnel (performance bias) | High risk | Trial was unblinded. |
|  | Blinded outcome assessment (assessor bias) | High risk | “Health care assistants, family physicians, and researchers were not blinded to assignment once the trial was started.”  Outcome assessment was not blind. |
|  | Incomplete outcome data (attrition bias) | High risk | Amount of missing data was enough to induce bias in intervention effect estimate. |
|  | Similarity in attrition across trial arms (attrition bias) | High risk | Different proportion of participants lost to follow-up across trial arms (12% and 20%). |
| [83] Glazebrook C, Marlow N, Israel C, Croudace T, Johnson S, White IR, et al. Randomised trial of a parenting intervention during neonatal intensive care. Archives of Disease in Childhood Fetal & Neonatal Edition. 2007;92:F438-F43. | Jadad score (0-5; N/A for trial protocols) | 3 | Study was described as randomised, with appropriate utilisation of random allocation, and included a description of withdrawals/dropouts. |
|  | Allocation sequence (selection bias) | Low risk | Used coin tossing. |
|  | Allocation concealment (selection bias) | Unclear | Not enough information to make judgement. |
|  | Order of randomisation and consent (selection bias) | High risk | The trial flow chart (Figure) indicates that participant enrolment took place after randomisation.  Randomisation preceded consent. |
|  | Order of randomisation and baseline measures (selection bias) | High risk | The trial flow chart (Figure) indicates that participant enrolment took place after randomisation.  Randomisation preceded baseline measures. |
|  | Similarity of baseline outcome measurements across trial arms (selection bias) | Unclear | Not enough information to make judgement. |
|  | Similarity of baseline characteristics across trial arms (selection bias) | Low risk | No evidence of a difference in baseline characteristics between trial arms (see Table 1). |
|  | Blinding of participants and personnel (performance bias) | High risk | Trial was unblinded. |
|  | Blinded outcome assessment (assessor bias) | Low risk | “All raters were blind to the group allocation of the participant.”  Outcome assessment was blind. |
|  | Incomplete outcome data (attrition bias) | High risk | Amount of missing data was enough to induce bias in intervention effect estimate. |
|  | Similarity in attrition across trial arms (attrition bias) | Low risk | Different proportion of participants lost to follow-up across trial arms (32% and 33%). |
| [84] de Godoy DV, de Godoy RF. A randomized controlled trial of the effect of psychotherapy on anxiety and depression in chronic obstructive pulmonary disease. Archives of physical medicine and rehabilitation. 2003;84:1154-7. | Jadad score (0-5; N/A for trial protocols) | 2 | Study was described as randomised, and included a description of withdrawals/dropouts. |
|  | Allocation sequence (selection bias) | Unclear | Not enough information to make judgement. |
|  | Allocation concealment (selection bias) | Unclear | Not enough information to make judgement. |
|  | Order of randomisation and consent (selection bias) | Unclear | Not enough information to make judgement. |
|  | Order of randomisation and baseline measures (selection bias) | Unclear | Not enough information to make judgement. |
|  | Similarity of baseline outcome measurements across trial arms (selection bias) | Low risk | No evidence of a difference in baseline outcome measures between trial arms (see Table 1). |
|  | Similarity of baseline characteristics across trial arms (selection bias) | Low risk | No evidence of a difference in baseline characteristics between trial arms (see Table 1). |
|  | Blinding of participants and personnel (performance bias) | High risk | Trial was unblinded. |
|  | Blinded outcome assessment (assessor bias) | Unclear | Not enough information to make judgement. |
|  | Incomplete outcome data (attrition bias) | Low risk | No incomplete outcome data. |
|  | Similarity in attrition across trial arms (attrition bias) | Low risk | No participants were lost over follow-up. |
| [85] Gold DT, Shipp KM, Pieper CF, Duncan PW, Martinez S, Lyles KW. Group Treatment Improves Trunk Strength and Psychological Status in Older Women with Vertebral Fractures: Results of a Randomized, Clinical Trial. Journal of the American Geriatrics Society. 2004;52:1471-8. | Jadad score (0-5; N/A for trial protocols) | 3 | Study was described as randomised, with appropriate utilisation of random allocation, and included a description of withdrawals/dropouts. |
|  | Allocation sequence (selection bias) | Low risk | Used a random number generator. |
|  | Allocation concealment (selection bias) | Low risk | “All researchers except the biostatistician were masked to allocation status until a site was enrolled.”  Allocation was concealed. |
|  | Order of randomisation and consent (selection bias) | Unclear | Not enough information to make judgement. |
|  | Order of randomisation and baseline measures (selection bias) | Unclear | Not enough information to make judgement. |
|  | Similarity of baseline outcome measurements across trial arms (selection bias) | Low risk | No evidence of a difference in baseline outcome measures between trial arms (see Table 3). |
|  | Similarity of baseline characteristics across trial arms (selection bias) | Low risk | No evidence of a difference in baseline characteristics between trial arms (see Table 2). |
|  | Blinding of participants and personnel (performance bias) | High risk | Trial was unblinded. |
|  | Blinded outcome assessment (assessor bias) | Low risk | “All personnel involved with subject contacts, data collection, and intervention administration were masked to the intervention status of the sites and to the study hypotheses throughout the trial.”  Outcome assessment was blind. |
|  | Incomplete outcome data (attrition bias) | High risk | Amount of missing data was enough to induce bias in intervention effect estimate. |
|  | Similarity in attrition across trial arms (attrition bias) | High risk | Different proportion of participants lost to follow-up across trial arms (23% and 15%). |
| [86] Han C-K, Ssewamala FM, Wang JS-H. Family economic empowerment and mental health among AIDS-affected children living in AIDS-impacted communities: evidence from a randomised evaluation in southwestern Uganda. Journal of Epidemiology & Community Health. 2013;67:225-30. | Jadad score (0-5; N/A for trial protocols) | 1 | Study was described as randomised. |
|  | Allocation sequence (selection bias) | Unclear | Not enough information to make judgement. |
|  | Allocation concealment (selection bias) | Unclear | Not enough information to make judgement. |
|  | Order of randomisation and consent (selection bias) | Unclear | Not enough information to make judgement. |
|  | Order of randomisation and baseline measures (selection bias) | Unclear | Not enough information to make judgement. |
|  | Similarity of baseline outcome measurements across trial arms (selection bias) | Low risk | No evidence of a difference in baseline outcome measures between trial arms (see Table 1). |
|  | Similarity of baseline characteristics across trial arms (selection bias) | Low risk | No evidence of a difference in baseline characteristics between trial arms (see Table 1). |
|  | Blinding of participants and personnel (performance bias) | High risk | Trial was unblinded. |
|  | Blinded outcome assessment (assessor bias) | Unclear | Not enough information to make judgement. |
|  | Incomplete outcome data (attrition bias) | Unclear | Not enough information to make judgement. |
|  | Similarity in attrition across trial arms (attrition bias) | Unclear | Not enough information to make judgement. |
| [87] Hansson L, Svensson B, Bjorkman T, Bullenkamp J, Lauber C, Martinez-Leal R, et al. What works for whom in a computer-mediated communication intervention in community psychiatry? Moderators of outcome in a cluster randomized trial. Acta Psychiatrica Scandinavica. 2008;118:404-9. | Jadad score (0-5; N/A for trial protocols) | 3 | Study was described as randomised, with appropriate utilisation of random allocation, and included a description of withdrawals/dropouts. |
|  | Allocation sequence (selection bias) | Low risk | Used computerised randomisation (from <https://doi.org/10.1192/bjp.bp.107.036939>). |
|  | Allocation concealment (selection bias) | Unclear | “The process of allocating clinicians to the treatment as usual or intervention groups was by as usual or intervention groups was by numbered, sealed envelopes.” From <https://doi.org/10.1192/bjp.bp.107.036939>. Not clear if envelopes were opaque.  Not enough information to make judgement. |
|  | Order of randomisation and consent (selection bias) | Low risk | The trial flow chart (Figure 2) indicates that consent took place before randomisation.  Randomisation followed consent. |
|  | Order of randomisation and baseline measures (selection bias) | Unclear | Not enough information to make judgement. |
|  | Similarity of baseline outcome measurements across trial arms (selection bias) | Low risk | No evidence of a difference in baseline outcome measures between trial arms (see Table 1). |
|  | Similarity of baseline characteristics across trial arms (selection bias) | Low risk | No evidence of a difference in baseline characteristics between trial arms (see Table 1). |
|  | Blinding of participants and personnel (performance bias) | High risk | Trial was unblinded. |
|  | Blinded outcome assessment (assessor bias) | High risk | “Masking of researchers to the allocation of the patients was attempted for the duration of the study.” However, “masking of interviewers could not be maintained for the majority of patients.”  Outcome assessment was not blind. |
|  | Incomplete outcome data (attrition bias) | High risk | Amount of missing data was enough to induce bias in intervention effect estimate. |
|  | Similarity in attrition across trial arms (attrition bias) | Low risk | Similar proportion of participants lost to follow-up across trial arms (10% and 12%). |
| [88] Harvey AG, Belanger L, Talbot L, Eidelman P, Beaulieu-Bonneau S, Fortier-Brochu E, et al. Comparative Efficacy of Behavior Therapy, Cognitive Therapy, and Cognitive Behavior Therapy for Chronic Insomnia: A Randomized Controlled Trial. Journal of Consulting & Clinical Psychology. 2014;82:670-83. | Jadad score (0-5; N/A for trial protocols) | 2 | Study was described as randomised, and included a description of withdrawals/dropouts. |
|  | Allocation sequence (selection bias) | Unclear | Not enough information to make judgement. |
|  | Allocation concealment (selection bias) | Low risk | “Group allocation concealment was achieved by sequentially numbered, opaque, sealed envelopes opened by the project coordinator at each study site.”  Allocation was concealed. |
|  | Order of randomisation and consent (selection bias) | Low risk | The trial flow chart (Figure 2) indicates that consent took place before randomisation.  Randomisation followed consent. |
|  | Order of randomisation and baseline measures (selection bias) | Unclear | Not enough information to make judgement. |
|  | Similarity of baseline outcome measurements across trial arms (selection bias) | Low risk | “There were no significant differences between groups at baseline on… insomnia duration, and baseline insomnia severity.”  No evidence of a difference in baseline outcome measures between trial arms. |
|  | Similarity of baseline characteristics across trial arms (selection bias) | Low risk | “There were no significant differences between groups at baseline on demographic variables, medical or psychological comorbidity...”  No evidence of a difference in baseline characteristics between trial arms. |
|  | Blinding of participants and personnel (performance bias) | High risk | Trial was unblinded. |
|  | Blinded outcome assessment (assessor bias) | Unclear | Not enough information to make judgement. |
|  | Incomplete outcome data (attrition bias) | High risk | Amount of missing data was enough to induce bias in intervention effect estimate. |
|  | Similarity in attrition across trial arms (attrition bias) | High risk | Different proportion of participants lost to follow-up across trial arms (13%, 14% and 5%). |
| [89] Haukka E, Pehkonen I, Leino-Arjas P, Viikari-Juntura E, Takala E-P, Malmivaara A, et al. Effect of a participatory ergonomics intervention on psychosocial factors at work in a randomised controlled trial. Occupational & Environmental Medicine. 2010;67:170-7. | Jadad score (0-5; N/A for trial protocols) | 1 | Study was described as randomised. |
|  | Allocation sequence (selection bias) | Unclear | Not enough information to make judgement. |
|  | Allocation concealment (selection bias) | Unclear | Not enough information to make judgement. |
|  | Order of randomisation and consent (selection bias) | Unclear | Not enough information to make judgement. |
|  | Order of randomisation and baseline measures (selection bias) | Unclear | Not enough information to make judgement. |
|  | Similarity of baseline outcome measurements across trial arms (selection bias) | Low risk | No evidence of a difference in baseline outcome measures between trial arms (Figure 1). |
|  | Similarity of baseline characteristics across trial arms (selection bias) | Low risk | No evidence of a difference in baseline characteristics between trial arms (Table 1). |
|  | Blinding of participants and personnel (performance bias) | High risk | Trial was unblinded. |
|  | Blinded outcome assessment (assessor bias) | Unclear | Not enough information to make judgement. |
|  | Incomplete outcome data (attrition bias) | Unclear | Not enough information to make judgement. |
|  | Similarity in attrition across trial arms (attrition bias) | Unclear | Not enough information to make judgement. |
| [90] Hayes L, Boyd C, Sewell J. Acceptance and commitment therapy for the treatment of adolescent depression: A pilot study in a psychiatric outpatient setting. Mindfulness. 2011;2:86-94. | Jadad score (0-5; N/A for trial protocols) | 3 | Study was described as randomised, with appropriate utilisation of random allocation, and included a description of withdrawals/dropouts. |
|  | Allocation sequence (selection bias) | Low risk | Used computer-generated random number table. |
|  | Allocation concealment (selection bias) | Unclear | Not enough information to make judgement. |
|  | Order of randomisation and consent (selection bias) | High risk | The trial flow chart (Figure 1) indicates that consent took place after randomisation.  Randomisation preceded consent. |
|  | Order of randomisation and baseline measures (selection bias) | High risk | The trial flow chart (Figure 1) indicates that baseline measures took place after randomisation.  Randomisation preceded baseline measures. |
|  | Similarity of baseline outcome measurements across trial arms (selection bias) | Low risk | “The two groups were equivalent in clinical severity on pretreatment measures (Tables 1 and 3).  No evidence of a difference in baseline outcome measures between trial arms. |
|  | Similarity of baseline characteristics across trial arms (selection bias) | Low risk | The two groups were equivalent in clinical severity on pretreatment measures (Tables 1 and 3).  No evidence of a difference in baseline characteristics between trial arms. |
|  | Blinding of participants and personnel (performance bias) | High risk | Trial was unblinded. |
|  | Blinded outcome assessment (assessor bias) | Unclear | Not enough information to make judgement. |
|  | Incomplete outcome data (attrition bias) | High risk | Amount of missing data was enough to induce bias in intervention effect estimate. |
|  | Similarity in attrition across trial arms (attrition bias) | High risk | Different proportion of participants lost to follow-up across trial arms (64% and 75%). |
| [91] Heirich M, Sieck CJ. Worksite cardiovascular wellness programs as a route to substance abuse prevention. Journal of occupational and environmental medicine. 2000;42:47-56. | Jadad score (0-5; N/A for trial protocols) | 1 | Study was described as randomised. |
|  | Allocation sequence (selection bias) | Unclear | Not enough information to make judgement. |
|  | Allocation concealment (selection bias) | Unclear | Not enough information to make judgement. |
|  | Order of randomisation and consent (selection bias) | Unclear | Not enough information to make judgement. |
|  | Order of randomisation and baseline measures (selection bias) | Unclear | Not enough information to make judgement. |
|  | Similarity of baseline outcome measurements across trial arms (selection bias) | Unclear | Not enough information to make judgement. |
|  | Similarity of baseline characteristics across trial arms (selection bias) | Unclear | Not enough information to make judgement. |
|  | Blinding of participants and personnel (performance bias) | High risk | Trial was unblinded. |
|  | Blinded outcome assessment (assessor bias) | Unclear | Not enough information to make judgement. |
|  | Incomplete outcome data (attrition bias) | Unclear | Not enough information to make judgement. |
|  | Similarity in attrition across trial arms (attrition bias) | Unclear | Not enough information to make judgement. |
| [92] Herz MI, Lamberti JS, Mintz J, Scott R, O'Dell SP, McCartan L, et al. A Program for Relapse Prevention in Schizophrenia: A Controlled Study. Archives of General Psychiatry. 2000;57:277-83. | Jadad score (0-5; N/A for trial protocols) | 2 | Study was described as randomised, with appropriate utilisation of random allocation. |
|  | Allocation sequence (selection bias) | Low risk | Used computerised randomisation. |
|  | Allocation concealment (selection bias) | Unclear | Treatment allocation was written on “cards stored in sealed envelopes”. Does not describe if these were opaque and if they were sequentially numbered.  Not enough information to make judgement. |
|  | Order of randomisation and consent (selection bias) | Low risk | “After a complete description of the study to each subject, written informed consent was obtained. By use of computer-generated cards stored in sealed envelopes, patients who met inclusion criteria and no exclusion criteria were randomly assigned to 1 of 2 treatment groups…”  Randomisation followed consent. |
|  | Order of randomisation and baseline measures (selection bias) | Unclear | Not enough information to make judgement. |
|  | Similarity of baseline outcome measurements across trial arms (selection bias) | Low risk | No evidence of a difference in baseline outcome measures between trial arms (Table 1). |
|  | Similarity of baseline characteristics across trial arms (selection bias) | Low risk | No evidence of a difference in baseline characteristics between trial arms (Table 1). |
|  | Blinding of participants and personnel (performance bias) | High risk | Trial was unblinded. |
|  | Blinded outcome assessment (assessor bias) | Low risk | “Assessments were done by research interviewers who were master's-level mental health professionals, blinded as to patient group assignment…”  Outcome assessment was blind. |
|  | Incomplete outcome data (attrition bias) | Unclear | Not enough information to make judgement. |
|  | Similarity in attrition across trial arms (attrition bias) | Unclear | Not enough information to make judgement. |
| [93] Hjorthoj CR, Fohlmann A, Larsen AM, Gluud C, Arendt M, Nordentoft M. Specialized psychosocial treatment plus treatment as usual (TAU) versus TAU for patients with cannabis use disorder and psychosis: the CapOpus randomized trial. Psychological Medicine. 2013;43:1499-510. | Jadad score (0-5; N/A for trial protocols) | 3 | Study was described as randomised, with appropriate utilisation of random allocation, and included a description of withdrawals/dropouts. |
|  | Allocation sequence (selection bias) | Low risk | Used computerised randomisation. |
|  | Allocation concealment (selection bias) | Low risk | Used central allocation.  Allocation was concealed. |
|  | Order of randomisation and consent (selection bias) | Low risk | “After complete description of the trial to the participants, written informed consent was obtained before randomization.”  Randomisation followed consent. |
|  | Order of randomisation and baseline measures (selection bias) | Low risk | The trial flow chart (Figure 1) indicates that baseline measures took place before randomisation.  Randomisation followed baseline measures. |
|  | Similarity of baseline outcome measurements across trial arms (selection bias) | Low risk | No evidence of a difference in baseline outcome measures between trial arms (Table 1). |
|  | Similarity of baseline characteristics across trial arms (selection bias) | Low risk | No evidence of a difference in baseline characteristics between trial arms (Table 1). |
|  | Blinding of participants and personnel (performance bias) | High risk | Trial was unblinded. |
|  | Blinded outcome assessment (assessor bias) | Low risk | “The outcome assessor was kept blind to allocation by asking participants not to divulge the allocation, staff names, etc.”  Outcome assessment was blind. |
|  | Incomplete outcome data (attrition bias) | High risk | Amount of missing data was enough to induce bias in intervention effect estimate. |
|  | Similarity in attrition across trial arms (attrition bias) | High risk | Different proportion of participants lost to follow-up across trial arms (29% and 39%). |
| [94] Hoffman SL, Hanrahan SJ. Mental Skills for Musicians: Managing Music Performance Anxiety and Enhancing Performance. Sport, Exercise, & Performance Psychology. 2012;1:17-28. | Jadad score (0-5; N/A for trial protocols) | 1 | Study was described as randomised. |
|  | Allocation sequence (selection bias) | Unclear | Not enough information to make judgement. |
|  | Allocation concealment (selection bias) | Unclear | Not enough information to make judgement. |
|  | Order of randomisation and consent (selection bias) | Unclear | Not enough information to make judgement. |
|  | Order of randomisation and baseline measures (selection bias) | Unclear | Not enough information to make judgement. |
|  | Similarity of baseline outcome measurements across trial arms (selection bias) | Unclear | Not enough information to make judgement. |
|  | Similarity of baseline characteristics across trial arms (selection bias) | Unclear | Not enough information to make judgement. |
|  | Blinding of participants and personnel (performance bias) | High risk | Trial was unblined. |
|  | Blinded outcome assessment (assessor bias) | N/A | No outcome assessors (participants completed questionnaires). |
|  | Incomplete outcome data (attrition bias) | Unclear | Not enough information to make judgement. |
|  | Similarity in attrition across trial arms (attrition bias) | Unclear | Not enough information to make judgement. |
| [95] Horowitz JA, Murphy CA, Gregory K, Wojcik J, Pulcini J, Solon L. Nurse Home Visits Improve Maternal/Infant Interaction and Decrease Severity of Postpartum Depression. JOGNN Journal of Obstetric, Gynecologic, & Neonatal Nursing May/June. 2013;42:287-300. | Jadad score (0-5; N/A for trial protocols) | 1 | Study was described as randomised. |
|  | Allocation sequence (selection bias) | Unclear | Not enough information to make judgement. |
|  | Allocation concealment (selection bias) | Unclear | Not enough information to make judgement. |
|  | Order of randomisation and consent (selection bias) | Low risk | “If study criteria were met, and the mother agreed to participate in Phase II after informed consent, then the mother and her infant were randomized…”  Randomisation followed consent. |
|  | Order of randomisation and baseline measures (selection bias) | Unclear | Not enough information to make judgement. |
|  | Similarity of baseline outcome measurements across trial arms (selection bias) | Low risk | “No significant differences were noted between the treatment and control group mothers at baseline on any variable.”  No evidence of a difference in baseline outcome measures between trial arms. |
|  | Similarity of baseline characteristics across trial arms (selection bias) | Low risk | “No significant differences were noted between the treatment and control group mothers at baseline on any variable.”  No evidence of a difference in baseline characteristics between trial arms. |
|  | Blinding of participants and personnel (performance bias) | High risk | Trial was unblinded. |
|  | Blinded outcome assessment (assessor bias) | Unclear | Not enough information to make judgement. |
|  | Incomplete outcome data (attrition bias) | High risk | Inappropriate application of simple imputation (used mean substitution) |
|  | Similarity in attrition across trial arms (attrition bias) | Unclear | Not enough information to make judgement. |
| [96] Horowitz JL, Garber J, Ciesla JA, Young JF, Mufson L. Prevention of Depressive Symptoms in Adolescents: A Randomized Trial of Cognitive-Behavioral and Interpersonal Prevention Programs. Journal of Consulting & Clinical Psychology. 2007;75:693-706. | Jadad score (0-5; N/A for trial protocols) | 1 | Study was described as randomised. |
|  | Allocation sequence (selection bias) | Unclear | Not enough information to make judgement. |
|  | Allocation concealment (selection bias) | Unclear | Not enough information to make judgement. |
|  | Order of randomisation and consent (selection bias) | Low risk | The trial flow chart (Figure 1) indicates that participant enrolment took place before randomisation.  Randomisation followed consent. |
|  | Order of randomisation and baseline measures (selection bias) | Unclear | Not enough information to make judgement. |
|  | Similarity of baseline outcome measurements across trial arms (selection bias) | Low risk | No evidence of a difference in baseline outcome measures between trial arms (see Table 1). |
|  | Similarity of baseline characteristics across trial arms (selection bias) | Low risk | “Students in the three conditions did not differ significantly in age, sex, or race/ethnicity...”  No evidence of a difference in baseline characteristics between trial arms. |
|  | Blinding of participants and personnel (performance bias) | High risk | Trial was unblinded. |
|  | Blinded outcome assessment (assessor bias) | Low risk | “…those conducting the assessments did not know to which condition students had been assigned.”  Outcome assessment was blind. |
|  | Incomplete outcome data (attrition bias) | High risk | Amount of missing data was enough to induce bias in intervention effect estimate. |
|  | Similarity in attrition across trial arms (attrition bias) | Low risk | Similar proportion of participants lost to follow-up across trial arms (21%, 15% and 16%). |
| [97] Horrell L, Goldsmith KA, Tylee AT, Schmidt UH, Murphy CL, Bonin E-M, et al. One-day cognitive-behavioural therapy self-confidence workshops for people with depression: randomised controlled trial. British Journal of Psychiatry. 2014;204:222-33. | Jadad score (0-5; N/A for trial protocols) | 3 | Study was described as randomised, with appropriate utilisation of random allocation, and included a description of withdrawals/dropouts. |
|  | Allocation sequence (selection bias) | Low risk | Used minimisation. |
|  | Allocation concealment (selection bias) | Low risk | Used central allocation (web-based service).  Allocation was concealed. |
|  | Order of randomisation and consent (selection bias) | Low risk | “Eligible participants were randomly assigned to one of the two treatment arms after baseline assessments were completed and consent obtained.”  Randomisation followed consent. |
|  | Order of randomisation and baseline measures (selection bias) | Low risk | “Eligible participants were randomly assigned to one of the two treatment arms after baseline assessments were completed and consent obtained.”  Randomisation followed baseline measures. |
|  | Similarity of baseline outcome measurements across trial arms (selection bias) | Low risk | No evidence of a difference in baseline outcome measures between trial arms (Table 4). |
|  | Similarity of baseline characteristics across trial arms (selection bias) | Low risk | “Baseline characteristics of the participants were comparable  between arms, except for a greater proportion being married in  the control arm, in addition to differences in employment status.”  Little evidence of a difference in baseline characteristics between trial arms. |
|  | Blinding of participants and personnel (performance bias) | High risk | Trial was unblinded. |
|  | Blinded outcome assessment (assessor bias) | N/A | No outcome assessors (participants completed questionnaires). |
|  | Incomplete outcome data (attrition bias) | High risk | Amount of missing data was enough to induce bias in intervention effect estimate. |
|  | Similarity in attrition across trial arms (attrition bias) | High risk | Different proportion of participants lost to follow-up across trial arms (17% and 10%). |
| [98] van den Hout JHC, Vlaeyen JWS, Heuts PHTG, Zijlema JHL, Wijnen JAG. Secondary Prevention of Work-Related Disability in Nonspecific Low Back Pain: Does Problem-Solving Therapy Help? A Randomized Clinical Trial. Clinical Journal of Pain March/April. 2003;19:87-96. | Jadad score (0-5; N/A for trial protocols) | 2 | Study was described as randomised, with appropriate utilisation of random allocation. |
|  | Allocation sequence (selection bias) | Low risk | Used computerised randomisation. |
|  | Allocation concealment (selection bias) | Unclear | “The randomization scheme… was known only to the logistic planner of the rehabilitation center.”  Not enough information to make judgement. |
|  | Order of randomisation and consent (selection bias) | Low risk | “Patients had to sign an informed consent. After being selected, subjects were assigned to one of two treatment conditions...”  Randomisation followed consent. |
|  | Order of randomisation and baseline measures (selection bias) | Unclear | Not enough information to make judgement. |
|  | Similarity of baseline outcome measurements across trial arms (selection bias) | High risk | Some evidence of a difference in baseline outcome measures between trial arms (Table 1). |
|  | Similarity of baseline characteristics across trial arms (selection bias) | Low risk | No evidence of a difference in baseline characteristics between trial arms (Table 1). |
|  | Blinding of participants and personnel (performance bias) | High risk | Trial was unblinded. |
|  | Blinded outcome assessment (assessor bias) | Unclear | Not enough information to make judgement. |
|  | Incomplete outcome data (attrition bias) | Low risk | Amount of missing data was low and balanced across trial arms. |
|  | Similarity in attrition across trial arms (attrition bias) | Low risk | Similar proportion of participants lost to follow-up across trial arms (9% and 11%). |
| [99] Huibers MJH, Beurskens AJHM, Van Schayck CP, Bazelmans E, Metsemakers JFM, Knottnerus JA, et al. Efficacy of cognitive-behavioural therapy by general practitioners for unexplained fatigue among employees: Randomised controlled trial. British Journal of Psychiatry March. 2004;184:240-6. | Jadad score (0-5; N/A for trial protocols) | 3 | Study was described as randomised, with appropriate utilisation of random allocation, and included a description of withdrawals/dropouts. |
|  | Allocation sequence (selection bias) | Low risk | Used computer-generated list of random numbers. |
|  | Allocation concealment (selection bias) | Unclear | “Allocation to group was carried out by the principal investigator (M.J.H.H.) using cards in sealed, opaque envelopes.” Does not say if envelopes were sequentially numbered.  Not enough information to make judgement. |
|  | Order of randomisation and consent (selection bias) | High risk | “After randomisation, patients were informed only about the procedures in the condition they were allocated to, and informed consent was obtained.”  Randomisation followed consent. |
|  | Order of randomisation and baseline measures (selection bias) | Unclear | Not enough information to make judgement. |
|  | Similarity of baseline outcome measurements across trial arms (selection bias) | High risk | “Baseline… clinical characteristics were fairly similar in both groups, except for physical functioning and psychological distress.”  Some evidence of a difference in baseline outcome measures between trial arms. |
|  | Similarity of baseline characteristics across trial arms (selection bias) | Low risk | “Baseline demographic… were fairly similar in both groups.”  No evidence of a difference in baseline characteristics between trial arms. |
|  | Blinding of participants and personnel (performance bias) | High risk | Trial was unblinded. |
|  | Blinded outcome assessment (assessor bias) | N/A | No outcome assessors (participants completed questionnaires). |
|  | Incomplete outcome data (attrition bias) | Low risk | Amount of missing data was low and balanced across trial arms. |
|  | Similarity in attrition across trial arms (attrition bias) | Low risk | Similar proportion of participants lost to follow-up across trial arms (8% and 9%). |
| [100] Jalon GGE, Lennon S, Peoples L, Murphy S, Lowe-Strong A. Energy conservation for fatigue management in multiple sclerosis: a pilot randomized controlled trial. Clinical Rehabilitation. 2013;27:63-74. | Jadad score (0-5; N/A for trial protocols) | 3 | Study was described as randomised, with appropriate utilisation of random allocation, and included a description of withdrawals/dropouts. |
|  | Allocation sequence (selection bias) | Low risk | Used computer-generation random number table. |
|  | Allocation concealment (selection bias) | Unclear | “Group allocation cards were concealed in sealed opaque envelopes to be opened by the treating research therapist, following baseline assessment.” Does not say if envelopes were sequentially numbered.  Not enough information to make judgement. |
|  | Order of randomisation and consent (selection bias) | Low risk | “Patients were given a five-day cooling off period prior to obtaining written consent. They were then randomly allocated to an experimental or a control group.”  Randomisation followed consent. |
|  | Order of randomisation and baseline measures (selection bias) | Low risk | “Group allocation cards were concealed in sealed opaque envelopes to be opened by the treating research therapist, following baseline assessment.”  Randomisation followed baseline measures. |
|  | Similarity of baseline outcome measurements across trial arms (selection bias) | Low risk | No evidence of a difference in baseline outcome measures between trial arms (Table 2). |
|  | Similarity of baseline characteristics across trial arms (selection bias) | Low risk | No evidence of a difference in baseline characteristics between trial arms (Table 1). |
|  | Blinding of participants and personnel (performance bias) | High risk | Trial was unblinded. |
|  | Blinded outcome assessment (assessor bias) | Low risk | “Outcome measures were administered by an independent blinded assessor…”  Outcome assessment was blind. |
|  | Incomplete outcome data (attrition bias) | High risk | Used inappropriate simple imputation (used last observation carried forward) |
|  | Similarity in attrition across trial arms (attrition bias) | Low risk | Similar number of participants lost to follow-up across trial arms (0 and 2). |
| [101] Jellema P, van der Windt DA, van der Horst HE, Twisk JW, Stalman WA, Bouter LM. Should treatment of (sub)acute low back pain be aimed at psychosocial prognostic factors? Cluster randomised clinical trial in general practice. BMJ. 2005;331:84. | Jadad score (0-5; N/A for trial protocols) | 3 | Study was described as randomised, with appropriate utilisation of random allocation, and included a description of withdrawals/dropouts. |
|  | Allocation sequence (selection bias) | Low risk | Used random numbers table. |
|  | Allocation concealment (selection bias) | Unclear | Not enough information to make judgement. |
|  | Order of randomisation and consent (selection bias) | High risk | The trial flow chart (Figure) indicates that participant enrolment took place after randomisation.  Randomisation preceded consent. |
|  | Order of randomisation and baseline measures (selection bias) | High risk | The trial flow chart (Figure) indicates that participant enrolment took place after randomisation.  Randomisation preceded baseline measures. |
|  | Similarity of baseline outcome measurements across trial arms (selection bias) | Low risk | No evidence of a difference in baseline outcome measures between trial arms (Table 1). |
|  | Similarity of baseline characteristics across trial arms (selection bias) | Low risk | No evidence of a difference in baseline characteristics between trial arms (Table 1). |
|  | Blinding of participants and personnel (performance bias) | High risk | Trial was unblinded. |
|  | Blinded outcome assessment (assessor bias) | N/A | No outcome assessors (participants completed questionnaires). |
|  | Incomplete outcome data (attrition bias) | Low risk | Amount of missing data was low and balanced across trial arms. |
|  | Similarity in attrition across trial arms (attrition bias) | Low risk | Similar proportion of participants lost to follow-up across trial arms (8% and 9%). |
| [102] Johnson S, Thornicroft G, Afuwape S, Leese M, Hughes E, Waingarante S, et al. Effects of training community staff in interventions for substance misue in dual diagnosis patients with psychosis (COMO study): Cluster randomised trial. British Journal of Psychiatry November. 2007;191:451-2. | Jadad score (0-5; N/A for trial protocols) | 2 | Study was described as randomised, and included a description of withdrawals/dropouts. |
|  | Allocation sequence (selection bias) | Unclear | Not enough information to make judgement. |
|  | Allocation concealment (selection bias) | Unclear | Not enough information to make judgement. |
|  | Order of randomisation and consent (selection bias) | Unclear | Not enough information to make judgement. |
|  | Order of randomisation and baseline measures (selection bias) | Unclear | Not enough information to make judgement. |
|  | Similarity of baseline outcome measurements across trial arms (selection bias) | Unclear | Not enough information to make judgement. |
|  | Similarity of baseline characteristics across trial arms (selection bias) | Low risk | “Experimental and control groups were similar except for an imbalance for White ethnic group (61% of the control group v. 43% of the intervention.”  Little evidence of a difference in baseline characteristics between trial arms. |
|  | Blinding of participants and personnel (performance bias) | High risk | Trial was unblinded. |
|  | Blinded outcome assessment (assessor bias) | Unclear | Not enough information to make judgement. |
|  | Incomplete outcome data (attrition bias) | High risk | Amount of missing data was enough to induce bias in intervention effect estimate. |
|  | Similarity in attrition across trial arms (attrition bias) | High risk | Different proportion of participants lost to follow-up across trial arms (38% and 26%). |
| [103] Jones C, Skirrow P, Griffiths RD, Humphris GH, Ingleby S, Eddleston J, et al. Rehabilitation after critical illness: A randomized, controlled trial. Critical Care Medicine. 2003;31:2456-61. | Jadad score (0-5; N/A for trial protocols) | 2 | Study was described as randomised, and included a description of withdrawals/dropouts. |
|  | Allocation sequence (selection bias) | Unclear | Not enough information to make judgement. |
|  | Allocation concealment (selection bias) | Unclear | “Patients were assigned to treatment or control groups using a closed envelope technique.” Not clear if envelopes were opaque, sealed and sequentially numbered.  Not enough information to make judgement. |
|  | Order of randomisation and consent (selection bias) | Unclear | Not enough information to make judgement. |
|  | Order of randomisation and baseline measures (selection bias) | Unclear | Not enough information to make judgement. |
|  | Similarity of baseline outcome measurements across trial arms (selection bias) | Low risk | No evidence of a difference in baseline outcome measures between trial arms (Table 1). |
|  | Similarity of baseline characteristics across trial arms (selection bias) | Low risk | No evidence of a difference in baseline characteristics between trial arms (Table 1). |
|  | Blinding of participants and personnel (performance bias) | High risk | Trial was unblinded. |
|  | Blinded outcome assessment (assessor bias) | Low risk | “At follow-up assessment in an outpatient clinic, neither the doctor nor the outcome assessor knew which group the patients were in.”  Outcome assessment was blind. |
|  | Incomplete outcome data (attrition bias) | High risk | Amount of missing data was enough to induce bias in intervention effect estimate. |
|  | Similarity in attrition across trial arms (attrition bias) | High risk | Different proportion of participants lost to follow-up across trial arms (16% and 23%). |
| [104] Joosten EA, de Jong CAJ, de Weert-van Oene GH, Sensky T, van der Staak CPF. Shared Decision-Making Reduces Drug Use and Psychiatric Severity in Substance-Dependent Patients. Psychotherapy & Psychosomatics. 2009;78:245-53. | Jadad score (0-5; N/A for trial protocols) | 3 | Study was described as randomised, with appropriate utilisation of random allocation, and included a description of withdrawals/dropouts. |
|  | Allocation sequence (selection bias) | Low risk | Clusters (clinicians) were allocated using computerised randomisation. |
|  | Allocation concealment (selection bias) | Unclear | Not enough information to make judgement. |
|  | Order of randomisation and consent (selection bias) | High risk | Participants entered the trial after clusters (clinicians) had been assigned to a trial arm.  Randomisation preceded consent. |
|  | Order of randomisation and baseline measures (selection bias) | High risk | Participants entered the trial after clusters (clinicians) had been assigned to a trial arm.  Randomisation preceded baseline measures. |
|  | Similarity of baseline outcome measurements across trial arms (selection bias) | Low risk | No evidence of a difference in baseline outcome measures between trial arms (Table 1). |
|  | Similarity of baseline characteristics across trial arms (selection bias) | Low risk | No evidence of a difference in baseline characteristics between trial arms (Table 1). |
|  | Blinding of participants and personnel (performance bias) | High risk | Trial was unblinded. |
|  | Blinded outcome assessment (assessor bias) | High risk | “Neither study participants nor study staff were blinded to intervention assignment.”  Outcome assessment was not blind. |
|  | Incomplete outcome data (attrition bias) | High risk | Amount of missing data was enough to induce bias in intervention effect estimate. |
|  | Similarity in attrition across trial arms (attrition bias) | High risk | Different proportion of participants lost to follow-up across trial arms (26% and 17%). |
| [105] Jordans MJD, Komproe IH, Tol WA, Kohrt BA, Luitel NP, Macy RD, et al. Evaluation of a classroom-based psychosocial intervention in conflict-affected Nepal: a cluster randomized controlled trial. Journal of Child Psychology & Psychiatry. 2010;51:818-26. | Jadad score (0-5; N/A for trial protocols) | 3 | Study was described as randomised, with appropriate utilisation of random allocation, and included a description of withdrawals/dropouts. |
|  | Allocation sequence (selection bias) | Low risk | Clusters (schools) were allocated using computerised randomisation. |
|  | Allocation concealment (selection bias) | Unclear | Not enough information to make judgement. |
|  | Order of randomisation and consent (selection bias) | High risk | The trial flow chart (Figure) indicates that participant enrolment took place after randomisation.  Randomisation preceded consent. |
|  | Order of randomisation and baseline measures (selection bias) | High risk | The trial flow chart (Figure) indicates that participant enrolment took place after randomisation.  Randomisation preceded baseline measures. |
|  | Similarity of baseline outcome measurements across trial arms (selection bias) | Low risk | No evidence of a difference in baseline outcome measures between trial arms (Table 2). |
|  | Similarity of baseline characteristics across trial arms (selection bias) | Low risk | No evidence of a difference in baseline characteristics between trial arms (Table 1). |
|  | Blinding of participants and personnel (performance bias) | High risk | Trial was unblinded. |
|  | Blinded outcome assessment (assessor bias) | High risk | “It was not possible to blind assessors to treatment status as they needed to visit schools to conduct the interviews.”  Outcome assessment was not blind. |
|  | Incomplete outcome data (attrition bias) | Low risk | Amount of missing data was low and balanced across trial arms. |
|  | Similarity in attrition across trial arms (attrition bias) | Low risk | Similar proportion of participants lost to follow-up across trial arms (1% and 0%). |
| [106] Kaale A, Smith L, Sponheim E. A randomized controlled trial of preschool-based joint attention intervention for children with autism. Journal of Child Psychology & Psychiatry. 2012;53:97-105. | Jadad score (0-5; N/A for trial protocols) | 2 | Study was described as randomised, and included a description of withdrawals/dropouts. |
|  | Allocation sequence (selection bias) | Unclear | Not enough information to make judgement. |
|  | Allocation concealment (selection bias) | Unclear | Not enough information to make judgement. |
|  | Order of randomisation and consent (selection bias) | Low risk | The trial flow chart (Figure) indicates that participant enrolment (including consent) took place before randomisation.  Randomisation followed consent. |
|  | Order of randomisation and baseline measures (selection bias) | Low risk | “Randomization… was undertaken by the first author (AK) after the baseline assessment.”  Randomisation followed baseline measures. |
|  | Similarity of baseline outcome measurements across trial arms (selection bias) | Low risk | No evidence of a difference in baseline outcome measures between trial arms (Table 2). |
|  | Similarity of baseline characteristics across trial arms (selection bias) | Low risk | No evidence of a difference in baseline characteristics between trial arms (Table 1). |
|  | Blinding of participants and personnel (performance bias) | High risk | Trial was unblinded. |
|  | Blinded outcome assessment (assessor bias) | Low risk | “Testers, parents, preschool staff, and counselors were blind to allocation status at baseline. The testers were independent of the research group. All video coding was done by research assistants blind to study purpose, group allocation and testing order.”  Outcome assessment was blind. |
|  | Incomplete outcome data (attrition bias) | Low risk | No incomplete outcome data. |
|  | Similarity in attrition across trial arms (attrition bias) | Low risk | No participants were lost over follow-up. |
| [107] Katon WJ, Lin EH, Von Korff M, Ciechanowski P, Ludman EJ, Young B, et al. Collaborative Care for Patients with Depression and Chronic Illnesses. New England Journal of Medicine. 2010;363:2611-20. | Jadad score (0-5; N/A for trial protocols) | 2 | Study was described as randomised, and included a description of withdrawals/dropouts. |
|  | Allocation sequence (selection bias) | Unclear | Not enough information to make judgement. |
|  | Allocation concealment (selection bias) | Unclear | Not enough information to make judgement. |
|  | Order of randomisation and consent (selection bias) | Low risk | The trial flow chart (Figure) indicates that participant enrolment (including consent) took place before randomisation.  Randomisation followed consent. |
|  | Order of randomisation and baseline measures (selection bias) | Low risk | “After the baseline evaluation, a study nurse contacted patients assigned to the intervention to initiate treatment.”  Randomisation followed baseline measures. |
|  | Similarity of baseline outcome measurements across trial arms (selection bias) | Low risk | No evidence of a difference in baseline outcome measures between trial arms (Table 2). |
|  | Similarity of baseline characteristics across trial arms (selection bias) | Low risk | No evidence of a difference in baseline characteristics between trial arms (Table 1). |
|  | Blinding of participants and personnel (performance bias) | High risk | Trial was unblinded. |
|  | Blinded outcome assessment (assessor bias) | Low risk | This was a “single-blind study” with “research assistants who were unaware of the intervention status implemented study procedures”.  Outcome assessment was blind. |
|  | Incomplete outcome data (attrition bias) | High risk | Amount of missing data was enough to induce bias in intervention effect estimate. |
|  | Similarity in attrition across trial arms (attrition bias) | Low risk | Similar proportion of participants lost to follow-up across trial arms (11% and 15%). |
| [108] Katon WJ, Von Korff M, Lin EH, Simon G, Ludman E, Russo J, et al. The Pathways Study: A Randomized Trial of Collaborative Care in Patients With Diabetes and Depression. Archives of General Psychiatry. 2004;61:1042-9. | Jadad score (0-5; N/A for trial protocols) | 3 | Study was described as randomised, with appropriate utilisation of random allocation, and included a description of withdrawals/dropouts. |
|  | Allocation sequence (selection bias) | Low risk | Used computerised randomisation. |
|  | Allocation concealment (selection bias) | Unclear | Not enough information to make judgement. |
|  | Order of randomisation and consent (selection bias) | Low risk | The trial flow chart (Figure 1) indicates that participant enrolment took place before randomisation.  Randomisation followed consent. |
|  | Order of randomisation and baseline measures (selection bias) | Low risk | The trial flow chart (Figure 1) indicates that participant enrolment took place before randomisation.  Randomisation followed baseline measures. |
|  | Similarity of baseline outcome measurements across trial arms (selection bias) | Low risk | No evidence of a difference in baseline outcome measures between trial arms (Table 1). |
|  | Similarity of baseline characteristics across trial arms (selection bias) | Low risk | No evidence of a difference in baseline characteristics between trial arms (Table 1). |
|  | Blinding of participants and personnel (performance bias) | High risk | Trial was unblinded. |
|  | Blinded outcome assessment (assessor bias) | Low risk | “After randomization, telephone interviews were provided at 3, 6, and 12 months by a telephone survey team who were blinded to intervention status.”  Outcome assessment was blind. |
|  | Incomplete outcome data (attrition bias) | High risk | Amount of missing data was enough to induce bias in intervention effect estimate. |
|  | Similarity in attrition across trial arms (attrition bias) | Low risk | Similar proportion of participants lost to follow-up across trial arms (11% and 14%). |
| [109] Kehoe CE, Havighurst SS, Harley AE. Tuning in to Teens: Improving Parent Emotion Socialization to Reduce Youth Internalizing Difficulties. Social Development. 2014;23:413-31. | Jadad score (0-5; N/A for trial protocols) | 3 | Study was described as randomised, with appropriate utilisation of random allocation, and included a description of withdrawals/dropouts. |
|  | Allocation sequence (selection bias) | Low risk | Used computerised randomisation. |
|  | Allocation concealment (selection bias) | Unclear | Not enough information to make judgement. |
|  | Order of randomisation and consent (selection bias) | High risk | The trial flow chart (Figure 1) indicates that participant enrolment took place after randomisation.  Randomisation preceded consent. |
|  | Order of randomisation and baseline measures (selection bias) | High risk | The trial flow chart (Figure 1) indicates that participant enrolment took place after randomisation.  Randomisation preceded baseline measures. |
|  | Similarity of baseline outcome measurements across trial arms (selection bias) | Low risk | No evidence of a difference in baseline outcome measures between trial arms (Table 2). |
|  | Similarity of baseline characteristics across trial arms (selection bias) | High risk | “Group comparison of demographic characteristics showed significant differences on two variables: youth in the intervention condition were slightly younger, t (223)=−6.24, p<.001 (mean difference in months=−.36), and there were more boys (N=67,55.4%) in the intervention condition compared with the control condition (N=42,40.4%), χ2(1, N=225)=4.48,p=.035, phi=.15.”  Evidence of a difference in baseline characteristics between trial arms. |
|  | Blinding of participants and personnel (performance bias) | High risk | Trial was unblinded. |
|  | Blinded outcome assessment (assessor bias) | N/A | No outcome assessors (participants completed questionnaires). |
|  | Incomplete outcome data (attrition bias) | Low risk | Authors imputed missing scale items using mean of a participant’s non-missing data (if more than 80% of data were non-missing for the scale). Amount of missing data was low and balanced across trial arms. |
|  | Similarity in attrition across trial arms (attrition bias) | Low risk | Similar proportion of participants lost to follow-up across trial arms (8% and 5%). |
| [110] Kellett S, Wilbram M, Davis C, Hardy G. Team consultancy using cognitive analytic therapy: a controlled study in assertive outreach. Journal of Psychiatric & Mental Health Nursing. 2014;21:687-97. | Jadad score (0-5; N/A for trial protocols) | 3 | Study was described as randomised, with appropriate utilisation of random allocation, and included a description of withdrawals/dropouts. |
|  | Allocation sequence (selection bias) | Low risk | Used computerised randomisation. |
|  | Allocation concealment (selection bias) | Unclear | Not enough information to make judgement. |
|  | Order of randomisation and consent (selection bias) | Unclear | Not enough information to make judgement. |
|  | Order of randomisation and baseline measures (selection bias) | Unclear | Not enough information to make judgement. |
|  | Similarity of baseline outcome measurements across trial arms (selection bias) | Low risk | No evidence of a difference in baseline outcome measures between trial arms (Table 1). |
|  | Similarity of baseline characteristics across trial arms (selection bias) | Low risk | “There were no differences apparent between CAC and TAU in terms of age (U = 28.00, Z = 0.10, p = 0.10), years of contact with mental health services (U = 45.00, Z = 0.73, p = 0.73), years of contact with AOT (U = 37.50, Z = 0.35, p = 0.35) or number of admissions (U = 36.50, Z = 0.31, 0.31).”  No evidence of a difference in baseline characteristics between trial arms. |
|  | Blinding of participants and personnel (performance bias) | High risk | Trial was unblinded. |
|  | Blinded outcome assessment (assessor bias) | Unclear | Not enough information to make judgement. |
|  | Incomplete outcome data (attrition bias) | Unclear | Not enough information to make judgement. |
|  | Similarity in attrition across trial arms (attrition bias) | Low risk | Similar number of participants lost to follow-up across trial arms (2 and 1). |
| [111] Khumalo-Sakutukwa G, Morin SF, Fritz K, Charlebois ED, van Rooyen H, Chingono A, et al. Project Accept (HPTN 043): A Community-Based Intervention to Reduce HIV Incidence in Populations at Risk for HIV in Sub-Saharan Africa and Thailand. JAIDS Journal of Acquired Immune Deficiency Syndromes. 2008;49:422-31. | Jadad score (0-5; N/A for trial protocols) | N/A | Article provides results about adherence to intervention but nothing about outcomes. |
|  | Allocation sequence (selection bias) | Unclear | Not enough information to make judgement. |
|  | Allocation concealment (selection bias) | Unclear | Not enough information to make judgement. |
|  | Order of randomisation and consent (selection bias) | Unclear | Not enough information to make judgement. |
|  | Order of randomisation and baseline measures (selection bias) | Unclear | Not enough information to make judgement. |
|  | Similarity of baseline outcome measurements across trial arms (selection bias) | Unclear | Not enough information to make judgement. |
|  | Similarity of baseline characteristics across trial arms (selection bias) | Unclear | Not enough information to make judgement. |
|  | Blinding of participants and personnel (performance bias) | High risk | Trial was unblinded. |
|  | Blinded outcome assessment (assessor bias) | Unclear | Not enough information to make judgement. |
|  | Incomplete outcome data (attrition bias) | Unclear | Not enough information to make judgement. |
|  | Similarity in attrition across trial arms (attrition bias) | Unclear | Not enough information to make judgement. |
| [112] Kikkenborg Berg S, Stoier L, Moons P, Zwisler AD, Winkel P, Ulrich Pedersen P. Emotions and health: findings from a randomized clinical trial on psychoeducational nursing to patients with implantable cardioverter defibrillator. The Journal of cardiovascular nursing. 2015;30:197-204. | Jadad score (0-5; N/A for trial protocols) | 2 | Study was described as randomised, and included a description of withdrawals/dropouts. |
|  | Allocation sequence (selection bias) | Unclear | Not enough information to make judgement. |
|  | Allocation concealment (selection bias) | Unclear | Not enough information to make judgement. |
|  | Order of randomisation and consent (selection bias) | Low risk | The trial flow chart (Figure) indicates that participant consent took place before randomisation.  Randomisation followed consent. |
|  | Order of randomisation and baseline measures (selection bias) | Unclear | Not enough information to make judgement. |
|  | Similarity of baseline outcome measurements across trial arms (selection bias) | Low risk | No evidence of a difference in baseline outcome measures between trial arms (Table 3). |
|  | Similarity of baseline characteristics across trial arms (selection bias) | Low risk | No evidence of a difference in baseline characteristics between trial arms (Table 2). |
|  | Blinding of participants and personnel (performance bias) | High risk | Trial was unblinded. |
|  | Blinded outcome assessment (assessor bias) | Unclear | Not enough information to make judgement. |
|  | Incomplete outcome data (attrition bias) | High risk | Amount of missing data was enough to induce bias in intervention effect estimate. |
|  | Similarity in attrition across trial arms (attrition bias) | High risk | Different proportion of participants lost to follow-up across trial arms (13% and 19%). |
| [113] Klein RG, Abikoff H, Hechtman L, Weiss G. Design and Rationale of Controlled Study of Long-Term Methylphenidate and Multimodal Psychosocial Treatment in Children With ADHD. Journal of the American Academy of Child & Adolescent Psychiatry. 2004;43:792-801. | Jadad score (0-5; N/A for trial protocols) | N/A | Article provides results about rationale, methodology and characteristics of a trial, but nothing about outcomes. |
|  | Allocation sequence (selection bias) | Unclear | Not enough information to make judgement. |
|  | Allocation concealment (selection bias) | Unclear | Not enough information to make judgement. |
|  | Order of randomisation and consent (selection bias) | Low risk | Informed consent is described as a criterion for study participation.  Randomisation followed consent. |
|  | Order of randomisation and baseline measures (selection bias) | Unclear | Not enough information to make judgement. |
|  | Similarity of baseline outcome measurements across trial arms (selection bias) | Low risk | “Except for socioeconomic status, there were no group differences.”  Evidence of a difference in baseline outcome measures between trial arms. |
|  | Similarity of baseline characteristics across trial arms (selection bias) | High risk | “A significant group difference was found for socioeconomic status between M alone and M + ACT (Tukey honestly significant difference, p< .05).”  Evidence of a difference in baseline characteristics between trial arms. |
|  | Blinding of participants and personnel (performance bias) | High risk | Trial was unblinded. |
|  | Blinded outcome assessment (assessor bias) | Unclear | Not enough information to make judgement. |
|  | Incomplete outcome data (attrition bias) | Unclear | Not enough information to make judgement. |
|  | Similarity in attrition across trial arms (attrition bias) | N/A | No follow-up described. |
| [114] Kohler L, Meinke-Franze C, Hein J, Fendrich K, Heymann R, Thyrian JR, et al. Does an Interdisciplinary Network Improve Dementia Care? Results from the IDemUck-Study. Current Alzheimer Research. 2014;11:538-48. | Jadad score (0-5; N/A for trial protocols) | 2 | Study was described as randomised, and included a description of withdrawals/dropouts. |
|  | Allocation sequence (selection bias) | Unclear | Not enough information to make judgement. |
|  | Allocation concealment (selection bias) | Unclear | Not enough information to make judgement. |
|  | Order of randomisation and consent (selection bias) | Unclear | Not enough information to make judgement. |
|  | Order of randomisation and baseline measures (selection bias) | Unclear | Not enough information to make judgement. |
|  | Similarity of baseline outcome measurements across trial arms (selection bias) | Low risk | No evidence of a difference in baseline outcome measures between trial arms (Table 2). |
|  | Similarity of baseline characteristics across trial arms (selection bias) | Low risk | No evidence of a difference in baseline characteristics between trial arms (Table 1). |
|  | Blinding of participants and personnel (performance bias) | High risk | Trial was unblinded. |
|  | Blinded outcome assessment (assessor bias) | Unclear | Not enough information to make judgement. |
|  | Incomplete outcome data (attrition bias) | High risk | Amount of missing data was enough to induce bias in intervention effect estimate. |
|  | Similarity in attrition across trial arms (attrition bias) | High risk | Different proportion of participants lost to follow-up across trial arms (9% and 18%). |
| [115] Kronish IM, Rieckmann N, Burg MM, Edmondson D, Schwartz JE, Davidson KW. The effect of enhanced depression care on adherence to risk-reducing behaviors after acute coronary syndromes: Findings from the COPES trial. American Heart Journal. 2012;164:524-9. | Jadad score (0-5; N/A for trial protocols) | 1 | Study was described as randomised. |
|  | Allocation sequence (selection bias) | Unclear | Not enough information to make judgement. |
|  | Allocation concealment (selection bias) | Unclear | Not enough information to make judgement. |
|  | Order of randomisation and consent (selection bias) | Low risk | “Eligible patients who consent to the RCT at the 3-month visit are randomized to either the INT or UCC condition.” From <https://doi.org/10.1016/j.cct.2007.08.003>.  Randomisation followed consent. |
|  | Order of randomisation and baseline measures (selection bias) | Low risk | Participants were assessed at baseline and then three months later. Following this they were then randomly allocated to one treatment.  Randomisation followed baseline measures. |
|  | Similarity of baseline outcome measurements across trial arms (selection bias) | Unclear | Not enough information to make judgement. |
|  | Similarity of baseline characteristics across trial arms (selection bias) | Low risk | No evidence of a difference in baseline characteristics between trial arms (Table 1). |
|  | Blinding of participants and personnel (performance bias) | High risk | Trial was unblinded. |
|  | Blinded outcome assessment (assessor bias) | Low risk | “Throughout the process of randomization, selection of treatment element, and subsequent treatment participation, full effort is put forth to ensure that those staff responsible for the collection of treatment outcome data are kept blinded to patient treatment assignment.” From <https://doi.org/10.1016/j.cct.2007.08.003>.  Outcome assessment was blind. |
|  | Incomplete outcome data (attrition bias) | Unclear | Not enough information to make judgement. |
|  | Similarity in attrition across trial arms (attrition bias) | Unclear | Not enough information to make judgement. |
| [116] Kypri K, McCambridge J, Vater T, Bowe SJ, Saunders JB, Cunningham JA, et al. Web-based alcohol intervention for Maori university students: double-blind, multi-site randomized controlled trial. Addiction. 2013;108:331-8. | Jadad score (0-5; N/A for trial protocols) | 3 | Study was described as randomised, with appropriate utilisation of random allocation, and included a description of withdrawals/dropouts. |
|  | Allocation sequence (selection bias) | Low risk | Used computerised randomisation. |
|  | Allocation concealment (selection bias) | Low risk | Randomisation was done remotely (by web server). “…randomization and all other study procedures were fully automated and thus could not be subverted.”  Allocation was concealed. |
|  | Order of randomisation and consent (selection bias) | Low risk | The trial flow chart (Figure 1) indicates that participant consent took place before randomisation.  Randomisation followed consent. |
|  | Order of randomisation and baseline measures (selection bias) | Low risk | The trial flow chart (Figure 1) indicates that participant enrolment took place before randomisation. In addition, “Students were sent an e-mail containing a hyperlink to a web questionnaire and were informed that: ‘the main focus of this study is student alcohol use over time and its consequences’.” Randomisation followed this.  Randomisation followed baseline measures. |
|  | Similarity of baseline outcome measurements across trial arms (selection bias) | Low risk | No evidence of a difference in baseline outcome measures between trial arms (Table 2). |
|  | Similarity of baseline characteristics across trial arms (selection bias) | Low risk | No evidence of a difference in baseline characteristics between trial arms (Table 2). |
|  | Blinding of participants and personnel (performance bias) | High risk | Trial was unblinded. |
|  | Blinded outcome assessment (assessor bias) | N/A | No outcome assessors (participants completed questionnaires). |
|  | Incomplete outcome data (attrition bias) | High risk | Amount of missing data was enough to induce bias in intervention effect estimate. |
|  | Similarity in attrition across trial arms (attrition bias) | Low risk | Similar proportion of participants lost to follow-up across trial arms (20% and 22%). |
| [117] Lam CLK, Fong DYT, Chin W-Y, Lee PWH, Lam ETP, Lo YYC. Brief problem-solving treatment in primary care (pst-pc) was not more effective than placebo for elderly patients screened positive of psychological problems. International Journal of Geriatric Psychiatry. 2010;25:968-80. | Jadad score (0-5; N/A for trial protocols) | 3 | Study was described as randomised, with appropriate utilisation of random allocation, and included a description of withdrawals/dropouts. |
|  | Allocation sequence (selection bias) | Low risk | Used computerised randomisation. |
|  | Allocation concealment (selection bias) | Unclear | Not enough information to make judgement. |
|  | Order of randomisation and consent (selection bias) | Low risk | “Subjects with positive HADS scores (AS ≥3 or DS ≥6) were invited by telephone to participate in the randomised control trial (RCT). Those who agreed were randomised into either the intervention (PST-PC) group or the placebo (video) group.”  Randomisation followed consent. |
|  | Order of randomisation and baseline measures (selection bias) | Unclear | Not enough information to make judgement. |
|  | Similarity of baseline outcome measurements across trial arms (selection bias) | Low risk | No evidence of a difference in baseline outcome measures between trial arms (Table 1). |
|  | Similarity of baseline characteristics across trial arms (selection bias) | Low risk | No evidence of a difference in baseline characteristics between trial arms (Table 1). |
|  | Blinding of participants and personnel (performance bias) | High risk | Trial was unblinded. |
|  | Blinded outcome assessment (assessor bias) | Low risk | “The same survey instruments (excluding socio-demography) were administered at each follow up by a trained interviewer who was blinded to the screening result, group allocation or treatment of the subject.”  Outcome assessment was blind. |
|  | Incomplete outcome data (attrition bias) | High risk | Used inappropriate simple imputation (used last observation carried forward). |
|  | Similarity in attrition across trial arms (attrition bias) | Low risk | Similar proportion of participants lost to follow-up across trial arms (31% and 29%). |
| [118] Lamers F, Jonkers CCM, Bosma H, Kempen GIJM, Meijer JAMJ, Penninx BWJH, et al. A Minimal Psychological Intervention in Chronically Ill Elderly Patients with Depression: A Randomized Trial. Psychotherapy & Psychosomatics. 2010;79:217-26. | Jadad score (0-5; N/A for trial protocols) | 3 | Study was described as randomised, with appropriate utilisation of random allocation, and included a description of withdrawals/dropouts. |
|  | Allocation sequence (selection bias) | Low risk | Used computerised random number generator. |
|  | Allocation concealment (selection bias) | Low risk | Used central allocation (web-based service).  Allocation was concealed. |
|  | Order of randomisation and consent (selection bias) | Low risk | “After signing an informed consent form and completing a baseline questionnaire, patients were randomly allocated to the MPI or to care as usual.”  Randomisation followed consent. |
|  | Order of randomisation and baseline measures (selection bias) | Low risk | “After signing an informed consent form and completing a baseline questionnaire, patients were randomly allocated to the MPI or to care as usual.”  Randomisation followed baseline measures. |
|  | Similarity of baseline outcome measurements across trial arms (selection bias) | Low risk | No evidence of a difference in baseline outcome measures between trial arms (Table 2). |
|  | Similarity of baseline characteristics across trial arms (selection bias) | Low risk | No evidence of a difference in baseline characteristics between trial arms (Table 2). |
|  | Blinding of participants and personnel (performance bias) | High risk | Trial was unblinded. |
|  | Blinded outcome assessment (assessor bias) | N/A | No outcome assessors (participants completed questionnaires). |
|  | Incomplete outcome data (attrition bias) | High risk | Amount of missing data was enough to induce bias in intervention effect estimate. |
|  | Similarity in attrition across trial arms (attrition bias) | High risk | Different proportion of participants lost to follow-up across trial arms (37% and 29%). |
| [119] Lanken PN, Novack DH, Daetwyler C, Gallop R, Landis JR, Lapin J, et al. Efficacy of an Internet-Based Learning Module and Small-Group Debriefing on Trainees' Attitudes and Communication Skills Toward Patients With Substance Use Disorders: Results of a Cluster Randomized Controlled Trial. Academic Medicine. 2015;90:345-54. | Jadad score (0-5; N/A for trial protocols) | 3 | Study was described as randomised, with appropriate utilisation of random allocation, and included a description of withdrawals/dropouts. |
|  | Allocation sequence (selection bias) | Low risk | Used computerised randomisation. |
|  | Allocation concealment (selection bias) | Unclear | Not enough information to make judgement. |
|  | Order of randomisation and consent (selection bias) | Low risk | “The protocol’s order of events for residents was (1) informed consent; (2) presurvey (in paper format) (see Supplemental Digital Appendix 1); (3) real-time online interview of a standardized patient (SP) (Case 1); (4) disclosure of randomization result for given cluster.”  Randomisation in effect followed consent. |
|  | Order of randomisation and baseline measures (selection bias) | Low risk | “The protocol’s order of events for residents was (1) informed consent; (2) presurvey (in paper format) (see Supplemental Digital Appendix 1); (3) real-time online interview of a standardized patient (SP) (Case 1); (4) disclosure of randomization result for given cluster.”  Randomisation in effect followed baseline measures. |
|  | Similarity of baseline outcome measurements across trial arms (selection bias) | Low risk | “We found no significant differences between the I- and C-groups in the mean scores for any of the eight outcome scales on the presurvey (baseline survey) (see [Table 1](javascript:void(0))).”  No evidence of a difference in baseline outcome measures between trial arms. |
|  | Similarity of baseline characteristics across trial arms (selection bias) | Low risk | “We found no statistically significant differences between the I- and C-groups in demographic and other baseline characteristics.”  No evidence of a difference in baseline characteristics between trial arms. |
|  | Blinding of participants and personnel (performance bias) | High risk | Trial was unblinded. |
|  | Blinded outcome assessment (assessor bias) | Unclear | Not enough information to make judgement. |
|  | Incomplete outcome data (attrition bias) | Low risk | Amount of missing data was low and balanced across trial arms. |
|  | Similarity in attrition across trial arms (attrition bias) | Low risk | Similar proportion of participants lost to follow-up across trial arms (3% and 3%). |
| [120] Lash SJ, Stephens RS, Burden JL, Grambow SC, DeMarce JM, Jones ME, et al. Contracting, Prompting, and Reinforcing Substance Use Disorder Continuing Care: A Randomized Clinical Trial. Psychology of Addictive Behaviors. 2007;21:387-97. | Jadad score (0-5; N/A for trial protocols) | 3 | Study was described as randomised, with appropriate utilisation of random allocation, and included a description of withdrawals/dropouts. |
|  | Allocation sequence (selection bias) | Low risk | Used computerised randomisation. |
|  | Allocation concealment (selection bias) | Unclear | Not enough information to make judgement. |
|  | Order of randomisation and consent (selection bias) | Low risk | “…the principal investigator, Steven J. Lash, met with each patient to determine his/her study eligibility and obtain his/her informed consent to participate according to the research protocol approved by Salem VAMC’s Internal Review Board. Participants completed baseline assessments on the 24th day of residential care, prior to random assignment to STX or CPR.”  Randomisation followed consent. |
|  | Order of randomisation and baseline measures (selection bias) | Low risk | “…the principal investigator, Steven J. Lash, met with each patient to determine his/her study eligibility and obtain his/her informed consent to participate according to the research protocol approved by Salem VAMC’s Internal Review Board. Participants completed baseline assessments on the 24th day of residential care, prior to random assignment to STX or CPR.”  Randomisation followed baseline measures. |
|  | Similarity of baseline outcome measurements across trial arms (selection bias) | Low risk | No evidence of a difference in baseline outcome measures between trial arms (Table 3). |
|  | Similarity of baseline characteristics across trial arms (selection bias) | Unclear | Not enough information to make judgement. |
|  | Blinding of participants and personnel (performance bias) | High risk | Trial was unblinded. |
|  | Blinded outcome assessment (assessor bias) | Low risk | “All assessment instruments were administered by four graduate students in clinical psychology who were blind to participants’ treatment condition.”  Outcome assessment was blind. |
|  | Incomplete outcome data (attrition bias) | High risk | Amount of missing data was enough to induce bias in intervention effect estimate. |
|  | Similarity in attrition across trial arms (attrition bias) | Low risk | Similar proportion of participants lost to follow-up across trial arms (22% and 21%). |
| [121] Lee KA, Gay CL. Can modifications to the bedroom environment improve the sleep of new parents? Two randomized controlled trials. Research in Nursing & Health. 2011;34:7-19. | Jadad score (0-5; N/A for trial protocols) | 1 | Study was described as randomisation. |
|  | Allocation sequence (selection bias) | Unclear | Not enough information to make judgement. |
|  | Allocation concealment (selection bias) | Unclear | Not enough information to make judgement. |
|  | Order of randomisation and consent (selection bias) | Low risk | The trial flow chart (Figure 1) indicates that participant consent took place before randomisation.  Randomisation followed consent. |
|  | Order of randomisation and baseline measures (selection bias) | Unclear | Not enough information to make judgement. |
|  | Similarity of baseline outcome measurements across trial arms (selection bias) | Unclear | Not enough information to make judgement. |
|  | Similarity of baseline characteristics across trial arms (selection bias) | Unclear | Not enough information to make judgement. |
|  | Blinding of participants and personnel (performance bias) | High risk | Trial was unblinded. |
|  | Blinded outcome assessment (assessor bias) | Low risk | “Trained research assistants blinded to intervention group analyzed the actigraph data [main outcome data] using the autoscoring Cole–Kripke algorithm program available in Action4 software.”  Outcome assessment was blind. |
|  | Incomplete outcome data (attrition bias) | High risk | Amount of missing data was enough to induce bias in intervention effect estimate. |
|  | Similarity in attrition across trial arms (attrition bias) | High risk | Different proportion of participants lost to follow-up across trial arms (3% and 0% in sample 1; 22% and 11% in sample 2). |
| [122] Leeuw M, Goossens ME, van Breukelen GJ, de Jong JR, Heuts PH, Smeets RJ, et al. Exposure in vivo versus operant graded activity in chronic low back pain patients: Results of a randomized controlled trial. Pain. 2008;138:192-207. | Jadad score (0-5; N/A for trial protocols) | 3 | Study was described as randomised, with appropriate utilisation of random allocation, and included a description of withdrawals/dropouts. |
|  | Allocation sequence (selection bias) | Low risk | Used computerised randomisation. |
|  | Allocation concealment (selection bias) | Unclear | “Because of the complexity of the randomization schedule, forthcoming assignments could never be predicted. The randomization schedule was only accessible for the research assistant performing the randomization. After the second pre-treatment measurement, patients received a sealed envelope from the research assistant containing a sheet of coloured paper indicating treatment assignment, which they opened together with the psychologist during the intake.” Not clear if envelope was opaque and whether it was sequentially numbered (or whether this was necessary).  Not enough information to make judgement. |
|  | Order of randomisation and consent (selection bias) | Low risk | The trial flow chart (Figure 1) indicates that participant consent took place before randomisation.  Randomisation followed consent. |
|  | Order of randomisation and baseline measures (selection bias) | Unclear | Not enough information to make judgement. |
|  | Similarity of baseline outcome measurements across trial arms (selection bias) | Low risk | No evidence of a difference in baseline outcome measures between trial arms (Table 1). |
|  | Similarity of baseline characteristics across trial arms (selection bias) | Low risk | No evidence of a difference in baseline characteristics between trial arms (Table 1). |
|  | Blinding of participants and personnel (performance bias) | High risk | Trial was unblinded. |
|  | Blinded outcome assessment (assessor bias) | N/A | No outcome assessors (participants completed questionnaires). |
|  | Incomplete outcome data (attrition bias) | High risk | Amount of missing data was enough to induce bias in intervention effect estimate. |
|  | Similarity in attrition across trial arms (attrition bias) | High risk | Different proportion of participants lost to follow-up across trial arms (10% and 19%). |
| [123] L'Engle KL, Mwarogo P, Kingola N, Sinkele W, Weiner DH. A Randomized Controlled Trial of a Brief Intervention to Reduce Alcohol Use Among Female Sex Workers in Mombasa, Kenya. JAIDS Journal of Acquired Immune Deficiency Syndromes. 2014;67:446-53. | Jadad score (0-5; N/A for trial protocols) | 3 | Study was described as randomised, with appropriate utilisation of random allocation, and included a description of withdrawals/dropouts. |
|  | Allocation sequence (selection bias) | Low risk | Used computerised randomisation. |
|  | Allocation concealment (selection bias) | Unclear | “A statistician not otherwise involved in the study generated the randomization sequences using the random function RANUNI in SAS [SAS Institute, Cary, NC] and produced written assignments sealed in individual tamper-evident opaque envelopes.” Not clear if they were sequentially numbered.  Not enough information to make judgement. |
|  | Order of randomisation and consent (selection bias) | Low risk | “The envelopes were fully protected until the site coordinator confirmed the prospective participant's eligibility, obtained written informed consent, and collected all baseline data.”  Randomisation followed consent. |
|  | Order of randomisation and baseline measures (selection bias) | Low risk | “The envelopes were fully protected until the site coordinator confirmed the prospective participant's eligibility, obtained written informed consent, and collected all baseline data.”  Randomisation followed baseline measures. |
|  | Similarity of baseline outcome measurements across trial arms (selection bias) | Low risk | No evidence of a difference in baseline outcome measures between trial arms (Table 2). |
|  | Similarity of baseline characteristics across trial arms (selection bias) | Low risk | No evidence of a difference in baseline characteristics between trial arms (Table 1). |
|  | Blinding of participants and personnel (performance bias) | High risk | Trial was unblinded. |
|  | Blinded outcome assessment (assessor bias) | Unclear | Not enough information to make judgement. |
|  | Incomplete outcome data (attrition bias) | Low risk | Amount of missing data was low and balanced across trial arms. |
|  | Similarity in attrition across trial arms (attrition bias) | Low risk | Similar proportion of participants lost to follow-up across trial arms (9% and 7%). |
| [124] Leon L, Jover JA, Candelas G, Lajas C, Vadillo C, Blanco M, et al. Effectiveness of an early cognitive-behavioral treatment in patients with work disability due to musculoskeletal disorders. Arthritis & Rheumatism. 2009;61:996-1003. | Jadad score (0-5; N/A for trial protocols) | 2 | Study was described as randomised, with appropriate utilisation of random allocation. |
|  | Allocation sequence (selection bias) | Low risk | Used computerised randomisation. |
|  | Allocation concealment (selection bias) | Unclear | Participants were “allocated to the 2 specialized care centers by means of sealed envelopes.” Unclear if envelopes were opaque and sequentially numbered.  Not enough information to make judgement. |
|  | Order of randomisation and consent (selection bias) | Low risk | “All eligible patients were invited to participate in the study, and those who gave their verbal consent were randomly assigned to either the intervention group.”  Randomisation followed consent. |
|  | Order of randomisation and baseline measures (selection bias) | Unclear | Not enough information to make judgement. |
|  | Similarity of baseline outcome measurements across trial arms (selection bias) | N/A | Outcomes not measured at baseline. |
|  | Similarity of baseline characteristics across trial arms (selection bias) | Low risk | No evidence of a difference in baseline characteristics between trial arms (Table 1). |
|  | Blinding of participants and personnel (performance bias) | High risk | Trial was unblinded. |
|  | Blinded outcome assessment (assessor bias) | Unclear | Not enough information to make judgement. |
|  | Incomplete outcome data (attrition bias) | Unclear | Not enough information to make judgement. |
|  | Similarity in attrition across trial arms (attrition bias) | Unclear | Not enough information to make judgement. |
| [125] Lewis FM, Brandt PA, Cochrane BB, Griffith KA, Grant M, Haase JE, et al. The Enhancing Connections Program: A Six-State Randomized Clinical Trial of a Cancer Parenting Program. Journal of Consulting & Clinical Psychology. 2015;83:12-23. | Jadad score (0-5; N/A for trial protocols) | 2 | Study was described as randomised, and included a description of withdrawals/dropouts. |
|  | Allocation sequence (selection bias) | Unclear | Not enough information to make judgement. |
|  | Allocation concealment (selection bias) | Unclear | Not enough information to make judgement. |
|  | Order of randomisation and consent (selection bias) | Low risk | “After consenting and obtaining baseline measures, study participants were randomized into experimental or control groups.”  Randomisation followed consent. |
|  | Order of randomisation and baseline measures (selection bias) | Low risk | “After consenting and obtaining baseline measures, study participants were randomized into experimental or control groups.”  Randomisation followed baseline measures. |
|  | Similarity of baseline outcome measurements across trial arms (selection bias) | Low risk | “There were no significant differences between groups between baseline and 2 months or between 2 and 12 months on demographic, treatment, or outcome variables.”  No evidence of a difference in baseline outcome measures between trial arms (also Tables 3 and 4). |
|  | Similarity of baseline characteristics across trial arms (selection bias) | Low risk | “There were no significant differences between groups between baseline and 2 months or between 2 and 12 months on demographic, treatment, or outcome variables.”  No evidence of a difference in baseline characteristics between trial arms. |
|  | Blinding of participants and personnel (performance bias) | High risk | Trial was unblinded. |
|  | Blinded outcome assessment (assessor bias) | Low risk | “Members of the data collection team were masked on the randomization status of each study participant for the duration of the trial.”  Outcome assessment was blind. |
|  | Incomplete outcome data (attrition bias) | High risk | Amount of missing data was enough to induce bias in intervention effect estimate. |
|  | Similarity in attrition across trial arms (attrition bias) | Low risk | Similar proportion of participants lost to follow-up across trial arms (43% and 41%). |
| [126] Li L, Hien NT, Lin C, Tuan NA, Tuan LA, Farmer SC, et al. An Intervention to Improve Mental Health and Family Well-Being of Injecting Drug Users and Family Members in Vietnam. Psychology of Addictive Behaviors. 2014;28:607-13. | Jadad score (0-5; N/A for trial protocols) | 2 | Study was described as randomised, and included a description of withdrawals/dropouts. |
|  | Allocation sequence (selection bias) | Unclear | Not enough information to make judgement. |
|  | Allocation concealment (selection bias) | Unclear | Not enough information to make judgement. |
|  | Order of randomisation and consent (selection bias) | Unclear | Not enough information to make judgement. |
|  | Order of randomisation and baseline measures (selection bias) | Unclear | “After the baseline assessment, the two communes in each pair were randomized to either an intervention or a standard care condition.” However, Figure 1 implies the opposite.  Not enough information to make judgement. |
|  | Similarity of baseline outcome measurements across trial arms (selection bias) | Low risk | No evidence of a difference in baseline outcome measures between trial arms (Figure 2). |
|  | Similarity of baseline characteristics across trial arms (selection bias) | High risk | “No baseline differences were observed for gender, age, marital status, or employment status of IDUs. Fewer IDUs (15.0%) in the standard care group had 9 years or less education than IDUs in the intervention group (46.5%, p= .0020).”  No evidence of a difference in baseline characteristics between trial arms (also see Table 1). |
|  | Blinding of participants and personnel (performance bias) | High risk | Trial was unblinded. |
|  | Blinded outcome assessment (assessor bias) | Unclear | Not enough information to make judgement. |
|  | Incomplete outcome data (attrition bias) | Low risk | Amount of missing data was low and balanced across trial arms. |
|  | Similarity in attrition across trial arms (attrition bias) | Low risk | Similar proportion of participants lost to follow-up across trial arms (1% and 0%). |
| [127] Limm H, Gundel H, Heinmuller M, Marten-Mittag B, Nater UM, Siegrist J, et al. Stress management interventions in the workplace improve stress reactivity: a randomised controlled trial. Occupational & Environmental Medicine. 2011;68:126-33. | Jadad score (0-5; N/A for trial protocols) | 2 | Study was described as randomised, and included a description of withdrawals/dropouts. |
|  | Allocation sequence (selection bias) | Unclear | Not enough information to make judgement. |
|  | Allocation concealment (selection bias) | Unclear | Not enough information to make judgement. |
|  | Order of randomisation and consent (selection bias) | Low risk | The trial flow chart (Figure 1) indicates that participant consent took place before randomisation.  Randomisation followed consent. |
|  | Order of randomisation and baseline measures (selection bias) | Low risk | “All participants were invited to a 1.5 h medical and psychological examination by an experienced team consisting of a psychologist (HL) and a physician (MH). Written informed consent was obtained. All volunteers were required to complete a battery of questionnaires, participate in a basic physical examination with blood sampling and collect saliva samples the next working day. This initial health check included feedback to each participant a few days later… Eligible participants were randomised after the initial evaluation to one of two groups:  Randomisation followed baseline measures. |
|  | Similarity of baseline outcome measurements across trial arms (selection bias) | Low risk | No evidence of a difference in baseline outcome measures between trial arms (Table 2). |
|  | Similarity of baseline characteristics across trial arms (selection bias) | Low risk | No evidence of a difference in baseline characteristics between trial arms (Table 1). |
|  | Blinding of participants and personnel (performance bias) | High risk | Trial was unblinded. |
|  | Blinded outcome assessment (assessor bias) | Unclear | Not enough information to make judgement. |
|  | Incomplete outcome data (attrition bias) | High risk | Amount of missing data was enough to induce bias in intervention effect estimate. |
|  | Similarity in attrition across trial arms (attrition bias) | Low risk | Similar proportion of participants lost to follow-up across trial arms (14% and 9%). |
| [128] Linares LO, Montalto D, Li M, Oza VS. A Promising Parenting Intervention in Foster Care. Journal of Consulting & Clinical Psychology. 2006;74:32-41. | Jadad score (0-5; N/A for trial protocols) | 2 | Study was described as randomised, and included a description of withdrawals/dropouts. |
|  | Allocation sequence (selection bias) | Unclear | Not enough information to make judgement. |
|  | Allocation concealment (selection bias) | Unclear | Not enough information to make judgement. |
|  | Order of randomisation and consent (selection bias) | Low risk | The trial flow chart (Figure 1) indicates that participant consent took place before randomisation.  Randomisation followed consent. |
|  | Order of randomisation and baseline measures (selection bias) | Unclear | Not enough information to make judgement. |
|  | Similarity of baseline outcome measurements across trial arms (selection bias) | Low risk | “No statistically significant differences at baseline by study condition were found for… study outcomes.”  No evidence of a difference in baseline outcome measures between trial arms. |
|  | Similarity of baseline characteristics across trial arms (selection bias) | Unclear | Not enough information to make judgement. |
|  | Blinding of participants and personnel (performance bias) | High risk | Trial was unblinded. |
|  | Blinded outcome assessment (assessor bias) | High risk | “…those assessing study outcomes were blind to group assignment.”  Outcome assessment was blind. |
|  | Incomplete outcome data (attrition bias) | High risk | Amount of missing data was enough to induce bias in intervention effect estimate. |
|  | Similarity in attrition across trial arms (attrition bias) | High risk | Different proportion of participants lost to follow-up across trial arms (19% and 29%). |
| [129] Lincoln NB, Flannaghan T. Cognitive Behavioral Psychotherapy for Depression Following Stroke: A Randomized Controlled Trial. Stroke. 2003;34:111-5. | Jadad score (0-5; N/A for trial protocols) | 3 | Study was described as randomised, with appropriate utilisation of random allocation, and included a description of withdrawals/dropouts. |
|  | Allocation sequence (selection bias) | Low risk | Used computerised randomisation. |
|  | Allocation concealment (selection bias) | Low risk | “A computer-generated random number sequence was prepared in advance and sealed in opaque, consecutively numbered envelopes by an independent researcher.”  Allocation was concealed. |
|  | Order of randomisation and consent (selection bias) | Low risk | The trial flow chart (Figure 1) indicates that participant consent took place before randomisation.  Randomisation followed consent. |
|  | Order of randomisation and baseline measures (selection bias) | Low risk | Participants completed the various outcomes (BDI, WDI, LHS, EADL) during the phase before randomisation.  Randomisation followed baseline measures. |
|  | Similarity of baseline outcome measurements across trial arms (selection bias) | Low risk | “There were no statistically significant differences between the groups at baseline, 3 months, or 6 months on any of the measures.”  No evidence of a difference in baseline outcome measures between trial arms (also see Table 2). |
|  | Similarity of baseline characteristics across trial arms (selection bias) | Low risk | No evidence of a difference in baseline characteristics between trial arms (Table 1). |
|  | Blinding of participants and personnel (performance bias) | High risk | Trial was unblinded. |
|  | Blinded outcome assessment (assessor bias) | Low risk | “Outcome assessments were administered by an assistant psychologist, who was blind to the group allocation, 3 and 6 months after randomization.”  Outcome assessment was blind. |
|  | Incomplete outcome data (attrition bias) | Low risk | Amount of missing data was low and balanced across trial arms. |
|  | Similarity in attrition across trial arms (attrition bias) | Low risk | Similar proportion of participants lost to follow-up across trial arms (12%, 5% and 13%). |
| [130] Lipsitz JD, Gur M, Vermes D, Petkova E, Cheng J, Miller N, et al. A randomized trial of interpersonal therapy versus supportive therapy for social anxiety disorder. Depression and Anxiety. 2008;25:542-53. | Jadad score (0-5; N/A for trial protocols) | 3 | Study was described as randomised, with appropriate utilisation of random allocation, and included a description of withdrawals/dropouts. |
|  | Allocation sequence (selection bias) | Low risk | Used computerised randomisation. |
|  | Allocation concealment (selection bias) | Unclear | Not enough information to make judgement. |
|  | Order of randomisation and consent (selection bias) | Low risk | “All participants provided informed consent in writing  after thorough discussion of study procedures and  before entering the study.”  Randomisation followed consent. |
|  | Order of randomisation and baseline measures (selection bias) | Unclear | Not enough information to make judgement. |
|  | Similarity of baseline outcome measurements across trial arms (selection bias) | Low risk | No evidence of a difference in baseline outcome measures between trial arms (Table 2). |
|  | Similarity of baseline characteristics across trial arms (selection bias) | High risk | Evidence of a difference in baseline characteristics between trial arms (Table 1). |
|  | Blinding of participants and personnel (performance bias) | High risk | Trial was unblinded. |
|  | Blinded outcome assessment (assessor bias) | Low risk | “Assessment included both clinician and self-rated measures.  All clinician-rated measures were administered by independent evaluators (IEs)… All IEs were kept blind to the patient’s treatment group status.”  Outcome assessments were blind. |
|  | Incomplete outcome data (attrition bias) | High risk | Amount of missing data was enough to induce bias in intervention effect estimate. |
|  | Similarity in attrition across trial arms (attrition bias) | Low risk | Similar proportion of participants lost to follow-up across trial arms (28% and 24%). |
| [131] Littbrand H, Lundin-Olsson L, Gustafson Y, Rosendahl E. The Effect of a High-Intensity Functional Exercise Program on Activities of Daily Living: A Randomized Controlled Trial in Residential Care Facilities. Journal of the American Geriatrics Society. 2009;57:1741-9. | Jadad score (0-5; N/A for trial protocols) | 2 | Study was described as randomised, and included a description of withdrawals/dropouts. |
|  | Allocation sequence (selection bias) | Unclear | Not enough information to make judgement. |
|  | Allocation concealment (selection bias) | Unclear | “Researchers not involved in the study performed the randomization using lots in sealed nontransparent envelopes.” Unclear if envelopes were sequentially numbered.  Not enough information to make judgement. |
|  | Order of randomisation and consent (selection bias) | Low risk | “To exclude the possibility of selection bias, randomization was performed after inclusion of the participants and baseline assessments.”  Randomisation followed consent. |
|  | Order of randomisation and baseline measures (selection bias) | Low risk | “To exclude the possibility of selection bias, randomization was performed after inclusion of the participants and baseline assessments.”  Randomisation followed baseline measures. |
|  | Similarity of baseline outcome measurements across trial arms (selection bias) | Low risk | No evidence of a difference in baseline outcome measures between trial arms (Table 2). |
|  | Similarity of baseline characteristics across trial arms (selection bias) | Low risk | No evidence of a difference in baseline characteristics between trial arms (Table 1). |
|  | Blinding of participants and personnel (performance bias) | High risk | Trial was unblinded. |
|  | Blinded outcome assessment (assessor bias) | Low risk | “Trained PTs blinded to group allocations and previous test results recorded the Barthel Index [the outcome] at baseline and at the 3- (end of intervention period) and 6-month follow-ups.”  Outcome assessment was blind. |
|  | Incomplete outcome data (attrition bias) | High risk | Amount of missing data was enough to induce bias in intervention effect estimate. |
|  | Similarity in attrition across trial arms (attrition bias) | Low risk | Similar proportion of participants lost to follow-up across trial arms (14% and 9%). |
| [132] Lobban F, Taylor L, Chandler C, Tyler E, Kinderman P, Kolamunnage-Dona R, et al. Enhanced relapse prevention for bipolar disorder by community mental health teams: cluster feasibility randomised trial. British Journal of Psychiatry. 2010;196:59-63. | Jadad score (0-5; N/A for trial protocols) | 2 | Study was described as randomised, and included a description of withdrawals/dropouts. |
|  | Allocation sequence (selection bias) | Unclear | Not enough information to make judgement. |
|  | Allocation concealment (selection bias) | Unclear | Not enough information to make judgement. |
|  | Order of randomisation and consent (selection bias) | High risk | “Attempts to recruit participants prior to randomisation also failed and approximately half of participants were recruited after randomisation.”  Randomisation sometimes preceded consent. |
|  | Order of randomisation and baseline measures (selection bias) | High risk | “Attempts to recruit participants prior to randomisation also failed and approximately half of participants were recruited after randomisation.”  Randomisation sometimes preceded baseline measures. |
|  | Similarity of baseline outcome measurements across trial arms (selection bias) | Low risk | No evidence of a difference in baseline outcome measures between trial arms (Figure DS1). |
|  | Similarity of baseline characteristics across trial arms (selection bias) | Low risk | “There was no difference between the demographic and clinical characteristics of the participants in each arm, except that more participants in the usual treatment arm had children (online Table DS1).”  No evidence of a difference in baseline characteristics between trial arms. |
|  | Blinding of participants and personnel (performance bias) | High risk | Trial was unblinded. |
|  | Blinded outcome assessment (assessor bias) | Low risk | “Individuals with a clinical diagnosis of bipolar disorder were assessed by researchers (C.C., E.T.) masked to allocation, using an interview to confirm diagnosis, and a range of measures at baseline and at 12 weeks, 24 weeks, 36 weeks and 48 weeks following the onset of treatment.”  Outcome assessment was blind. |
|  | Incomplete outcome data (attrition bias) | High risk | Amount of missing data was low but was not balanced across trial arms. |
|  | Similarity in attrition across trial arms (attrition bias) | High risk | Different proportion of participants lost to follow-up across trial arms (11% and 0%). |
| [133] Lokk JCT, Arnetz BB. Impact of Management Change and an Intervention Program on Health Care Personnel. Psychotherapy & Psychosomatics. 2000;69:79-85. | Jadad score (0-5; N/A for trial protocols) | 1 | Study was described as randomised. |
|  | Allocation sequence (selection bias) | Unclear | Not enough information to make judgement. |
|  | Allocation concealment (selection bias) | Unclear | Not enough information to make judgement. |
|  | Order of randomisation and consent (selection bias) | Unclear | Not enough information to make judgement. |
|  | Order of randomisation and baseline measures (selection bias) | Unclear | Not enough information to make judgement. |
|  | Similarity of baseline outcome measurements across trial arms (selection bias) | Low risk | No evidence of a difference in baseline outcome measures between trial arms (Table 3). |
|  | Similarity of baseline characteristics across trial arms (selection bias) | Low risk | No evidence of a difference in baseline characteristics between trial arms (Table 1). |
|  | Blinding of participants and personnel (performance bias) | High risk | Trial was unblinded. |
|  | Blinded outcome assessment (assessor bias) | Unclear | Not enough information to make judgement. |
|  | Incomplete outcome data (attrition bias) | Unclear | Not enough information to make judgement. |
|  | Similarity in attrition across trial arms (attrition bias) | Unclear | Not enough information to make judgement. |
| [134] Lourenco LBdA, Rodrigues RCM, Ciol MA, Sao-Joao TM, Cornelio ME, Dantas RA, et al. A randomized controlled trial of the effectiveness of planning strategies in the adherence to medication for coronary artery disease. Journal of Advanced Nursing. 2014;70:1616-28. | Jadad score (0-5; N/A for trial protocols) | 3 | Study was described as randomised, with appropriate utilisation of random allocation, and included a description of withdrawals/dropouts. |
|  | Allocation sequence (selection bias) | Low risk | Used computerised randomisation. |
|  | Allocation concealment (selection bias) | Unclear | Not enough information to make judgement. |
|  | Order of randomisation and consent (selection bias) | Low risk | “At the enrolment visit (hereafter, denominated baseline), the participants signed an informed consent and were randomized to either intervention or control groups.”  Randomisation followed consent. |
|  | Order of randomisation and baseline measures (selection bias) | High risk | Participants were “randomized into two groups (64 in the intervention and 62 in the control group). Eleven participants did not come to the interview at baseline and were excluded from further contact.”  Randomisation preceded baseline measures. |
|  | Similarity of baseline outcome measurements across trial arms (selection bias) | High risk | Evidence of a difference in baseline outcome measures between trial arms (Table 2). |
|  | Similarity of baseline characteristics across trial arms (selection bias) | Low risk | No evidence of a difference in baseline characteristics between trial arms (Table 1). |
|  | Blinding of participants and personnel (performance bias) | High risk | Trial was unblinded. |
|  | Blinded outcome assessment (assessor bias) | Unclear | Not enough information to make judgement. |
|  | Incomplete outcome data (attrition bias) | Low risk | Amount of missing data was low and balanced across trial arms. |
|  | Similarity in attrition across trial arms (attrition bias) | Low risk | Similar proportion of participants lost to follow-up across trial arms (8% and 10%). |
| [135] Lu D-F, Hart LK, Lutgendorf SK, Oh H, Schilling M. Slowing progression of early stages of AD with alternative therapies: A feasibility study. Geriatric Nursing. 2013;34:457-64. | Jadad score (0-5; N/A for trial protocols) | 2 | Study was described as randomised, with appropriate utilisation of random allocation. |
|  | Allocation sequence (selection bias) | Low risk | Used coin tossing. |
|  | Allocation concealment (selection bias) | Unclear | Not enough information to make judgement. |
|  | Order of randomisation and consent (selection bias) | Unclear | Not enough information to make judgement. |
|  | Order of randomisation and baseline measures (selection bias) | Unclear | Not enough information to make judgement. |
|  | Similarity of baseline outcome measurements across trial arms (selection bias) | Low risk | “The groups did not differ significantly at baseline… on the outcome variables (cognitive function, mood, & depression).”  No evidence of a difference in baseline outcome measures between trial arms. |
|  | Similarity of baseline characteristics across trial arms (selection bias) | High risk | Some evidence of a difference in baseline characteristics between trial arms (Table 2). |
|  | Blinding of participants and personnel (performance bias) | High risk | Trial was unblinded. |
|  | Blinded outcome assessment (assessor bias) | Unclear | Not enough information to make judgement. |
|  | Incomplete outcome data (attrition bias) | Low risk | Amount of missing data was low and balanced across trial arms. |
|  | Similarity in attrition across trial arms (attrition bias) | Low risk | Similar numbers of participants were lost over follow-up (0 and 1 participants). |
| [136] Malow RM, Stein JA, McMahon RC, Devieux JG, Rosenberg R, Jean-Gilles M. Effects of a Culturally Adapted HIV Prevention Intervention in Haitian Youth. Journal of the Association of Nurses in AIDS Care March/April. 2009;20:110-21. | Jadad score (0-5; N/A for trial protocols) | 1 | Study was described as randomised. |
|  | Allocation sequence (selection bias) | Unclear | Not enough information to make judgement. |
|  | Allocation concealment (selection bias) | Unclear | Not enough information to make judgement. |
|  | Order of randomisation and consent (selection bias) | Unclear | Not enough information to make judgement. |
|  | Order of randomisation and baseline measures (selection bias) | Unclear | Not enough information to make judgement. |
|  | Similarity of baseline outcome measurements across trial arms (selection bias) | Low risk | No evidence of a difference in baseline outcome measures between trial arms (Table 1). |
|  | Similarity of baseline characteristics across trial arms (selection bias) | Unclear | Not enough information to make judgement. |
|  | Blinding of participants and personnel (performance bias) | High risk | Trial was unblinded. |
|  | Blinded outcome assessment (assessor bias) | Unclear | Not enough information to make judgement. |
|  | Incomplete outcome data (attrition bias) | High risk | Amount of missing data was enough to induce bias in intervention effect estimate. |
|  | Similarity in attrition across trial arms (attrition bias) | High risk | Different proportion of participants were lost over follow-up (43% and 57%). |
| [137] Marshall M, Lockwood A, Green G, Zajac-Roles G, Roberts C, Harrison G. Systematic assessments of need and care planning in severe mental illness: Cluster randomised controlled trial. British Journal of Psychiatry August. 2004;185:163-8. | Jadad score (0-5; N/A for trial protocols) | 3 | Study was described as randomised, with appropriate utilisation of random allocation, and included a description of withdrawals/dropouts. |
|  | Allocation sequence (selection bias) | Low risk | Used computerised randomisation. |
|  | Allocation concealment (selection bias) | Unclear | Used “numbered, sealed envelopes”. Not clear if they were opaque.  Not enough information to make judgement. |
|  | Order of randomisation and consent (selection bias) | Unclear | Not enough information to make judgement. |
|  | Order of randomisation and baseline measures (selection bias) | Unclear | Not enough information to make judgement. |
|  | Similarity of baseline outcome measurements across trial arms (selection bias) | Unclear | Not enough information to make judgement. |
|  | Similarity of baseline characteristics across trial arms (selection bias) | Low risk | No evidence of a difference in baseline characteristics between trial arms (Table 2). |
|  | Blinding of participants and personnel (performance bias) | High risk | Trial was unblinded. |
|  | Blinded outcome assessment (assessor bias) | Low risk | “Follow-up at 12 months was conducted by a different research nurse who was masked to group allocation.”  Outcome assessment was blind. |
|  | Incomplete outcome data (attrition bias) | High risk | Amount of missing data was enough to induce bias in intervention effect estimate. |
|  | Similarity in attrition across trial arms (attrition bias) | Low risk | Similar proportion of participants were lost over follow-up (22%, 21% and 18%). |
| [138] Martens MP, Smith AE, Murphy JG. The Efficacy of Single-Component Brief Motivational Interventions Among at-Risk College Drinkers. Journal of Consulting & Clinical Psychology. 2013;81:691-701. | Jadad score (0-5; N/A for trial protocols) | 3 | Study was described as randomised, with appropriate utilisation of random allocation, and included a description of withdrawals/dropouts. |
|  | Allocation sequence (selection bias) | Low risk | Used random number table. |
|  | Allocation concealment (selection bias) | Unclear | Not enough information to make judgement. |
|  | Order of randomisation and consent (selection bias) | Low risk | “After completing the informed consent questionnaire participants completed the additional eligibility screening questionnaires assessing for the presence of alcohol dependence, regular drug use, and elevated depressive symptoms. Ineligible students were informed of the reason for their ineligibility and provided a referral to the university counseling center. All eligible individuals completed the baseline battery of questionnaires, were randomly assigned to one of the three intervention conditions, and completed their intervention.”  Randomisation followed consent. |
|  | Order of randomisation and baseline measures (selection bias) | Low risk | “After completing the informed consent questionnaire participants completed the additional eligibility screening questionnaires assessing for the presence of alcohol dependence, regular drug use, and elevated depressive symptoms. Ineligible students were informed of the reason for their ineligibility and provided a referral to the university counseling center. All eligible individuals completed the baseline battery of questionnaires, were randomly assigned to one of the three intervention conditions, and completed their intervention.”  Randomisation followed baseline measures. |
|  | Similarity of baseline outcome measurements across trial arms (selection bias) | Low risk | No evidence of a difference in baseline outcome measures between trial arms (Tables 1 and 2). |
|  | Similarity of baseline characteristics across trial arms (selection bias) | Low risk | No evidence of a difference in baseline characteristics between trial arms (Table 1). |
|  | Blinding of participants and personnel (performance bias) | High risk | Trial was unblinded. |
|  | Blinded outcome assessment (assessor bias) | N/A | No outcome assessors (participants completed questionnaires). |
|  | Incomplete outcome data (attrition bias) | Low risk | Amount of missing data was low and balanced across trial arms. |
|  | Similarity in attrition across trial arms (attrition bias) | Low risk | Similar proportion of participants were lost over follow-up (7%, 7% and 4%). |
| [139] Martinsen M, Bahr R, Borresen R, Holme I, Pensgaard AM, Sundgot-Borgen J. Preventing Eating Disorders among Young Elite Athletes: A Randomized Controlled Trial. Medicine & Science in Sports & Exercise. 2014;46:435-47. | Jadad score (0-5; N/A for trial protocols) | 2 | Study was described as randomised, and included a description of withdrawals/dropouts. |
|  | Allocation sequence (selection bias) | Unclear | Not enough information to make judgement. |
|  | Allocation concealment (selection bias) | Unclear | Not enough information to make judgement. |
|  | Order of randomisation and consent (selection bias) | Low risk | The trial flow chart (Figure 1) indicates that participant enrolment took place before randomisation.  Randomisation followed consent. |
|  | Order of randomisation and baseline measures (selection bias) | Low risk | The trial flow chart (Figure 1) indicates that participant enrolment took place before randomisation.  Randomisation followed baseline measures. |
|  | Similarity of baseline outcome measurements across trial arms (selection bias) | Low risk | No evidence of a difference in baseline outcome measures between trial arms (Tables 3 and 4). |
|  | Similarity of baseline characteristics across trial arms (selection bias) | Low risk | No evidence of a difference in baseline characteristics between trial arms (Table 2). |
|  | Blinding of participants and personnel (performance bias) | High risk | Trial was unblinded. |
|  | Blinded outcome assessment (assessor bias) | Unclear | Not enough information to make judgement. |
|  | Incomplete outcome data (attrition bias) | High risk | Amount of missing data was enough to induce bias in intervention effect estimate. |
|  | Similarity in attrition across trial arms (attrition bias) | Low risk | Similar proportion of participants were lost over follow-up (18% and 21%). |
| [140] Masia Warner C, Fisher PH, Shrout PE, Rathor S, Klein RG. Treating adolescents with social anxiety disorder in school: an attention control trial. Journal of Child Psychology & Psychiatry. 2007;48:676-86. | Jadad score (0-5; N/A for trial protocols) | 2 | Study was described as randomised, and included a description of withdrawals/dropouts. |
|  | Allocation sequence (selection bias) | Unclear | Not enough information to make judgement. |
|  | Allocation concealment (selection bias) | Unclear | Not enough information to make judgement. |
|  | Order of randomisation and consent (selection bias) | Unclear | Not enough information to make judgement. |
|  | Order of randomisation and baseline measures (selection bias) | Unclear | Not enough information to make judgement. |
|  | Similarity of baseline outcome measurements across trial arms (selection bias) | Low risk | No evidence of a difference in baseline outcome measures between trial arms (Table 2). |
|  | Similarity of baseline characteristics across trial arms (selection bias) | Low risk | No evidence of a difference in baseline characteristics between trial arms (Table 1). |
|  | Blinding of participants and personnel (performance bias) | High risk | Trial was unblinded. |
|  | Blinded outcome assessment (assessor bias) | Low risk | “Trained independent evaluators (IE), blind to treatment condition, conducted all clinical assessments.”  Outcome assessment was blind. |
|  | Incomplete outcome data (attrition bias) | Low risk | Amount of missing data was low and balanced across trial arms. |
|  | Similarity in attrition across trial arms (attrition bias) | Low risk | Similar numbers of participants were lost over follow-up (2 and 2 participants). |
| [141] McCambridge J, Bendtsen M, Karlsson N, White IR, Nilsen P, Bendtsen P. Alcohol assessment and feedback by email for university students: main findings from a randomised controlled trial. British Journal of Psychiatry. 2013;203:334-40. | Jadad score (0-5; N/A for trial protocols) | 3 | Study was described as randomised, with appropriate utilisation of random allocation, and included a description of withdrawals/dropouts. |
|  | Allocation sequence (selection bias) | Low risk | Used computerised randomisation. |
|  | Allocation concealment (selection bias) | Unclear | Not enough information to make judgement. |
|  | Order of randomisation and consent (selection bias) | N/A | There was no consent process. |
|  | Order of randomisation and baseline measures (selection bias) | High risk | The trial flow chart (Figure 1) indicates that participant enrolment took place after randomisation.  Randomisation preceded baseline measures. |
|  | Similarity of baseline outcome measurements across trial arms (selection bias) | Low risk | “The proportion who were risky drinkers at baseline was similar in groups 1 and 2.”  No evidence of a difference in baseline outcome measures between trial arms. |
|  | Similarity of baseline characteristics across trial arms (selection bias) | Low risk | No evidence of a difference in baseline characteristics between trial arms (Table 2). |
|  | Blinding of participants and personnel (performance bias) | High risk | Trial was unblinded (participants did not know they were taking part in a trial but those in active treatment arm did know about existence of the feedback intervention). |
|  | Blinded outcome assessment (assessor bias) | N/A | No outcome assessors (participants completed questionnaires). |
|  | Incomplete outcome data (attrition bias) | High risk | Amount of missing data was enough to induce bias in intervention effect estimate. |
|  | Similarity in attrition across trial arms (attrition bias) | Low risk | Similar proportion of participants were lost over follow-up (49%, 48% and 46%). |
| [142] McCambridge J, Strang J. The efficacy of single-session motivational interviewing in reducing drug consumption and perceptions of drug-related risk and harm among young people: results from a multi-site cluster randomized trial. Addiction. 2004;99:39-52. | Jadad score (0-5; N/A for trial protocols) | 1 | Study was described as randomised. |
|  | Allocation sequence (selection bias) | Unclear | Not enough information to make judgement. |
|  | Allocation concealment (selection bias) | Unclear | Not enough information to make judgement. |
|  | Order of randomisation and consent (selection bias) | Unclear | Not enough information to make judgement. |
|  | Order of randomisation and baseline measures (selection bias) | Unclear | Not enough information to make judgement. |
|  | Similarity of baseline outcome measurements across trial arms (selection bias) | High risk | Some evidence of a difference in baseline outcome measures between trial arms (Table 1). |
|  | Similarity of baseline characteristics across trial arms (selection bias) | High risk | Some evidence of a difference in baseline characteristics between trial arms (Table 1). |
|  | Blinding of participants and personnel (performance bias) | High risk | Trial was unblinded. |
|  | Blinded outcome assessment (assessor bias) | High risk | “A further area of possible bias was that intervention recipients might report more favourable outcome data to the researcher who had delivered the intervention (J.M.).”  Outcome assessment was not blind. |
|  | Incomplete outcome data (attrition bias) | High risk | Amount of missing data was enough to induce bias in intervention effect estimate. |
|  | Similarity in attrition across trial arms (attrition bias) | High risk | Different proportion of participants were lost over follow-up (8% and 14%). |
| [143] McLaughlin TJ, Aupont O, Bambauer KZ, Stone P, Mullan MG, Colagiovanni J, et al. Improving Psychologic Adjustment to Chronic Illness in Cardiac Patients: The Role of Depression and Anxiety. Journal of General Internal Medicine. 2005;20:1084-90. | Jadad score (0-5; N/A for trial protocols) | 3 | Study was described as randomised, with appropriate utilisation of random allocation, and included a description of withdrawals/dropouts. |
|  | Allocation sequence (selection bias) | Low risk | Used coin tossing. |
|  | Allocation concealment (selection bias) | Unclear | Not enough information to make judgement. |
|  | Order of randomisation and consent (selection bias) | Low risk | “Once patients consented, the study coordinator administered the 14-item HADS by phone to assess symptoms of anxiety and depression. Subjects with scores between 7 and 15 on either the anxiety or depression scale were enrolled and randomized by coin flip.”  Randomisation followed consent. |
|  | Order of randomisation and baseline measures (selection bias) | High risk | “Eight experimental patients (15.1%) dropped out of the study before baseline measures were collected.”  Randomisation preceded baseline measures. |
|  | Similarity of baseline outcome measurements across trial arms (selection bias) | Low risk | No evidence of a difference in baseline outcome measures between trial arms (Table 1). |
|  | Similarity of baseline characteristics across trial arms (selection bias) | Low risk | No evidence of a difference in baseline characteristics between trial arms (Table 1). |
|  | Blinding of participants and personnel (performance bias) | High risk | Trial was unblinded. |
|  | Blinded outcome assessment (assessor bias) | Unclear | Not enough information to make judgement. |
|  | Incomplete outcome data (attrition bias) | High risk | Amount of missing data was enough to induce bias in intervention effect estimate. |
|  | Similarity in attrition across trial arms (attrition bias) | High risk | Different proportion of participants were lost over follow-up (28% and 15%). |
| [144] McSweeney K, Jeffreys A, Griffith J, Plakiotis C, Kharsas R, O'Connor DW. Specialist mental health consultation for depression in Australian aged care residents with dementia: a cluster randomized trial. International Journal of Geriatric Psychiatry. 2012;27:1163-71. | Jadad score (0-5; N/A for trial protocols) | 3 | Study was described as randomised, with appropriate utilisation of random allocation, and included a description of withdrawals/dropouts. |
|  | Allocation sequence (selection bias) | Low risk | Used coin tossing. |
|  | Allocation concealment (selection bias) | Unclear | Not enough information to make judgement. |
|  | Order of randomisation and consent (selection bias) | Unclear | Not enough information to make judgement. |
|  | Order of randomisation and baseline measures (selection bias) | Unclear | Not enough information to make judgement. |
|  | Similarity of baseline outcome measurements across trial arms (selection bias) | High risk | Evidence of a difference in baseline outcome measures between trial arms (Table 1). |
|  | Similarity of baseline characteristics across trial arms (selection bias) | High risk | Evidence of a difference in baseline characteristics between trial arms (Table 1). |
|  | Blinding of participants and personnel (performance bias) | High risk | Trial was unblinded. |
|  | Blinded outcome assessment (assessor bias) | Low risk | “The post-intervention assessment was scheduled for approximately 15 weeks following the pre-intervention assessment, and was conducted by a psychologist blind to study condition.”  Outcome assessment was blind. |
|  | Incomplete outcome data (attrition bias) | Low risk | Amount of missing data was low and balanced across trial arms. |
|  | Similarity in attrition across trial arms (attrition bias) | Low risk | Similar number of participants were lost over follow-up (4 and 1 participants). |
| [145] Mealer M, Conrad D, Evans J, Jooste K, Solyntjes J, Rothbaum B, et al. Feasibility and Acceptability of a Resilience Training Program for Intensive Care Unit Nurses. American Journal of Critical Care. 2014;23:e97-e105. | Jadad score (0-5; N/A for trial protocols) | 2 | Study was described as randomised, and included a description of withdrawals/dropouts. |
|  | Allocation sequence (selection bias) | Unclear | Not enough information to make judgement. |
|  | Allocation concealment (selection bias) | Unclear | Not enough information to make judgement. |
|  | Order of randomisation and consent (selection bias) | Unclear | Not enough information to make judgement. |
|  | Order of randomisation and baseline measures (selection bias) | Unclear | Not enough information to make judgement. |
|  | Similarity of baseline outcome measurements across trial arms (selection bias) | Low risk | No evidence of a difference in baseline outcome measures between trial arms (Table 1). |
|  | Similarity of baseline characteristics across trial arms (selection bias) | Low risk | No evidence of a difference in baseline characteristics between trial arms (Table 1). |
|  | Blinding of participants and personnel (performance bias) | High risk | Trial was unblinded. |
|  | Blinded outcome assessment (assessor bias) | Unclear | Not enough information to make judgement. |
|  | Incomplete outcome data (attrition bias) | Low risk | Amount of missing data was low and balanced across trial arms. |
|  | Similarity in attrition across trial arms (attrition bias) | Low risk | Similar number of participants were lost over follow-up (1 and 1 participants). |
| [146] van Meijel B, Kruitwagen C, van der Gaag M, Kahn RS, Grypdonck MHF. An Intervention Study to Prevent Relapse in Patients With Schizophrenia. Journal of Nursing Scholarship. 2006;38:42-9. | Jadad score (0-5; N/A for trial protocols) | 2 | Study was described as randomised, and included a description of withdrawals/dropouts. |
|  | Allocation sequence (selection bias) | Unclear | Not enough information to make judgement. |
|  | Allocation concealment (selection bias) | Unclear | Not enough information to make judgement. |
|  | Order of randomisation and consent (selection bias) | High risk | “…selected nurses were then divided at random per department between the experimental and the control conditions... The nurses then listed the patients under their care who met the selection criteria. To avoid a selection bias, the researcher determined at random the order in which the patients would be approached for participation in the study.”  Randomisation followed consent. |
|  | Order of randomisation and baseline measures (selection bias) | High risk | “…selected nurses were then divided at random per department between the experimental and the control conditions... The nurses then listed the patients under their care who met the selection criteria. To avoid a selection bias, the researcher determined at random the order in which the patients would be approached for participation in the study.”  Randomisation followed baseline measures. |
|  | Similarity of baseline outcome measurements across trial arms (selection bias) | Low risk | No evidence of a difference in baseline outcome measures between trial arms (Table 1). |
|  | Similarity of baseline characteristics across trial arms (selection bias) | High risk | Evidence of a difference in baseline characteristics between trial arms (Table 1). |
|  | Blinding of participants and personnel (performance bias) | High risk | Trial was unblinded. |
|  | Blinded outcome assessment (assessor bias) | Unclear | Not enough information to make judgement. |
|  | Incomplete outcome data (attrition bias) | High risk | Amount of missing data was enough to induce bias in intervention effect estimate. |
|  | Similarity in attrition across trial arms (attrition bias) | High risk | Different proportion of participants were lost over follow-up (22% and 5%). |
| [147] Melville JL, Reed SD, Russo J, Croicu CA, Ludman E, LaRocco-Cockburn A, et al. Improving Care for Depression in Obstetrics and Gynecology: A Randomized Controlled Trial. Obstetrics & Gynecology. 2014;123:1237-46. | Jadad score (0-5; N/A for trial protocols) | 3 | Study was described as randomised, with appropriate utilisation of random allocation, and included a description of withdrawals/dropouts. |
|  | Allocation sequence (selection bias) | Low risk | Used computerised randomisation. |
|  | Allocation concealment (selection bias) | Low risk | Allocation was done remotely (“off site”).  Allocation was concealed. |
|  | Order of randomisation and consent (selection bias) | Low risk | The trial flow chart (Figure 1) indicates that participant consent took place before randomisation. The text indicates that consent was a process rather than event; the figure confirms that it occurred before randomisation.  Randomisation followed consent. |
|  | Order of randomisation and baseline measures (selection bias) | Low risk | “Baseline data were collected by research assistants screening patients in each clinic.” The trial flow chart (Figure 1) indicates that screening took place before randomisation.  Randomisation followed baseline measures. |
|  | Similarity of baseline outcome measurements across trial arms (selection bias) | Low risk | “There were no baseline differences between groups (Table 1).” Table 1 includes details of baseline outcome measures.  No evidence of a difference in baseline outcome measures between trial arms. |
|  | Similarity of baseline characteristics across trial arms (selection bias) | Low risk | “There were no baseline differences between groups (Table 1).” Table 1 includes details of baseline characteristics.  No evidence of a difference in baseline characteristics between trial arms. |
|  | Blinding of participants and personnel (performance bias) | High risk | Trial was unblinded. |
|  | Blinded outcome assessment (assessor bias) | Low risk | “Outcomes were measured at 6, 12, and 18 months utilizing standardized questionnaires, collected by phone by a research assistant blinded to intervention status.”  Outcome assessment was blind. |
|  | Incomplete outcome data (attrition bias) | High risk | Amount of missing data was enough to induce bias in intervention effect estimate. |
|  | Similarity in attrition across trial arms (attrition bias) | Low risk | Similar proportion of participants were lost over follow-up (16% and 19%). |
| [148] Meredith LS, Jackson-Triche M, Duan N, Rubenstein LV, Camp P, Wells KB. Quality Improvement for Depression Enhances Long-term Treatment Knowledge for Primary Care Clinicians. Journal of General Internal Medicine. 2000;15:868-77. | Jadad score (0-5; N/A for trial protocols) | 1 | Study was described as randomised. |
|  | Allocation sequence (selection bias) | Unclear | Not enough information to make judgement. |
|  | Allocation concealment (selection bias) | Unclear | Not enough information to make judgement. |
|  | Order of randomisation and consent (selection bias) | Unclear | Not enough information to make judgement. |
|  | Order of randomisation and baseline measures (selection bias) | Unclear | Not enough information to make judgement. |
|  | Similarity of baseline outcome measurements across trial arms (selection bias) | High risk | “QI-therapy clinicians [scored] significantly lower than usual  care clinicians [on the general treatment knowledge scale].”  Evidence of a difference in baseline outcome measures between trial arms (also see Table 3). |
|  | Similarity of baseline characteristics across trial arms (selection bias) | High risk | “Although not statistically significant, there were fewer nonphysicians in the QI-meds group relative to the QI-therapy and usual care groups, and moderate differences in gender, ethnicity, board certification, and readiness to change.”  Evidence of a difference in baseline characteristics between trial arms (also see Table 1). |
|  | Blinding of participants and personnel (performance bias) | High risk | Trial was unblinded. |
|  | Blinded outcome assessment (assessor bias) | N/A | No outcome assessors (participants completed questionnaires). |
|  | Incomplete outcome data (attrition bias) | Unclear | Not enough information to make judgement. |
|  | Similarity in attrition across trial arms (attrition bias) | Unclear | Not enough information to make judgement. |
| [149] Merritt RK, Price JR, Mollison J, Geddes JR. A cluster randomized controlled trial to assess the effectiveness of an intervention to educate students about depression. Psychological Medicine. 2007;37:363-72. | Jadad score (0-5; N/A for trial protocols) | 3 | Study was described as randomised, with appropriate utilisation of random allocation, and included a description of withdrawals/dropouts. |
|  | Allocation sequence (selection bias) | Low risk | Used computer-generated random numbers. |
|  | Allocation concealment (selection bias) | Unclear | Not enough information to make judgement. |
|  | Order of randomisation and consent (selection bias) | Unclear | Not enough information to make judgement. |
|  | Order of randomisation and baseline measures (selection bias) | Unclear | Not enough information to make judgement. |
|  | Similarity of baseline outcome measurements across trial arms (selection bias) | Low risk | No evidence of a difference in baseline outcome measures between trial arms (also see Table 2). |
|  | Similarity of baseline characteristics across trial arms (selection bias) | Low risk | No evidence of a difference in baseline characteristics between trial arms (also see Table 1). |
|  | Blinding of participants and personnel (performance bias) | High risk | Trial was unblinded. |
|  | Blinded outcome assessment (assessor bias) | N/A | No outcome assessors (participants completed questionnaires). |
|  | Incomplete outcome data (attrition bias) | High risk | Amount of missing data was enough to induce bias in intervention effect estimate. |
|  | Similarity in attrition across trial arms (attrition bias) | Low risk | Similar proportion of participants were lost over follow-up (73% and 69%). |
| [150] The Metropolitan Area Child Study Research G, Tolan P. A Cognitive-Ecological Approach to Preventing Aggression in Urban Settings: Initial Outcomes for High-Risk Children. Journal of Consulting & Clinical Psychology. 2002;70:179-94. | Jadad score (0-5; N/A for trial protocols) | 1 | Study was described as randomised. |
|  | Allocation sequence (selection bias) | Unclear | Not enough information to make judgement. |
|  | Allocation concealment (selection bias) | Unclear | Not enough information to make judgement. |
|  | Order of randomisation and consent (selection bias) | Unclear | Not enough information to make judgement. |
|  | Order of randomisation and baseline measures (selection bias) | Unclear | Not enough information to make judgement. |
|  | Similarity of baseline outcome measurements across trial arms (selection bias) | High risk | Some evidence of a difference in baseline outcome measures between trial arms (Table 1), particularly for the early + late intervention. |
|  | Similarity of baseline characteristics across trial arms (selection bias) | Unclear | Not enough information to make judgement. |
|  | Blinding of participants and personnel (performance bias) | High risk | Trial was unblinded. |
|  | Blinded outcome assessment (assessor bias) | Unclear | Not enough information to make judgement. |
|  | Incomplete outcome data (attrition bias) | Unclear | Not enough information to make judgement. |
|  | Similarity in attrition across trial arms (attrition bias) | Unclear | Not enough information to make judgement. |
| [151] Midtgaard J, Christensen JF, Tolver A, Jones LW, Uth J, Rasmussen B, et al. Efficacy of multimodal exercise-based rehabilitation on physical activity, cardiorespiratory fitness, and patient-reported outcomes in cancer survivors: a randomized, controlled trial. Annals of Oncology. 2013;24:2267-73. | Jadad score (0-5; N/A for trial protocols) | 3 | Study was described as randomised, with appropriate utilisation of random allocation, and included a description of withdrawals/dropouts. |
|  | Allocation sequence (selection bias) | Low risk | Used computerised randomisation. |
|  | Allocation concealment (selection bias) | Low risk | Used central allocation (web-based system).  Allocation was concealed. |
|  | Order of randomisation and consent (selection bias) | Unclear | Not enough information to make judgement. |
|  | Order of randomisation and baseline measures (selection bias) | Low risk | “Following baseline assessments, patients were randomly allocated to one of the two experimental groups.”  Randomisation followed baseline measures. |
|  | Similarity of baseline outcome measurements across trial arms (selection bias) | Low risk | No evidence of a difference in baseline outcome measures between trial arms (also see Tables 1, 2, 3 and 4). |
|  | Similarity of baseline characteristics across trial arms (selection bias) | Low risk | No evidence of a difference in baseline characteristics between trial arms (also see Table 1). |
|  | Blinding of participants and personnel (performance bias) | High risk | Trial was unblinded. |
|  | Blinded outcome assessment (assessor bias) | Low risk | “All outcome data were entered and analysed by research assistants and a biostatistician (AT) blinded to participant randomization.”  Outcome assessment was blind. |
|  | Incomplete outcome data (attrition bias) | High risk | Amount of missing data was enough to induce bias in intervention effect estimate. |
|  | Similarity in attrition across trial arms (attrition bias) | High risk | Different proportion of participants were lost over follow-up (25% and 32%). |
| [152] Mills M, Loney P, Jamieson E, Gafni A, Browne G, Bell B, et al. A primary care cardiovascular risk reduction clinic in Canada was more effective and no more expensive than usual on-demand primary care - a randomised controlled trial. Health & Social Care in the Community. 2010;18:30-40. | Jadad score (0-5; N/A for trial protocols) | 3 | Study was described as randomised, with appropriate utilisation of random allocation, and included a description of withdrawals/dropouts. |
|  | Allocation sequence (selection bias) | Low risk | Used computer-generated allocation sequence. |
|  | Allocation concealment (selection bias) | Unclear | Used “opaque envelopes for concealment” and these were “sequential”. Not clear if these were sealed.  Not enough information to make judgement. |
|  | Order of randomisation and consent (selection bias) | Low risk | The trial flow chart (Figure 1) indicates that consent took place before randomisation.  Randomisation followed consent. |
|  | Order of randomisation and baseline measures (selection bias) | Low risk | “Subjects received group assignment by picking the next sequential envelope after all baseline measures were completed (end of the clinical assessment).”  Randomisation followed baseline measures. |
|  | Similarity of baseline outcome measurements across trial arms (selection bias) | Low risk | No evidence of a difference in baseline outcome measures between trial arms (also see Tables 2, 3 and 4). |
|  | Similarity of baseline characteristics across trial arms (selection bias) | Low risk | No evidence of a difference in baseline characteristics between trial arms (also see Table 2). |
|  | Blinding of participants and personnel (performance bias) | High risk | Trial was unblinded. |
|  | Blinded outcome assessment (assessor bias) | Low risk | “Interviewers and the data analyst were blind to group assignment.”  Outcome assessment was blind. |
|  | Incomplete outcome data (attrition bias) | Low risk | Amount of missing data was low and balanced across trial arms. |
|  | Similarity in attrition across trial arms (attrition bias) | Low risk | Similar proportion of participants were lost over follow-up (2%, 5% and 2%). |
| [153] Moadel AB, Bernstein SL, Mermelstein RJ, Arnsten JH, Dolce EH, Shuter J. A Randomized Controlled Trial of a Tailored Group Smoking Cessation Intervention for HIV-Infected Smokers. JAIDS Journal of Acquired Immune Deficiency Syndromes. 2012;61:208-15. | Jadad score (0-5; N/A for trial protocols) | 2 | Study was described as randomised, and included a description of withdrawals/dropouts. |
|  | Allocation sequence (selection bias) | Unclear | Not enough information to make judgement. |
|  | Allocation concealment (selection bias) | Unclear | Not enough information to make judgement. |
|  | Order of randomisation and consent (selection bias) | Low risk | “Subjects who provided consent were randomized in a 1:1 schedule to the two study conditions.”  Randomisation followed consent. |
|  | Order of randomisation and baseline measures (selection bias) | Unclear | Not enough information to make judgement. |
|  | Similarity of baseline outcome measurements across trial arms (selection bias) | Low risk | No evidence of a difference in baseline outcome measures between trial arms (also see Table 1). |
|  | Similarity of baseline characteristics across trial arms (selection bias) | Low risk | No evidence of a difference in baseline characteristics between trial arms (also see Table 1). |
|  | Blinding of participants and personnel (performance bias) | High risk | Trial was unblinded. |
|  | Blinded outcome assessment (assessor bias) | N/A | No outcome assessors (participants completed questionnaires). |
|  | Incomplete outcome data (attrition bias) | Low risk | Amount of missing data was low and balanced across trial arms. |
|  | Similarity in attrition across trial arms (attrition bias) | Low risk | Similar proportion of participants were lost over follow-up (5% and 3%). |
| [154] Moffett JAK, Jackson DA, Richmond S, Hahn S, Coulton S, Farrin A, et al. Randomised trial of a brief physiotherapy intervention compared with usual physiotherapy for neck pain patients: outcomes and patients' preference. BMJ. 2005;330:75. | Jadad score (0-5; N/A for trial protocols) | 2 | Study was described as randomised, and included a description of withdrawals/dropouts. |
|  | Allocation sequence (selection bias) | Unclear | Not enough information to make judgement. |
|  | Allocation concealment (selection bias) | Low risk | Used central allocation (via telephone).  Allocation was concealed. |
|  | Order of randomisation and consent (selection bias) | Low risk | Providing consent was an inclusion criterion.  Randomisation followed consent. |
|  | Order of randomisation and baseline measures (selection bias) | Low risk | “After completing the questionnaires the participants were asked by the research physiotherapist if they had a preference for one or the other treatment group and then randomised to a group.”  Randomisation followed baseline measures. |
|  | Similarity of baseline outcome measurements across trial arms (selection bias) | Low risk | No evidence of a difference in baseline outcome measures between trial arms (also see Table 2). |
|  | Similarity of baseline characteristics across trial arms (selection bias) | Low risk | No evidence of a difference in baseline characteristics between trial arms (also see Table 2). |
|  | Blinding of participants and personnel (performance bias) | High risk | Trial was unblinded. |
|  | Blinded outcome assessment (assessor bias) | Low risk | “…those assessing the outcomes were unaware of the intervention provided.”  Outcome assessment was blind. |
|  | Incomplete outcome data (attrition bias) | High risk | Amount of missing data was enough to induce bias in intervention effect estimate. |
|  | Similarity in attrition across trial arms (attrition bias) | Low risk | Similar proportion of participants were lost over follow-up (13% and 17%). |
| [155] Mohr DC, Carmody T, Erickson L, Jin L, Leader J. Telephone-Administered Cognitive Behavioral Therapy for Veterans Served by Community-Based Outpatient Clinics. Journal of Consulting & Clinical Psychology. 2011;79:261-5. | Jadad score (0-5; N/A for trial protocols) | 2 | Study was described as randomised, and included a description of withdrawals/dropouts. |
|  | Allocation sequence (selection bias) | Unclear | Not enough information to make judgement. |
|  | Allocation concealment (selection bias) | Unclear | Not enough information to make judgement. |
|  | Order of randomisation and consent (selection bias) | Low risk | The trial flow chart (Figure 1) indicates that participant enrolment (including consent) took place before randomisation.  Randomisation followed consent. |
|  | Order of randomisation and baseline measures (selection bias) | Unclear | Not enough information to make judgement. |
|  | Similarity of baseline outcome measurements across trial arms (selection bias) | Low risk | “There was a trend toward higher baseline Ham-D scores in the T-CBT arm, t(83)=–1.93, p=.06; however, no significant differences were present at baseline for the PHQ-9, t(83)=0.25, p=.81, or for PTSD diagnosis, χ^2^(1)=0.16, p=.69.”  Little evidence of a difference in baseline outcome measures between trial arms (also see Table 1). |
|  | Similarity of baseline characteristics across trial arms (selection bias) | Low risk | “There were no significant differences across treatment arm on any of the demographic variables.”  No evidence of a difference in baseline characteristics between trial arms. |
|  | Blinding of participants and personnel (performance bias) | High risk | Trial was unblinded. |
|  | Blinded outcome assessment (assessor bias) | Low risk | “Interview measures were conducted over the telephone by blinded evaluators.”  Outcome assessment was blind. |
|  | Incomplete outcome data (attrition bias) | High risk | Amount of missing data was low but was not balanced across trial arms. |
|  | Similarity in attrition across trial arms (attrition bias) | High risk | Different proportion of participants were lost over follow-up (5% and 16%). |
| [156] Monti PM, Rohsenow DJ, Swift RM, Gulliver SB, Colby SM, Mueller TI, et al. Naltrexone and Cue Exposure With Coping and Communication Skills Training for Alcoholics: Treatment Process and 1-Year Outcomes. Alcoholism: Clinical & Experimental Research. 2001;25:1634-47. | Jadad score (0-5; N/A for trial protocols) | 1 | Study was described as randomised. |
|  | Allocation sequence (selection bias) | Unclear | Not enough information to make judgement. |
|  | Allocation concealment (selection bias) | Unclear | Not enough information to make judgement. |
|  | Order of randomisation and consent (selection bias) | Low risk | 2 Of 1549 alcoholic patients screened, 1162 (75%) were ineligible, and of 384 eligible patients approached for the study, 196 (51%) declined participation… Of 183 patients recruited, 18 left the program quickly, and 165 patients (90%) entered psychosocial treatment (group intent-to-treat sample). “  Randomisation followed consent. |
|  | Order of randomisation and baseline measures (selection bias) | Unclear | Not enough information to make judgement. |
|  | Similarity of baseline outcome measurements across trial arms (selection bias) | Low risk | “In 2 x 2 group medication univariate ANOVAs and logistical regression analyses, no significant main or interaction effects were found for pre-treatment percentage of drinking days, percentage of heavy drinking days, number of drinks per day, number of drug use days, Alcohol Dependence Scale (Skinner and Allen, 1982) total score, sex, race, marital or employment status, education, or age.”  Little evidence of a difference in baseline outcome measures between trial arms. |
|  | Similarity of baseline characteristics across trial arms (selection bias) | Low risk | “In 2 x 2 group medication univariate ANOVAs and logistical regression analyses, no significant main or interaction effects were found for pre-treatment percentage of drinking days, percentage of heavy drinking days, number of drinks per day, number of drug use days, Alcohol Dependence Scale (Skinner and Allen, 1982) total score, sex, race, marital or employment status, education, or age.”  Little evidence of a difference in baseline characteristics between trial arms. |
|  | Blinding of participants and personnel (performance bias) | High risk | Trial was unblinded. |
|  | Blinded outcome assessment (assessor bias) | Low risk | “Assessments during the medication visits and at 3-, 6-, and 12-month follow-ups were administered in person by research assistants who were kept blind to treatments.”  Outcome assessment was blind. |
|  | Incomplete outcome data (attrition bias) | High risk | Amount of missing data was enough (13%) to induce bias in intervention effect estimate. |
|  | Similarity in attrition across trial arms (attrition bias) | Low risk | “…completion rates do not differ significantly by either treatment condition (p>0.20)” |
| [157] Moorey S, Cort E, Kapari M, Monroe B, Hansford P, Mannix K, et al. A cluster randomized controlled trial of cognitive behaviour therapy for common mental disorders in patients with advanced cancer. Psychological Medicine. 2009;39:713-23. | Jadad score (0-5; N/A for trial protocols) | 2 | Study was described as randomised, and included a description of withdrawals/dropouts. |
|  | Allocation sequence (selection bias) | Unclear | Not enough information to make judgement. |
|  | Allocation concealment (selection bias) | Unclear | Not enough information to make judgement. |
|  | Order of randomisation and consent (selection bias) | High risk | The authors “opted to randomize nurses and make sure that we captured all their new case-load. It was therefore possible for nurses to bias the trial by selecting patients who were more likely to benefit from their intervention.”  Randomisation preceded consent. |
|  | Order of randomisation and baseline measures (selection bias) | High risk | The authors “opted to randomize nurses and make sure that we captured all their new case-load. It was therefore possible for nurses to bias the trial by selecting patients who were more likely to benefit from their intervention.”  Randomisation preceded baseline measures. |
|  | Similarity of baseline outcome measurements across trial arms (selection bias) | Low risk | Little evidence of a difference in baseline outcome measures between trial arms (Table 4). |
|  | Similarity of baseline characteristics across trial arms (selection bias) | Low risk | Little evidence of a difference in baseline characteristics between trial arms (Table 3). |
|  | Blinding of participants and personnel (performance bias) | High risk | Trial was unblinded. |
|  | Blinded outcome assessment (assessor bias) | Unclear | Not enough information to make judgement. |
|  | Incomplete outcome data (attrition bias) | High risk | Amount of missing data was enough to induce bias in intervention effect estimate. |
|  | Similarity in attrition across trial arms (attrition bias) | High risk | Different proportion of participants were lost over follow-up (64% and 49%). |
| [158] Morey B, Walker R, Davenport A. More dietetic time, better outcome? Nephron Clin Pract. 2008;109:C173-C80. | Jadad score (0-5; N/A for trial protocols) | 3 | Study was described as randomised, with appropriate utilisation of random allocation, and included a description of withdrawals/dropouts. |
|  | Allocation sequence (selection bias) | Unclear | Not enough information to make judgement. |
|  | Allocation concealment (selection bias) | Unclear | Not enough information to make judgement. |
|  | Order of randomisation and consent (selection bias) | Low risk | The trial flow chart (Figure 1) indicates that participant consent took place before randomisation.  Randomisation followed consent. |
|  | Order of randomisation and baseline measures (selection bias) | Unclear | Not enough information to make judgement. |
|  | Similarity of baseline outcome measurements across trial arms (selection bias) | Low risk | Little evidence of a difference in baseline outcome measures between trial arms (Table 2). |
|  | Similarity of baseline characteristics across trial arms (selection bias) | Low risk | Little evidence of a difference in baseline characteristics between trial arms (Table 1). |
|  | Blinding of participants and personnel (performance bias) | High risk | Trial was unblinded. |
|  | Blinded outcome assessment (assessor bias) | Unclear | Not enough information to make judgement. |
|  | Incomplete outcome data (attrition bias) | High risk | Amount of missing data was enough to induce bias in intervention effect estimate. |
|  | Similarity in attrition across trial arms (attrition bias) | Low risk | Similar proportion of participants were lost over follow-up (12% and 9%). |
| [159] Morrell CJ, Slade P, Warner R, Paley G, Dixon S, Walters SJ, et al. Clinical effectiveness of health visitor training in psychologically informed approaches for depression in postnatal women: pragmatic cluster randomised trial in primary care. BMJ. 2009;338:276-85. | Jadad score (0-5; N/A for trial protocols) | 3 | Study was described as randomised, with appropriate utilisation of random allocation, and included a description of withdrawals/dropouts. |
|  | Allocation sequence (selection bias) | Low risk | Used computerised randomisation. |
|  | Allocation concealment (selection bias) | Low risk | Allocation done by central allocation (by independent statistician).  Allocation was concealed. |
|  | Order of randomisation and consent (selection bias) | High risk | The trial flow chart (Figure 1) indicates that participant enrolment took place after randomisation.  Randomisation preceded consent. |
|  | Order of randomisation and baseline measures (selection bias) | High risk | The trial flow chart (Figure 1) indicates that participant enrolment took place after randomisation.  Randomisation preceded baseline measures. |
|  | Similarity of baseline outcome measurements across trial arms (selection bias) | Low risk | No evidence of a difference in baseline outcome measures between trial arms (Table 1). |
|  | Similarity of baseline characteristics across trial arms (selection bias) | Low risk | No evidence of a difference in baseline characteristics between trial arms (Table 1). |
|  | Blinding of participants and personnel (performance bias) | High risk | Trial was unblinded. |
|  | Blinded outcome assessment (assessor bias) | N/A | No outcome assessors (participants completed questionnaires). |
|  | Incomplete outcome data (attrition bias) | High risk | Amount of missing data was enough to induce bias in intervention effect estimate. |
|  | Similarity in attrition across trial arms (attrition bias) | Low risk | Similar proportion of participants were lost over follow-up (68% and 67%). |
| [160] Morriss R, Dowrick C, Salmon P, Peters S, Dunn G, Rogers A, et al. Cluster randomised controlled trial of training practices in reattribution for medically unexplained symptoms. The British Journal of Psychiatry. 2007;191:536-42. | Jadad score (0-5; N/A for trial protocols) | 3 | Study was described as randomised, with appropriate utilisation of random allocation, and included a description of withdrawals/dropouts. |
|  | Allocation sequence (selection bias) | Low risk | Used computer-generated randomisation sequence. |
|  | Allocation concealment (selection bias) | Low risk | Used central allocation (by telephone). “The randomisation sequence was communicated to the trial coordinator and trainers by telephone but to no other member of the research team until all patients completed follow-up. “  Allocation was concealed. |
|  | Order of randomisation and consent (selection bias) | High risk | After randomisation and “once reattribution training was completed, patients were recruited by a researcher by screening consecutive patients attending a surgery in the waiting room.” Providing consent was an inclusion criterion.  Randomisation preceded consent. |
|  | Order of randomisation and baseline measures (selection bias) | High risk | After randomisation and “once reattribution training was completed, patients were recruited by a researcher by screening consecutive patients attending a surgery in the waiting room.”  Randomisation preceded baseline measures. |
|  | Similarity of baseline outcome measurements across trial arms (selection bias) | Low risk | No evidence of a difference in baseline outcome measures between trial arms (Table 2). |
|  | Similarity of baseline characteristics across trial arms (selection bias) | Low risk | No evidence of a difference in baseline characteristics between trial arms (Table 2). |
|  | Blinding of participants and personnel (performance bias) | High risk | Trial was unblinded. |
|  | Blinded outcome assessment (assessor bias) | Low risk | “All names and places were removed from the transcript so that both raters (L.G. and R.C.) were masked to the intervention group.”  Outcome assessment was blind. |
|  | Incomplete outcome data (attrition bias) | High risk | Amount of missing data was enough to induce bias in intervention effect estimate. |
|  | Similarity in attrition across trial arms (attrition bias) | Low risk | Similar proportion of participants were lost over follow-up (14% and 9%). |
| [161] Muntingh A, van der Feltz-Cornelis C, van Marwijk H, Spinhoven P, Assendelft W, de Waal M, et al. Effectiveness of Collaborative Stepped Care for Anxiety Disorders in Primary Care: A Pragmatic Cluster Randomised Controlled Trial. Psychotherapy & Psychosomatics. 2013;83:37-44. | Jadad score (0-5; N/A for trial protocols) | 3 | Study was described as randomised, with appropriate utilisation of random allocation, and included a description of withdrawals/dropouts. |
|  | Allocation sequence (selection bias) | Low risk | Used “sequences obtained with an automated random sequence generation algorithm”. |
|  | Allocation concealment (selection bias) | Unclear | Not enough information to make judgement. |
|  | Order of randomisation and consent (selection bias) | High risk | The trial flow chart (Figure 1) indicates that participant enrolment took place after randomisation.  Randomisation preceded consent. |
|  | Order of randomisation and baseline measures (selection bias) | High risk | The trial flow chart (Figure 1) indicates that participant enrolment took place after randomisation.  Randomisation preceded baseline measures. |
|  | Similarity of baseline outcome measurements across trial arms (selection bias) | High risk | Evidence of a difference in baseline outcome measures between trial arms (Table 1). |
|  | Similarity of baseline characteristics across trial arms (selection bias) | High risk | Evidence of a difference in baseline characteristics between trial arms (Table 1). |
|  | Blinding of participants and personnel (performance bias) | High risk | Trial was unblinded. |
|  | Blinded outcome assessment (assessor bias) | Low risk | “Questionnaires were processed by research assistants kept blind to group assignment.”  Outcome assessment was blind. |
|  | Incomplete outcome data (attrition bias) | High risk | Amount of missing data was enough to induce bias in intervention effect estimate. |
|  | Similarity in attrition across trial arms (attrition bias) | Low risk | Similar proportion of participants were lost over follow-up (18% and 23%). |
| [162] Murphy AW, Cupples ME, Smith SM, Byrne M, Byrne MC, Newell J, et al. Effect of tailored practice and patient care plans on secondary prevention of heart disease in general practice: cluster randomised controlled trial. BMJ October. 2009;31. | Jadad score (0-5; N/A for trial protocols) | 3 | Study was described as randomised, with appropriate utilisation of random allocation, and included a description of withdrawals/dropouts. |
|  | Allocation sequence (selection bias) | Low risk | Used minimisation. |
|  | Allocation concealment (selection bias) | Unclear | Not enough information to make judgement. |
|  | Order of randomisation and consent (selection bias) | Low risk | “Those who consented attended a baseline consultation at their own practice, where the practice nurse measured their blood pressure… To minimise potential recruitment bias we collected baseline data before randomisation of practices to intervention and control groups.”  Randomisation followed consent. |
|  | Order of randomisation and baseline measures (selection bias) | Low risk | “To minimise potential recruitment bias we collected baseline data before randomisation of practices to intervention and control groups.”  Randomisation followed baseline measures. |
|  | Similarity of baseline outcome measurements across trial arms (selection bias) | Low risk | No evidence of a difference in baseline outcome measures between trial arms (Tables 2 and 3). |
|  | Similarity of baseline characteristics across trial arms (selection bias) | Low risk | No evidence of a difference in baseline characteristics between trial arms (Table 1). |
|  | Blinding of participants and personnel (performance bias) | High risk | Trial was unblinded. |
|  | Blinded outcome assessment (assessor bias) | High risk | “Data collection was not blinded as is common in studies such as this one.”  Outcome assessment was unblind. |
|  | Incomplete outcome data (attrition bias) | High risk | Amount of missing data was enough to induce bias in intervention effect estimate. |
|  | Similarity in attrition across trial arms (attrition bias) | Low risk | Similar proportion of participants were lost over follow-up (5% and 5%; 17% and 15%; 18% and 12% for the various types of data collection). |
| [163] Naylor MD, Hirschman KB, Hanlon AL, Bowles KH, Bradway C, McCauley KM, et al. Comparison of evidence-based interventions on outcomes of hospitalized, cognitively impaired older adults. Journal of Comparative Effectiveness Research. 2014;3:245-57. | Jadad score (0-5; N/A for trial protocols) | 2 | Study was described as randomised, and included a description of withdrawals/dropouts. |
|  | Allocation sequence (selection bias) | Unclear | Not enough information to make judgement. |
|  | Allocation concealment (selection bias) | Unclear | Not enough information to make judgement. |
|  | Order of randomisation and consent (selection bias) | Unclear | Not enough information to make judgement. |
|  | Order of randomisation and baseline measures (selection bias) | Unclear | Not enough information to make judgement. |
|  | Similarity of baseline outcome measurements across trial arms (selection bias) | Low risk | Evidence of a difference in baseline outcome measures between trial arms (Table 1). |
|  | Similarity of baseline characteristics across trial arms (selection bias) | High risk | Evidence of a difference in baseline characteristics between trial arms (Table 1). |
|  | Blinding of participants and personnel (performance bias) | High risk | Trial was unblinded. |
|  | Blinded outcome assessment (assessor bias) | Unclear | Not enough information to make judgement. |
|  | Incomplete outcome data (attrition bias) | High risk | Amount of missing data was enough to induce bias in intervention effect estimate. |
|  | Similarity in attrition across trial arms (attrition bias) | High risk | Similar proportion of participants were lost over follow-up (20%, 22% and 21%). |
| [164] Nourhashemi F, Andrieu S, Gillette-Guyonnet S, Giraudeau B, Cantet C, Coley N, et al. Effectiveness of a specific care plan in patients with Alzheimer's disease: cluster randomised trial (PLASA study). BMJ June. 2010;5. | Jadad score (0-5; N/A for trial protocols) | 3 | Study was described as randomised, with appropriate utilisation of random allocation, and included a description of withdrawals/dropouts. |
|  | Allocation sequence (selection bias) | Low risk | Used computerised randomisation. |
|  | Allocation concealment (selection bias) | Low risk | Randomisation was done remotely (by trial statistician who independent of trial centres).  Allocation was concealed. |
|  | Order of randomisation and consent (selection bias) | High risk | The trial flow chart (Figure) indicates that participant enrolment took place after randomisation.  Randomisation preceded consent. |
|  | Order of randomisation and baseline measures (selection bias) | High risk | The trial flow chart (Figure) indicates that participant enrolment took place after randomisation.  Randomisation preceded baseline measures. |
|  | Similarity of baseline outcome measurements across trial arms (selection bias) | Low risk | No evidence of a difference in baseline outcome measures between trial arms (Table 2). |
|  | Similarity of baseline characteristics across trial arms (selection bias) | Low risk | No evidence of a difference in baseline characteristics between trial arms (Table 1). |
|  | Blinding of participants and personnel (performance bias) | High risk | Trial was unblinded. |
|  | Blinded outcome assessment (assessor bias) | High risk | “…assessors were not blinded to intervention status.”  Outcome assessment was not blind. |
|  | Incomplete outcome data (attrition bias) | High risk | Amount of missing data was enough to induce bias in intervention effect estimate. |
|  | Similarity in attrition across trial arms (attrition bias) | Low risk | Similar proportion of participants were lost over follow-up (42% and 38%). |
| [165] Perkins KA, Marcus MD, Levine MD, D'Amico D, Miller A, Broge M, et al. Cognitive-Behavioral Therapy to Reduce Weight Concerns Improves Smoking Cessation Outcome in Weight-Concerned Women. Journal of Consulting & Clinical Psychology. 2001;69:604-13. | Jadad score (0-5; N/A for trial protocols) | 1 | Study was described as randomised. |
|  | Allocation sequence (selection bias) | Unclear | Not enough information to make judgement. |
|  | Allocation concealment (selection bias) | Unclear | Not enough information to make judgement. |
|  | Order of randomisation and consent (selection bias) | Unclear | Not enough information to make judgement. |
|  | Order of randomisation and baseline measures (selection bias) | Unclear | Not enough information to make judgement. |
|  | Similarity of baseline outcome measurements across trial arms (selection bias) | Unclear | Not enough information to make judgement. |
|  | Similarity of baseline characteristics across trial arms (selection bias) | Low risk | No evidence of a difference in baseline characteristics between trial arms (Table 1). |
|  | Blinding of participants and personnel (performance bias) | High risk | Trial was unblinded. |
|  | Blinded outcome assessment (assessor bias) | Unclear | Not enough information to make judgement. |
|  | Incomplete outcome data (attrition bias) | Unclear | Not enough information to make judgement. |
|  | Similarity in attrition across trial arms (attrition bias) | Unclear | Not enough information to make judgement. |
| [166] Peterson MA, Hamilton EB, Russell AD. Starting well: Facilitating the middle school transition. Journal of Applied School Psychology. 2009;25:286-304. | Jadad score (0-5; N/A for trial protocols) | 1 | Study was described as randomised. |
|  | Allocation sequence (selection bias) | Unclear | Not enough information to make judgement. |
|  | Allocation concealment (selection bias) | Unclear | Not enough information to make judgement. |
|  | Order of randomisation and consent (selection bias) | Unclear | Not enough information to make judgement. |
|  | Order of randomisation and baseline measures (selection bias) | Unclear | Not enough information to make judgement. |
|  | Similarity of baseline outcome measurements across trial arms (selection bias) | Unclear | Not enough information to make judgement. |
|  | Similarity of baseline characteristics across trial arms (selection bias) | Low risk | No evidence of a difference in baseline characteristics between trial arms (Table 1). |
|  | Blinding of participants and personnel (performance bias) | High risk | Trial was unblinded. |
|  | Blinded outcome assessment (assessor bias) | N/A | No outcome assessors (participants completed questionnaires). |
|  | Incomplete outcome data (attrition bias) | Unclear | Not enough information to make judgement. |
|  | Similarity in attrition across trial arms (attrition bias) | Unclear | Not enough information to make judgement. |
| [167] Pfiffner LJ, Hinshaw SP, Owens E, Zalecki C, Kaiser NM, Villodas M, et al. A Two-Site Randomized Clinical Trial of Integrated Psychosocial Treatment for ADHD-Inattentive Type. Journal of Consulting & Clinical Psychology. 2014;82:1115-27. | Jadad score (0-5; N/A for trial protocols) | 2 | Study was described as randomised, and included a description of withdrawals/dropouts. |
|  | Allocation sequence (selection bias) | Unclear | Not enough information to make judgement. |
|  | Allocation concealment (selection bias) | Unclear | Not enough information to make judgement. |
|  | Order of randomisation and consent (selection bias) | Low risk | The trial flow chart (Figure) indicates that participant enrolment (including consent) took place after randomisation.  Randomisation preceded consent. |
|  | Order of randomisation and baseline measures (selection bias) | Unclear | Not enough information to make judgement. |
|  | Similarity of baseline outcome measurements across trial arms (selection bias) | Unclear | Not enough information to make judgement. |
|  | Similarity of baseline characteristics across trial arms (selection bias) | Low risk | Little evidence of a difference in baseline characteristics between trial arms (Table 1). |
|  | Blinding of participants and personnel (performance bias) | High risk | Trial was unblinded. |
|  | Blinded outcome assessment (assessor bias) | High risk | “…because the core outcome measures showing treatment effects were gathered from parents and teachers involved in the treatment, rater bias or expectancy is a potential explanatory factor.”  Outcome assessment was not blind. |
|  | Incomplete outcome data (attrition bias) | High risk | Amount of missing data was enough to induce bias in intervention effect estimate. Also, used inappropriate simple imputation (used last observation carried forward). |
|  | Similarity in attrition across trial arms (attrition bias) | High risk | Different proportion of participants were lost over follow-up (7%, 1% and 12%). |
| [168] Pfiffner LJ, Yee Mikami A, Huang-Pollock C, Easterlin B, Zalecki C, McBurnett K. A Randomized, Controlled Trial of Integrated Home-School Behavioral Treatment for ADHD, Predominantly Inattentive Type. Journal of the American Academy of Child & Adolescent Psychiatry. 2007;46:1041-50. | Jadad score (0-5; N/A for trial protocols) | 2 | Study was described as randomised, and included a description of withdrawals/dropouts. |
|  | Allocation sequence (selection bias) | Unclear | Not enough information to make judgement. |
|  | Allocation concealment (selection bias) | Unclear | Not enough information to make judgement. |
|  | Order of randomisation and consent (selection bias) | Unclear | Not enough information to make judgement. |
|  | Order of randomisation and baseline measures (selection bias) | Unclear | Not enough information to make judgement. |
|  | Similarity of baseline outcome measurements across trial arms (selection bias) | Low risk | No evidence of a difference in baseline outcome measures between trial arms (Table 2). |
|  | Similarity of baseline characteristics across trial arms (selection bias) | Low risk | “Groups did not differ significantly on child age, sex, race, symptoms of hyperactivity/impulsivity, comorbid oppositional defiant disorder, anxiety or depression, IQ or academic achievement (p values > .1).”  No evidence of a difference in baseline characteristics between trial arms. |
|  | Blinding of participants and personnel (performance bias) | High risk | Trial was unblinded. |
|  | Blinded outcome assessment (assessor bias) | Low risk | “Interviewers and raters were blind to child’s group assignment.”  Outcome assessment was blind. |
|  | Incomplete outcome data (attrition bias) | High risk | Amount of missing data was enough to induce bias in intervention effect estimate. |
|  | Similarity in attrition across trial arms (attrition bias) | Low risk | Similar proportion of participants were lost over follow-up (19% and 24%). |
| [169] Phillips G, Bottomley C, Schmidt E, Tobi P, Lais S, Yu G, et al. Well London Phase-1: results among adults of a cluster-randomised trial of a community engagement approach to improving health behaviours and mental well-being in deprived inner-city neighbourhoods. Journal of Epidemiology & Community Health. 2014;68:606-14. | Jadad score (0-5; N/A for trial protocols) | 2 | Study was described as randomised, and included a description of withdrawals/dropouts (at cluster level). |
|  | Allocation sequence (selection bias) | Unclear | Not enough information to make judgement. |
|  | Allocation concealment (selection bias) | Unclear | Not enough information to make judgement. |
|  | Order of randomisation and consent (selection bias) | High risk | “Households were randomly selected in each intervention and control neighbourhood, using the Post Office Address File as a sampling frame. At responding addresses, interviews were sought with every eligible household member aged 16 years and older, and interviews were conducted where consent was given.”  Randomisation preceded consent. |
|  | Order of randomisation and baseline measures (selection bias) | High risk | “Households were randomly selected in each intervention and control neighbourhood, using the Post Office Address File as a sampling frame. At responding addresses, interviews were sought with every eligible household member aged 16 years and older, and interviews were conducted where consent was given.”  Randomisation preceded baseline measures. |
|  | Similarity of baseline outcome measurements across trial arms (selection bias) | Low risk | “The baseline survey findings showed intervention and control neighbourhoods to be similar in terms of demographic characteristics and primary health outcomes (table 1).”  No evidence of a difference in baseline outcome measures between trial arms. |
|  | Similarity of baseline characteristics across trial arms (selection bias) | Low risk | “The baseline survey findings showed intervention and control neighbourhoods to be similar in terms of demographic characteristics and primary health outcomes (table 1).”  No evidence of a difference in baseline characteristics between trial arms. |
|  | Blinding of participants and personnel (performance bias) | High risk | Trial was unblinded. |
|  | Blinded outcome assessment (assessor bias) | Unclear | Not enough information to make judgement. |
|  | Incomplete outcome data (attrition bias) | Low risk | “All analyses were conducted using complete cases because the levels of missing data in the outcomes were low (see online supplementary file 7). For all outcomes, complete cases were defined as survey respondents who were not missing the outcome variable or any sociodemographic variables used for adjustment.”  Amount of missing data was low. |
|  | Similarity in attrition across trial arms (attrition bias) | N/A | Different samples at baseline and follow-up. |
| [170] Powers SW, Kashikar-Zuck SM, Allen JR, LeCates SL, Slater SK, Zafar M, et al. Cognitive Behavioral Therapy Plus Amitriptyline for Chronic Migraine in Children and Adolescents: A Randomized Clinical Trial. JAMA. 2013;310:2622-30. | Jadad score (0-5; N/A for trial protocols) | 3 | Study was described as randomised, with appropriate utilisation of random allocation, and included a description of withdrawals/dropouts. |
|  | Allocation sequence (selection bias) | Low risk | Used computerised randomisation. |
|  | Allocation concealment (selection bias) | Low risk | Allocation was centrally and sent via email.  Allocation was concealed. |
|  | Order of randomisation and consent (selection bias) | Low risk | The trial flow chart (Figure 1) indicates that participant enrolment (including consent) took place before randomisation.  Randomisation followed consent. |
|  | Order of randomisation and baseline measures (selection bias) | Low risk | “All participants completed a baseline assessment that included a medical and psychosocial screening and prospective 28-day headache diary... Participants were randomly assigned to either CBT plus amitriptyline or headache education plus amitriptyline with a 1:1 allocation.”  Randomisation followed baseline measures. |
|  | Similarity of baseline outcome measurements across trial arms (selection bias) | Low risk | No evidence of a difference in baseline outcome measures between trial arms (Table 1). |
|  | Similarity of baseline characteristics across trial arms (selection bias) | Low risk | No evidence of a difference in baseline characteristics between trial arms (Table 1). |
|  | Blinding of participants and personnel (performance bias) | High risk | Trial was unblinded. |
|  | Blinded outcome assessment (assessor bias) | Low risk | “Outcome assessments were conducted by blinded study personnel.”  Outcome assessment was blind. |
|  | Incomplete outcome data (attrition bias) | Low risk | Amount of missing data was low and balanced across trial arms. |
|  | Similarity in attrition across trial arms (attrition bias) | Low risk | Similar proportion of participants were lost over follow-up (11% and 6%). |
| [171] Puschner B, Schofer D, Knaup C, Becker T. Outcome management in in-patient psychiatric care. Acta Psychiatrica Scandinavica. 2009;120:308-19. | Jadad score (0-5; N/A for trial protocols) | 2 | Study was described as randomised, and included a description of withdrawals/dropouts. |
|  | Allocation sequence (selection bias) | Unclear | Not enough information to make judgement. |
|  | Allocation concealment (selection bias) | Low risk | Allocation done centrally (by independent unit).  Allocation was concealed. |
|  | Order of randomisation and consent (selection bias) | Unclear | The Methods section implies that participants were recruited after clusters (clinicians) were allocated to trial arms. Figure 2 implies the opposite.  Not enough information to make judgement. |
|  | Order of randomisation and baseline measures (selection bias) | Unclear | The Methods section implies that participants were recruited after clusters (clinicians) were allocated to trial arms. Figure 2 implies the opposite.  Not enough information to make judgement. |
|  | Similarity of baseline outcome measurements across trial arms (selection bias) | Low risk | No evidence of a difference in baseline outcome measures between trial arms (Table 5). |
|  | Similarity of baseline characteristics across trial arms (selection bias) | Low risk | No evidence of a difference in baseline characteristics between trial arms (Table 1). |
|  | Blinding of participants and personnel (performance bias) | High risk | Trial was unblinded. |
|  | Blinded outcome assessment (assessor bias) | N/A | No outcome assessors (participants completed questionnaires). |
|  | Incomplete outcome data (attrition bias) | High risk | Amount of missing data was enough to induce bias in intervention effect estimate. |
|  | Similarity in attrition across trial arms (attrition bias) | Low risk | Similar proportion of participants were lost over follow-up (19% and 18%). |
| [172] Rabow MW, Dibble SL, Pantilat SZ, McPhee SJ. The Comprehensive Care Team: A Controlled Trial of Outpatient Palliative Medicine Consultation. Archives of Internal Medicine. 2004;164:83-91. | Jadad score (0-5; N/A for trial protocols) | 2 | Study was described as randomised, with appropriate utilisation of random allocation. |
|  | Allocation sequence (selection bias) | Low risk | Used coin tossing. |
|  | Allocation concealment (selection bias) | Low risk | Only two clusters (clinic modules) so only one coin toss needed.  Allocation was concealed. |
|  | Order of randomisation and consent (selection bias) | High risk | Clusters (module clinics) were allocated to treatment, then participants recruitment took place. “We invited all eligible patients referred by their PCP to participate. We excluded patients with nonmelanoma skin cancers, dementia, or psychosis; those enrolled in hospice care; and those unable to complete a written survey in English or Spanish. The PCPs and patients were informed that GMA was assigned to the intervention arm and GMB to the control arm.” Recruitment included obtaining consent.  Randomisation preceded consent. |
|  | Order of randomisation and baseline measures (selection bias) | High risk | Clusters (module clinics) were allocated to treatment, then participants recruitment took place. “We invited all eligible patients referred by their PCP to participate. We excluded patients with nonmelanoma skin cancers, dementia, or psychosis; those enrolled in hospice care; and those unable to complete a written survey in English or Spanish. The PCPs and patients were informed that GMA was assigned to the intervention arm and GMB to the control arm.” Recruitment included consent followed by baseline measures.  Randomisation preceded baseline measures. |
|  | Similarity of baseline outcome measurements across trial arms (selection bias) | Low risk | “There were no significant baseline group differences in physical symptoms, psychological well-being, spiritual well-being, satisfaction, or advance care planning measures.”  No evidence of a difference in baseline outcome measures between trial arms (Table 2). |
|  | Similarity of baseline characteristics across trial arms (selection bias) | Low risk | Little evidence of a difference in baseline characteristics between trial arms (Table 1). |
|  | Blinding of participants and personnel (performance bias) | High risk | Trial was unblinded. |
|  | Blinded outcome assessment (assessor bias) | Unclear | Not enough information to make judgement. |
|  | Incomplete outcome data (attrition bias) | Unclear | Not enough information to make judgement. |
|  | Similarity in attrition across trial arms (attrition bias) | Unclear | “There were no significant group differences between the number of patients who died (*P* = .63) or who failed to complete the study for any other reason (loss of cognitive capacity, loss to follow-up, or refusal to participate).”  Not enough information to make judgement. |
| [173] Rebergen DS, Bruinvels DJ, Bezemer PD, van der Beek AJ, van Mechelen W. Guideline-Based Care of Common Mental Disorders by Occupational Physicians (CO-OP study): A Randomized Controlled Trial. Journal of Occupational & Environmental Medicine. 2009;51:305-12. | Jadad score (0-5; N/A for trial protocols) | 2 | Study was described as randomised, with appropriate utilisation of random allocation. |
|  | Allocation sequence (selection bias) | Low risk | Used computerised randomisation (see protocol article at <https://doi.org/10.1186/1471-2458-7-183>). |
|  | Allocation concealment (selection bias) | Unclear | Used envelopes containing treatment allocation. Protocol article (<https://doi.org/10.1186/1471-2458-7-183>) indicates that envelopes were sealed and sequentially numbered. Not clear if they were opaque.  Not enough information to make judgement. |
|  | Order of randomisation and consent (selection bias) | Low risk | “After a worker had signed informed consent, the OP unsealed a study envelope containing the allocated treatment for the patient.”  Randomisation followed consent. |
|  | Order of randomisation and baseline measures (selection bias) | High risk | “After a worker had signed informed consent, the OP unsealed a study envelope containing the allocated treatment for the patient. In the same consultation the worker received the baseline questionnaires and first treatment satisfaction questionnaire and was asked to return them to the researcher after completion.”  Randomisation preceded baseline measures. |
|  | Similarity of baseline outcome measurements across trial arms (selection bias) | N/A | Outcomes not measured at baseline. |
|  | Similarity of baseline characteristics across trial arms (selection bias) | Low risk | No evidence of a difference in baseline characteristics between trial arms (Table 1). |
|  | Blinding of participants and personnel (performance bias) | High risk | Trial was unblinded. |
|  | Blinded outcome assessment (assessor bias) | N/A | No outcome assessors (participants completed questionnaires). |
|  | Incomplete outcome data (attrition bias) | Unclear | Not enough information to make judgement (for the primary outcome). |
|  | Similarity in attrition across trial arms (attrition bias) | Unclear | Not enough information to make judgement (for the primary outcome). |
| [174] Redhead K, Bradshaw T, Braynion P, Doyle M. An evaluation of the outcomes of psychosocial intervention training for qualified and unqualified nursing staff working in a low-secure mental health unit. Journal of Psychiatric & Mental Health Nursing. 2011;18:59-66. | Jadad score (0-5; N/A for trial protocols) | 1 | Study was described as randomised. |
|  | Allocation sequence (selection bias) | Unclear | Not enough information to make judgement. |
|  | Allocation concealment (selection bias) | Unclear | Not enough information to make judgement. |
|  | Order of randomisation and consent (selection bias) | Unclear | Not enough information to make judgement. |
|  | Order of randomisation and baseline measures (selection bias) | Unclear | Not enough information to make judgement. |
|  | Similarity of baseline outcome measurements across trial arms (selection bias) | Low risk | “There were no significant differences in knowledge, attitude or levels burnout at baseline between the two groups [for qualified and unqualified staff].”  No evidence of a difference in baseline outcome measures between trial arms (Tables 4 and 5). |
|  | Similarity of baseline characteristics across trial arms (selection bias) | Low risk | “There were no significant differences at baseline between the experimental and control groups in terms of age, gender, clinical area or qualification.”  No evidence of a difference in baseline characteristics between trial arms (Table 3). |
|  | Blinding of participants and personnel (performance bias) | High risk | Trial was unblinded. |
|  | Blinded outcome assessment (assessor bias) | High risk | “All assessments were undertaken by the first author (K. R.) who although not blind to group allocation did not work in the same NHS Trust where the study was conducted.”  Outcome assessment was not blind. |
|  | Incomplete outcome data (attrition bias) | Unclear | Not enough information to make judgement. |
|  | Similarity in attrition across trial arms (attrition bias) | Unclear | Not enough information to make judgement. |
| [175] Richards DA, Hill JJ, Gask L, Lovell K, Chew-Graham C, Bower P, et al. Clinical effectiveness of collaborative care for depression in UK primary care (CADET): cluster randomised controlled trial. BMJ August. 2013;24. | Jadad score (0-5; N/A for trial protocols) | 3 | Study was described as randomised, with appropriate utilisation of random allocation, and included a description of withdrawals/dropouts. |
|  | Allocation sequence (selection bias) | Low risk | Used minimisation. |
|  | Allocation concealment (selection bias) | Low risk | “The allocation sequence was concealed from researchers recruiting practices and administered centrally using Minim.”  Allocation was concealed. |
|  | Order of randomisation and consent (selection bias) | High risk | The trial flow chart (Figure) indicates that participant enrolment (including consent) took place after randomisation.  Randomisation preceded consent. |
|  | Order of randomisation and baseline measures (selection bias) | High risk | The trial flow chart (Figure) indicates that participant enrolment took place after randomisation.  Randomisation preceded baseline measures. |
|  | Similarity of baseline outcome measurements across trial arms (selection bias) | Low risk | No evidence of a difference in baseline outcome measures between trial arms (Table 2). |
|  | Similarity of baseline characteristics across trial arms (selection bias) | Low risk | No evidence of a difference in baseline characteristics between trial arms (Table 1). |
|  | Blinding of participants and personnel (performance bias) | High risk | Trial was unblinded. |
|  | Blinded outcome assessment (assessor bias) | Low risk | “Research workers blind to allocation, assessed for eligibility and collected outcome measures using patients’ self report questionnaires to minimise the effect of potential unblinding.”  Outcome assessment was blind. |
|  | Incomplete outcome data (attrition bias) | High risk | Amount of missing data was enough to induce bias in intervention effect estimate. |
|  | Similarity in attrition across trial arms (attrition bias) | Low risk | Similar proportion of participants were lost over follow-up (15% and 14%). |
| [176] Richards D, Lovell K, Gilbody S, Gask L, Torgerson D, Barkham M, et al. Collaborative care for depression in UK primary care: A randomized controlled trial. Psychological Medicine. 2008;38:279-87. | Jadad score (0-5; N/A for trial protocols) | 3 | Study was described as randomised, with appropriate utilisation of random allocation, and included a description of withdrawals/dropouts. |
|  | Allocation sequence (selection bias) | Low risk | Used computerised randomisation. |
|  | Allocation concealment (selection bias) | Low risk | “Allocation was by a remote computer-generated number sequence concealed from researchers and conducted independently...” |
|  | Order of randomisation and consent (selection bias) | High risk | Allocation was “conducted independently after patients were enrolled in the study by research interviewers”. However, it seems this only applied to the individual randomised part of the trial… “To try to reduce the possibility of recruitment bias GPs were given no information about the allocation of their practice.”  Randomisation preceded consent (for the cluster randomised part of the trial). |
|  | Order of randomisation and baseline measures (selection bias) | High risk | Randomisation preceded baseline measures (for the cluster randomised part of the trial; see above) |
|  | Similarity of baseline outcome measurements across trial arms (selection bias) | Low risk | No evidence of a difference in baseline outcome measures between trial arms (Table 1). |
|  | Similarity of baseline characteristics across trial arms (selection bias) | High risk | Some evidence of a difference in baseline characteristics between trial arms (Table 1), in particular between the individual randomised trials arms and the cluster randomised arm. |
|  | Blinding of participants and personnel (performance bias) | High risk | Trial was unblinded. |
|  | Blinded outcome assessment (assessor bias) | Low risk | “All assessments were completed at baseline and 3 months post-randomization by trained assessors blind to participant allocation.”  Outcome assessment was blind. |
|  | Incomplete outcome data (attrition bias) | High risk | Amount of missing data was enough to induce bias in intervention effect estimate. |
|  | Similarity in attrition across trial arms (attrition bias) | High risk | Different proportion of participants were lost over follow-up (15%, 10% and 23%). |
| [177] Robinson LA, Vander Weg MW, Riedel BW, Klesges RC, McLain-Allen B. "Start to stop": results of a randomised controlled trial of a smoking cessation programme for teens. Tobacco Control. 2003;12 Supplement:iv26-iv33. | Jadad score (0-5; N/A for trial protocols) | 1 | Study was described as randomised. |
|  | Allocation sequence (selection bias) | Unclear | Not enough information to make judgement. |
|  | Allocation concealment (selection bias) | Unclear | Not enough information to make judgement. |
|  | Order of randomisation and consent (selection bias) | Unclear | Not enough information to make judgement. |
|  | Order of randomisation and baseline measures (selection bias) | Unclear | Not enough information to make judgement. |
|  | Similarity of baseline outcome measurements across trial arms (selection bias) | Low risk | No evidence of a difference in baseline outcome measures between trial arms (Table 1). |
|  | Similarity of baseline characteristics across trial arms (selection bias) | Low risk | No evidence of a difference in baseline characteristics between trial arms (Table 1). |
|  | Blinding of participants and personnel (performance bias) | High risk | Trial was unblinded. |
|  | Blinded outcome assessment (assessor bias) | Unclear | Not enough information to make judgement. |
|  | Incomplete outcome data (attrition bias) | Unclear | Not enough information to make judgement. |
|  | Similarity in attrition across trial arms (attrition bias) | Unclear | Not enough information to make judgement. |
| [178] Rolland Y, Pillard F, Klapouszczak A, Reynish E, Thomas D, Andrieu S, et al. Exercise program for nursing home residents with Alzheimer's disease: A 1-year randomized, controlled trial. Journal of the American Geriatrics Society. 2007;55:158-65. | Jadad score (0-5; N/A for trial protocols) | 3 | Study was described as randomised, with appropriate utilisation of random allocation, and included a description of withdrawals/dropouts. |
|  | Allocation sequence (selection bias) | Low risk | Used lottery draws. |
|  | Allocation concealment (selection bias) | Unclear | Not enough information to make judgement. |
|  | Order of randomisation and consent (selection bias) | Low risk | “After a screening visit and informed consent, subjects were randomly assigned to the exercise program or to routine medical care.”  Randomisation followed consent. |
|  | Order of randomisation and baseline measures (selection bias) | Unclear | Not enough information to make judgement. |
|  | Similarity of baseline outcome measurements across trial arms (selection bias) | Low risk | No evidence of a difference in baseline outcome measures between trial arms (Table 1). |
|  | Similarity of baseline characteristics across trial arms (selection bias) | Low risk | No evidence of a difference in baseline characteristics between trial arms (Table 1). |
|  | Blinding of participants and personnel (performance bias) | High risk | Trial was unblinded. |
|  | Blinded outcome assessment (assessor bias) | Low risk | “A single geriatrician (AK) who was blinded to the intervention assignment measured outcomes at baseline, 6 months, and 12 months on different days from the intervention.”  Outcome assessment was blind. |
|  | Incomplete outcome data (attrition bias) | High risk | Amount of missing data was enough to induce bias in intervention effect estimate. Also, used inappropriate simple imputation (used last observation carried forward). |
|  | Similarity in attrition across trial arms (attrition bias) | Low risk | Similar proportion of participants were lost over follow-up (16% and 19%). |
| [179] Rondeau V, Allain H, Bakchine S, Bonet P, Brudon F, Chauplannaz G, et al. General practice-based intervenion for suspecting and detecting dementia in France: A cluster randomized controlled trial. Dementia. 2008;7:433-50. | Jadad score (0-5; N/A for trial protocols) | 1 | Study was described as randomised. |
|  | Allocation sequence (selection bias) | Unclear | Not enough information to make judgement. |
|  | Allocation concealment (selection bias) | Unclear | Not enough information to make judgement. |
|  | Order of randomisation and consent (selection bias) | High risk | The trial flow chart (Figure 1) indicates that participant enrolment (including consent) took place after randomisation.  Randomisation preceded consent. |
|  | Order of randomisation and baseline measures (selection bias) | High risk | The trial flow chart (Figure) indicates that participant enrolment took place after randomisation.  Randomisation preceded baseline measures. |
|  | Similarity of baseline outcome measurements across trial arms (selection bias) | N/A | “The primary outcome was suspicion of dementia by GPs. The secondary outcome was accurate detection of dementia by the GPs.” Outcomes not measured at baseline. |
|  | Similarity of baseline characteristics across trial arms (selection bias) | Low risk | No evidence of a difference in baseline characteristics between trial arms (Table 1). |
|  | Blinding of participants and personnel (performance bias) | High risk | Trial was unblinded. |
|  | Blinded outcome assessment (assessor bias) | High risk | “The primary outcome was suspicion of dementia by GPs. The secondary outcome was accurate detection of dementia by the GPs.” GPs were not blinded because they knew about the intervention, which they provided. |
|  | Incomplete outcome data (attrition bias) | Unclear | Not enough information to make judgement. |
|  | Similarity in attrition across trial arms (attrition bias) | Unclear | Not enough information to make judgement. |
| [180] Ross R, Lam M, Blair SN, Church TS, Godwin M, Hotz SB, et al. Trial of Prevention and Reduction of Obesity Through Active Living in Clinical Settings: A Randomized Controlled Trial. Archives of Internal Medicine. 2012;172:414-24. | Jadad score (0-5; N/A for trial protocols) | 3 | Study was described as randomised, with appropriate utilisation of random allocation, and included a description of withdrawals/dropouts. |
|  | Allocation sequence (selection bias) | Low risk | Used computerised randomisation. |
|  | Allocation concealment (selection bias) | Unclear | Not enough information to make judgement. |
|  | Order of randomisation and consent (selection bias) | Low risk | “Participants provided written informed consent before participation.” In addition, the trial flow chart (Figure 1) shows that consent preceded randomisation.  Randomisation followed consent. |
|  | Order of randomisation and baseline measures (selection bias) | Low risk | “…baseline data were collected before randomization.”  Randomisation followed baseline measures. |
|  | Similarity of baseline outcome measurements across trial arms (selection bias) | Low risk | No evidence of a difference in baseline outcome measures between trial arms (Tables 1 and 2). |
|  | Similarity of baseline characteristics across trial arms (selection bias) | Low risk | No evidence of a difference in baseline characteristics between trial arms (Table 1). |
|  | Blinding of participants and personnel (performance bias) | High risk | Trial was unblinded. |
|  | Blinded outcome assessment (assessor bias) | Unclear | Not enough information to make judgement. |
|  | Incomplete outcome data (attrition bias) | High risk | Amount of missing data was enough to induce bias in intervention effect estimate. |
|  | Similarity in attrition across trial arms (attrition bias) | High risk | Different proportion of participants were lost over follow-up (15% and 24%). |
| [181] Roy-Byrne PP, Craske MG, Stein MB, Sullivan G, Bystritsky A, Katon W, et al. A Randomized Effectiveness Trial of Cognitive-Behavioral Therapy and Medication for Primary Care Panic Disorder. Archives of General Psychiatry. 2005;62:290-8. | Jadad score (0-5; N/A for trial protocols) | 1 | Study was described as randomised, with inappropriate utilisation of random allocation, and included a description of withdrawals/dropouts. |
|  | Allocation sequence (selection bias) | High risk | “The interviewer, blind to the randomization scheme, then gave eligible subjects’ names to a study coordinator who randomized subjects using alternating assignment, stratified within site by comorbid major depression and referral status (referred vs screened).”  Allocation was not random. |
|  | Allocation concealment (selection bias) | High risk | Used alternation.  Allocation was not concealed. |
|  | Order of randomisation and consent (selection bias) | Low risk | The trial flow chart (Figure 1) shows that consent preceded randomisation.  Randomisation followed consent. |
|  | Order of randomisation and baseline measures (selection bias) | Unclear | Not enough information to make judgement. |
|  | Similarity of baseline outcome measurements across trial arms (selection bias) | Low risk | “Intervention and usual care groups were comparable at baseline on all measures.”  No evidence of a difference in baseline outcome measures between trial arms (also see Tables 1 and 2). |
|  | Similarity of baseline characteristics across trial arms (selection bias) | Low risk | “Intervention and usual care groups were comparable at baseline on all measures.”  No evidence of a difference in baseline characteristics between trial arms (also see Table 1). |
|  | Blinding of participants and personnel (performance bias) | High risk | Trial was unblinded. |
|  | Blinded outcome assessment (assessor bias) | Low risk | “Assessments were derived from telephone interviewer administered questionnaires, queried by interviewers blind to subject intervention status.”  Outcome assessment was blind. |
|  | Incomplete outcome data (attrition bias) | High risk | Amount of missing data was enough to induce bias in intervention effect estimate. |
|  | Similarity in attrition across trial arms (attrition bias) | Low risk | Similar proportion of participants were lost over follow-up (24% and 22%). |
| [182] Russell AJ, Jassi A, Fullana MA, Mack H, Johnston K, Heyman I, et al. Cognitive behavior therapy for comorbid obsessive-compulsive disorder in high-functioning autism spectrum disorders: A randomized controlled trial. Depression and Anxiety. 2013;30:697-708. | Jadad score (0-5; N/A for trial protocols) | 3 | Study was described as randomised, with appropriate utilisation of random allocation, and included a description of withdrawals/dropouts. |
|  | Allocation sequence (selection bias) | Low risk | Used table of random numbers. |
|  | Allocation concealment (selection bias) | Unclear | Not enough information to make judgement. |
|  | Order of randomisation and consent (selection bias) | Low risk | “Seventeen (22.6%) of these 75 individuals did not meet eligibility criteria for the study (see Fig. 1), 2 people were eligible but geography prevented participation and 10 people did not consent to take part. Twenty-three people were randomized to each of the two treatment groups (AM and CBT) with 20 treatment completers in each group.” In addition, the trial flow chart (Figure 1) shows that consent preceded randomisation.  Randomisation followed consent. |
|  | Order of randomisation and baseline measures (selection bias) | Unclear | Not enough information to make judgement. |
|  | Similarity of baseline outcome measurements across trial arms (selection bias) | Low risk | No evidence of a difference in baseline outcome measures between trial arms (Table 2). |
|  | Similarity of baseline characteristics across trial arms (selection bias) | Low risk | “The treatment groups did not differ with respect to gender distribution (AM group 69.6%, CBT group 82.6% male), or the proportion of those under the age of 18 (AM group n=6 (26.1%), CBT group n=3(13%) youth protocol).”  No evidence of a difference in baseline characteristics between trial arms. |
|  | Blinding of participants and personnel (performance bias) | High risk | Trial was unblinded. |
|  | Blinded outcome assessment (assessor bias) | Low risk | “Symptom ratings were made by assessors blind to treatment group prior to commencing treatment.”  Outcome assessment was blind. |
|  | Incomplete outcome data (attrition bias) | High risk | Amount of missing data was enough to induce bias in intervention effect estimate. |
|  | Similarity in attrition across trial arms (attrition bias) | Low risk | Similar proportion of participants were lost over follow-up (22% and 26%). |
| [183] Safren SA, Sprich S, Mimiaga MJ, Surman C, Knouse L, Groves M, et al. Cognitive Behavioral Therapy vs Relaxation With Educational Support for Medication-Treated Adults With ADHD and Persistent Symptoms: A Randomized Controlled Trial. JAMA. 2010;304:875-80. | Jadad score (0-5; N/A for trial protocols) | 3 | Study was described as randomised, with appropriate utilisation of random allocation, and included a description of withdrawals/dropouts. |
|  | Allocation sequence (selection bias) | Low risk | Used coin tossing. |
|  | Allocation concealment (selection bias) | Unclear | Not enough information to make judgement. |
|  | Order of randomisation and consent (selection bias) | Low risk | The trial flow chart (Figure 1) shows that consent preceded randomisation.  Randomisation followed consent. |
|  | Order of randomisation and baseline measures (selection bias) | Unclear | Not enough information to make judgement. |
|  | Similarity of baseline outcome measurements across trial arms (selection bias) | Low risk | No evidence of a difference in baseline outcome measures between trial arms (Table 2). |
|  | Similarity of baseline characteristics across trial arms (selection bias) | Low risk | No evidence of a difference in baseline characteristics between trial arms (Table 1). |
|  | Blinding of participants and personnel (performance bias) | High risk | Trial was unblinded. |
|  | Blinded outcome assessment (assessor bias) | Low risk | “The assessor was blinded to treatment condition assignment.”  Outcome assessment was blind. |
|  | Incomplete outcome data (attrition bias) | Low risk | Amount of missing data was enough to induce bias in intervention effect estimate. |
|  | Similarity in attrition across trial arms (attrition bias) | High risk | Different proportion of participants were lost over follow-up (12% and 26%). |
| [184] Saitz R, Cheng D, Winter M, Kim T, Meli S, Allensworth-Davies D, et al. Chronic Care Management for Dependence on Alcohol and Other Drugs: The AHEAD Randomized Trial. JAMA. 2013;310:1156-67. | Jadad score (0-5; N/A for trial protocols) | 2 | Study was described as randomised, and included a description of withdrawals/dropouts. |
|  | Allocation sequence (selection bias) | Unclear | Not enough information to make judgement. |
|  | Allocation concealment (selection bias) | Low risk | Used central allocation (via website).  Allocation was concealed. |
|  | Order of randomisation and consent (selection bias) | Low risk | The trial flow chart (Figure) shows that consent preceded randomisation.  Randomisation followed consent. |
|  | Order of randomisation and baseline measures (selection bias) | Low risk | “After the baseline assessment and via a central secure website (providing allocation concealment), participants were randomly assigned in a 1:1 ratio to receive either the CCM intervention or usual primary care.”  Randomisation followed baseline measures. |
|  | Similarity of baseline outcome measurements across trial arms (selection bias) | Low risk | No evidence of a difference in baseline outcome measures between trial arms (Table 1). |
|  | Similarity of baseline characteristics across trial arms (selection bias) | Low risk | No evidence of a difference in baseline characteristics between trial arms (Table 1). |
|  | Blinding of participants and personnel (performance bias) | High risk | Trial was unblinded. |
|  | Blinded outcome assessment (assessor bias) | Unclear | Not enough information to make judgement. |
|  | Incomplete outcome data (attrition bias) | Low risk | Amount of missing data was low and balanced across trial arms. |
|  | Similarity in attrition across trial arms (attrition bias) | Low risk | Different proportion of participants were lost over follow-up (4% and 7%). |
| [185] Samet JH, Raj A, Cheng DM, Blokhina E, Bridden C, Chaisson CE, et al. HERMITAGE-a randomized controlled trial to reduce sexually transmitted infections and HIV risk behaviors among HIV-infected Russian drinkers. Addiction. 2015;110:80-90. | Jadad score (0-5; N/A for trial protocols) | 2 | Study was described as randomised, and included a description of withdrawals/dropouts. |
|  | Allocation sequence (selection bias) | Unclear | Not enough information to make judgement. |
|  | Allocation concealment (selection bias) | Unclear | Not enough information to make judgement. |
|  | Order of randomisation and consent (selection bias) | Low risk | The trial flow chart (Figure 1) shows that consent preceded randomisation.  Randomisation followed consent. |
|  | Order of randomisation and baseline measures (selection bias) | Low risk | “The baseline assessment occurred prior to randomization.”  Randomisation followed baseline measures. |
|  | Similarity of baseline outcome measurements across trial arms (selection bias) | Low risk | No evidence of a difference in baseline outcome measures between trial arms (Table 2). |
|  | Similarity of baseline characteristics across trial arms (selection bias) | Low risk | No evidence of a difference in baseline characteristics between trial arms (Table 1). |
|  | Blinding of participants and personnel (performance bias) | High risk | Trial was unblinded. |
|  | Blinded outcome assessment (assessor bias) | Low risk | “RAs conducting interview assessments were blinded to the individual’s randomization status.” |
|  | Incomplete outcome data (attrition bias) | High risk | Amount of missing data was enough to induce bias in intervention effect estimate. |
|  | Similarity in attrition across trial arms (attrition bias) | Low risk | Similar proportion of participants were lost over follow-up (31% and 28%). |
| [186] Samuel-Hodge CD, Keyserling TC, Park S, Johnston LF, Gizlice Z, Bangdiwala SI. A Randomized Trial of a Church-Based Diabetes Self-management Program for African Americans With Type 2 Diabetes. Diabetes Educator May/June. 2009;35:439-54. | Jadad score (0-5; N/A for trial protocols) | 3 | Study was described as randomised, with appropriate utilisation of random allocation, and included a description of withdrawals/dropouts. |
|  | Allocation sequence (selection bias) | Low risk | Used computer-generated random numbers. |
|  | Allocation concealment (selection bias) | Unclear | “A research assistant accomplished randomization by opening the next envelope from a set of sequentially numbered sealed envelopes containing study group assignment as determined by random numbers generated by a statistical consultant using a personal computer.” Not clear if envelopes were opaque.  Not enough information to make judgement. |
|  | Order of randomisation and consent (selection bias) | Low risk | The trial flow chart (Figure 1) shows that consent preceded randomisation.  Randomisation followed consent. |
|  | Order of randomisation and baseline measures (selection bias) | Low risk | “Churches were randomized after required baseline data were collected from all participants within a church.”  Randomisation followed baseline measures. |
|  | Similarity of baseline outcome measurements across trial arms (selection bias) | Low risk | No evidence of a difference in baseline outcome measures between trial arms (Table 2). |
|  | Similarity of baseline characteristics across trial arms (selection bias) | Low risk | Little evidence of a difference in baseline characteristics between trial arms (Table 1). |
|  | Blinding of participants and personnel (performance bias) | High risk | Trial was unblinded. |
|  | Blinded outcome assessment (assessor bias) | Low risk | “…personnel conducting follow-up interviews were masked to the participants’ study group.”  Outcome assessment was blind. |
|  | Incomplete outcome data (attrition bias) | High risk | Amount of missing data was enough to induce bias in intervention effect estimate. Also, used inappropriate simple imputation (used last observation carried forward). |
|  | Similarity in attrition across trial arms (attrition bias) | Low risk | Similar proportion of participants were lost over follow-up (14% and 18%). |
| [187] Sandgren AK, McCaul KD. Short-Term Effects of Telephone Therapy for Breast Cancer Patients. Health Psychology. 2003;22:310-5. | Jadad score (0-5; N/A for trial protocols) | 1 | Study was described as randomised. |
|  | Allocation sequence (selection bias) | Unclear | Not enough information to make judgement. |
|  | Allocation concealment (selection bias) | Unclear | Not enough information to make judgement. |
|  | Order of randomisation and consent (selection bias) | Low risk | “Informed consent included a 15–20 min interview in which the patients learned about the study purpose and that they would be randomly assigned either to a nurse phone intervention condition or to standard care.”  Randomisation followed consent. |
|  | Order of randomisation and baseline measures (selection bias) | Unclear | Not enough information to make judgement. |
|  | Similarity of baseline outcome measurements across trial arms (selection bias) | Low risk | No evidence of a difference in baseline outcome measures between trial arms (Table 2). |
|  | Similarity of baseline characteristics across trial arms (selection bias) | Unclear | Not enough information to make judgement. |
|  | Blinding of participants and personnel (performance bias) | High risk | Trial was unblinded. |
|  | Blinded outcome assessment (assessor bias) | Unclear | Not enough information to make judgement. |
|  | Incomplete outcome data (attrition bias) | Unclear | Not enough information to make judgement. |
|  | Similarity in attrition across trial arms (attrition bias) | Unclear | Not enough information to make judgement. |
| [188] Schiller KR, Luo X, Anderson AJ, Jensen JA, Allen SS, Hatsukami DK. Comparing an Immediate Cessation Versus Reduction Approach to Smokeless Tobacco Cessation. Nicotine & Tobacco Research. 2011;14:902-9. | Jadad score (0-5; N/A for trial protocols) | 1 | Study was described as randomised. |
|  | Allocation sequence (selection bias) | Unclear | Not enough information to make judgement. |
|  | Allocation concealment (selection bias) | Unclear | Not enough information to make judgement. |
|  | Order of randomisation and consent (selection bias) | High risk | “Interested and eligible subjects were assigned a randomization number at this first phone contact , and the appropriate study description (reduction or immediate cessation [the treatment conditions]) was given to the subject. Subjects were asked to come into the research clinic for an orientation visit to obtain informed consent and engage in more thorough screening.”  Randomisation followed consent. |
|  | Order of randomisation and baseline measures (selection bias) | Unclear | Not enough information to make judgement. |
|  | Similarity of baseline outcome measurements across trial arms (selection bias) | Unclear | Not enough information to make judgement. |
|  | Similarity of baseline characteristics across trial arms (selection bias) | Unclear | Not enough information to make judgement. |
|  | Blinding of participants and personnel (performance bias) | High risk | Trial was unblinded. |
|  | Blinded outcome assessment (assessor bias) | Unclear | Not enough information to make judgement. |
|  | Incomplete outcome data (attrition bias) | High risk | Amount of missing data was enough to induce bias in intervention effect estimate. |
|  | Similarity in attrition across trial arms (attrition bias) | High risk | Different proportion of participants were lost over follow-up (46% and 53%). |
| [189] Schmidt U, Oldershaw A, Jichi F, Sternheim L, Startup H, McIntosh V, et al. Out-patient psychological therapies for adults with anorexia nervosa: randomised controlled trial. British Journal of Psychiatry. 2012;201:392-9. | Jadad score (0-5; N/A for trial protocols) | 3 | Study was described as randomised, with appropriate utilisation of random allocation, and included a description of withdrawals/dropouts. |
|  | Allocation sequence (selection bias) | Low risk | Used computerised randomisation |
|  | Allocation concealment (selection bias) | Low risk | “Treatment assignment codes were contained in numbered sealed opaque envelopes held by the independent researcher.”  Allocation was concealed. |
|  | Order of randomisation and consent (selection bias) | Low risk | The trial flow chart (Figure 1) shows that consent preceded randomisation.  Randomisation followed consent. |
|  | Order of randomisation and baseline measures (selection bias) | Low risk | The pre-randomisation (baseline) measure of the outcome was included as a covariate as was the stratification factor baseline BMI.  Randomisation followed baseline measures. |
|  | Similarity of baseline outcome measurements across trial arms (selection bias) | Low risk | No evidence of a difference in baseline outcome measures between trial arms (Table 1). |
|  | Similarity of baseline characteristics across trial arms (selection bias) | Low risk | Little evidence of a difference in baseline characteristics between trial arms (Table 1). |
|  | Blinding of participants and personnel (performance bias) | High risk | Trial was unblinded. |
|  | Blinded outcome assessment (assessor bias) | Low risk | “Outcome assessments were conducted by two assessors  masked to treatment allocation.”  Outcome assessment was blind. |
|  | Incomplete outcome data (attrition bias) | High risk | Amount of missing data was enough to induce bias in intervention effect estimate. In addition, loss to follow-up was different between trial arms. |
|  | Similarity in attrition across trial arms (attrition bias) | High risk | Different proportion of participants were lost over follow-up (6% and 0% for EDE; 12% and 27% for BMI). |
| [190] Schneider JK, Cook JH, Luke DA. Unexpected effects of cognitive-behavioural therapy on self-reported exercise behaviour and functional outcomes in older adults. Age & Ageing. 2011;40:163-8. | Jadad score (0-5; N/A for trial protocols) | 3 | Study was described as randomised, with appropriate utilisation of random allocation, and included a description of withdrawals/dropouts. |
|  | Allocation sequence (selection bias) | Low risk | Used computerised randomisation. |
|  | Allocation concealment (selection bias) | Unclear | Not enough information to make judgement. |
|  | Order of randomisation and consent (selection bias) | Low risk | Eight hundred and forty-two older adults telephoned; of which, 311 refused for personal reasons, such as not interested, too far to travel and involved too much time; 189 did not meet the inclusion criteria… Using computer-generated randomisation allocation tables, we randomised the subjects to three groups: men, women and couples.  Randomisation followed consent. |
|  | Order of randomisation and baseline measures (selection bias) | Unclear | Not enough information to make judgement. |
|  | Similarity of baseline outcome measurements across trial arms (selection bias) | Low risk | No evidence of a difference in baseline outcome measures between trial arms (Table 2). |
|  | Similarity of baseline characteristics across trial arms (selection bias) | Low risk | No evidence of a difference in baseline characteristics between trial arms (Table 1). |
|  | Blinding of participants and personnel (performance bias) | High risk | Trial was unblinded. |
|  | Blinded outcome assessment (assessor bias) | Low risk | “Blinded data collectors measured follow-up exercise behaviour and functional outcomes at 3-month intervals.”  Outcome assessment was blind. |
|  | Incomplete outcome data (attrition bias) | Low risk | Amount of missing data was low and balanced across trial arms. |
|  | Similarity in attrition across trial arms (attrition bias) | Low risk | Similar proportion of participants were lost over follow-up (6%, 5% and 7%). |
| [191] Schneider RH, Grim CE, Rainforth MV, Kotchen T, Nidich SI, Gaylord-King C, et al. Stress Reduction in the Secondary Prevention of Cardiovascular Disease: Randomized, Controlled Trial of Transcendental Meditation and Health Education in Blacks. Circulation: Cardiovascular Quality & Outcomes. 2012;5:750-8. | Jadad score (0-5; N/A for trial protocols) | 2 | Study was described as randomised, and included a description of withdrawals/dropouts. |
|  | Allocation sequence (selection bias) | Unclear | Not enough information to make judgement. |
|  | Allocation concealment (selection bias) | Low risk | Allocation performed remotely. “Random allocation was performed by the study biostatistician who concealed the allocation schedule and conveyed the assignments to the study coordinator”  Allocation was concealed. |
|  | Order of randomisation and consent (selection bias) | Low risk | The trial flow chart (Figure 1) shows that consent preceded randomisation.  Randomisation followed consent. |
|  | Order of randomisation and baseline measures (selection bias) | Unclear | Not enough information to make judgement. |
|  | Similarity of baseline outcome measurements across trial arms (selection bias) | Low risk | No evidence of a difference in baseline outcome measures between trial arms (Table 1). |
|  | Similarity of baseline characteristics across trial arms (selection bias) | Low risk | Little evidence of a difference in baseline characteristics between trial arms (Table 1). |
|  | Blinding of participants and personnel (performance bias) | High risk | Trial was unblinded. |
|  | Blinded outcome assessment (assessor bias) | Low risk | “Investigators, data collectors, and data management staff were  blinded to group assignment.”  Outcome assessment was blind. |
|  | Incomplete outcome data (attrition bias) | High risk | Amount of missing data was enough to induce bias in intervention effect estimate. |
|  | Similarity in attrition across trial arms (attrition bias) | Low risk | Similar proportion of participants were lost over follow-up (23% and 25%). |
| [192] Schonert-Reichl KA, Oberle E, Lawlor MS, Abbott D, Thomson K, Oberlander TF, et al. Enhancing cognitive and social-emotional development through a simple-to-administer mindfulness-based school program for elementary school children: A randomized controlled trial. Developmental Psychology. 2015;51:52-66. | Jadad score (0-5; N/A for trial protocols) | 2 | Study was described as randomised, with appropriate utilisation of random allocation. |
|  | Allocation sequence (selection bias) | Low risk | Used coin tossing. |
|  | Allocation concealment (selection bias) | Unclear | Not enough information to make judgement. |
|  | Order of randomisation and consent (selection bias) | Unclear | Not enough information to make judgement. |
|  | Order of randomisation and baseline measures (selection bias) | Low risk | “After the collection of baseline data, randomization was done by a coin flip…”  Randomisation followed baseline measures. |
|  | Similarity of baseline outcome measurements across trial arms (selection bias) | Low risk | Little evidence of a difference in baseline outcome measures between trial arms (Tables 2, 4 and 5). |
|  | Similarity of baseline characteristics across trial arms (selection bias) | Low risk | No evidence of a difference in baseline characteristics between trial arms (Table 1). |
|  | Blinding of participants and personnel (performance bias) | High risk | Trial was unblinded. |
|  | Blinded outcome assessment (assessor bias) | Low risk | “Trained research assistants, blind to teacher and student study conditions, administered all assessments and collected diurnal cortisol samples from students at pre- and posttest.”  Outcome assessment was blind. |
|  | Incomplete outcome data (attrition bias) | Unclear | Not enough information to make judgement. |
|  | Similarity in attrition across trial arms (attrition bias) | Unclear | Not enough information to make judgement. |
| [193] Sells D, Davidson L, Jewell C, Falzer P, Rowe M. The Treatment Relationship in Peer-Based and Regular Case Management for Clients With Severe Mental Illness. Psychiatric Services. 2006;57:1179-84. | Jadad score (0-5; N/A for trial protocols) | 1 | Study was described as randomised. |
|  | Allocation sequence (selection bias) | Unclear | Not enough information to make judgement. |
|  | Allocation concealment (selection bias) | Unclear | Not enough information to make judgement. |
|  | Order of randomisation and consent (selection bias) | Low risk | “If consent was granted, investigators randomly assigned participants to either the experimental (peer provider) or control (regular treatment) condition.”  Randomisation followed consent. |
|  | Order of randomisation and baseline measures (selection bias) | Unclear | Not enough information to make judgement. |
|  | Similarity of baseline outcome measurements across trial arms (selection bias) | Unclear | Not enough information to make judgement. |
|  | Similarity of baseline characteristics across trial arms (selection bias) | Unclear | Not enough information to make judgement. |
|  | Blinding of participants and personnel (performance bias) | High risk | Trial was unblinded. |
|  | Blinded outcome assessment (assessor bias) | Unclear | Not enough information to make judgement. |
|  | Incomplete outcome data (attrition bias) | High risk | Amount of missing data was enough to induce bias in intervention effect estimate. |
|  | Similarity in attrition across trial arms (attrition bias) | High risk | Different proportion of participants were lost over follow-up (22% and 37%). |
| [194] Sensky T, Turkington D, Kingdon D, Scott JL, Scott JM, Siddle R, et al. A Randomized Controlled Trial of Cognitive-Behavioral Therapy for Persistent Symptoms in Schizophrenia Resistant to Medication. Archives of General Psychiatry. 2000;57:165-72. | Jadad score (0-5; N/A for trial protocols) | 2 | Study was described as randomised, and included a description of withdrawals/dropouts. |
|  | Allocation sequence (selection bias) | Unclear | Not enough information to make judgement. |
|  | Allocation concealment (selection bias) | Unclear | Not enough information to make judgement. |
|  | Order of randomisation and consent (selection bias) | Low risk | “After written informed consent was obtained, patients were then assigned to one of the treatment arms using simple randomization.”  Randomisation followed consent. |
|  | Order of randomisation and baseline measures (selection bias) | Unclear | Not enough information to make judgement. |
|  | Similarity of baseline outcome measurements across trial arms (selection bias) | Low risk | Little evidence of a difference in baseline outcome measures between trial arms (Table 2). |
|  | Similarity of baseline characteristics across trial arms (selection bias) | Low risk | No evidence of a difference in baseline characteristics between trial arms (Table 1). |
|  | Blinding of participants and personnel (performance bias) | High risk | Trial was unblinded. |
|  | Blinded outcome assessment (assessor bias) | Low risk | “The assessors were independent of the randomization procedure and remained blind to each patient's assigned group throughout the study.”  Outcome assessment was blind. |
|  | Incomplete outcome data (attrition bias) | Low risk | “Data at the 3 points (baseline, treatment outcome, and 9-month follow-up evaluation) were collected for all the patients randomized, except that SANS data were not collected for 2 patients from Newcastle.”  Amount of missing outcome data was very low. |
|  | Similarity in attrition across trial arms (attrition bias) | Low risk | Amount of missing outcome data was very low (missing two participants) and therefore balanced across trial arms. |
| [195] Sharpe H, Schober I, Treasure J, Schmidt U. Feasibility, acceptability and efficacy of a school-based prevention programme for eating disorders: cluster randomised controlled trial. British Journal of Psychiatry. 2013;203:428-35. | Jadad score (0-5; N/A for trial protocols) | 3 | Study was described as randomised, with appropriate utilisation of random allocation, and included a description of withdrawals/dropouts. |
|  | Allocation sequence (selection bias) | Low risk | Used online random number generator. |
|  | Allocation concealment (selection bias) | Unclear | Not enough information to make judgement. |
|  | Order of randomisation and consent (selection bias) | High risk | “Informed consent for participants was obtained from all participants’ parents/carers following randomisation.”  Randomisation preceded consent. |
|  | Order of randomisation and baseline measures (selection bias) | High risk | “In addition, participants provided written assent post-randomisation, when completing the pre-intervention questionnaire measures.”  Randomisation preceded baseline measures. |
|  | Similarity of baseline outcome measurements across trial arms (selection bias) | Low risk | Little evidence of a difference in baseline outcome measures between trial arms (Table 1). |
|  | Similarity of baseline characteristics across trial arms (selection bias) | Low risk | No evidence of a difference in baseline characteristics between trial arms (Table 1). |
|  | Blinding of participants and personnel (performance bias) | High risk | Trial was unblinded. |
|  | Blinded outcome assessment (assessor bias) | N/A | No outcome assessors (participants completed questionnaires). |
|  | Incomplete outcome data (attrition bias) | Low risk | Amount of missing data was low and balanced across trial arms. |
|  | Similarity in attrition across trial arms (attrition bias) | Low risk | Similar proportion of participants were lost over follow-up (5% and 8%). |
| [196] Shellman JM, Mokel M, Hewitt N. The Effects of Integrative Reminiscence on Depressive Symptoms in Older African Americans. Western Journal of Nursing Research. 2009;31:772-86. | Jadad score (0-5; N/A for trial protocols) | 1 | Study was described as randomised. |
|  | Allocation sequence (selection bias) | Unclear | Not enough information to make judgement. |
|  | Allocation concealment (selection bias) | Unclear | Not enough information to make judgement. |
|  | Order of randomisation and consent (selection bias) | Low risk | “The consent form was read to each participant and signed before data collection began. Once data had been collected for Time 1 (pretest), the participants were randomized to the reminiscence (experimental), attention control (health education), or control group.”  Randomisation followed consent. |
|  | Order of randomisation and baseline measures (selection bias) | Low risk | “The consent form was read to each participant and signed before data collection began. Once data had been collected for Time 1 (pretest), the participants were randomized to the reminiscence (experimental), attention control (health education), or control group.”  Randomisation followed baseline measures. |
|  | Similarity of baseline outcome measurements across trial arms (selection bias) | Low risk | No evidence of a difference in baseline outcome measures between trial arms (Table 2). |
|  | Similarity of baseline characteristics across trial arms (selection bias) | Unclear | Not enough information to make judgement. |
|  | Blinding of participants and personnel (performance bias) | High risk | Trial was unblinded. |
|  | Blinded outcome assessment (assessor bias) | Unclear | Not enough information to make judgement. |
|  | Incomplete outcome data (attrition bias) | Unclear | Not enough information to make judgement. |
|  | Similarity in attrition across trial arms (attrition bias) | Unclear | Not enough information to make judgement. |
| [197] Shemilt I, Harvey I, Shepstone L, Swift L, Reading R, Mugford M, et al. A national evaluation of school breakfast clubs: evidence from a cluster randomized controlled trial and an observational analysis. Child: Care, Health & Development. 2004;30:413-27. | Jadad score (0-5; N/A for trial protocols) | 2 | Study was described as randomised, and included a description of withdrawals/dropouts. |
|  | Allocation sequence (selection bias) | Unclear | Not enough information to make judgement. |
|  | Allocation concealment (selection bias) | Unclear | Not enough information to make judgement. |
|  | Order of randomisation and consent (selection bias) | High risk | “Following randomization, institutional consent for pupils’ participation was sought.”  Randomisation preceded consent. |
|  | Order of randomisation and baseline measures (selection bias) | High risk | The trial flow chart (Figure 1) shows that enrolment followed randomisation.  Randomisation preceded baseline measures. |
|  | Similarity of baseline outcome measurements across trial arms (selection bias) | Low risk | No evidence of a difference in baseline outcome measures between trial arms (Table 1). |
|  | Similarity of baseline characteristics across trial arms (selection bias) | Low risk | No evidence of a difference in baseline characteristics between trial arms (Table 1). |
|  | Blinding of participants and personnel (performance bias) | High risk | Trial was unblinded. |
|  | Blinded outcome assessment (assessor bias) | Unclear | Not enough information to make judgement. |
|  | Incomplete outcome data (attrition bias) | High risk | Amount of missing data was enough to induce bias in intervention effect estimate. |
|  | Similarity in attrition across trial arms (attrition bias) | Low risk | Similar proportion of participants were lost over follow-up (35% and 31%). |
| [198] Shernoff ES, Kratochwill TR. Transporting an Evidence-Based Classroom Management Program for Preschoolers With Disruptive Behavior Problems to a School: An Analysis of Implementation, Outcomes, and Contextual Variables. School Psychology Quarterly. 2007;22:449-72. | Jadad score (0-5; N/A for trial protocols) | 1 | Study was described as randomised. |
|  | Allocation sequence (selection bias) | Unclear | Not enough information to make judgement. |
|  | Allocation concealment (selection bias) | Unclear | Not enough information to make judgement. |
|  | Order of randomisation and consent (selection bias) | Unclear | Not enough information to make judgement. |
|  | Order of randomisation and baseline measures (selection bias) | Unclear | Not enough information to make judgement. |
|  | Similarity of baseline outcome measurements across trial arms (selection bias) | Unclear | Not enough information to make judgement. |
|  | Similarity of baseline characteristics across trial arms (selection bias) | Unclear | Not enough information to make judgement. |
|  | Blinding of participants and personnel (performance bias) | High risk | Trial was unblinded. |
|  | Blinded outcome assessment (assessor bias) | Low risk | “Undergraduate students blind to training conditions, goals, and hypotheses of the study conducted student observations.”  Outcome assessment was blind. |
|  | Incomplete outcome data (attrition bias) | Unclear | Not enough information to make judgement. |
|  | Similarity in attrition across trial arms (attrition bias) | Unclear | Not enough information to make judgement. |
| [199] Simon GE, Ludman EJ, Unutzer J, Bauer MS, Operskalski B, Rutter C. Randomized trial of a population-based care program for people with bipolar disorder. Psychological Medicine. 2005;35:13-24. | Jadad score (0-5; N/A for trial protocols) | 2 | Study was described as randomised, with appropriate utilisation of random allocation. |
|  | Allocation sequence (selection bias) | Low risk | Used computer-generated random numbers. |
|  | Allocation concealment (selection bias) | Low risk | Used central allocation (allocated revealed via phone).  Allocation was concealed. |
|  | Order of randomisation and consent (selection bias) | Low risk | “…consenting participants were randomly assigned to continued usual care or usual care plus a multi-component intervention program.”  Randomisation followed consent. |
|  | Order of randomisation and baseline measures (selection bias) | Low risk | “Participants provided written informed consent at three stages: prior to the baseline assessment, prior to enrollment in the randomized trial, and (for those in the intervention group) prior to the initial intervention visit.”  Randomisation followed baseline measures. |
|  | Similarity of baseline outcome measurements across trial arms (selection bias) | Low risk | No evidence of a difference in baseline outcome measures between trial arms (Table 1). |
|  | Similarity of baseline characteristics across trial arms (selection bias) | Low risk | No evidence of a difference in baseline characteristics between trial arms (Table 1). |
|  | Blinding of participants and personnel (performance bias) | High risk | Trial was unblinded. |
|  | Blinded outcome assessment (assessor bias) | Low risk | “All participants were contacted for blinded in-person follow-up assessments every 3 months.”  Outcome assessment was blind. |
|  | Incomplete outcome data (attrition bias) | Unclear | Not enough information to make judgement. |
|  | Similarity in attrition across trial arms (attrition bias) | Unclear | Unclear how many participants were followed up at the final assessment. Not enough information to make judgement. |
| [200] Slade M, McCrone P, Kuipers E, Leese M, Cahill S, Parabiaghi A, et al. Use of standardised outcome measures in adult mental health services: Randomised controlled trial. British Journal of Psychiatry. 2006;189:330-6. | Jadad score (0-5; N/A for trial protocols) | 3 | Study was described as randomised, with appropriate utilisation of random allocation, and included a description of withdrawals/dropouts. |
|  | Allocation sequence (selection bias) | Low risk | Used computerised randomisation. |
|  | Allocation concealment (selection bias) | Low risk | “Allocation was concealed until the intervention was assigned.”  Allocation was concealed. |
|  | Order of randomisation and consent (selection bias) | Low risk | The trial flow chart (Figure 1) shows that consent preceded randomisation.  Randomisation followed consent. |
|  | Order of randomisation and baseline measures (selection bias) | Low risk | “Following baseline assessment, patients were allocated by an independent statistician who was blind to the results of the baseline assessment.”  Randomisation followed baseline measures. |
|  | Similarity of baseline outcome measurements across trial arms (selection bias) | Low risk | Little evidence of a difference in baseline outcome measures between trial arms (Table 1). |
|  | Similarity of baseline characteristics across trial arms (selection bias) | Low risk | No evidence of a difference in baseline characteristics between trial arms (Table 1). |
|  | Blinding of participants and personnel (performance bias) | High risk | Trial was unblinded. |
|  | Blinded outcome assessment (assessor bias) | Low risk | “Researchers conducting the follow-up interviews were partially blind – they guessed allocation status correctly for 38% of staff and for 68% of patients.”  Outcome assessment was blind. |
|  | Incomplete outcome data (attrition bias) | High risk | Amount of missing data was enough to induce bias in intervention effect estimate. |
|  | Similarity in attrition across trial arms (attrition bias) | High risk | Different proportion of participants were lost over follow-up (8% and 17%). |
| [201] Speca M, Carlson LE, Goodey E, Angen M. A Randomized, Wait-List Controlled Clinical Trial: The Effect of a Mindfulness Meditation-Based Stress Reduction Program on Mood and Symptoms of Stress in Cancer Outpatients. Psychosomatic Medicine September/October. 2000;62:613-22. | Jadad score (0-5; N/A for trial protocols) | 3 | Study was described as randomised, with appropriate utilisation of random allocation, and included a description of withdrawals/dropouts. |
|  | Allocation sequence (selection bias) | Low risk | Used table of random numbers. |
|  | Allocation concealment (selection bias) | Unclear | Not enough information to make judgement. |
|  | Order of randomisation and consent (selection bias) | Low risk | “When a sufficient number of participants were registered for randomization, all were interviewed individually 1 to 3 weeks before the start of the intervention. Informed consent was obtained at that time, and the baseline psychometric assessment was completed (time 1)... After all registered patients had been interviewed and assessed, they were randomly assigned to either the immediate intervention group (treatment condition) or a wait-list control group (control condition).”  Randomisation followed consent. |
|  | Order of randomisation and baseline measures (selection bias) | Low risk | “When a sufficient number of participants were registered for randomization, all were interviewed individually 1 to 3 weeks before the start of the intervention. Informed consent was obtained at that time, and the baseline psychometric assessment was completed (time 1). Demographic and disease data were also collected and later verified by review of hospital charts. After all registered patients had been interviewed and assessed, they were randomly assigned to either the immediate intervention group (treatment condition) or a wait-list control group (control condition).”  Randomisation followed baseline measures. |
|  | Similarity of baseline outcome measurements across trial arms (selection bias) | Low risk | Little evidence of a difference in baseline outcome measures between trial arms (Tables 3 and 4). |
|  | Similarity of baseline characteristics across trial arms (selection bias) | Low risk | No evidence of a difference in baseline characteristics between trial arms (Table 1). |
|  | Blinding of participants and personnel (performance bias) | High risk | Trial was unblinded. |
|  | Blinded outcome assessment (assessor bias) | Unclear | Not enough information to make judgement. |
|  | Incomplete outcome data (attrition bias) | High risk | Amount of missing data was enough to induce bias in intervention effect estimate. |
|  | Similarity in attrition across trial arms (attrition bias) | High risk | Different proportion of participants were lost over follow-up (13% and 23%). |
| [202] Sripada BN, Henry DB, Jobe TH, Winer JA, Schoeny ME, Gibbons RD. A randomized controlled trial of a feedback method for improving empathic accuracy in psychotherapy. Psychology & Psychotherapy: Theory, Research & Practice. 2011;84:113-27. | Jadad score (0-5; N/A for trial protocols) | 2 | Study was described as randomised, with appropriate utilisation of random allocation. |
|  | Allocation sequence (selection bias) | Low risk | Used coin tossing. |
|  | Allocation concealment (selection bias) | Unclear | Not enough information to make judgement. |
|  | Order of randomisation and consent (selection bias) | Low risk | “IRB‐approved informed consent was obtained from suitable patients by their therapists. Patient–therapist pairs were randomly assigned by the first author to the intervention or control group by flipping a coin.”  Randomisation followed consent. |
|  | Order of randomisation and baseline measures (selection bias) | Unclear | Not enough information to make judgement. |
|  | Similarity of baseline outcome measurements across trial arms (selection bias) | Unclear | Not enough information to make judgement. |
|  | Similarity of baseline characteristics across trial arms (selection bias) | Low risk | Little evidence of a difference in baseline characteristics between trial arms (Table 1). |
|  | Blinding of participants and personnel (performance bias) | High risk | Trial was unblinded. |
|  | Blinded outcome assessment (assessor bias) | N/A | No outcome assessors (participants completed questionnaires). |
|  | Incomplete outcome data (attrition bias) | Unclear | Not enough information to make judgement. |
|  | Similarity in attrition across trial arms (attrition bias) | Unclear | Not enough information to make judgement. |
| [203] Stallard P, Sayal K, Phillips R, Taylor JA, Spears M, Anderson R, et al. Classroom based cognitive behavioural therapy in reducing symptoms of depression in high risk adolescents: pragmatic cluster randomised controlled trial. BMJ October. 2012;6. | Jadad score (0-5; N/A for trial protocols) | 2 | Study was described as randomised, and included a description of withdrawals/dropouts. |
|  | Allocation sequence (selection bias) | Unclear | Not enough information to make judgement. |
|  | Allocation concealment (selection bias) | Unclear | Not enough information to make judgement. |
|  | Order of randomisation and consent (selection bias) | High risk | The trial flow chart (Figure) shows that consent followed randomisation.  Randomisation preceded consent. |
|  | Order of randomisation and baseline measures (selection bias) | High risk | The trial flow chart (Figure) shows that enrolment followed randomisation.  Randomisation preceded baseline measures. |
|  | Similarity of baseline outcome measurements across trial arms (selection bias) | Low risk | No evidence of a difference in baseline outcome measures between trial arms (Table 1). |
|  | Similarity of baseline characteristics across trial arms (selection bias) | Low risk | No evidence of a difference in baseline characteristics between trial arms (Table 1). |
|  | Blinding of participants and personnel (performance bias) | High risk | Trial was unblinded. |
|  | Blinded outcome assessment (assessor bias) | N/A | No outcome assessors (participants completed questionnaires). |
|  | Incomplete outcome data (attrition bias) | High risk | Amount of missing data was enough to induce bias in intervention effect estimate. |
|  | Similarity in attrition across trial arms (attrition bias) | Low risk | Similar proportion of participants were lost over follow-up (19%, 18% and 24%). |
| [204] Stein BD, Jaycox LH, Kataoka SH, Wong M, Tu W, Elliott MN, et al. A Mental Health Intervention for Schoolchildren Exposed to Violence: A Randomized Controlled Trial. JAMA. 2003;290:603-11. | Jadad score (0-5; N/A for trial protocols) | 3 | Study was described as randomised, with appropriate utilisation of random allocation, and included a description of withdrawals/dropouts. |
|  | Allocation sequence (selection bias) | Low risk | Used computer-generated random numbers. |
|  | Allocation concealment (selection bias) | Unclear | Not enough information to make judgement. |
|  | Order of randomisation and consent (selection bias) | Low risk | “After the school mental health clinician obtained parent consent and student assent to participate in the program, a central office was used to randomly assign students to an early intervention group (n = 61) or to a wait-list delayed intervention group (n = 65)…”  Randomisation followed consent. |
|  | Order of randomisation and baseline measures (selection bias) | Unclear | Not enough information to make judgement. |
|  | Similarity of baseline outcome measurements across trial arms (selection bias) | Low risk | No evidence of a difference in baseline outcome measures between trial arms (Table 1). |
|  | Similarity of baseline characteristics across trial arms (selection bias) | Low risk | No evidence of a difference in baseline characteristics between trial arms (Table 1). |
|  | Blinding of participants and personnel (performance bias) | High risk | Trial was unblinded. |
|  | Blinded outcome assessment (assessor bias) | High risk | “…none of the informants (students, parents, or teachers) were blinded to the treatment condition.”  Outcome assessment was not blind. |
|  | Incomplete outcome data (attrition bias) | High risk | Amount of missing data was enough to induce bias in intervention effect estimate. |
|  | Similarity in attrition across trial arms (attrition bias) | Low risk | Similar proportion of participants were lost over follow-up (13% and 8%). |
| [205] Steinglass JE, Albano AM, Simpson BH, Wang YP, Zou J, Attia E, et al. Confronting fear using exposure and response prevention for anorexia nervosa: A randomized controlled pilot study. International Journal Of Eating Disorders. 2014;47:174-80. | Jadad score (0-5; N/A for trial protocols) | 3 | Study was described as randomised, with appropriate utilisation of random allocation, and included a description of withdrawals/dropouts. |
|  | Allocation sequence (selection bias) | Low risk | Used computerised randomisation. |
|  | Allocation concealment (selection bias) | Unclear | Not enough information to make judgement. |
|  | Order of randomisation and consent (selection bias) | Low risk | The trial flow chart (Figure 1) shows that consent preceded randomisation.  Randomisation followed consent. |
|  | Order of randomisation and baseline measures (selection bias) | Low risk | “Patients were informed of their treatment assignment after completion of baseline assessments.”  Randomisation followed baseline measures. |
|  | Similarity of baseline outcome measurements across trial arms (selection bias) | Low risk | No evidence of a difference in baseline outcome measures between trial arms (Table 1). |
|  | Similarity of baseline characteristics across trial arms (selection bias) | Low risk | Little evidence of a difference in baseline characteristics between trial arms (Table 1). |
|  | Blinding of participants and personnel (performance bias) | High risk | Trial was unblinded. |
|  | Blinded outcome assessment (assessor bias) | Unclear | Not enough information to make judgement. |
|  | Incomplete outcome data (attrition bias) | Low risk | Amount of missing data was low and balanced across trial arms. |
|  | Similarity in attrition across trial arms (attrition bias) | Low risk | Similar number of participants were lost over follow-up (1 and 1 participants). |
| [206] Stevens VJ, Glasgow RE, Hollis JF, Mount K. Implementation and Effectiveness of a Brief Smoking-Cessation Intervention for Hospital Patients. Medical Care. 2000;38:451-9. | Jadad score (0-5; N/A for trial protocols) | 1 | Study was described as randomised. |
|  | Allocation sequence (selection bias) | Unclear | Not enough information to make judgement. |
|  | Allocation concealment (selection bias) | Unclear | Not enough information to make judgement. |
|  | Order of randomisation and consent (selection bias) | Unclear | Not enough information to make judgement. |
|  | Order of randomisation and baseline measures (selection bias) | Unclear | Not enough information to make judgement. |
|  | Similarity of baseline outcome measurements across trial arms (selection bias) | N/A | Outcomes not measured at baseline. |
|  | Similarity of baseline characteristics across trial arms (selection bias) | Low risk | No evidence of a difference in baseline characteristics between trial arms (Table 1). |
|  | Blinding of participants and personnel (performance bias) | High risk | Trial was unblinded. |
|  | Blinded outcome assessment (assessor bias) | N/A | No outcome assessors (participants completed questionnaires). |
|  | Incomplete outcome data (attrition bias) | Unclear | Not enough information to make judgement. |
|  | Similarity in attrition across trial arms (attrition bias) | Unclear | Not enough information to make judgement. |
| [207] Stewart-Brown S, Patterson J, Mockford C, Barlow J, Klimes I, Pyper C. Impact of a general practice based group parenting programme: quantitative and qualitative results from a controlled trial at 12 months. Archives of Disease in Childhood. 2004;89:519-25. | Jadad score (0-5; N/A for trial protocols) | 1 | Study was described as randomised. |
|  | Allocation sequence (selection bias) | Unclear | Not enough information to make judgement. |
|  | Allocation concealment (selection bias) | Unclear | Not enough information to make judgement. |
|  | Order of randomisation and consent (selection bias) | Low risk | “Consenting parents were allocated to one of two groups…”  Randomisation followed consent. |
|  | Order of randomisation and baseline measures (selection bias) | Unclear | Not enough information to make judgement. |
|  | Similarity of baseline outcome measurements across trial arms (selection bias) | Low risk | No evidence of a difference in baseline outcome measures between trial arms (Table 1). |
|  | Similarity of baseline characteristics across trial arms (selection bias) | Unclear | Not enough information to make judgement. |
|  | Blinding of participants and personnel (performance bias) | High risk | Trial was unblinded. |
|  | Blinded outcome assessment (assessor bias) | N/A | No outcome assessors (participants completed questionnaires). |
|  | Incomplete outcome data (attrition bias) | High risk | Amount of missing data was enough to induce bias in intervention effect estimate. |
|  | Similarity in attrition across trial arms (attrition bias) | Low risk | Similar proportion of participants were lost over follow-up (25% and 27%). |
| [208] Stuifbergen AK, Blozis SA, Becker H, Phillips L, Timmerman G, Kullberg V, et al. A randomized controlled trial of a wellness intervention for women with fibromyalgia syndrome. Clinical Rehabilitation. 2010;24:305-18. | Jadad score (0-5; N/A for trial protocols) | 3 | Study was described as randomised, with appropriate utilisation of random allocation, and included a description of withdrawals/dropouts. |
|  | Allocation sequence (selection bias) | Low risk | Used coin tossing. |
|  | Allocation concealment (selection bias) | Unclear | Not enough information to make judgement. |
|  | Order of randomisation and consent (selection bias) | Low risk | “The women who were initially screened as ‘eligible’ were told that we would notify them later with exact dates for classes. We mailed two copies of the consent form and a covering letter explaining the project to each woman.” Figure 2 indicates that this phase occurred before randomisation.  Randomisation followed consent. |
|  | Order of randomisation and baseline measures (selection bias) | High risk | “A total of 198 women (85% of those allocated to a treatment group) completed the baseline questionnaire.”  Randomisation preceded baseline measures. |
|  | Similarity of baseline outcome measurements across trial arms (selection bias) | Low risk | No evidence of a difference in baseline outcome measures between trial arms (Table 2). |
|  | Similarity of baseline characteristics across trial arms (selection bias) | Low risk | No evidence of a difference in baseline characteristics between trial arms (Table 1). |
|  | Blinding of participants and personnel (performance bias) | High risk | Trial was unblinded. |
|  | Blinded outcome assessment (assessor bias) | Low risk | “The staff member conducting baseline and final assessments was blinded to the class to which the person was assigned.”  Outcome assessment was blind. |
|  | Incomplete outcome data (attrition bias) | High risk | Amount of missing data was enough to induce bias in intervention effect estimate. In addition, used inappropriate single imputation: “In the case where participants were missing an intermediate value (e.g. completed time 1, 3 and 4, missing time 2) we imputed the missing value as the average of the two nearest time-points.” |
|  | Similarity in attrition across trial arms (attrition bias) | Low risk | Similar proportion of participants were lost over follow-up (32% and 27%). |
| [209] Tate DF, Jackvony EH, Wing RR. Effects of Internet Behavioral Counseling on Weight Loss in Adults at Risk for Type 2 Diabetes: A Randomized Trial. JAMA. 2003;289:1833-6. | Jadad score (0-5; N/A for trial protocols) | 3 | Study was described as randomised, with appropriate utilisation of random allocation, and included a description of withdrawals/dropouts. |
|  | Allocation sequence (selection bias) | Low risk | Used computerised random numbers. |
|  | Allocation concealment (selection bias) | Unclear | Not enough information to make judgement. |
|  | Order of randomisation and consent (selection bias) | Low risk | “The protocol was approved by the institutional review board at Miriam Hospital, RI, and written informed consent was obtained from all participants. Following completion of baseline measurements, participants were randomly assigned…” Figure 1 indicates that consent occurred before randomisation.  Randomisation followed consent. |
|  | Order of randomisation and baseline measures (selection bias) | Low risk | “Following completion of baseline measurements, participants were randomly assigned following simple randomization procedures (computerized random numbers) to 1 of 2 treatment groups.”  Randomisation followed baseline measures. |
|  | Similarity of baseline outcome measurements across trial arms (selection bias) | Low risk | No evidence of a difference in baseline outcome measures between trial arms (Table). |
|  | Similarity of baseline characteristics across trial arms (selection bias) | Low risk | No evidence of a difference in baseline characteristics between trial arms (Table). |
|  | Blinding of participants and personnel (performance bias) | High risk | Trial was unblinded. |
|  | Blinded outcome assessment (assessor bias) | Unclear | Not enough information to make judgement. |
|  | Incomplete outcome data (attrition bias) | High risk | Amount of missing data was enough to induce bias in intervention effect estimate. |
|  | Similarity in attrition across trial arms (attrition bias) | Low risk | Similar proportion of participants were lost over follow-up (15% and 17%). |
| [210] Thompson EA, Eggert LL, Randell BP, Pike KC. Evaluation of Indicated Suicide Risk Prevention Approaches for Potential High School Dropouts. American Journal of Public Health. 2001;91:742-52. | Jadad score (0-5; N/A for trial protocols) | 1 | Study was described as randomised. |
|  | Allocation sequence (selection bias) | Unclear | Not enough information to make judgement. |
|  | Allocation concealment (selection bias) | Unclear | Not enough information to make judgement. |
|  | Order of randomisation and consent (selection bias) | High risk | Cluster (schools) were randomly allocated to interventions. Following this, a two-step process was used to identify participants and then invite them to enrol in the research. Consent followed this.  Randomisation preceded consent. |
|  | Order of randomisation and baseline measures (selection bias) | High risk | Cluster (schools) were randomly allocated to interventions. Following this, a two-step process was used to identify participants and then invite them to enrol in the research. Baseline data collection followed this.  Randomisation preceded baseline measures. |
|  | Similarity of baseline outcome measurements across trial arms (selection bias) | Low risk | “Study groups also were equivalent on all outcome variables except for problem-solving coping (F2,457= 4.30, p=.01).”  Little evidence of a difference in baseline outcome measures between trial arms. |
|  | Similarity of baseline characteristics across trial arms (selection bias) | Low risk | “One-way analysis of variance in the intent-to-treat sample (n = 460) showed no significant differences among groups for the background variables of sex, race/ethnicity, and percentage living with both biological parents. CAST youths, however, tended to be slightly older (F2,457= 3.19, p=.04), but by no more than 5 months on average.”  Little evidence of a difference in baseline characteristics between trial arms. |
|  | Blinding of participants and personnel (performance bias) | High risk | Trial was unblinded. |
|  | Blinded outcome assessment (assessor bias) | Unclear | Not enough information to make judgement. |
|  | Incomplete outcome data (attrition bias) | Low risk | Amount of missing data was low and balanced across trial arms. |
|  | Similarity in attrition across trial arms (attrition bias) | Low risk | “Retention at 9-month follow-up (T_4_) was equivalent across conditions: 86% for C-CARE, 93% for CAST, and 90% for the control (χ^2^(2)= 3.7, P=.15).”  Therefore, similar proportion of participants were lost over follow-up (14%, 7% and 10%). |
| [211] Thorn BE, Day MA, Burns J, Kuhajda MC, Gaskins SW, Sweeney K, et al. Randomized trial of group cognitive behavioral therapy compared with a pain education control for low-literacy rural people with chronic pain. Pain. 2011;152:2710-20. | Jadad score (0-5; N/A for trial protocols) | 3 | Study was described as randomised, with appropriate utilisation of random allocation, and included a description of withdrawals/dropouts. |
|  | Allocation sequence (selection bias) | Low risk | Used coin tossing. |
|  | Allocation concealment (selection bias) | Unclear | “The assignment for each participant number was recorded on a slip of paper and concealed in an envelope.” The authors indicate that the assignments were consecutive but do not say whether envelopes were opaque.  Not enough information to make judgement. |
|  | Order of randomisation and consent (selection bias) | Low risk | “During the informed consent procedure, participants were told that they would be randomly assigned to 1 of 2 treatments…” Also see Figure 1, which indicates that consent occurred before randomisation.  Randomisation followed consent. |
|  | Order of randomisation and baseline measures (selection bias) | Low risk | Participants who “were interested in potentially participating were scheduled for a 90-min, face-to-face pretreatment assessment during which written informed consent was obtained and the structured pain interview, demographics questionnaire, and all outcomes measures were administered. During the informed consent procedure, participants were told that they would be randomly assigned to 1 of 2 treatments…”  Randomisation followed baseline measures. |
|  | Similarity of baseline outcome measurements across trial arms (selection bias) | Low risk | Little evidence of a difference in baseline outcome measures between trial arms (Table 2). |
|  | Similarity of baseline characteristics across trial arms (selection bias) | Low risk | Little evidence of a difference in baseline characteristics between trial arms (Table 1). |
|  | Blinding of participants and personnel (performance bias) | High risk | Trial was unblinded. |
|  | Blinded outcome assessment (assessor bias) | High risk | “All assessments were completed in person, face-to-face by a research assistant not involved in treatment delivery. The interviewer was not blind to subject treatment group assignment.”  Outcome assessment was not blind. |
|  | Incomplete outcome data (attrition bias) | High risk | Amount of missing data was enough to induce bias in intervention effect estimate. |
|  | Similarity in attrition across trial arms (attrition bias) | High risk | Different proportion of participants were lost over follow-up (45% and 21%). |
| [212] Tiwari A, Leung WC, Leung TW, Humphreys J, Parker B, Ho PC. A randomised controlled trial of empowerment training for Chinese abused pregnant women in Hong Kong. BJOG: An International Journal of Obstetrics & Gynaecology. 2005;112:1249-56. | Jadad score (0-5; N/A for trial protocols) | 3 | Study was described as randomised, with appropriate utilisation of random allocation, and included a description of withdrawals/dropouts. |
|  | Allocation sequence (selection bias) | Low risk | Used computer-generated randomisation sequence. |
|  | Allocation concealment (selection bias) | Unclear | The randomisation sequence was “concealed in consecutively numbered, sealed envelopes by a researcher not involved in the study. The sequence was concealed until interventions were assigned.” Does not say that envelopes were opaque.  Not enough information to make judgement. |
|  | Order of randomisation and consent (selection bias) | Low risk | The trial flow chart (Figure 1) shows that consent preceded randomisation.  Randomisation followed consent. |
|  | Order of randomisation and baseline measures (selection bias) | Low risk | “At the time of enrolment, the modified CTS, SF‐36 and demographic questionnaire were administered… At this point, the woman was randomly assigned to either the experimental or control group.”  Randomisation followed baseline measures. |
|  | Similarity of baseline outcome measurements across trial arms (selection bias) | Low risk | No evidence of a difference in baseline outcome measures between trial arms (Table 2). |
|  | Similarity of baseline characteristics across trial arms (selection bias) | Low risk | No evidence of a difference in baseline characteristics between trial arms (Table 1). |
|  | Blinding of participants and personnel (performance bias) | High risk | Trial was unblinded. |
|  | Blinded outcome assessment (assessor bias) | Unclear | Not enough information to make judgement. |
|  | Incomplete outcome data (attrition bias) | Low risk | Amount of missing data was low and balanced across trial arms. |
|  | Similarity in attrition across trial arms (attrition bias) | Low risk | Similar numbers of participants were lost over follow-up (4 and 0 participants). |
| [213] Tol WA, Komproe IH, Susanty D, Jordans M, Macy RD, De Jong JT. School-Based Mental Health Intervention for Children Affected by Political Violence in Indonesia: A Cluster Randomized Trial. JAMA. 2008;300:655-62. | Jadad score (0-5; N/A for trial protocols) | 2 | Study was described as randomised, and included a description of withdrawals/dropouts. |
|  | Allocation sequence (selection bias) | Unclear | Not enough information to make judgement. |
|  | Allocation concealment (selection bias) | Unclear | Not enough information to make judgement. |
|  | Order of randomisation and consent (selection bias) | High risk | The trial flow chart (Figure) shows that enrolment (including consent) followed randomisation.  Randomisation preceded consent. |
|  | Order of randomisation and baseline measures (selection bias) | High risk | The trial flow chart (Figure) shows that baseline measures followed randomisation.  Randomisation preceded baseline measures. |
|  | Similarity of baseline outcome measurements across trial arms (selection bias) | Low risk | Little evidence of a difference in baseline outcome measures between trial arms (Table 1). |
|  | Similarity of baseline characteristics across trial arms (selection bias) | High risk | Evidence of a difference in baseline characteristics between trial arms (Table 1). |
|  | Blinding of participants and personnel (performance bias) | High risk | Trial was unblinded. |
|  | Blinded outcome assessment (assessor bias) | High risk | “Four local assessors with a bachelor’s degree in a social science completed a 5-week training course to competently administer the instruments. It was not possible to blind them to treatment status.”  Outcome assessment was not blind. |
|  | Incomplete outcome data (attrition bias) | High risk | Amount of missing data was enough to induce bias in intervention effect estimate. |
|  | Similarity in attrition across trial arms (attrition bias) | High risk | Different proportion of participants were lost over follow-up (3% and 14% of children; 8% and 20%). |
| [214] Tolan P, Gorman-Smith D, Henry D. Supporting Families in a High-Risk Setting: Proximal Effects of the SAFEChildren Preventive Intervention. Journal of Consulting & Clinical Psychology. 2004;72:855-69. | Jadad score (0-5; N/A for trial protocols) | 1 | Study was described as randomised. |
|  | Allocation sequence (selection bias) | Unclear | Not enough information to make judgement. |
|  | Allocation concealment (selection bias) | Unclear | Not enough information to make judgement. |
|  | Order of randomisation and consent (selection bias) | Low risk | “…55% of the children with consent in each classroom were randomly assigned to the intervention condition, and 45% were assigned to the control condition.”  Randomisation followed consent. |
|  | Order of randomisation and baseline measures (selection bias) | Low risk | “After completing a baseline assessment during the spring of their children’s kindergarten year, families were randomly assigned to the intervention group or to a non-intervention control group.”  Randomisation followed baseline measures. |
|  | Similarity of baseline outcome measurements across trial arms (selection bias) | Low risk | No evidence of a difference in baseline outcome measures between trial arms (Table 2). |
|  | Similarity of baseline characteristics across trial arms (selection bias) | Unclear | Not enough information to make judgement. |
|  | Blinding of participants and personnel (performance bias) | High risk | Trial was unblinded. |
|  | Blinded outcome assessment (assessor bias) | Unclear | Not enough information to make judgement. |
|  | Incomplete outcome data (attrition bias) | Unclear | Not enough information to make judgement. |
|  | Similarity in attrition across trial arms (attrition bias) | Unclear | Not enough information to make judgement. |
| [215] Tross S, Campbell A, Cohen LR, Calsyn D, Pavlicova M, Miele GM, et al. Effectiveness of HIV/STD Sexual Risk Reduction Groups for Women in Substance Abuse Treatment Programs: Results of NIDA Clinical Trials Network Trial. JAIDS Journal of Acquired Immune Deficiency Syndromes. 2008;48:581-9. | Jadad score (0-5; N/A for trial protocols) | 1 | Study was described as randomised. |
|  | Allocation sequence (selection bias) | Unclear | Not enough information to make judgement. |
|  | Allocation concealment (selection bias) | Low risk | Used central allocation (via telephone).  Allocation was concealed. |
|  | Order of randomisation and consent (selection bias) | Low risk | The trial flow chart (Figure 1) shows that consent occurred before randomisation.  Randomisation followed consent. |
|  | Order of randomisation and baseline measures (selection bias) | Low risk | Not enough information to make judgement. |
|  | Similarity of baseline outcome measurements across trial arms (selection bias) | Low risk | No evidence of a difference in baseline outcome measures between trial arms (Table 3). |
|  | Similarity of baseline characteristics across trial arms (selection bias) | Low risk | No evidence of a difference in baseline characteristics between trial arms (Table 3). |
|  | Blinding of participants and personnel (performance bias) | High risk | Trial was unblinded. |
|  | Blinded outcome assessment (assessor bias) | Low risk | “Procedures used to protect the blinding of the research assistants conducting the assessments included: (1) instructing intervention counselors, site Project Coordinators, and study participants not to disclose randomization to research staff; and (2) using highly structured assessments and computer assisted self-interviews to minimize spontaneous discussion between research assistants and participants.”  Outcome assessment was blind. |
|  | Incomplete outcome data (attrition bias) | High risk | Amount of missing data was enough to induce bias in intervention effect estimate. |
|  | Similarity in attrition across trial arms (attrition bias) | Low risk | Similar proportion of participants were lost over follow-up (38% and 33%). |
| [216] Tucker T, Fry CL, Lintzeris N, Baldwin S, Ritter A, Donath S, et al. Randomized controlled trial of a brief behavioural intervention for reducing hepatitis C virus risk practices among injecting drug users. Addiction. 2004;99:1157-66. | Jadad score (0-5; N/A for trial protocols) | 1 | Study was described as randomised. |
|  | Allocation sequence (selection bias) | Unclear | Not enough information to make judgement. |
|  | Allocation concealment (selection bias) | Unclear | “…each randomization outcome was enclosed in a sealed envelope, to conceal intervention allocations.” Not clear if envelopes were sequentially numbered.  Not enough information to make judgement. |
|  | Order of randomisation and consent (selection bias) | Low risk | “The clinical researcher opened the next available randomization envelope after written informed consent and baseline data collection.”  Randomisation followed consent. |
|  | Order of randomisation and baseline measures (selection bias) | Low risk | “The clinical researcher opened the next available randomization envelope after written informed consent and baseline data collection.”  Randomisation followed baseline measures. |
|  | Similarity of baseline outcome measurements across trial arms (selection bias) | High risk | Evidence of a difference in baseline outcome measures between trial arms (Table 1). |
|  | Similarity of baseline characteristics across trial arms (selection bias) | Unclear | Not enough information to make judgement. |
|  | Blinding of participants and personnel (performance bias) | High risk | Trial was unblinded. |
|  | Blinded outcome assessment (assessor bias) | Low risk | “All outcome measurement (BBV-TRAQ and client satisfaction data) at follow-up was conducted by a separate research assistant (SB) blind to the intervention each participant had received.”  Outcome assessment was blind. |
|  | Incomplete outcome data (attrition bias) | High risk | Amount of missing data was enough to induce bias in intervention effect estimate. |
|  | Similarity in attrition across trial arms (attrition bias) | High risk | Different proportion of participants were lost over follow-up (12% and 18%). |
| [217] Unutzer J, Katon W, Callahan CM, Williams JWJ, Hunkeler E, Harpole L, et al. Collaborative Care Management of Late-Life Depression in the Primary Care Setting: A Randomized Controlled Trial. JAMA. 2002;288:2836-45. | Jadad score (0-5; N/A for trial protocols) | 3 | Study was described as randomised, with appropriate utilisation of random allocation, and included a description of withdrawals/dropouts. |
|  | Allocation sequence (selection bias) | Low risk | Used computer-generated random numbers. |
|  | Allocation concealment (selection bias) | Unclear | “Random assignment information was contained in a set of numbered, sealed envelopes…” Not clear if envelopes were opaque.  Not enough information to make judgement. |
|  | Order of randomisation and consent (selection bias) | Low risk | The trial flow chart (Figure 1) shows that consent occurred before randomisation.  Randomisation followed consent. |
|  | Order of randomisation and baseline measures (selection bias) | Low risk | “Baseline interviews were conducted by trained lay interviewers using structured computerized interviews[^36^](https://jamanetwork.com/journals/jama/fullarticle/195599?version=meter%20at%20null&module=meter-Links&pgtype=Blogs&contentId=&mediaId=&referrer=&priority=true&action=click&contentCollection=meter-links-click#REF-JOC21093-36) before randomization.”  Randomisation followed baseline measures. |
|  | Similarity of baseline outcome measurements across trial arms (selection bias) | Low risk | No evidence of a difference in baseline outcome measures between trial arms (Table 3). |
|  | Similarity of baseline characteristics across trial arms (selection bias) | Low risk | No evidence of a difference in baseline characteristics between trial arms (Table 2). |
|  | Blinding of participants and personnel (performance bias) | High risk | Trial was unblinded. |
|  | Blinded outcome assessment (assessor bias) | Low risk | “Blind follow-up interviews were performed at 3, 6, and 12 months by trained interviewers at a telephone survey research group using computer-assisted telephone interviews.”  Outcome assessment was blind. |
|  | Incomplete outcome data (attrition bias) | High risk | Amount of missing data was enough to induce bias in intervention effect estimate. |
|  | Similarity in attrition across trial arms (attrition bias) | Low risk | Similar proportion of participants were lost over follow-up (16% and 14%). |
| [218] Verbiest MEA, Crone MR, Scharloo M, Chavannes NH, van der Meer V, Kaptein AA, et al. One-Hour Training for General Practitioners in Reducing the Implementation Gap of Smoking Cessation Care: A Cluster-Randomized Controlled Trial. Nicotine & Tobacco Research. 2014;16:1-10. | Jadad score (0-5; N/A for trial protocols) | 3 | Study was described as randomised, with appropriate utilisation of random allocation, and included a description of withdrawals/dropouts. |
|  | Allocation sequence (selection bias) | Low risk | Used coin tossing. |
|  | Allocation concealment (selection bias) | Unclear | Not enough information to make judgement. |
|  | Order of randomisation and consent (selection bias) | Unclear | Not enough information to make judgement. |
|  | Order of randomisation and baseline measures (selection bias) | Unclear | Not enough information to make judgement. |
|  | Similarity of baseline outcome measurements across trial arms (selection bias) | Low risk | No evidence of a difference in baseline outcome measures between trial arms (Table 2). |
|  | Similarity of baseline characteristics across trial arms (selection bias) | Low risk | No evidence of a difference in baseline characteristics between trial arms (Table 3). |
|  | Blinding of participants and personnel (performance bias) | High risk | Trial was unblinded for GPs (but not for patients) |
|  | Blinded outcome assessment (assessor bias) | N/A | No outcome assessors (participants completed questionnaires). |
|  | Incomplete outcome data (attrition bias) | High risk | Amount of missing data was enough to induce bias in intervention effect estimate. |
|  | Similarity in attrition across trial arms (attrition bias) | High risk | Different proportion of participants were lost over follow-up (62% and 70%). |
| [219] Vidal R, Bosch R, Nogueira M, Gomez-Barros N, Valero S, Palomar G, et al. Psychoeducation for Adults With Attention Deficit Hyperactivity Disorder vs. Cognitive Behavioral Group Therapy: A Randomized Controlled Pilot Study. Journal of Nervous & Mental Disease. 2013;201:894-900. | Jadad score (0-5; N/A for trial protocols) | 3 | Study was described as randomised, with appropriate utilisation of random allocation, and included a description of withdrawals/dropouts. |
|  | Allocation sequence (selection bias) | Low risk | Used computerised randomisation. |
|  | Allocation concealment (selection bias) | Unclear | Not enough information to make judgement. |
|  | Order of randomisation and consent (selection bias) | Low risk | The trial flow chart (Figure 1) shows that consent occurred before randomisation.  Randomisation followed consent. |
|  | Order of randomisation and baseline measures (selection bias) | Unclear | Not enough information to make judgement. |
|  | Similarity of baseline outcome measurements across trial arms (selection bias) | Low risk | No evidence of a difference in baseline outcome measures between trial arms (Table 3). |
|  | Similarity of baseline characteristics across trial arms (selection bias) | Low risk | No evidence of a difference in baseline characteristics between trial arms (Table 2). |
|  | Blinding of participants and personnel (performance bias) | High risk | Trial was unblinded. |
|  | Blinded outcome assessment (assessor bias) | Low risk | “The pretreatment and posttreatment evaluations were conducted by a psychologist blinded to this study.”  Outcome assessment was blind. |
|  | Incomplete outcome data (attrition bias) | High risk | Used inappropriate simple imputation (used last observation carried forward). |
|  | Similarity in attrition across trial arms (attrition bias) | Low risk | Similar number of participants were lost over follow-up (1 and 3 participants). |
| [220] Vlasveld MC, van der Feltz-Cornelis CM, Ader HJ, Anema JR, Hoedeman R, van Mechelen W, et al. Collaborative care for sick-listed workers with major depressive disorder: a randomised controlled trial from the Netherlands Depression Initiative aimed at return to work and depressive symptoms. Occupational & Environmental Medicine. 2013;70:223-30. | Jadad score (0-5; N/A for trial protocols) | 3 | Study was described as randomised, with appropriate utilisation of random allocation, and included a description of withdrawals/dropouts. |
|  | Allocation sequence (selection bias) | Low risk | Used computerised randomisation. |
|  | Allocation concealment (selection bias) | Unclear | Not enough information to make judgement. |
|  | Order of randomisation and consent (selection bias) | Low risk | The trial flow chart (Figure 1) shows that consent occurred before randomisation.  Randomisation followed consent. |
|  | Order of randomisation and baseline measures (selection bias) | High risk | “Then, the participant was informed about the computer generated allocation status by the research assistant. Next, the baseline questionnaire was sent by mail.”  Randomisation preceded baseline measures. |
|  | Similarity of baseline outcome measurements across trial arms (selection bias) | Low risk | No evidence of a difference in baseline outcome measures between trial arms (Table 2). |
|  | Similarity of baseline characteristics across trial arms (selection bias) | Low risk | No evidence of a difference in baseline characteristics between trial arms (Table 1). |
|  | Blinding of participants and personnel (performance bias) | High risk | Trial was unblinded. |
|  | Blinded outcome assessment (assessor bias) | N/A | No outcome assessors (participants completed questionnaires). |
|  | Incomplete outcome data (attrition bias) | High risk | Amount of missing data was enough to induce bias in intervention effect estimate. |
|  | Similarity in attrition across trial arms (attrition bias) | High risk | Different proportion of participants were lost over follow-up (43% and 51% of questionnaire data; 6% and 13% of registry data). |
| [221] Waghorn G, Dias S, Gladman B, Harris M, Saha S. A multi-site randomised controlled trial of evidence-based supported employment for adults with severe and persistent mental illness. Australian Occupational Therapy Journal. 2014;61:424-36. | Jadad score (0-5; N/A for trial protocols) | 3 | Study was described as randomised, with appropriate utilisation of random allocation, and included a description of withdrawals/dropouts. |
|  | Allocation sequence (selection bias) | Low risk | Used computerised randomisation. |
|  | Allocation concealment (selection bias) | Unclear | Not enough information to make judgement. |
|  | Order of randomisation and consent (selection bias) | Low risk | “At the first meeting with interested volunteers, the research officer appointed to each site provided a full description of the study prior to seeking written informed consent… Each participant completed the baseline interview protocol before being randomly allocated…”  Randomisation followed consent. |
|  | Order of randomisation and baseline measures (selection bias) | Low risk | “Each participant completed the baseline interview protocol before being randomly allocated…”  Randomisation followed baseline measures. |
|  | Similarity of baseline outcome measurements across trial arms (selection bias) | N/A | Outcomes not measured at baseline. |
|  | Similarity of baseline characteristics across trial arms (selection bias) | Low risk | No evidence of a difference in baseline characteristics between trial arms (Table 1). |
|  | Blinding of participants and personnel (performance bias) | High risk | Trial was unblinded. |
|  | Blinded outcome assessment (assessor bias) | Unclear | Not enough information to make judgement. |
|  | Incomplete outcome data (attrition bias) | High risk | Amount of missing data was enough to induce bias in intervention effect estimate. |
|  | Similarity in attrition across trial arms (attrition bias) | High risk | Different proportion of participants were lost over follow-up (37% and 52%). |
| [222] Wagner GJ, Kanouse DE, Golinelli D, Miller LG, Daar ES, Witt MD, et al. Cognitive-behavioral intervention to enhance adherence to antiretroviral therapy: a randomized controlled trial (CCTG 578). AIDS. 2006;20:1295-302. | Jadad score (0-5; N/A for trial protocols) | 2 | Study was described as randomised, and included a description of withdrawals/dropouts. |
|  | Allocation sequence (selection bias) | Unclear | Not enough information to make judgement. |
|  | Allocation concealment (selection bias) | Unclear | Not enough information to make judgement. |
|  | Order of randomisation and consent (selection bias) | Unclear | Not enough information to make judgement. |
|  | Order of randomisation and baseline measures (selection bias) | Unclear | Not enough information to make judgement. |
|  | Similarity of baseline outcome measurements across trial arms (selection bias) | Unclear | Not enough information to make judgement. |
|  | Similarity of baseline characteristics across trial arms (selection bias) | Unclear | Not enough information to make judgement. |
|  | Blinding of participants and personnel (performance bias) | High risk | Trial was unblinded. |
|  | Blinded outcome assessment (assessor bias) | Unclear | Not enough information to make judgement. |
|  | Incomplete outcome data (attrition bias) | High risk | Amount of missing data was enough to induce bias in intervention effect estimate. |
|  | Similarity in attrition across trial arms (attrition bias) | Low risk | Similar proportion of participants were lost over follow-up (61%, 63% and 68%). |
| [223] Walpole B, Dettmer E, Morrongiello BA, McCrindle BW, Hamilton J. Motivational Interviewing to Enhance Self-Efficacy and Promote Weight Loss in Overweight and Obese Adolescents: A Randomized Controlled Trial. Journal of Pediatric Psychology. 2013;38:944-53. | Jadad score (0-5; N/A for trial protocols) | 3 | Study was described as randomised, with appropriate utilisation of random allocation, and included a description of withdrawals/dropouts. |
|  | Allocation sequence (selection bias) | Low risk | Used computerised randomisation. |
|  | Allocation concealment (selection bias) | Unclear | Not enough information to make judgement. |
|  | Order of randomisation and consent (selection bias) | Low risk | “Between September 2010 and Janurary 2012, ∼73 children and adolescents were enrolled in the pediatric outpatient weight program. Fifty-two of eligible participants remained, and 40 of them were consented and randomized to a study intervention group.”  Randomisation followed consent. |
|  | Order of randomisation and baseline measures (selection bias) | Unclear | Not enough information to make judgement. |
|  | Similarity of baseline outcome measurements across trial arms (selection bias) | Low risk | No evidence of a difference in baseline outcome measures between trial arms (Table 3). |
|  | Similarity of baseline characteristics across trial arms (selection bias) | Low risk | No evidence of a difference in baseline characteristics between trial arms (Table 2). |
|  | Blinding of participants and personnel (performance bias) | High risk | Trial was unblinded. |
|  | Blinded outcome assessment (assessor bias) | Unclear | Not enough information to make judgement. |
|  | Incomplete outcome data (attrition bias) | Low risk | Amount of missing data was low and balanced across trial arms. |
|  | Similarity in attrition across trial arms (attrition bias) | Low risk | Similar number of participants were lost over follow-up (2 and 0 participants). |
| [224] Webb MS, de Ybarra DR, Baker EA, Reis IM, Carey MP. Cognitive-Behavioral Therapy to Promote Smoking Cessation Among African American Smokers: A Randomized Clinical Trial. Journal of Consulting & Clinical Psychology. 2010;78:24-33. | Jadad score (0-5; N/A for trial protocols) | 1 | Study was described as randomised. |
|  | Allocation sequence (selection bias) | Unclear | Not enough information to make judgement. |
|  | Allocation concealment (selection bias) | Unclear | Not enough information to make judgement. |
|  | Order of randomisation and consent (selection bias) | High risk | “Eligible participants were assigned a tentative random assignment and scheduled for an orientation session, and only those who attended and provided informed consent were officially enrolled in the group.”  Randomisation preceded consent. |
|  | Order of randomisation and baseline measures (selection bias) | Unclear | Not enough information to make judgement. |
|  | Similarity of baseline outcome measurements across trial arms (selection bias) | N/A | Outcomes not measured at baseline. |
|  | Similarity of baseline characteristics across trial arms (selection bias) | Low risk | No evidence of a difference in baseline characteristics between trial arms (Table 1). |
|  | Blinding of participants and personnel (performance bias) | High risk | Trial was unblinded. |
|  | Blinded outcome assessment (assessor bias) | Low risk | “The 6-month assessment occurred via telephone by the RA, who was blinded to study condition.”  Outcome assessment was blind. |
|  | Incomplete outcome data (attrition bias) | High risk | Amount of missing data was enough to induce bias in intervention effect estimate. |
|  | Similarity in attrition across trial arms (attrition bias) | Low risk | Similar proportion of participants were lost over follow-up (29% and 31%). |
| [225] Wells DL, Dawson P, Sidani S, Craig D, Pringle D. Effects of an Abilities-Focused Program of Morning Care on Residents Who Have Dementia and On Caregivers. Journal of the American Geriatrics Society. 2000;48:442-9. | Jadad score (0-5; N/A for trial protocols) | 1 | Study was described as randomised. |
|  | Allocation sequence (selection bias) | Unclear | Not enough information to make judgement. |
|  | Allocation concealment (selection bias) | Unclear | Not enough information to make judgement. |
|  | Order of randomisation and consent (selection bias) | Unclear | Not enough information to make judgement. |
|  | Order of randomisation and baseline measures (selection bias) | Unclear | Not enough information to make judgement. |
|  | Similarity of baseline outcome measurements across trial arms (selection bias) | Low risk | No evidence of a difference in baseline outcome measures between trial arms (Tables 3 and 4). |
|  | Similarity of baseline characteristics across trial arms (selection bias) | Low risk | No evidence of a difference in baseline characteristics between trial arms (Tables 1 and 2). |
|  | Blinding of participants and personnel (performance bias) | High risk | Trial was unblinded. |
|  | Blinded outcome assessment (assessor bias) | Low risk | “A second research assistant, who was blind to the study design and hypotheses, was trained to collect data during observations of morning care using the MIBM, PAS, and IBM.”  Outcome assessment was blind. |
|  | Incomplete outcome data (attrition bias) | Unclear | Not enough information to make judgement. |
|  | Similarity in attrition across trial arms (attrition bias) | Unclear | Not enough information to make judgement. |
| [226] Wells KB, Sherbourne C, Schoenbaum M, Duan N, Meredith L, Unutzer J, et al. Impact of disseminating quality improvement programs for depression in managed primary care - A randomized controlled trial. Jama-J Am Med Assoc. 2000;283:212-20. | Jadad score (0-5; N/A for trial protocols) | 3 | Study was described as randomised, with appropriate utilisation of random allocation, and included a description of withdrawals/dropouts. |
|  | Allocation sequence (selection bias) | Low risk | Used random number table. |
|  | Allocation concealment (selection bias) | Unclear | Not enough information to make judgement. |
|  | Order of randomisation and consent (selection bias) | Low risk | “We asked all primary care clinicians to enrol prior to their knowledge of intervention status; 97% (n = 181) agreed, including internists and family practice physicians (87%) and nurse practitioners (13%).” (Patient consent preceded enrolment.) “Patients learned of their intervention status after enrolment.”  Randomisation followed consent. |
|  | Order of randomisation and baseline measures (selection bias) | Unclear | Not enough information to make judgement. |
|  | Similarity of baseline outcome measurements across trial arms (selection bias) | Low risk | No evidence of a difference in baseline outcome measures between trial arms (Tables 2, 3, 4 and 5). |
|  | Similarity of baseline characteristics across trial arms (selection bias) | Low risk | Little evidence of a difference in baseline characteristics between trial arms (Table 2). |
|  | Blinding of participants and personnel (performance bias) | High risk | Trial was unblinded. |
|  | Blinded outcome assessment (assessor bias) | Unclear | Not enough information to make judgement. |
|  | Incomplete outcome data (attrition bias) | High risk | Amount of missing data was enough to induce bias in intervention effect estimate. |
|  | Similarity in attrition across trial arms (attrition bias) | Low risk | Similar proportion of participants were lost over follow-up (16% and 18%). |
| [227] Werch CE, Bian H, Carlson JM, Moore MJ, DiClemente CC, Huang IC, et al. Brief integrative multiple behavior intervention effects and mediators for adolescents. Journal of Behavioral Medicine. 2011;34:3-12. | Jadad score (0-5; N/A for trial protocols) | 2 | Study was described as randomised, with appropriate utilisation of random allocation. |
|  | Allocation sequence (selection bias) | Low risk | Used computer-generated random numbers. |
|  | Allocation concealment (selection bias) | Unclear | Not enough information to make judgement. |
|  | Order of randomisation and consent (selection bias) | Unclear | Not enough information to make judgement. |
|  | Order of randomisation and baseline measures (selection bias) | Unclear | Not enough information to make judgement. |
|  | Similarity of baseline outcome measurements across trial arms (selection bias) | Low risk | No evidence of a difference in baseline outcome measures between trial arms (Tables 1 and 2). |
|  | Similarity of baseline characteristics across trial arms (selection bias) | Low risk | Little evidence of a difference in baseline characteristics between trial arms (Table 1). |
|  | Blinding of participants and personnel (performance bias) | High risk | Trial was unblinded. |
|  | Blinded outcome assessment (assessor bias) | Unclear | Not enough information to make judgement. |
|  | Incomplete outcome data (attrition bias) | Low risk | Amount of missing data was low (6%) and balanced across trial arms. |
|  | Similarity in attrition across trial arms (attrition bias) | Low risk | “No differences were found in the proportion of those who dropped out between treatment groups…”  Similar proportion of participants were lost over follow-up. |
| [228] Wild B, Friederich HC, Gross G, Teufel M, Herzog W, Giel KE, et al. The ANTOP study: focal psychodynamic psychotherapy, cognitive-behavioural therapy, and treatment-as-usual in outpatients with anorexia nervosa--a randomized controlled trial. Trials [Electronic Resource]. 2009;10:23. | Jadad score (0-5; N/A for trial protocols) | N/A | Clinical trial protocol |
|  | Allocation sequence (selection bias) | Unclear | Not enough information to make judgement. |
|  | Allocation concealment (selection bias) | Low risk | Used central allocation.  Allocation was concealed. |
|  | Order of randomisation and consent (selection bias) | Low risk | Obtaining consent was an inclusion criterion.  Randomisation followed consent. |
|  | Order of randomisation and baseline measures (selection bias) | Low risk | Baseline measures of outcome (BMI) are described as “pre-randomisation”.  Randomisation followed baseline measures. |
|  | Similarity of baseline outcome measurements across trial arms (selection bias) | N/A | Clinical trial protocol |
|  | Similarity of baseline characteristics across trial arms (selection bias) | N/A | Clinical trial protocol |
|  | Blinding of participants and personnel (performance bias) | High risk | Trial was unblinded. |
|  | Blinded outcome assessment (assessor bias) | Low risk | “Outcome assessments are either self-reported or performed by an independent blinded observer.”  Outcome assessment was blind. |
|  | Incomplete outcome data (attrition bias) | High risk | Using inappropriate single imputation (last observation carried forward). |
|  | Similarity in attrition across trial arms (attrition bias) | N/A | Clinical trial protocol |
| [229] Williams JW, Katon W, Lin EH, Noel PH, Worchel J, Cornell J, et al. The Effectiveness of Depression Care Management on Diabetes-Related Outcomes in Older Patients. Annals of Internal Medicine. 2004;140:1015-24. | Jadad score (0-5; N/A for trial protocols) | 2 | Study was described as randomised, and included a description of withdrawals/dropouts. |
|  | Allocation sequence (selection bias) | Unclear | Not enough information to make judgement. |
|  | Allocation concealment (selection bias) | Unclear | “Randomization information was contained in a set of numbered, sealed envelopes.” Not clear if envelopes were opaque.  Not enough information to make judgement. |
|  | Order of randomisation and consent (selection bias) | Low risk | The trial flow chart (Figure 1) shows that consent occurred before randomisation.  Randomisation followed consent. |
|  | Order of randomisation and baseline measures (selection bias) | Low risk | “After the baseline interview, we randomly assigned participants to the IMPACT intervention or usual care.”  Randomisation followed baseline measures. |
|  | Similarity of baseline outcome measurements across trial arms (selection bias) | Low risk | No evidence of a difference in baseline outcome measures between trial arms (Tables 2 and 3). |
|  | Similarity of baseline characteristics across trial arms (selection bias) | Low risk | No evidence of a difference in baseline characteristics between trial arms (Table 1). |
|  | Blinding of participants and personnel (performance bias) | High risk | Trial was unblinded. |
|  | Blinded outcome assessment (assessor bias) | Low risk | “Trained interviewers who were blinded to treatment assignment performed computer-assisted telephone interviews at 3, 6, and 12 months.”  Outcome assessment was blind. |
|  | Incomplete outcome data (attrition bias) | Low risk | Amount of missing data was low and balanced across trial arms. |
|  | Similarity in attrition across trial arms (attrition bias) | Low risk | Similar proportion of participants were lost over follow-up (6% and 6%). |
| [230] Wingood GM, DiClemente RJ, Robinson-Simpson L, Lang DL, Caliendo A, Hardin JW. Efficacy of an HIV Intervention in Reducing High-Risk Human Papillomavirus, Nonviral Sexually Transmitted Infections, and Concurrency Among African American Women: A Randomized-Controlled Trial. JAIDS Journal of Acquired Immune Deficiency Syndromes. 2013;63 Supplement:S36-S43. | Jadad score (0-5; N/A for trial protocols) | 3 | Study was described as randomised, with appropriate utilisation of random allocation, and included a description of withdrawals/dropouts. |
|  | Allocation sequence (selection bias) | Low risk | Used computerised randomisation. |
|  | Allocation concealment (selection bias) | Unclear | “As participants completed baseline assessments, sealed opaque envelopes were used to execute assignments.” Not clear if envelopes were sequentially numbered.  Not enough information to make judgement. |
|  | Order of randomisation and consent (selection bias) | Unclear | Not enough information to make judgement. |
|  | Order of randomisation and baseline measures (selection bias) | Low risk | “As participants completed baseline assessments, sealed opaque envelopes were used to execute assignments.”  Randomisation followed baseline measures. |
|  | Similarity of baseline outcome measurements across trial arms (selection bias) | Low risk | No evidence of a difference in baseline outcome measures between trial arms (Tables 1 and 4). |
|  | Similarity of baseline characteristics across trial arms (selection bias) | Low risk | No evidence of a difference in baseline characteristics between trial arms (Table 1). |
|  | Blinding of participants and personnel (performance bias) | High risk | Trial was unblinded. |
|  | Blinded outcome assessment (assessor bias) | N/A | No outcome assessors (participants completed questionnaires). |
|  | Incomplete outcome data (attrition bias) | High risk | Amount of missing data was enough to induce bias in intervention effect estimate. |
|  | Similarity in attrition across trial arms (attrition bias) | Low risk | Similar proportion of participants were lost over follow-up (25% and 25%). |
| [231] Wood JJ, Piacentini JC, Southam-Gerow M, Chu BC, Sigman M. Family Cognitive Behavioral Therapy for Child Anxiety Disorders. Journal of the American Academy of Child & Adolescent Psychiatry. 2006;45:314-21. | Jadad score (0-5; N/A for trial protocols) | 2 | Study was described as randomised, with appropriate utilisation of random allocation. |
|  | Allocation sequence (selection bias) | Low risk | Used computerised randomisation. |
|  | Allocation concealment (selection bias) | Low risk | “Children who met inclusion/exclusion criteria were randomized by a research assistant to either the CCBT or FCBT condition using a computer randomization program (the randomization sequence was concealed from investigators until interventions were assigned).”  Allocation was concealed. |
|  | Order of randomisation and consent (selection bias) | Low risk | “On the day of the assessment, consent forms were reviewed and signed, and the diagnostic interview and self-report measures were conducted. Children… were randomized…”  Randomisation followed consent. |
|  | Order of randomisation and baseline measures (selection bias) | Low risk | “On the day of the assessment, consent forms were reviewed and signed, and the diagnostic interview and self-report measures were conducted. Children… were randomized…”  Randomisation followed baseline measures. |
|  | Similarity of baseline outcome measurements across trial arms (selection bias) | Low risk | No evidence of a difference in baseline outcome measures between trial arms (Table 2). |
|  | Similarity of baseline characteristics across trial arms (selection bias) | Low risk | No evidence of a difference in baseline characteristics between trial arms (Table 1). |
|  | Blinding of participants and personnel (performance bias) | High risk | Trial was unblinded. |
|  | Blinded outcome assessment (assessor bias) | Low risk | “Trained independent evaluators blind to treatment condition conducted all interviews.”  Outcome assessment was blind. |
|  | Incomplete outcome data (attrition bias) | Unclear | Not enough information to make judgement. |
|  | Similarity in attrition across trial arms (attrition bias) | Unclear | Not enough information to make judgement. |
| [232] Wu L-M, Chiou S-S, Sheen J-M, Lin P-C, Liao YM, Chen H-M, et al. Evaluating the acceptability and efficacy of a psycho-educational intervention for coping and symptom management by children with cancer: a randomized controlled study. Journal of Advanced Nursing. 2014;70:1653-62. | Jadad score (0-5; N/A for trial protocols) | 2 | Study was described as randomised, and included a description of withdrawals/dropouts. |
|  | Allocation sequence (selection bias) | Unclear | Not enough information to make judgement. |
|  | Allocation concealment (selection bias) | Unclear | Not enough information to make judgement. |
|  | Order of randomisation and consent (selection bias) | Low risk | The trial flow chart (Figure 1) shows that consent occurred before randomisation.  Randomisation followed consent. |
|  | Order of randomisation and baseline measures (selection bias) | High risk | The trial flow chart (Figure 1) shows that baseline measures occurred after randomisation.  Randomisation preceded consent. |
|  | Similarity of baseline outcome measurements across trial arms (selection bias) | Low risk | No evidence of a difference in baseline outcome measures between trial arms (Tables 3 and 4). |
|  | Similarity of baseline characteristics across trial arms (selection bias) | Low risk | No evidence of a difference in baseline characteristics between trial arms (Table 1). |
|  | Blinding of participants and personnel (performance bias) | High risk | Trial was unblinded. |
|  | Blinded outcome assessment (assessor bias) | Unclear | Not enough information to make judgement. |
|  | Incomplete outcome data (attrition bias) | High risk | Amount of missing data was low and balanced across trial arms. |
|  | Similarity in attrition across trial arms (attrition bias) | Low risk | Similar proportion of participants were lost over follow-up (3% and 6%). |
| [233] Yoder P, Stone WL. Randomized Comparison of Two Communication Interventions for Preschoolers With Autism Spectrum Disorders. Journal of Consulting & Clinical Psychology. 2006;74:426-35. | Jadad score (0-5; N/A for trial protocols) | 3 | Study was described as randomised, with appropriate utilisation of random allocation, and included a description of withdrawals/dropouts. |
|  | Allocation sequence (selection bias) | Low risk | Used a computer random number generator. |
|  | Allocation concealment (selection bias) | Unclear | Not enough information to make judgement. |
|  | Order of randomisation and consent (selection bias) | Low risk | Randomisation “was performed after participants signed consent forms”. |
|  | Order of randomisation and baseline measures (selection bias) | Low risk | Randomisation “was performed after participants signed consent forms, qualified for the study, and completed Time 1 assessments.” |
|  | Similarity of baseline outcome measurements across trial arms (selection bias) | Unclear | Not enough information to make judgement. |
|  | Similarity of baseline characteristics across trial arms (selection bias) | Low risk | No evidence of a difference in baseline characteristics between trial arms (Table 1). |
|  | Blinding of participants and personnel (performance bias) | High risk | Trial was unblinded |
|  | Blinded outcome assessment (assessor bias) | High risk | “One of the weaknesses of the study is that the examiners conducting the pre- and postassessments were also the primary data coders and could not be kept blind to the children’s treatment assignment.”  Outcome assessment was not blind. |
|  | Incomplete outcome data (attrition bias) | Low risk | No missing data. |
|  | Similarity in attrition across trial arms (attrition bias) | Low risk | No participants were lost to follow-up in either trial arm. |
| [234] Zhou K, Li X, Yan H, Dang S, Wang D-l. Effects of music therapy on depression and duration of hospital stay of breast cancer patients after radical mastectomy. Chinese Medical Journal (English Edition). 2011;124:2321-7. | Jadad score (0-5; N/A for trial protocols) | 3 | Study was described as randomised, with appropriate utilisation of random allocation, and included a description of withdrawals/dropouts. |
|  | Allocation sequence (selection bias) | Low risk | Used computer-generated random numbers. |
|  | Allocation concealment (selection bias) | Unclear | Not enough information to make judgement. |
|  | Order of randomisation and consent (selection bias) | High risk | “Written consent form was signed up only by those who would receive music therapy…”  Randomisation preceded consent. |
|  | Order of randomisation and baseline measures (selection bias) | Unclear | Not enough information to make judgement. |
|  | Similarity of baseline outcome measurements across trial arms (selection bias) | Low risk | No evidence of a difference in baseline outcome measures between trial arms (Figure 2). |
|  | Similarity of baseline characteristics across trial arms (selection bias) | Low risk | No evidence of a difference in baseline characteristics between trial arms (Table 1). |
|  | Blinding of participants and personnel (performance bias) | High risk | Trial was unblinded |
|  | Blinded outcome assessment (assessor bias) | N/A | No outcome assessors (participants completed questionnaires). |
|  | Incomplete outcome data (attrition bias) | High risk | Amount of missing data was enough to induce bias in intervention effect estimate. |
|  | Similarity in attrition across trial arms (attrition bias) | Low risk | Similar proportion of participants were lost over follow-up (10% and 15%). |
